# The risk of thiamine deficiency in common eider ducklings increases when females consume large prey

**DOI:** 10.1371/journal.pone.0356777

**Published:** 2026-09-16

**Authors:** Maciej J. Ejsmond, Anna Ejsmond, Marc M. Hauber, Jón Einar Jónsson, Andreas M. Waser, Samuel Hylander

**Affiliations:** 1 Institute of Environmental Sciences, Faculty of Biology, Jagiellonian University, Krakow, Poland; 2 Centre for Ecological and Evolutionary Synthesis, Department of Biosciences, Faculty of Mathematics and Natural Sciences, University of Oslo, Oslo, Norway; 3 University of Iceland’s Research Center at Snæfellsnes, Stykkisholmur, Iceland; 4 Centre for Ecology and Evolution of Microbial Model Systems (EEMiS), Linnaeus University, Kalmar, Sweden; 5 Alfred Wegener Institute, Helmholtz Centre for Polar and Marine Research, Wadden Sea Station Sylt, List, Germany; University of Illinois, UNITED STATES OF AMERICA

## Abstract

Episodic mass mortality due to thiamine (vitamin B1) deficiency is a major threat to marine top-consumers worldwide. Recent reports have shown that thiamine deficiency can lead to high mortality rates of common eider (*Somateria mollissima*) ducklings in the Baltic population. It is unclear whether the drastic reduction in offspring recruitment is linked to the changes in egg thiamine levels. This would have an additional negative impact on the Baltic population of the common eider, which is mainly driven by increased predation pressure. As thiamine concentration decreases with the size of organisms at the interspecific level, we investigated whether the consumption of larger individuals of the dominant prey species may have a detrimental effect on adults and egg thiamine levels. We modelled female thiamine status in the life history perspective of a capital breeding sea duck, which accumulates body reserves prior to the nesting season. Thiamine uptake by model females was based on the empirical analysis of the thiamine concentrations in blue mussels *Mytilus edulis trossulus*, a predominant species in the diet of eiders. The model predicted that consumption of larger prey reduced thiamine levels in females and eggs, as thiamine concentration decreased with mussel size. Mechanisms of thiamine excretion were more important than seasonal variation and other life history determinants in shaping the egg thiamine levels in response to consumed prey size. The model also predicted higher levels of thiamine in the eggs of females that reduced their clutch size in response to changes in breeding phenology. These results suggest that the variation in thiamine concentration within prey species in relation to body size, is an important determinant of thiamine balance in top consumers. A reduction in clutch size is expected to increase offspring quality in females with poor thiamine status. Importantly, the consumption of large prey may predispose consumers to limitation of essential metabolic micronutrients.

## Introduction

Episodic mass juvenile mortality (early mortality) of marine top-consumers caused by thiamine (vitamin B1) deficiency is a serious conservation problem affecting ecosystems worldwide [1]. The deficiency-driven early mortality is accompanied by symptoms of muscle paralysis, neural dysfunction, and impaired locomotion, which prevent the body from functioning effectively and can cause death [2–5]. This is because thiamine plays a central role in several pathways that are key for the metabolism of amino acids, sugars, fatty acids, and nucleic acids [6,7]. This problem has mainly been reported in top consumers, such as salmonids, associated with the pelagic part of aquatic food webs including taxa in Baltic Sea, the Great Lakes, New York Finger Lakes, and California’s Central Valley [2,8–12]. Recent episodes with high mortality rates of juveniles in the Baltic Sea population of common eiders *Somateria mollissima* [5,13], a flagship benthic feeder in the northern hemisphere, raise concerns that the causes of thiamine deficiency, which are still poorly understood, are not necessarily restricted to the pelagic part of marine food webs [14].

**Fig 1:**
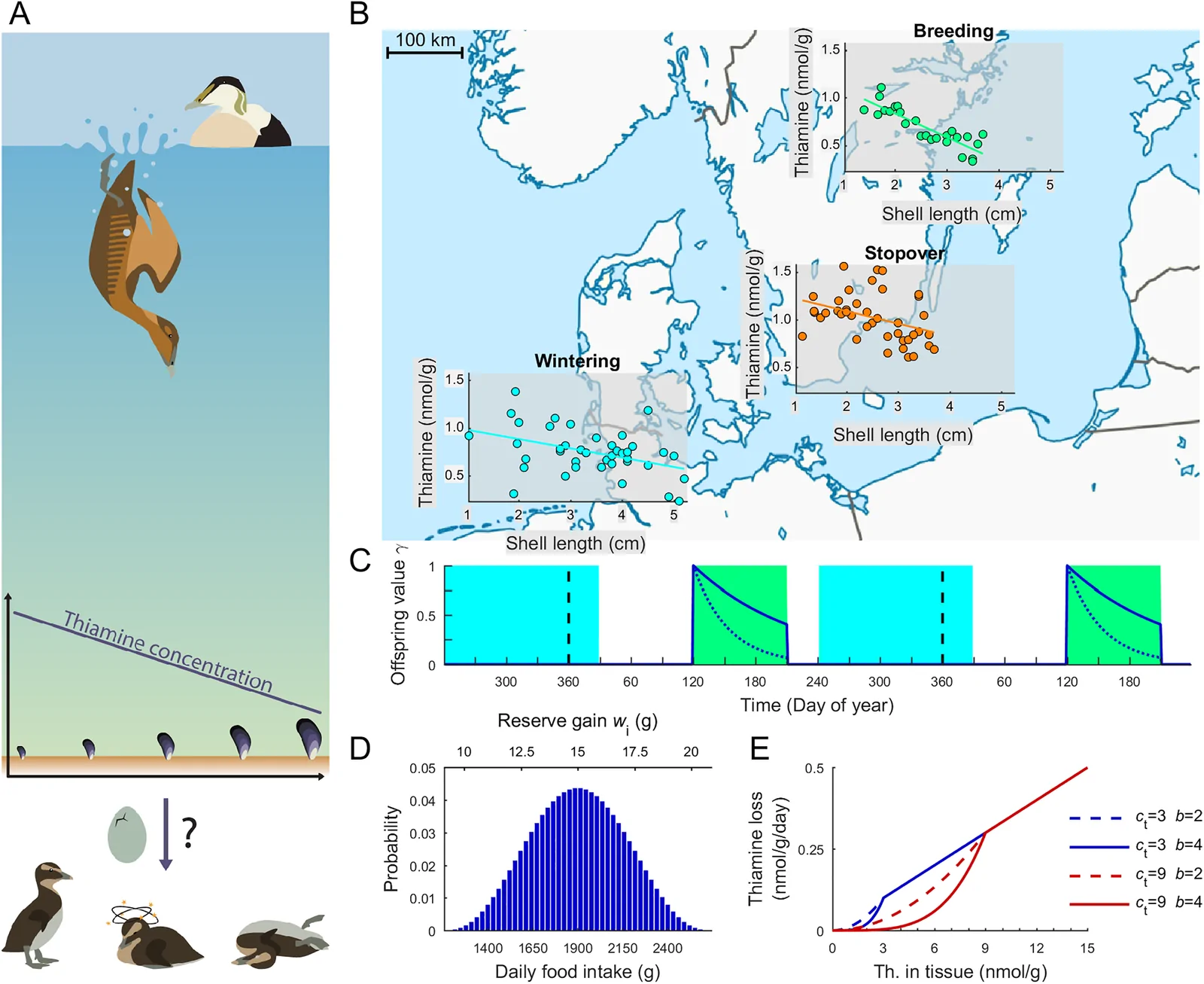
Thiamine levels in Baltic blue mussels and the model assumptions. (A) Schematic representation of the hypothesis tested in the paper. The preference for large prey, due to its low mass-specific thiamine content, increases the risk of thiamine deficiency in the offspring. Illustration by Marc M. Hauber. (B) The concentration of thiamine in the soft tissues of blue mussels, as a function of mussel size, sampled at geographical locations corresponding to the wintering grounds, migration stopover and breeding grounds of the Baltic population of common eiders. The background map shows the southern part of the Baltic Sea (Wikipedia, “An unlabeled map of Europe”, en.wikipedia.org – data from Natural Earth repository, http://naturalearthdata.com). (C) Outline of seasonality in the modeled environment. Periods of obligatory presence at the wintering grounds (light blue rectangles) are shown and then with temporal changes in the offspring value *γ*, i.e., chance to recruit to the next generations (green rectangles). The two alternative scenarios of offspring value are shown for *m*_d_ = 0.01 (solid blue line) and 0.03 (dotted blue line) (cf. Eq. 1). The end of the calendar year is shown by thick dashed lines. (D) Probability distribution of daily mussel consumption with the corresponding female reserve gains. (E) Four exemplary scenarios of thiamine excretion rates set by the shape parameters given in the legend: *c*_t_ (the threshold level above which only passive filtration is relevant) and *b* (the parameter defining the intensity with which active reabsorption contributes to the total excretion rate).

The reports of thiamine deficiency in the Baltic population of common eiders [3,5,13,15] add to the conservation concerns about the species, fueled by the drastic decline estimated to be more than one third of the population number in less than three decades [16,17]. Increasing predation pressure has been suggested as the main cause of the decline in the common eider population [18–21], but it is unclear whether thiamine deficiency could contribute to the negative trend, e.g., [5]. This raises general questions about the factors affecting the thiamine intake in this benthic feeder, cf. [22]. Common eiders in the Baltic population depend heavily on a single benthic species, the blue mussel (species complex; *Mytilus edulis trossulus* [23–25], which dominates their diet [26,27]. The concentration of thiamine in organisms decreases as species within the food web get larger, from single-celled organisms to piscivorous fish, through zooplankton and planktivorous fish [14]. The causes of the trophic dilution of thiamine with size of organisms in the food webs are not fully understood, but thiamine availability constrains the rate of metabolic processes, because thiamine diphosphate acts as a cofactor for enzymes in the Krebs cycle in mitochondria [6,7]. Dietary thiamine requirements are directly related to metabolic rate and body size [28,29], as metabolic rate scales allometrically, i.e., slower than proportionally, with body size across life forms [30]. This decrease is also observed within a species, e.g., Atlantic cod (*Gadus morhua*), where thiamine concentrations and metabolic rate decreases with size [31,32]. It is unclear whether the concentration of thiamine depends on the size of the blue mussels and, if so, whether the size of the mussels consumed affects the ingestion rate of thiamine in common eiders. Measurements of the mass-specific metabolic rate in blue mussels at mitochondrial level, i.e., a direct proxy of thiamine diphosphate demand, show a decrease with body size [33,34]. Hence, we hypothesized that the thiamine levels decrease with mussel size, and that consuming large blue mussels by common eiders, may alter thiamine intake with diet (Fig 1A).

Here we investigate whether females’ consumption of different sizes of the dominant prey species affects thiamine levels in the produced offspring (see Fig 1A). Our considerations are based on life history modelling and empirical analysis of thiamine concentrations in blue mussels from wintering, stopover and breeding grounds for the Baltic eider. The phenology of migration and reproductive strategy in the model resulted from fitness optimization under life history trade-offs related to the timing of nesting and the time spent building body reserves for reproduction. Our work tested whether a shift in the size of consumed prey towards larger mussels could significantly reduce the thiamine concentration in the produced eggs. In the model, we investigated the relative importance of physiological mechanisms of thiamine excretion and life history determinants in mediating the relationship between consumed prey size and thiamine levels in eggs. We also provide one of the first assessments of the size dependence and spatial variability of thiamine levels in blue mussels in the Baltic Sea.

## Materials and methods

### Empirical analysis of thiamine concentration in blue mussels

**Fig 2:**
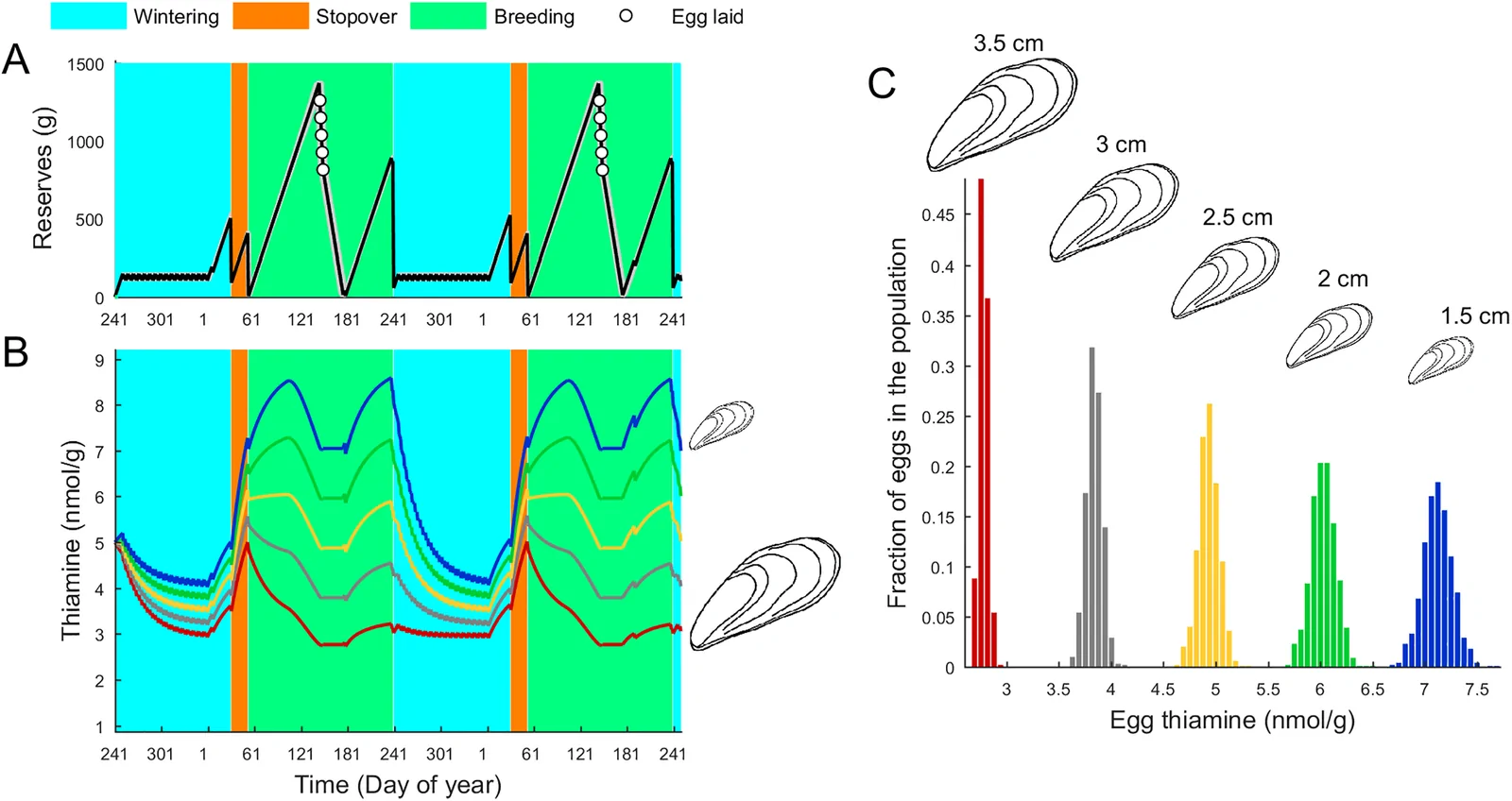
Dynamics of body reserves and thiamine tissue concentrations in populations of females with different prey size preferences. (A) The seasonal dynamics of female body reserves, timing of migration and nesting that maximize fitness. The solid black line represents a model female exposed to average food intake, while the shaded area around the thick black line represents variability between individuals due to stochastic food intake. (B) Thiamine concentrations in the body of females with different preferred prey sizes, ranging from 1.5 to 3.5 cm of the blue mussels shell length. The colors of the thick lines in B and C match. (C) Thiamine levels in the eggs produced by females in the population at the start of incubation. The distributions show the intrapopulation variance in egg thiamine levels, while the colors represent the preferred prey size (see legend). (A-C) The birth time dependent offspring value *γ* was determined by *m*_d_ = 0.01, the rate of thiamine loss was set by the shape parameters *c*_t_ = 3 and *b* = 2, with other model parameters at their baseline values (cf. Eq. 1). (A-B) Panels show trajectories over the first two years of a female’s life, representative of the 20 years of life assumed in the optimization.

Blue mussels of different sizes were collected in wintering, potential stopover areas, and breeding grounds of common eider (Fig 1B). The sampling locations have been defined within geographic areas known to be used by the Baltic eider for wintering, stopovers and breeding, e.g., [26,35]. All sampling sites coordinates and measurements are available in S1 Appendix (Figs 1-2 in S1 Appendix). Samples collected in wintering sites came from Sylt (Northwestern Germany on the border to Denmark) and Lysekil (West coast Sweden; Fig 1B, Figs 1-2 in S1 Appendix). Blue mussels sampled from these locations were of equal size range and soft-tissue concentrations were similar (t = −0.9, df = 38, p = 0.4). Hence, they were pooled to ensure a large sample size in the wintering area assuming that the birds can move freely in this area. Likewise, the closely located sites in Kalmar were assigned to stopover grounds (Figs 1-2 in S1 Appendix). Aligning with previous studies on prey size selection by common eider [26,36,37], we sampled blue mussels with shell length between 9–52 mm. We then used 0.4–1.2 g of soft tissues for thiamine analysis. If a blue mussel’s soft tissues fell below or above this weight range, samples were pooled or subsampled (S1 Appendix). In total, we obtained 40, 41, and 25 samples for wintering, stopover, and breeding grounds, respectively. Thiamine concentrations were analyzed following the established protocol by [38], with minor modifications (for details see S1 Appendix and [39]). Statistical analyses were performed using R, version 4.5.1, [40]. The relationship between mass-specific thiamine concentration and site (wintering, stopover and breeding) and length of mussels was analyzed using the lm() function. The interactive effect between mass-specific thiamine concentration and length tended to differ among sites, although not significantly (interaction effect length x site, F_2,100_ = 2.5, p = 0.089). This interaction was therefore removed from the final model but site-specific slopes were utilized in the down-stream modelling to retain biological information. Model fit was evaluated using diagnostic plots of residual distribution with the DHARMa package and p-values were computed based on robust covariance matrix estimation, i.e., Wald tests [41].

### The model

#### Outline.

We modelled the seasonal thiamine dynamics in common eider females and produced eggs. Our model organism is a sea duck that breeds as a capital breeder, cf. [42], i.e., it accumulates a vast majority of the reserves during the pre-laying period after arrival to the breeding grounds, which are then used to cover the energetic costs of egg production and incubation. The research approach consisted of two steps. First, we used a framework based on dynamic state-variable modelling [43] to derive female strategies for timing of migration, reserve building and nesting that maximize fitness. The term ‘strategy’ is used to refer to the physiological and behavioral responses that have evolved through natural selection. Similar models of capital breeding sea ducks have been used to investigate climate-induced changes in breeding synchrony in eiders and the fitness response to stochastic foraging under climate change [44,45]. Next, after obtaining optimal life history strategies, we calculated the dynamics of thiamine concentration in females following these optimal strategies, with thiamine intake parameterized using data on thiamine content in mussels sampled in the Baltic Sea (Fig 1B). We followed this two-step approach for the sake of simplicity and because, unlike internal body reserves, thiamine cannot be long-term stored due to a short ca. 2.5 week half-life of the vitamin stored in liver [46]. The thiamine status of females and the thiamine-dependent quality of hatchlings were compared for different scenarios of consumed prey size by foraging females.

#### Life history, migration phenology, mortality risk.

The model was inspired by the biology of common eiders, with features of seasonality and the modelled environment set to represent the conditions faced by this species in the Baltic Sea area (see S1 Appendix for details). However, as populations of common eiders may display considerable variability in various aspects of migration timing and pace, breeding phenology, changes in reserve levels during migration, and clutch size, e.g., [26,35,47,48], we analyzed the sensitivity of the model to changes in these traits (see below). The life history of modelled females started at the end of August (*t* = 240) at the wintering grounds before its first nesting season (Fig 1C). The body of the female on day *t* consisted of the lean body mass *M*_L_, assumed to be 1100 g, plus reserves *S*(*t*), that could reach up to *S*_x_ = 1500 g; the maximum body mass can reach up ca. 2.6 kg with possible reduction of up to 40% of body mass due to reserves loss [36,49]. The model set the date by which females were required to remain on their wintering grounds until the beginning of February, cf. [26,35], with *t* = 30 day of the year assumed in the results presented below. We also considered scenarios with different arrival times to the breeding grounds, in order to model the diversity of common eider migration [26], as well as the recent change in the predation regime affecting some Baltic populations, whereby females postpone the onset of nesting and produce smaller clutches [19]. Females migrate from wintering to breeding grounds with a stopover (Fig 1A), and migrate back after nesting season, with a loss of body reserves equivalent to energy expenditure during 2 days of flight (see S1 Appendix for details). Migration back to wintering grounds was modelled without stopover for the sake of simplicity. As the energetic cost of flight in common eiders increases rapidly with body mass [50], we assumed that females with high reserves would reach their destination close to or in a lean body condition (see S1 Appendix). However, we also modelled females arriving at the breeding grounds with high reserves, which increased clutch size, but this did not affect the main conclusions of our study regarding the effect of the size of consumed prey on differences in egg thiamine levels (Fig 5 in S1 Appendix). The timing of migration (i.e., the day of the year at which migration started) from wintering grounds to stopover *α*_1_, from stopover to breeding grounds *α*_2,_ and from breeding to wintering grounds *α*_3,_ evolved in our model in order to maximize fitness.

On the breeding grounds, females with sufficient body reserves *S* could initiate egg laying, produce up to *n*_x_ = 5 eggs with each egg weighting *E* = 110 g (see S1 Appendix for details), though the model predictions did not change for *n*_x_ = 6 (Fig 12 in S1 Appendix). Females stopped feeding and incubated the clutch for *I* = 26 days, which depleted *c*_m_ = 800 g of their reserves [49,51]. Because we modelled capital breeders, females that foraged for longer before nesting were able to produce more eggs, but as in other birds, delaying nesting reduced the probability of offspring recruitment [52–54]. The timing of hatching determined the probability of offspring recruitment *γ*(*t*), ‘offspring value’ hereafter, which was maximal at the beginning of the nesting period starting early May (*t* = 120) and decreased exponentially until the end of July (*t* = 210). The decrease in offspring value *γ*(*t*) was given by


$$\begin{array}{c} \gamma (i)=e^{-m_{\text{d}}i} \end{array}$$ (1)

with *i* matching the day of breeding period and the rate of decrease of the offspring value set by the parameter *m*_d_ (Fig 1C).

The daily mortality rate of adults was parameterized to reflect life expectancies of common eider females, with incubation being the riskiest behavioral action taken (e.g., [55], see details in S1 Appendix). However, our findings on the relationship between the size of prey consumed and egg thiamine levels did not change when we considered various combinations of background mortality rates (see Figs 6, 8 in S1 Appendix). The survival of model females was reduced if they carried extensive reserves (see S1 Appendix), as ducks with high body reserves and reduced mobility are more vulnerable to predation [50,56]. Females in lean body condition at breeding grounds, and those with reserves *S*(*t*)<*S*_r_ with *S*_r_ set to 100 g [57] at other geographic locations, starved with a 20% reduction in survival (see S1 Appendix). While some studies have suggested a positive relationship between reserve levels and the yearly survival of common eiders [16,52], we also analyzed whether the assumed starvation threshold *S*_r_ affects the response of egg thiamine levels to the size of the prey consumed (Fig 7 in S1 Appendix).

#### Stochastic food intake and rates of thiamine intake and loss.

To model thiamine intake, we parameterized the daily dietary intake of female eiders in terms of blue mussel mass using data on daily reserve gain and body maintenance costs. The model female staying at wintering, stopover, or breeding grounds, could rest and use reserves to sustain resting metabolic rate (RMR), thermoregulation, etc. by utilizing *h* = 37.5 g of reserves per day (see [49] and S1 Appendix). Alternatively, it could forage and build up reserves according to *S*(*t* + 1)*=S*(*t*)+*w*_i_, with a stochastically varying daily reserve gain *w*_*i*_ and the average gain *w* of 15 g per day [58,59]. The reserve gain was modelled with a β distribution of normal-like shape (Fig 1D, and [45]), with the average gain *w* corresponding to daily consumption of 1.9 kg of mussels [36]. However, the flesh-to-shell mass ratio of blue mussels can vary geographically, affecting net energy intake [60]. We also modelled scenarios with various conversion efficiencies of consumed food into reserves (see Fig 11 in S1 Appendix). Below, we present the results of five scenarios in which females foraged on mussels of fixed lengths: 1.5, 2, 2.5, 3 and 3.5 cm.

Our work investigates the effects of consumed prey size on thiamine concentrations in the offspring produced. We assumed that thiamine egg levels reflect the thiamine concentration in the body of the female at laying initiation. Although this assumption cannot be verified in bird models, it is consistent with changes in thiamine tissue status in the Baltic population of Atlantic salmon (*Salmo salar*), in which the concentrations of thiamine in muscles and gonads before spawning are close to being directly proportional to each other [61]. The main focus of our work was to investigate the relative differences in egg thiamine levels between scenarios that differ in consumed prey size. Because our model focused on thiamine levels at the time prior to incubation, it does not consider the release of thiamine stored in the liver and the reduction in organ mass in incubating females. These processes may slow or even prevent the loss of micronutrient levels during incubation. For simplicity, thiamine levels in incubating females were kept the same as at the time of laying cessation.

The temporal dynamics of thiamine in our model depended on the balance between micronutrient intake and loss. Thiamine intake in the model was an outcome of daily food intake, thiamine bioavailability set to 50% (cf. [14], and S1 Appendix), concentration in mussels at a given geographical location, and assumed scenario of consumed prey size (Fig 1B, D). To calculate thiamine input rates in the model, we recalculated the concentration of thiamine per mussel soft tissues (Fig 1B) to the concentration per 1 g of mussel consumed, assuming that thiamine levels in the shells and free water inside the shells were negligible (S1 Appendix). Thiamine loss was determined by renal excretion, with active reabsorption at low plasma concentrations [62,63]. An increase in plasma concentration reduces the relative capacity of transporters for reabsorption [64]. Therefore, at low thiamine concentrations, where active reabsorption significantly reduces the rate of excretion, an increase in plasma concentration leads to an acceleration of the rate of loss [64,65]. In the model, the thiamine loss rate *k* was composed of a power and a linear function (cf. [64]) given by


$$\begin{array}{c} k=\left\{ \begin{array}{c} @lac^{b},\text{ for }c<c_{\text{t}}\\ k_{\text{x}}\frac{c}{c_{\text{x}}},\text{ for }c\geq c_{\text{t}} \end{array}\right. \end{array}$$ (2)

with maximal excretion rate *k*_x_, coefficient *b* and $a=\frac{c_{\text{t}}k_{\text{x}}}{c_{\text{x}}c_{\text{t}}^{b}}$ that determined the shape of the function for renal active reabsorption, and the *c*_t_ matched concentration threshold above which only the passive transport determines the rate of excretion (Fig 1E).

There are only a few estimates of the egg thiamine threshold levels at which detrimental effects in common eider ducklings occur and suggested to fall somewhere around 5 nmol/g [3,15]. The predicted by the model thiamine concentrations in the eggs fall below and above this value, which suggest assumptions regarding thiamine intake and excretion rates have been realistically combined with a general model of the life history of common eiders. However, we believe that our simplified framework lacks the predictive power to draw definite conclusions about the relationship between the exact size of consumed prey and the likelihood of thiamine deficiency-induced mass mortality. This is because our aim was to create a flexible model that captures the diversity of life histories and migration phenology of various common eider populations.

**Fig 3:**
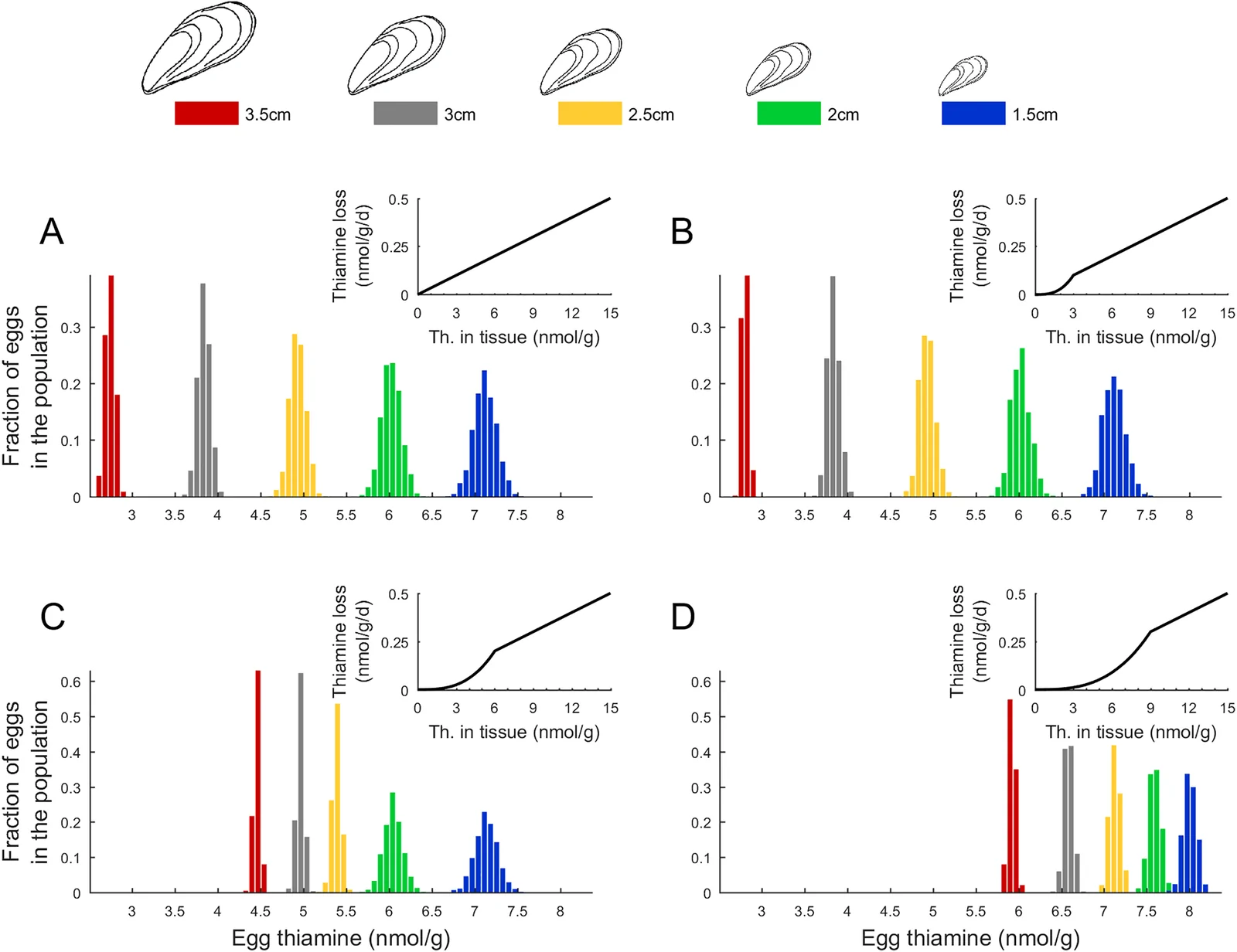
Egg thiamine in scenarios that differ in the preferred prey size, and physiological basis of thiamine loss. (A-D) Thiamine levels in eggs laid by females are shown for scenarios in which females differed in the preferred prey size (see legend). Panels from A to D show scenarios with increasing threshold *c*_t_ of thiamine levels in tissues, above which passive filtration was the dominant physiological mechanism determining thiamine loss rates with *c*_t_ = 0 (A), *c*_t_ = 3 (B), *c*_t_ = 6 (C), and *c*_t_ = 9 (D). The loss of efficiency of active reabsorption with increasing tissue thiamine levels was kept the same in all presented scenarios and set to *b* = 3 (cf. Eq. 2). The birth time dependent offspring value *γ* was determined by *m*_d_ = 0.01 (cf. Eq. 1), with other parameters set to their baseline values.

In the model, we calculated thiamine levels per female lean body mass plus the reserves required for egg production, excluding the pool of reserves used to cover the costs of migration, RMR and incubation. This exclusion of reserves used for needs other than egg production, reflects the fact that females deposit thiamine in the egg yolk and the vitamin is not allocated to body fat reserves, since it is a water-soluble vitamin, and not a fat-soluble one [7]. However, the conclusion of our work did not change when thiamine levels were calculated per lean body mass plus the mass of all reserves carried by the female (Fig 3 in S1 Appendix). Modelling was performed with MATLAB [66]; see S2 Appendix for the code of the program.

## Results

Soft-tissue thiamine concentrations in the dataset ranged from 0.2–1.6 nmol g^-1^ (Fig 1B, Fig 2 in S1 Appendix). Thiamine mass-specific levels in the soft tissues of blue mussels showed a strong decrease with size in all three sampling sites, i.e., wintering, stopover and breeding (Fig 1B, Fig 2 in S1 Appendix). Hence, there was a significant effect of length on mass-specific thiamine concentration (F1,100 = 29.2, p < 0.001) and also a significant effect of site (i.e., wintering, stopover and breeding; F2,100 = 18.7, p < 0.001).

**Fig 4:**
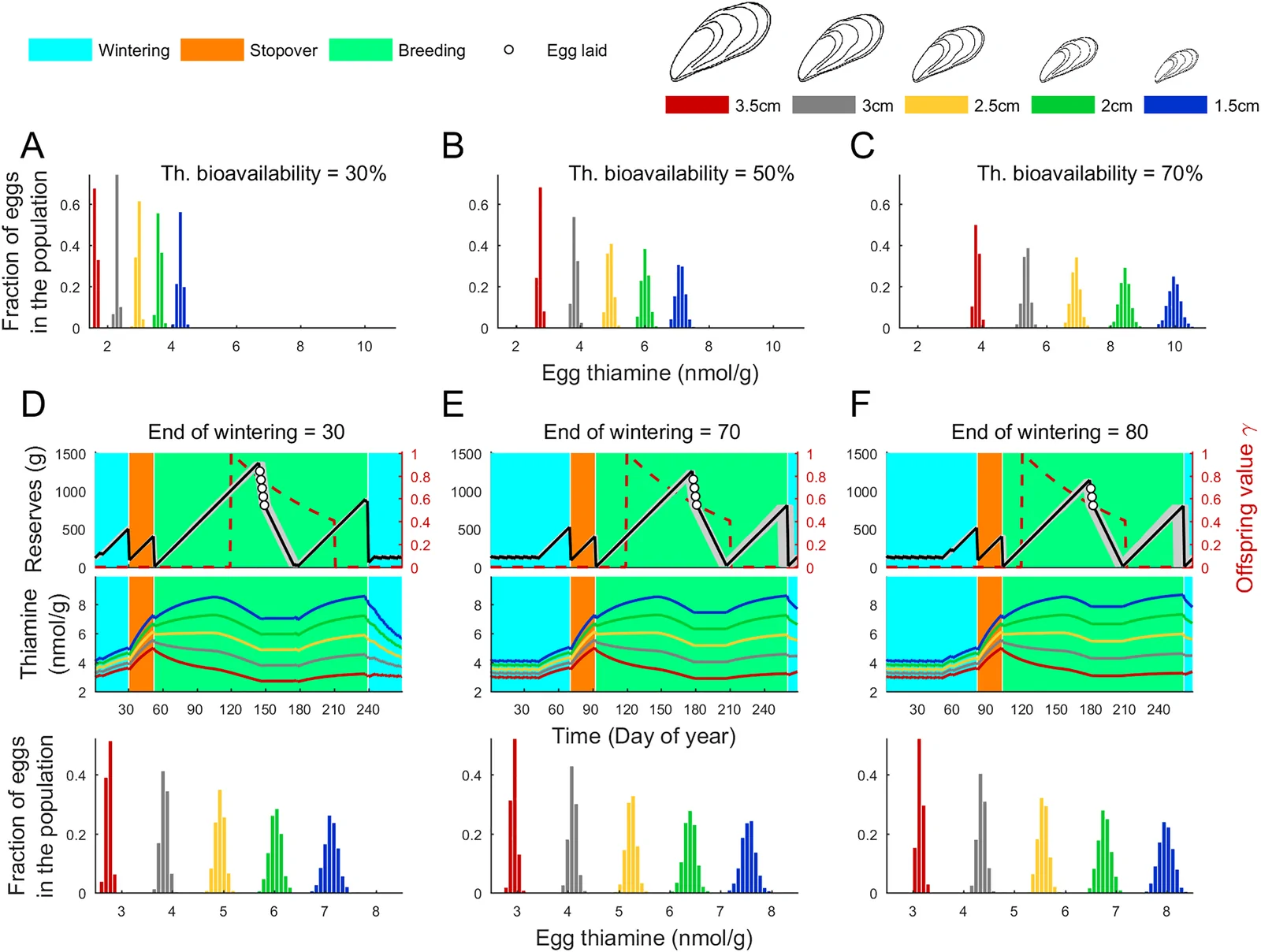
Egg thiamine in scenarios with females differing in preferred prey size and physiological and seasonal constraints. Thiamine levels in eggs laid by females differing in the preferred prey size (see legend), and thiamine bioavailability (A-C) or duration of obligatory wintering (D-F). (A-C) Egg thiamine at three different levels of thiamine bioavailability (given at the top of the panels). (D-F) Scenarios differing in the day obligatory overwintering ended (given at the top of the panels) and migration to the stopover was possible, with *t* = 30 assumed in the main results. The later the arrival at the breeding grounds, the smaller the clutch size and the later the onset of nesting of females in the modelled population (see reserves level trajectories from D to F). Thiamine bioavailability was set to its baseline value of 50%. (A-F) Other parameters were set at their baseline values, and the offspring value *γ* (panels D-F with dashed line) was determined by *m*_d_ = 0.01 (cf. Eq. 1).

The life history of the model females resembled the life cycle of the Baltic common eiders, with accumulation of body reserves preceding migration, egg laying and incubation (Fig 2A). Under the baseline set of parameters, which outlined the seasonality of the environment and the birth-time dependent prospects of the produced offspring (see Fig 1C), the consumption of large prey led to up to 50% reduction of egg thiamine (Fig 2B, C). The levels of thiamine in the eggs, were particularly sensitive to consumed prey size when active reabsorption did not mitigate the rates of thiamine loss, or when rates of loss were decreased at low thiamine tissue levels (Fig 3A-3B). In scenarios where active reabsorption reduced thiamine loss rates over a wide range of thiamine concentrations in tissues, consumed prey size had a relatively weaker effect on egg thiamine levels (Fig 3D). Similarly, low thiamine bioavailability led to a smaller difference in egg thiamine levels between scenarios with different consumed prey size (Fig 4A-4C). However, even in these scenarios females feeding on large prey produced eggs with considerably lower thiamine levels than females feeding on small prey (Fig 3C-3D, Fig 4A).

In scenarios where females arrived at the breeding grounds in a lean body condition (scenarios presented below), clutch size decreased with postponement of arrival and nesting onset because the time available to build up reserves was shortened (see Fig 4D–4F and Fig 4A-C in S1 Appendix). Similarly, in scenarios in which females in mild winters arrived at the breeding grounds with reserves, clutch size also decreased with postponement of nesting onset (Fig 5 in S1 Appendix). Conversely, the faster the decrease in offspring value (γ), the earlier females started to nest and the fewer eggs they laid (see Fig 4D–F in the S1 Appendix). This was because the rapid deterioration of offspring prospects with hatching time imposed a trade-off between nesting earlier with a lower level of reserves and producing offspring with a high fitness contribution, or starting to nest later with a higher level of reserves and producing more offspring, albeit with a relatively low fitness contribution. However, regardless of the phenological circumstances, level of thiamine in eggs always increased when clutch size was reduced (see the histograms in Fig 4D–F and Figs 4-5 in S1 Appendix). Similarly, the main prediction of our study, which was that egg thiamine levels would decrease alongside the size of the prey consumed, was relatively insensitive to variation in nesting phenology and seasonal time constraints, assumed mortality rates, migration costs, maximal clutch size, stochastic food intake, energy conversion and starvation scenarios that represent interspecific variability of life histories in common eider (Figs 4-12 in S1 Appendix).

## Discussion

Our work shows that the size of consumed prey is expected to affect the micronutrient balance of consumers. The decreasing concentration of micronutrients with prey size is not considered in classical optimal foraging theory, cf. [67,68], which links foraging time and energy income-expenditure in a conceptual framework used to investigate fitness-related consequences of animal feeding behavior. Consumption of large prey by consumers may lead to a reduction in the intake of essential metabolic micronutrients. This is reflected in the decreasing mass-specific thiamine levels with organism size found in blue mussels, but also in other species including zooplankton and fish, see [14,32,69,70]. From a functional perspective, the concentration of thiamine, a key coenzyme in the catabolism of sugars and amino acids [6,7], and other essential metabolic micronutrients is expected to scale with the rate of aerobic metabolism. While there are many reasons why metabolic rate increases allometrically with body size [30,71,72], it is not surprising that thiamine levels also decrease with increasing prey body size. The relationship between biochemical cofactors that mediate metabolic processes and organism size is the key element of the present study.

The concentration of thiamine decreases with the body size of organisms at interspecific levels, e.g., [14]. Our results show that this decrease is significant also in case of single-species prey with considerable variation in body size, see [32,70]. Hence, consumption of relatively large prey may predispose top consumers to limitation of essential micronutrients. However, the degree to which micronutrient intake depends on the prey size is likely to be variable and mediated by several aspects of trophic interactions. For example, consumers that are unable, or for other reasons, do not selectively consume different body regions of prey are more likely to be affected by micronutrient limitation caused by consumption of large-prey. This is because many consumers feed on specific regions of prey in a way that is driven by the nutrient-specific content of body parts [73,74]. Similarly, certain tissues with high metabolic activity (nervous system, muscles, liver) may contain much higher levels of metabolic enzyme cofactors than other organs (e.g., supportive tissues). However, animals such as common eider and predatory fish rather ingest whole prey items, reducing the capacity to gain micronutrients from consumption of micronutrient-rich body regions.

Elevated predation-induced mortality of incubating females and drastic reduction of recruitment in the first year of life are likely the predominant causes of the recent negative trends in the Baltic population [16,18–21]. In Baltic eiders, individual differences in brain size and female behavior are associated with variation in nesting success under predation pressure [75,76]. Specifically, in more dangerous years large-headed females breed later, which increases chances of brood survival, and require less time to form anti-predator brood-rearing coalitions [76]. It has been also shown that females with large brains may feed on considerably larger prey [27]. Consumption of large prey by females with large brains in certain geographic regions may affect their thiamine intake, given that the level of this micronutrient decreases with blue mussel body size. It is unclear to what extent episodes of high juvenile mortality caused by thiamine deficiency contribute to negative population trends in common eiders. In other organisms, however, such as lake trout (*Salvelinus namaycush*), thiamine deficiency has been shown to decrease predator avoidance in juveniles and increase overall mortality rates due to predation [77,78]. Therefore, future studies are needed to investigate whether thiamine deficiency and predation interact to affect juvenile recruitment to the next generation at a population level. Similarly, screening for thiamine concentration should be included in future studies investigating predation rates in Baltic common eiders.

Thiamine deficiency has been shown to occur in a number of vertebrate taxa [3,8,9,15,79,80], but the mechanisms leading to deficiency in the wild are not well known. Balk et al. [15] suggested that the quantitatively low concentration of thiamine in blue mussels alone would be enough to cause deficiency. The concentration of thiamine in blue mussel is indeed several times lower than those found in common eiders [15]. Recent population declines observed in Baltic common eider have been ascribed to recruitment failure [16,17]. Our study shows that relatively small shifts in consumed prey size can significantly affect the thiamine concentration in the produced offspring. Hence, the consumption of large prey by females, could lead to a further deterioration of egg thiamine levels. Conversely, blue mussel size in the Baltic Sea has decreased slightly by approximately 1 mm in shell length over the last decades [81], which might to some extent offset the reduction in thiamine intake selection of large prey. However, the reduction in mussel size reported by Åkermark et al. [81] is at least an order of magnitude smaller than the typical size variation of blue mussels consumed by common eiders [26,37]. It has been hypothesized that in salmonids, thiamine deficiency develops either due to consumption of prey items containing high thiaminase activity, which breaks down thiamine in the gut before uptake, or as a consequence of consumption lipid-rich prey items [9,82,83]. These prey items contain easily oxidized lipids and once catabolized would lead to a reduction of thiamine in somatic tissues given that thiamine show some antioxidant capacity [82,83]. It is not known if these two mechanisms would be valid in the case of mussel-eating ducks, but bivalves have been shown to exhibit some thiaminase activity [84]. Similarly, it is not well understood whether the relationship between thiamine concentration and shell size in blue mussels remains consistent across different seasons, despite some observed variation [15]. It could be hypothesized that the periodic investment of vitamins during the development of reproductive tissues in breeding individuals could alter this relationship [85,86]. However, in other organisms such as Atlantic salmon, the concentration of thiamine in the gonads decreases as spawning approaches [39]. Therefore, future studies should examine this relationship across the seasons and reproductive states of blue mussels and other organisms.

While it is known that mortality episodes in offspring of marine top-consumers are often associated with thiamine deficiency [2,3,5,8,11,13], relatively little is known about the female behaviors or adaptations that mitigate the negative fitness consequences of mass juvenile mortality. In years with mortality episodes in Atlantic salmon, there is no clear reduction in the number of eggs produced by females [87,88]. In contrast, thiamine deficiency in herring gull has been reported to be associated with a reduction in clutch size [3]. Our work shows that the reduction of clutch size by about 40%, due to changes in breeding phenology but not thiamine input levels, increased the thiamine egg content by up to 20% (cf. Fig 4D-F, Figs 4-5 in S1 Appendix). This may suggest that the reduction in clutch size of common eiders in years with thiamine deficiency and high juvenile mortality is an adaptive response by females that increases average recruitment at the expense of the number of offspring produced. Although, these potential effects need to be verified empirically. We suggest that the lack of a similar reduction in fry production by salmon females is due to differences in feeding intensity and rate of egg formation prior to fry release between these species. Common eider females feed intensively until incubation, whereas salmon females often starve for several months in the pre-spawning period. Moreover, egg production and laying in eiders takes several days, whereas egg formation in salmon takes 4–5 months [26,89]. We suggest that the temporal differences in follicle recruitment and growth between the two species may enable eider females, cf. [90], but not salmon females, to reduce offspring production in response to changes in maternal thiamine levels before breeding. This would also suggest that foraging strategy during the pre-breeding period is an important determinant of the extent to which females can offset the negative consequences of their poor thiamine status on offspring recruitment.

Modelling of micronutrient allocation to somatic and reproductive tissues in a life history context requires considerations to include the nonlinear dynamics of intake and excretion, as well as changes in tissue mass. This complicates model structure, see, e.g., [61], and forced us to simplify ecological aspects of prey size selection. Hence, our model did not investigate how egg thiamine levels would depend on more realistic prey size selection behaviors. For example, in common eiders shell processing constraints digestion, with the ratio of flesh to shell mass playing a key role in the selection of prey size [60,91]. This may cause eiders to select smaller mussels than one could expect from only size of the prey. Similarly, mussels analyzed in our work were sampled over a relatively narrow period of the year, which precludes modelling of the effect of the seasonal changes in thiamine content in the prey. Hence, future model development should take into account mechanisms of prey size selection, and the potential seasonal variation of thiamine content.

In conclusion, this study shows that consumption of large prey size can lead to reduced thiamine levels in the offspring, or even deficiency. While the concentration of thiamine decreases with the size of organisms in marine food webs [14,32,70], our work suggests that the size-specific reduction in thiamine concentration at the intraspecific level is an important factor in the dynamics of essential metabolic micronutrients in consumers.

## Supporting information

S1 Appendix Model parameterization and sensitivity analysis. (PDF)

S2 Appendix Programme code. (PDF)
