# Supplementary material for: The risk of thiamine deficiency in common eider ducklings increases when females consume large prey
Source: PLoS One. 2026 Sep 16;21(9):e0356777. doi: 10.1371/journal.pone.0356777 (PMC13580947; doi:10.1371/journal.pone.0356777)
Supplement: Supplementary file 1 [file pone.0356777.s001.pdf]

S1 Appendix. Model parameterization and sensitivity analysis

Table of contents

1. Thiamine concentrations in blue mussels – empirical data analysis

1.1 *Sampling and preparation* **2**

1.2 *Thiamine analysis* **3**

1.3 *Raw data* **4**

2. Model parameterization - common eider life history

2.1 *Time constraints for migration* **7**

2.2 *Breeding grounds* **7**

2.3 *Mortality risk* **8**

3. Model parameterization - daily food intake and thiamine input

3.1 *Stochastic food gain* **8**

3.2 *Thiamine intake* **9**

3.3 *Key model parameters* **9**

4. Sensitivity analysis

4.1 *Alternative calculations of thiamine levels in female eiders* **10**

4.2 *Phenology of overwintering and breeding* **11**

4.3 *Background and starvation-driven mortality risk* **13**

4.4 *Migration costs* **16**

4.5 *Stochastically fluctuating food intake* **17**

4.6 *Food to reserves conversion* **18**

4.7 *Maximal clutch size* **19**

5. References **19**

1. Thiamine concentrations in blue mussels – empirical data analysis

1.1 *Sampling and preparation*

Blue mussels in the full size range of the taxon were sampled during June 2023 in three locations representing wintering, stopover, and breeding grounds of common eider. Wintering grounds were represented by blue mussels sampled outside of Sylt (Germany, 55.02864N, 8.43403E) and Lysekil (Sweden, 58.16056N, 11.26034E). Close to Kalmar (Sweden), blue mussels were sampled and then pooled from four locations (56.41464N, 16.22102E; 56.76937N, 16.49037E; 56.75687N, 16.52480E; 56.76360N, 16.50217E) representing stopover grounds. Lastly, blue mussels were sampled from breeding grounds outside of Stockholm (Sweden, 59.01247N, 18.50471E). All mussels were transported on dry ice to laboratory facilities in Kalmar where they were stored at -80°C. The length of each mussel was measured (1 mm precision) and mussels between 9 and 52 mm in length were selected for further analysis, aligning with previous studies on prey size selection by common eiders (Guillemette 1998, Hamilton et al. 1999, Waltho and Coulson 2015). To ensure that mussels of the whole size range present at each location were analyzed, we separated them into five length classes. Sylt mussels had already been categorized on site into four length classes before the transport to laboratory facilities. Hence, Sylt was presented by only four length classes. Blue mussels were left at room temperature until the shell had barely thawed and could be removed. The frozen core containing the soft tissue and frozen encapsulated water was put aside to thaw and weighed (0.001 g precision) as soon as all encapsulated water had melted. Thawed soft tissue weighing between 0.4 and 1.2 g, was considered suitable for analysis. If soft tissues weighed less than 0.4 g, soft tissue of similarly sized mussels was combined until sample contained more than 0.4 g. If mussels weighed more than 1.2 g, they were cut into pieces of maximum 5 mm, and a subsample of tissue was randomly selected. We intended to prepare five samples per size class per location. As small mussels often needed to be pooled, we obtained 40 samples for wintering grounds (Sylt: 16; Lysekil: 24), 41 samples for stopover grounds (Kalmar), and 25 samples for breeding grounds (Stockholm). Directly after processing, all samples were placed in 15-mL Falcon tubes and stored at -80°C until further analysis.

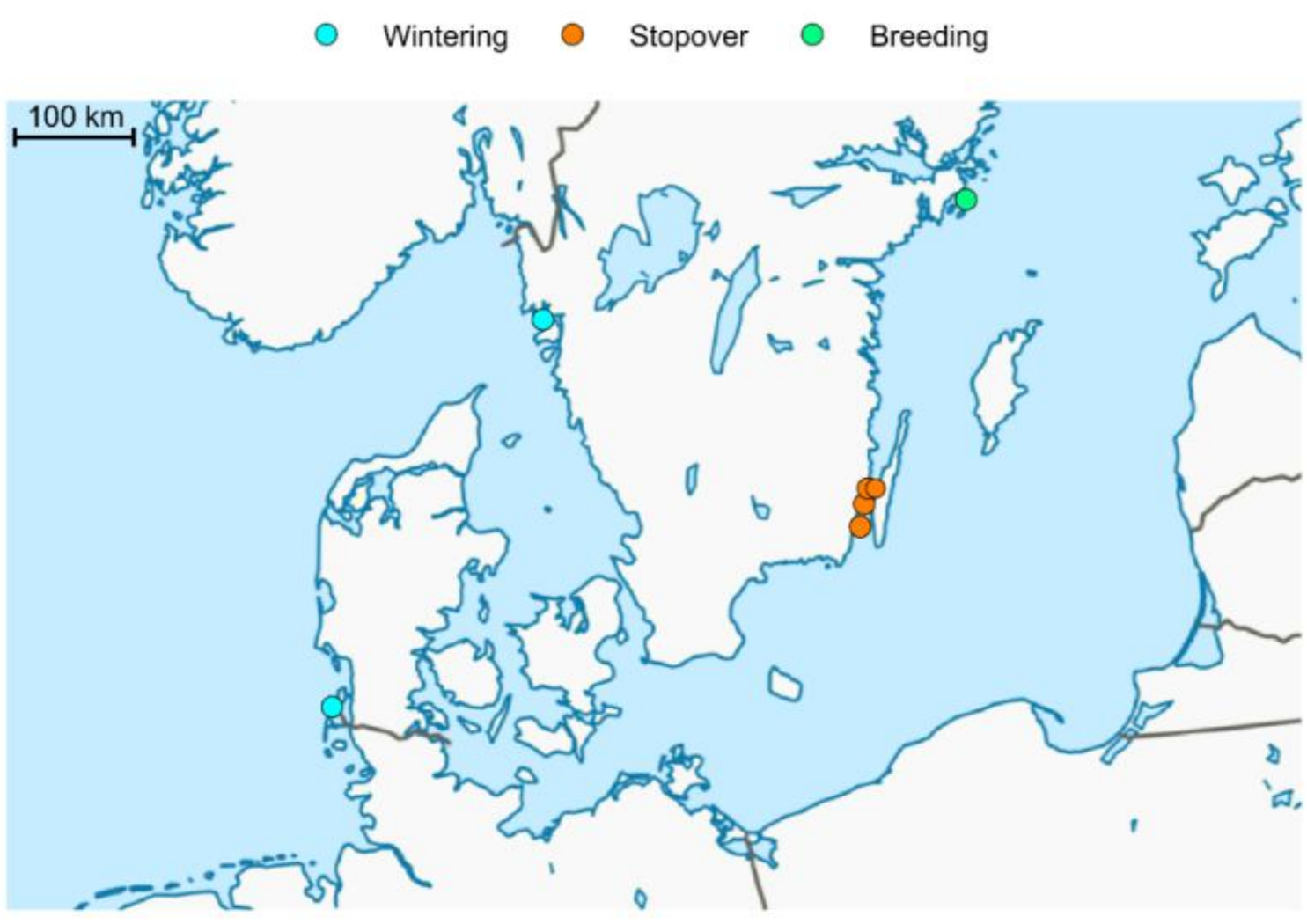

Fig 1. Sampling locations of blue mussels. Sampled mussels were analyzed for body size and thiamine concentration.

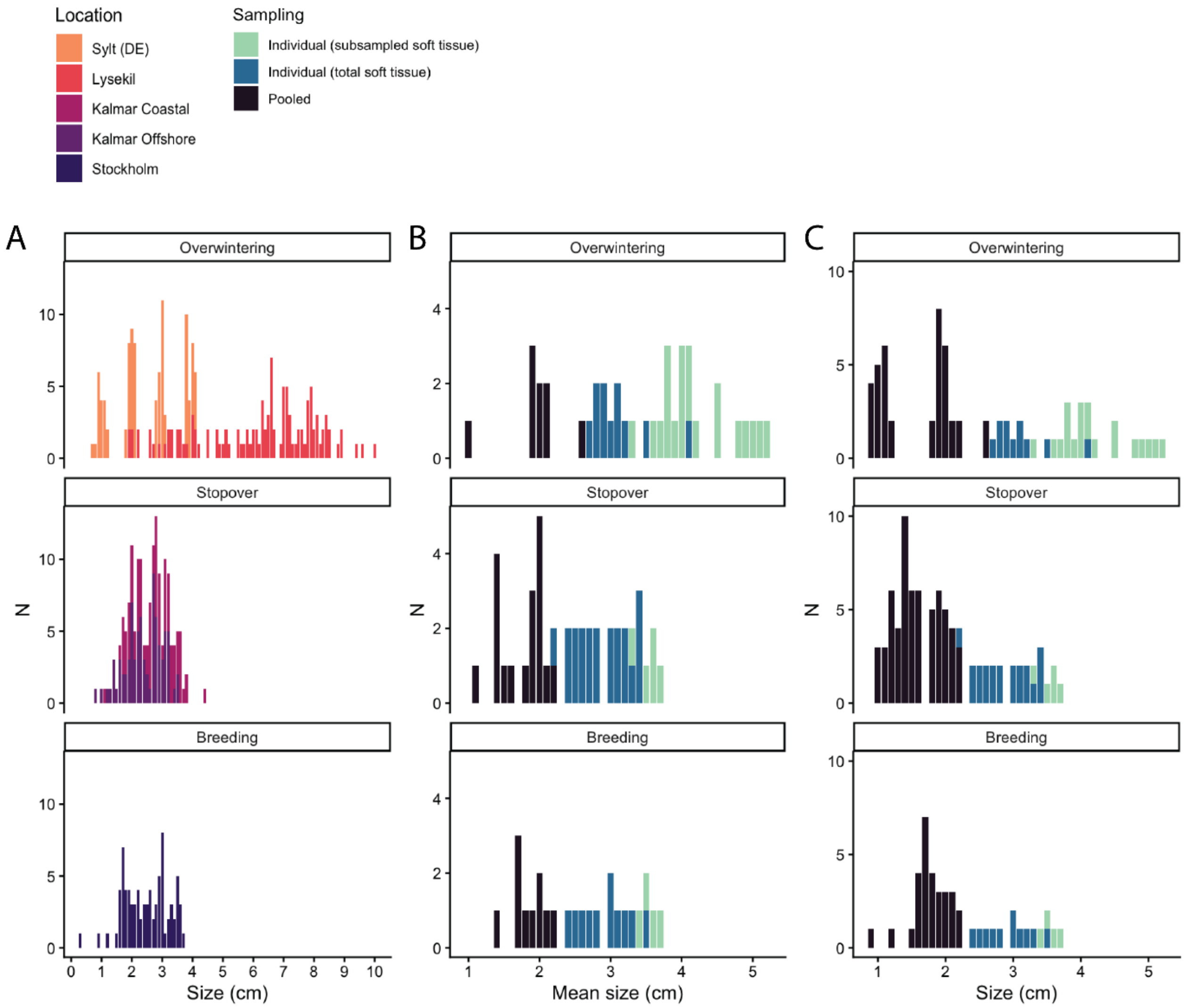

Fig 2. Histograms of size and number of blue mussels. (A) All blue mussels received from the different field locations. Note that blue mussels from Sylt were preselected in the field and so the presented size distribution does not reflect the natural size distribution. (B) Size distribution of the actual samples. If sample was pooled the mean size of the sample is shown. (C) Size distribution of all the blue mussels used for the thiamine analysis. Note the increase in small mussels in the pooled samples.

1.2 *Thiamine analysis*

Thiamine concentrations were analyzed according to Brown et al. (1998), with method modifications according to Vuorinen et al. (2002), Futia et al. (2017), Futia and Rinchard (2019). Blue mussel soft tissue samples were homogenized in 3 mL of ice-cold 2% trichloroacetic acid (TCA) by five stainless steel balls (Steelball Lysing Matrix) using the lysing system FastPrep-24 5G (MP Biomedicals) for 3 min at a speed of 6.5 m s^{-1}. After removing the lysing matrices, the samples were incubated in a water bath at 100°C for 10 min and placed on ice for additional 10 min. One and a half mL of 10% TCA was added, and samples were centrifuged at 14,000 × g for 15 min using the high-speed centrifuge Beckman Avanti J-HC. The samples' supernatant (1 mL) was washed four times with an equal volume of ethyl acetate:hexane (3:2 volume ratio), by inversion and removing of the upper phase. The lower phase (850 µl) was mixed with

150 µL of freshly made 0.1% K3Fe(CN)6 solubilized in a 1.2 M NaOH solution and was filtered through 0.45 µm PTFE/PP filters into glass vials. Standard solutions for the three vitamers of thiamine (free thiamine [TF], thiamine monophosphate [TMP], and thiamine diphosphate [TDP]) were subjected to the same procedure as the tissue samples except for the homogenization and the centrifugation steps. Standard solutions were prepared in 0.1 M HCl solution and aliquoted in a five-points standard series. Samples were analyzed using a Hitachi Chromaster HPLC system with a Hamilton PRP-1 column (5 µm particle size, 4.1 mm [I.D.] × 150 mm) protected by a guard column (10 µm particle size, 2.1 mm [I.D.] × 2.1 mm), equipped with a fluorescence detector (excitation wavelength 375 nm, emission wavelength 433 nm). The injection volume was 100 µL (flow rate 1 mL/min) with a runtime of 25 min. The mobile phase consisted of 25 mM potassium phosphate buffer (pH 8.4) and N, N-dimethylformamide (DMF) at changing ratios (Table S1). The analyses were conducted at 4°C/ 30°C in the autosampler/ column oven, respectively. The chromatograms were integrated automatically using the software OpenLab (Agilent Technologies) and inspected manually. This way, the three vitamers of thiamine, TF, TMP, and TDP, were quantified, and the amounts were summed and normalized by wet weight to total thiamine (Ttot) using the unit nmol g−1 wet weight.

Table S1: Proportional changes in mobile phase composition during HPLC run.

Time point (min)	25 mM pot. P. buffer (%)	DMF (%)
0	100	0
0.1	100	0
1	99.5	0.5
5	90	10
7	90	10
9	70	30
11	65	35
11.1	100	0

1.3 *Raw data*

Table S2: Musel shell length and thiamine concentration per soft tissues mass and whole mussel mass.

Geographic location (habitat)	Shell Length (cm)	Thiamine (nmol/g of soft tissues)	Thiamine (nmol/g of whole mussel)
Lysekil , Sweden (wintering grounds)			
	1.933	1.3827	0.2475
	2.133	0.6763	0.1068
	2.600	1.0185	0.2101
	2.700	1.1042	0.2294
	2.900	0.4959	0.1050
	3.100	0.6480	0.2016
	3.200	0.7717	0.1829
	3.300	0.7408	0.1602
	3.500	0.8974	0.2083
	3.600	0.5893	0.1502
	3.700	0.6647	0.1843

	3.800	0.6246	0.1402
	4.000	0.4173	0.0766
	4.000	0.9236	0.1976
	4.100	0.6511	0.1362
	4.100	0.6796	0.0585
	4.200	0.8078	0.1634
	4.500	1.1861	0.1506
	4.500	0.6094	0.1504
	4.800	0.7444	0.1239
	4.900	0.2796	0.0462
	5.000	0.7076	0.1798
	5.100	0.2343	0.0294
	5.200	0.4688	0.1064
Sylt , Germany (wintering grounds)			
	1.035	0.9197	0.1904
	1.850	1.1537	0.2689
	1.900	0.3120	0.0913
	1.967	0.8372	0.2129
	2.000	1.0585	0.2635
	2.100	0.5859	0.1606
	2.800	0.7579	0.1893
	2.900	0.8159	0.1662
	3.000	1.0408	0.2509
	2.800	0.7816	0.2521
	3.100	0.5890	0.1565
	3.800	0.7098	0.1209
	3.800	0.8173	0.1886
	3.900	0.7610	0.1214
	4.000	0.7359	0.1319
	4.100	0.7482	0.1526
Kalmar , Sweden (stopover)			
	1.150	0.8260	0.2427
	1.383	1.0759	0.2947
	1.375	1.0885	0.4046
	1.500	1.0201	0.3441
	1.600	1.0704	0.3525
	1.833	1.0921	0.2570
	1.900	1.0595	0.4149
	2.000	1.1035	0.3054
	2.100	1.0440	0.2650
	2.200	0.7936	0.3259
	2.400	1.0802	0.3016
	2.500	0.9656	0.4070
	2.600	1.0140	0.2655
	2.700	1.3207	0.4987
	2.800	0.6504	0.1701
	3.000	0.8564	0.3147
	3.100	0.6964	0.2315
	3.200	0.6070	0.1506

	3.300	0.6148	0.1519
	3.400	0.8756	0.1584
	1.360	1.2437	0.3288
	1.950	1.5601	0.5972
	1.850	1.1985	0.2889
	2.050	1.3113	0.3736
	2.000	1.0815	0.3299
	2.200	1.1681	0.4622
	2.400	0.9275	0.2861
	2.500	1.4119	0.4230
	2.600	1.5231	0.4048
	2.700	1.5139	0.4011
	2.800	0.8237	0.2694
	3.000	0.9667	0.3149
	3.100	0.7813	0.3473
	3.200	0.7902	0.2311
	3.300	0.8419	0.2466
	3.400	1.2452	0.3876
	3.400	1.2652	0.2501
	3.500	1.0451	0.3425
	3.600	0.7280	0.1726
	3.600	0.8430	0.1733
	3.700	0.6886	0.1327
Stockholm , Sweden (breeding grounds)			
	1.400	0.8737	0.3164
	1.667	0.8242	0.2997
	1.700	1.0155	0.4197
	1.733	1.1083	0.3285
	1.800	0.8676	0.2566
	1.900	0.8567	0.2825
	2.000	0.9085	0.3089
	2.050	0.9125	0.2796
	2.100	0.8490	0.3310
	2.200	0.7285	0.2967
	2.400	0.7601	0.2228
	2.500	0.5998	0.2010
	2.600	0.6040	0.2498
	2.700	0.5633	0.1549
	2.800	0.5760	0.1858
	3.000	0.6047	0.2017
	3.000	0.5373	0.1870
	3.100	0.6495	0.1147
	3.200	0.5862	0.1935
	3.300	0.3738	0.1043
	3.400	0.5918	0.1757
	3.500	0.3595	0.0822
	3.500	0.3349	0.1199
	3.600	0.5164	0.1363
	3.700	0.6178	0.1546

2. Model parameterization - common eider life history

2.1 *Time constraints and costs of migration*

The timing of migration of common eiders in the model, i.e. day of year at which females migrate from wintering grounds to stopover α_1, from stopover to breeding grounds α_2, and from breeding grounds to wintering grounds α_3, evolved to maximize fitness (see the main text). However, to mimic the migration phenology of common eiders in the Baltic Sea and the climate-related conditions, we imposed constraints on the timing of migration. (i) In the main results we assumed that females are obligated to stay on the wintering grounds at least until early February (Waltho and Coulson 2015, Laursen et al. 2019, and Fig 4A-C in S1 Appendix) implemented in the model by an unavoidable death of females leaving the wintering grounds before day of year t=30. After t=30, a model female could decide on which day of the year α_1 to migrate to the stopover and on which day of the year α_2 to migrate from the stopover to the breeding grounds. (ii) On the breeding grounds, females could lay eggs and reproduce (see the main text and section 1.2 in Appendix S1), and by the end of August (t=239) they could decide on the day of the year α_3 on which to migrate to the wintering grounds. Migration from breeding to wintering grounds was modelled for simplicity as migration without stopover. (iii) We also assumed that females that did not migrate to the wintering grounds before the end of December (t=360) would die. Common eider migration involves high energetic costs equivalent to 16-20 times the RMR cost (Guillemette and Ouellet 2005, Pelletier et al. 2008), with the energy expenditure of a few hours of flight corresponding approximately to the energetic value of 200 g of lipid reserves (Parker and Holm 1990). Consequently, we assumed in the model that migration from the wintering grounds to the stopover or from the stopover to the breeding grounds caused a loss of 400 g of reserves, and in the case of migration from the breeding grounds to the wintering grounds, the loss was 800 g (see Fig 9 in S1 Appendix for sensitivity analysis of the model to migration costs assumed). To account for the fact that the energetic cost of flight increases rapidly with body mass in common eiders (Guillemette and Ouellet 2005, Pelletier et al. 2008), we assumed that the reserve balance at arrival depended on body mass at departure. Females in the model that started migration with maximum reserves arrived in a lean body condition, whereas those that start migration with low but sufficient reserves arrived with up to 100 g of reserves i.e. the baseline reserve level in wintering and migrating females (Beuth et al. 2016). The reserves level at arrival S_a was given by

(S.1) $S_a = (S_x - S)\frac{100}{S_x - c_m}$

where c_m matches energetic costs of migration, and S_x is the maximal reserve level (see the main text).

2.2 *Breeding grounds*

On the breeding grounds, females optimized the timing of egg laying and clutch size. Females with sufficient reserves laid eggs at a rate of one per day (Watson et al. 1993). For simplicity, we assumed that a female that initiated egg laying could either lay another egg or start incubation, although lack of reserves to cover the energetic cost of incubation resulted in clutch loss and zero fitness payoff. Females could produce up to n_x=5 eggs, with the maximum clutch size reflecting the variation in clutch size of common eiders

(Erikstad et al. 1993, Waltho and Coulson 2015). For simplicity, we assumed that females produced eggs of the same size, E=110 g each (Parker and Holm 1990, Waltho and Coulson 2015), allocating the same mass of body reserves per egg produced plus 16.5 g lost during laying (Parker and Holm 1990). Incubation of the clutch lasted 26 days (Parker and Holm 1990, Öst and Steele 2010), during which females lost appriximately 40% of their body weight, set to c_m=800 g depletion of reserves S in the model, which is consistent with the empirical data on average mass loss during incubation for this species (Bolduc and Guillemette 2003, Waltho and Coulson 2015). To keep the nesting phenology consistent with the Baltic population of common eiders, we assumed that females could not initiate egg laying before t=120 i.e. early May (Waltho and Coulson 2015, Fig 4D-F in the main text, and Fig 4A-C in S1 Appendix). Offspring value $\gamma(t)$, was maximal in early May (t=120) and decreased exponentially until the end of July (t=210) (see the main text).

2.3 *Mortality risk*

The annual survival of adult females eiders is approximately 0.87 (Nicol-Harper et al. 2021), which was recalculated in our model to a daily survival probability m_f=0.87^(1/360). For simplicity, we also assumed that survival m_f applied to all female behaviors except incubation (Mohring et al. 2022), during which the probability of survival was lower and given by $m_i = m_f^v$, with v=5 in the main results (see Figs 6, 8 in S1 Appendix for the model sensitivity analysis of the model to survival rates m_f and m_i). As extensive storage in common eiders reduces female mobility and their vulnerability to predation (Guillemette and Ouellet 2005, Pelletier et al. 2008), we assumed that the survival component m_r related to carried reserves decreased exponentially according to

(S.2) $m_r = e^{-Sc}$ with the value of c set to 10^{-5} so that the daily survival of females carrying maximum body reserves S_x was lowered by 0.15%. Females in the model starve with a 20% reduction in survival probability in three cases: if they remain on the breeding grounds after t=239, i.e. the end of August (see above); when their reserve level S on the breeding grounds falls to 0; or when reserves on the wintering and stopover grounds fall below S_r=100 g - reported in the empirical literature as the minimum reserve level needed to survive in the pre-breeding phase (Beuth et al. 2016, see also Fig 6, 7 in S1 Appendix for sensitivity analysis of the model to starvation-induced survival rates and survival thresholds).

3. Model parameterization - daily food intake and thiamine input

3.1 *Stochastic food gain*

The average daily food gain of foraging females fluctuated stochastically in our model and was parameterized using data on the average reserves gain of foraging common eiders. A female that did not forage but rest maintain resting metabolism, thermoregulation, etc. and utilized 37.5 g of reserves (Parker and Holm 1990, Gabrielsen et al. 1991, Bolduc and Guillemette 2003). We assumed that the average daily reserve gain w of 15 g (Korschgen 1977, Rigou and Guillemette 2010) was equivalent to the consumption of 1.9 kg of blue mussels (Guillemette 1998). The daily reserve gain w_i fluctuated temporarily and represented the stochastic nature of the foraging process by varying between x_1w and x_2w. In our work we set x_1=0.6 and x_2=1.4, although a greater or lesser variation in the daily food gain defined by the parameters x_1 and x_2 did not affect the conclusions (see Fig 10 in S1 Appendix). Each day of foraging resulted in a specific outcome

in terms of food gain with i=1, 2, … k foraging outcomes in our model, each with probability of occurring p_i, a daily net food gain w_i, and k set to 50 in the results presented below. The probabilities p_i were derived from a β probability distribution as it is defined on the interval [0, 1], which, after rescaling, allowed us to keep the food gain within a predefined interval of x_1w and x_2w. We also set the shape of the probability distribution to resemble a normal distribution by setting the parameters of the β probability distribution $\beta_1=\beta_2=4$ (see Fig 1C in the main text). See Fig 10 in S1 Appendix for the sensitivity analysis of the model results to the assumed shape and variance of the distribution of daily food intake.

3.2 *Thiamine intake*

Thiamine input was determined by the daily food intake, thiamine bioavailability (i.e. the fraction of thiamine consumed that is assimilated), thiamine levels in blue mussels at given geographic location, and size of the consumed prey (see the main text). While carnivorous birds feeding on invertebrates metabolize approximately 75% of the energy consumed (Karasov 1990), data on thiamine bioavailability in trophic interactions of marine food webs are scarce. Hylander et al. (2024) estimated that thiamine availability may vary depending on the level of trophic interactions considered, with lower bioavailability in the range of 20-30% in lower parts of the food web, and higher bioavailability of about 50-75% for piscivorous fish feeding on planktivorous fish. Therefore, we assumed in the main results that thiamine bioavailability in ducks feeding on mussels is 50% (see also Fig 4A-C in the main text). The concentration of thiamine per mass of mussels consumed was calculated by assuming that the soft tissues contain thiamine while the shells and free water from the interior are free of thiamine, and by using the weights of soft tissues and whole mussels.

3.3 *Key model parameters*

Table 2. Key parameters of the model and their baseline values.

Parameter	Description	Unit	Value
M_L	Lean body mass	gram	1100
S_x	Maximal reserves	gram	1500
S_r	Reserves threshold for starvation	gram	100
n_x	Maximal clutch size	–	5
E	Egg mass	gram	110
I	Incubation duration	day	26
w	Net weight gain	$\text{gram}\cdot\text{day}^{-1}$	15
h	Maintenance costs	$\text{gram}\cdot\text{day}^{-1}$	37.5
c_m	Incubation costs	gram	800

4. Sensitivity analysis

4.1 *Alternative calculations of thiamine levels in female eiders*

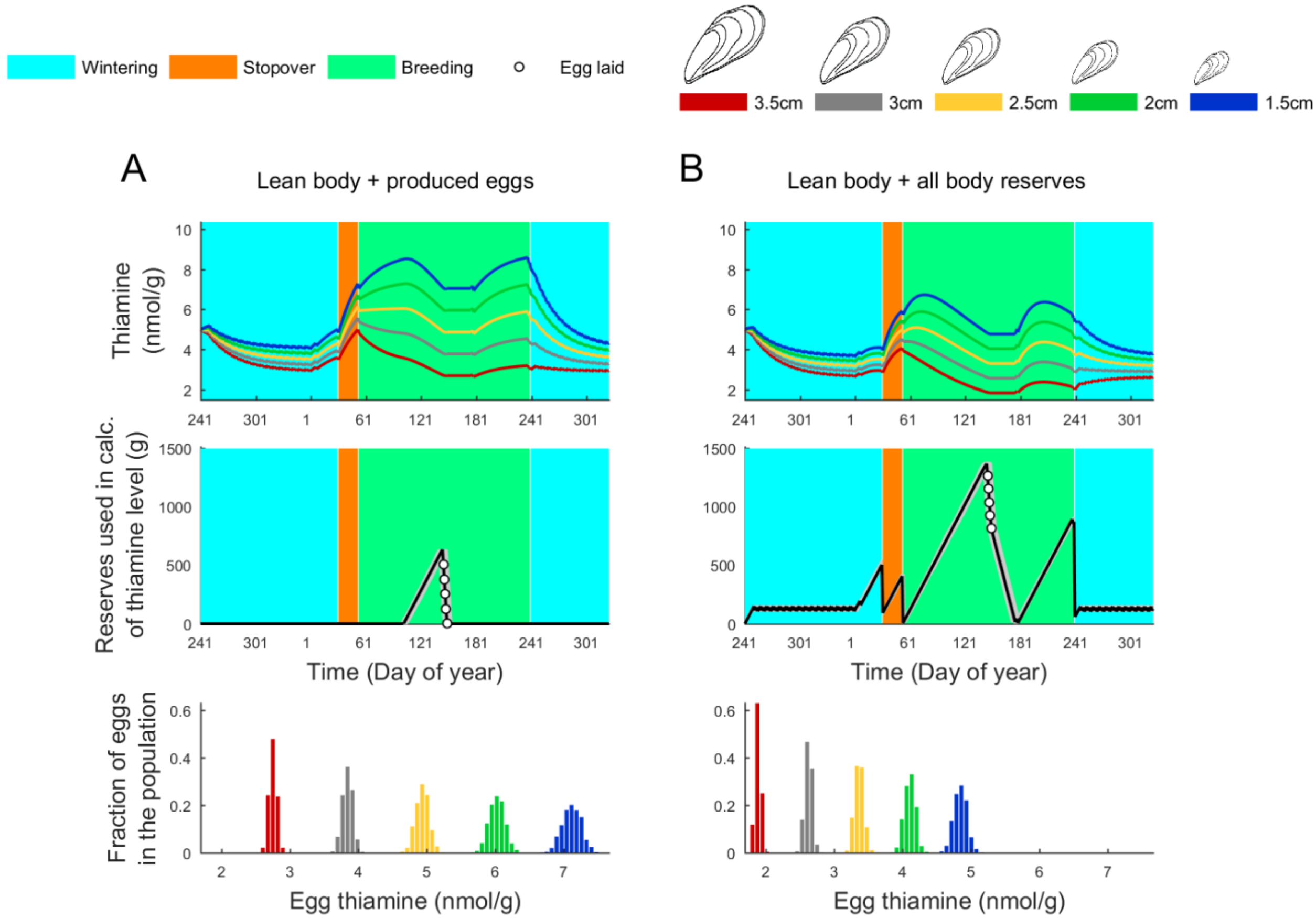

Fig 3. Thiamine levels and reserve trajectories in scenarios with alternative ways of calculating the reserves involved in thiamine retention. (A-B) Panels show results for a baseline scenario with thiamine concentration calculated in two alternative ways: (A) thiamine content divided by lean body mass plus the part of reserves devoted to egg production, i.e. according to the same procedure as in the main results. (B) An alternative way of calculation with thiamine content divided by lean body mass plus total body reserves. (A-B) The histograms show the intrapopulation variance in egg thiamine levels, while the colors represent the consumed prey size (see legend on the right). The solid black line represents a model female exposed to average food intake with colors of the background indicating time spent at wintering, stopover and breeding grounds (see legend on the left). Shaded area around the thick black line represents variability between individuals due to stochastic food intake. Scenarios were modelled with the offspring value γ determined by m_d=0.01 (see eq. 1 in the main text), the rate of thiamine loss set by c_t=3 and b=1 (see eq. 2 in the main text), and other model parameters at their baseline values. Plots of thiamine and reserve trajectories are representative examples of one year in the life of modelled females.

4.2 *Phenology of overwintering and breeding*

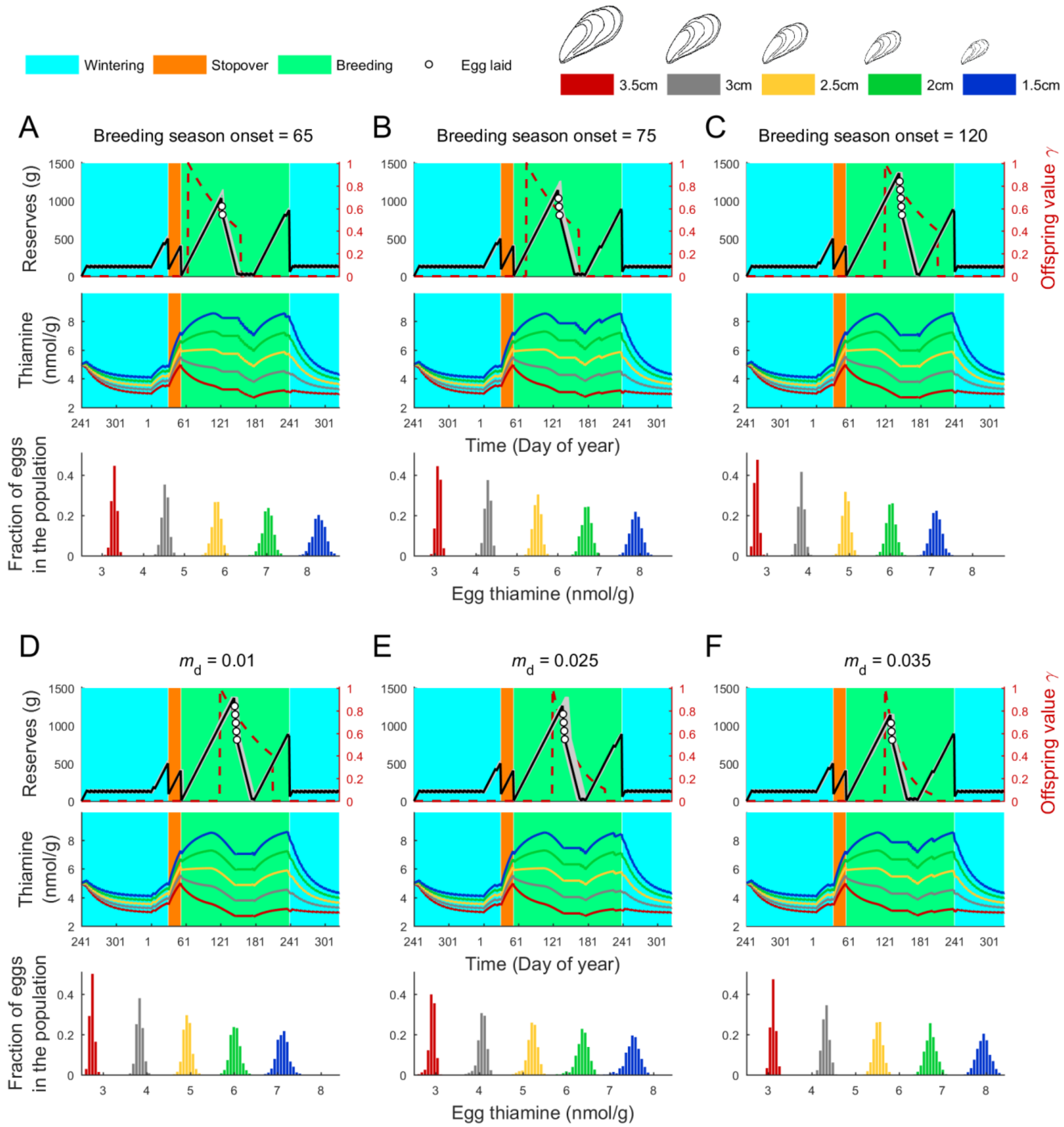

Fig 4. Thiamine levels and reserve trajectories under different seasonal constraints for nesting and wintering and offspring prospects as a function of birth timing. (A-C) Results for scenarios differing in the first possible day of nesting, with t=120 assumed in the main results, and (D-F) differing in the offspring value, with γ determined by m_d=0.01 in the main results. (A-F) Histograms show intrapopulation variance in egg thiamine levels, with colors representing consumed prey size (see legend on right). The solid black line represents a model female exposed to the average food intake, while the colors of the background indicate the time spent at wintering, stopover and breeding grounds (see legend on the left). The shaded area around the thick black line represents the variability between individuals due to stochastic food intake. The red dashed line shows the offspring value γ (see eq. 1 in the main text). The rate of thiamine loss was determined by c_t=3 and b=1 (see eq. 2 in the main text), and other model parameters at their baseline values. Plots of thiamine and reserve trajectories are representative examples of one year in the life of modelled females.

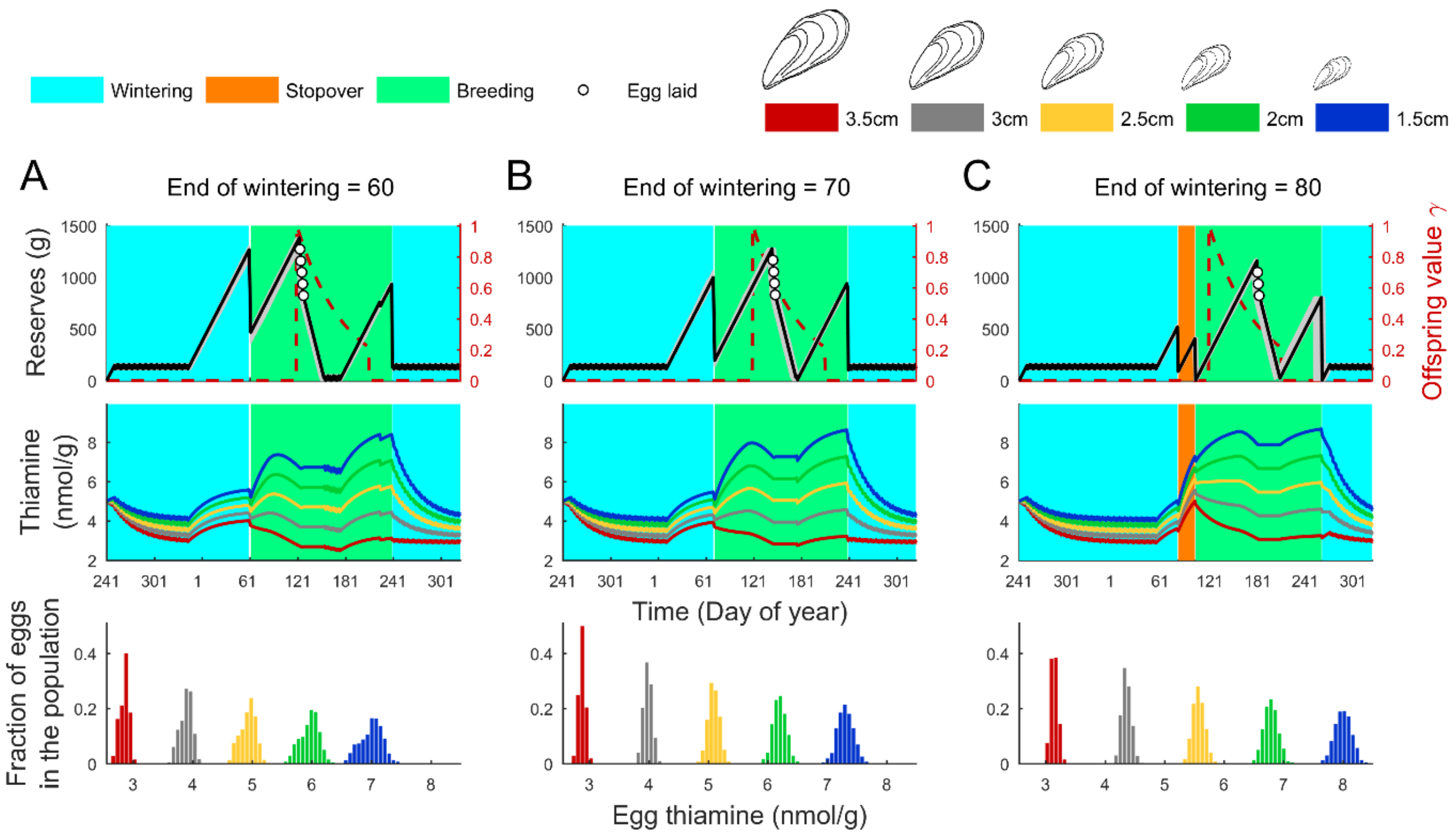

Fig 5. Thiamine levels and reserve trajectories under different seasonal constraints for spring migration and costs of overwintering. (A-C) Results for scenarios differing in the end of obligatory overwintering, with females arriving with: 450 g of reserves at short overwintering period (A), 200 g of reserves in case of intermediate length of overwintering period (B), and at lean body condition for long overwintering period (C). Skipping stopover in A and B by migrating females is a result of optimization. Histograms show intrapopulation variance in egg thiamine levels, with colors representing consumed prey size (see legend on right). The solid black line represents a model female exposed to the average food intake, while the colors of the background indicate the time spent at wintering, stopover and breeding grounds (see legend on the left). The shaded area around the thick black line represents the variability between individuals due to stochastic food intake. The red dashed line shows the offspring value γ (see eq. 1 in the main text). Scenarios were modelled with the offspring value γ determined by m_d=0.017, the rate of thiamine loss set by c_t=3 and b=1 (see eq. 2 in the main text), and other model parameters at their baseline values. Plots of thiamine and reserve trajectories are representative examples of one year in the life of modelled females.

4.3 *Background and starvation-driven mortality risk*

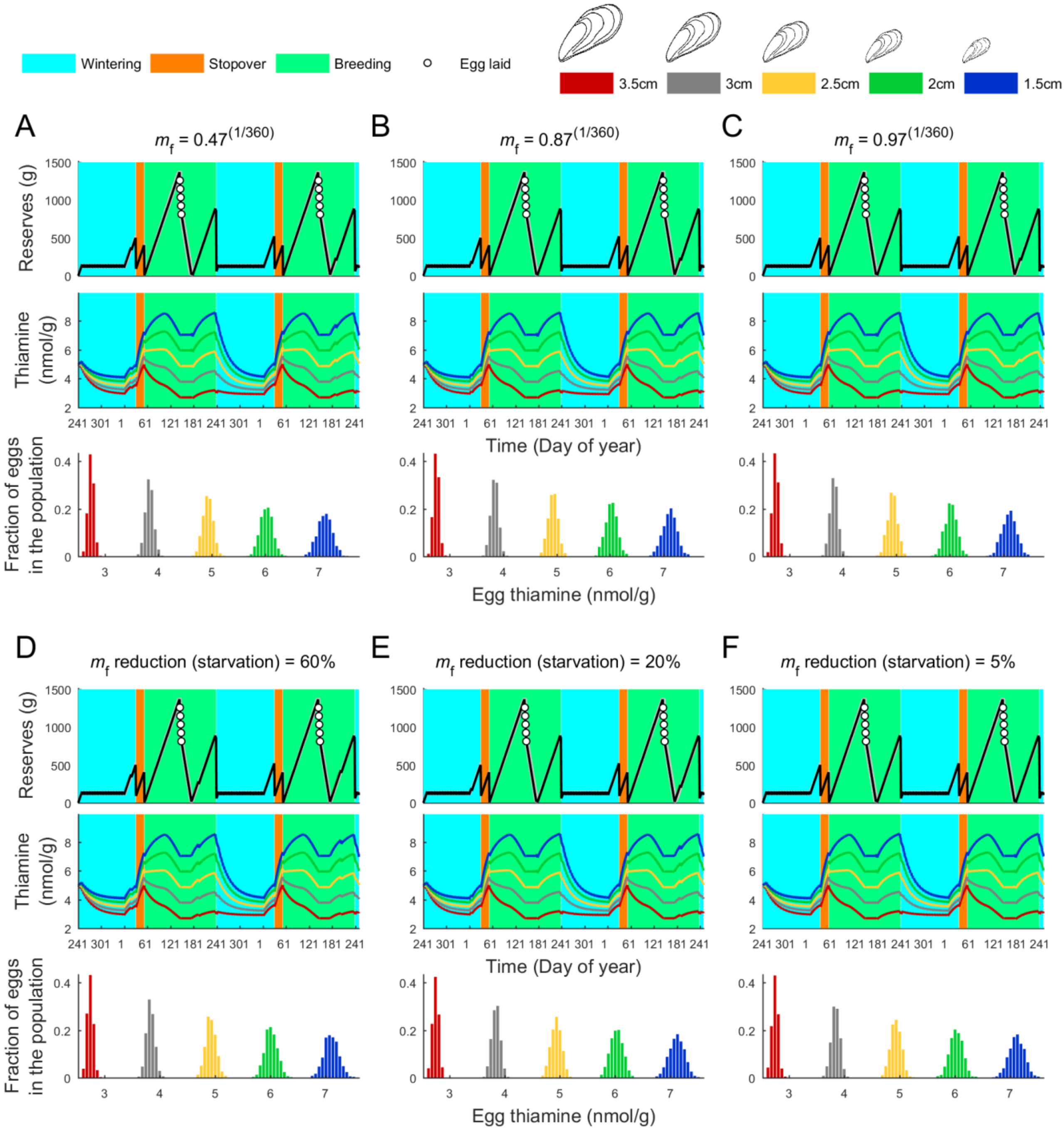

Fig 6. Thiamine levels and reserve trajectories under different survival rates and starvation-driven mortality risk. (A-C) Results for scenarios differing in the daily survival probability m_f, with m_f =0.87$^{(1/360)}$ assumed in the main results. (D-F) Results for scenarios with different starvation-driven mortality risk, with m_f reduced due to starvation by 20% in the main results. (A-F) Histograms show intrapopulation variance in egg thiamine levels, with colors representing consumed prey size (see legend on right). The solid black line represents a model female exposed to the average food intake, while the colors of the background indicate the time spent at wintering, stopover and breeding grounds (see legend on the left). The shaded area around the thick black line represents the variability between individuals due to stochastic food intake. Scenarios were modelled with the offspring value γ determined by m_d=0.01 (see eq. 1 in the main text), the rate of

thiamine loss set by c_t=3 and b=1 (see eq. 2 in the main text), and other model parameters at their baseline values. Plots of thiamine and reserve trajectories show representative examples of two nesting seasons in the life of modelled females.

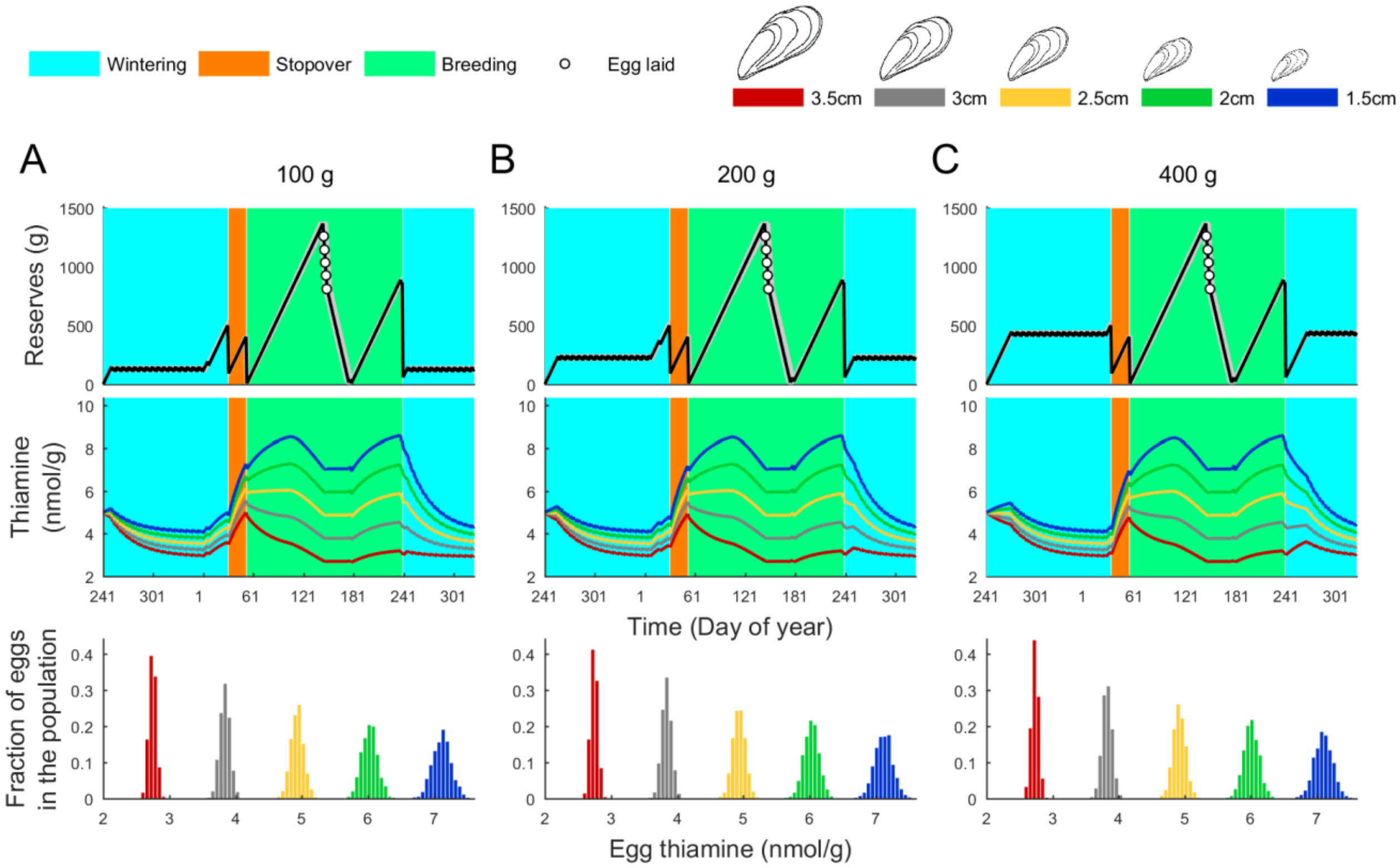

Fig 7. Thiamine levels and reserve trajectories under different starvation thresholds. (A-C) Results for scenarios with different starvation threshold S_r (given at the top of the panels). (A-C) Histograms show intrapopulation variance in egg thiamine levels, with colors representing consumed prey size (see legend on right). The solid black line represents a model female exposed to the average food intake, while the colors of the background indicate the time spent at wintering, stopover and breeding grounds (see legend on the left). The shaded area around the thick black line represents the variability between individuals due to stochastic food intake. Scenarios were modelled with the offspring value γ determined by m_d=0.01 (see eq. 1 in the main text), the rate of thiamine loss set by c_t=3 and b=1 (see eq. 2 in the main text), and other model parameters at their baseline values. Plots of thiamine and reserve trajectories show representative examples of two nesting seasons in the life of modelled females.

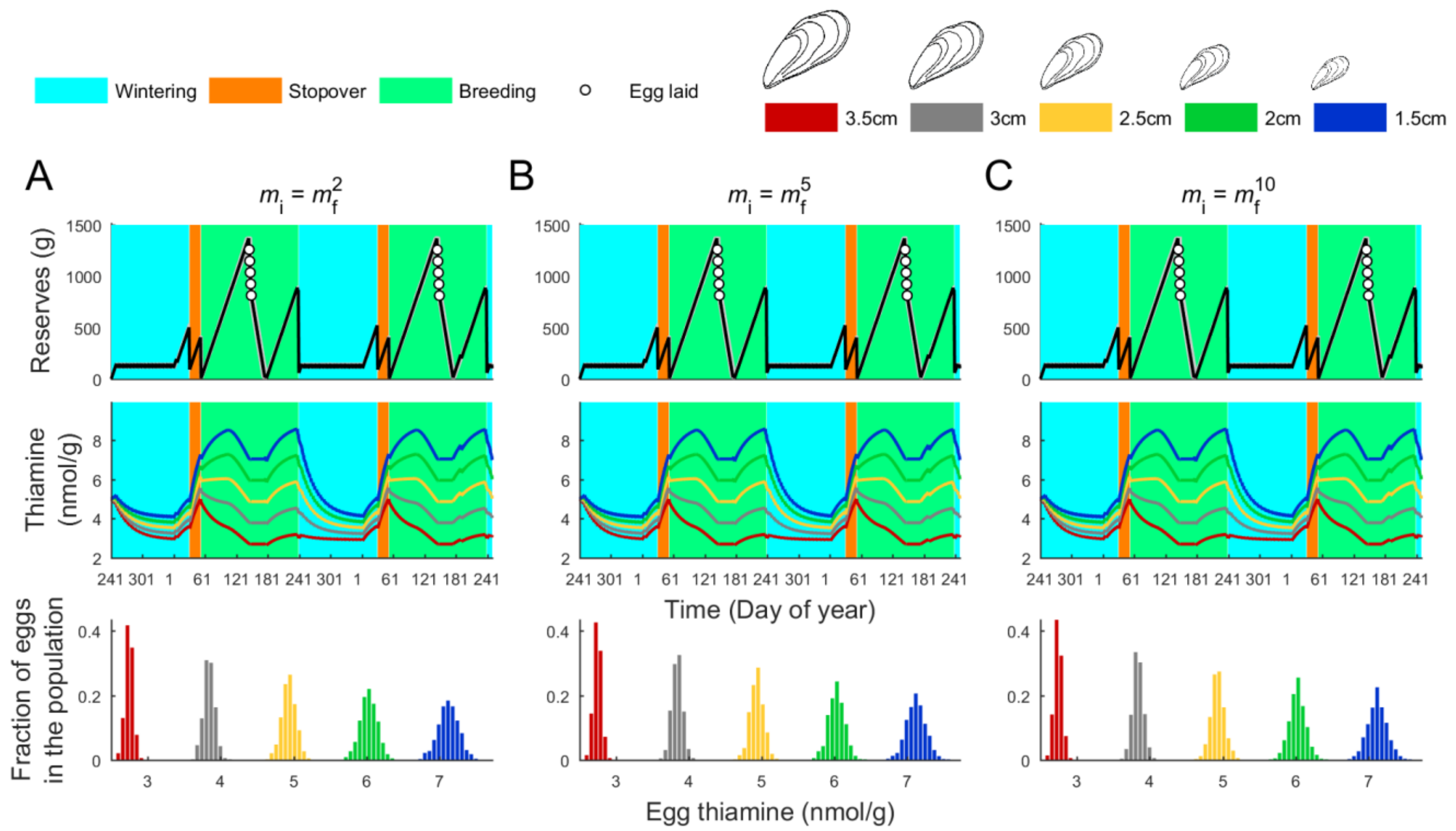

Fig 8. Thiamine levels and reserve trajectories under different survival rates during incubation. (A-C) Results for scenarios differing in the daily survival probability during incubation m_i, with $m_i = m_f^v$ and v set to 2, 5 or 10 (see labels above panels). The middle panel with v set to 5 depicts baseline scenario presented in the main text. (A-C) Histograms show intrapopulation variance in egg thiamine levels, with colors representing consumed prey size (see legend on right). The solid black line represents a model female exposed to the average food intake, while the colors of the background indicate the time spent at wintering, stopover and breeding grounds (see legend on the left). The shaded area around the thick black line represents the variability between individuals due to stochastic food intake. Scenarios were modelled with the offspring value γ determined by m_d=0.01 (see eq. 1 in the main text), the rate of thiamine loss set by c_t=3 and b=1 (see eq. 2 in the main text), and other model parameters at their baseline values. Plots of thiamine and reserve trajectories show representative examples of two nesting seasons in the life of modelled females.

4.4 *Migration costs*

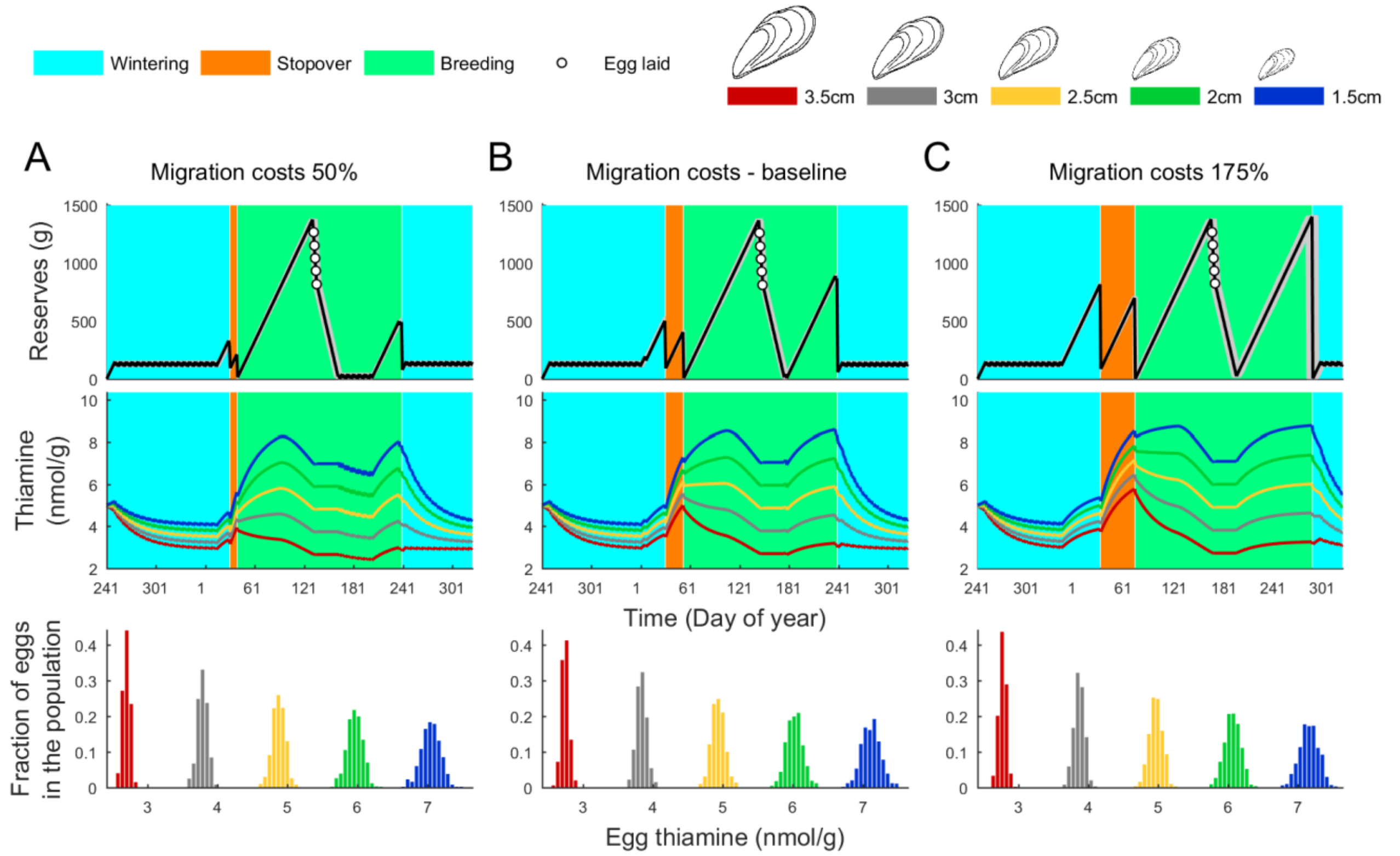

Fig 9. Thiamine levels and reserve trajectories in scenarios with different migration costs. (A-C) Panels A and C show results for scenarios where the energetic cost of migration, i.e. the amount of reserves required to complete migration, differed from the baseline scenario shown in B (see labels at the top of the graphs). In the main results, the baseline migration costs were set at 400g of reserve depletion for spring migrations and 800g depletion for autumn migration. (A-F) Histograms show intrapopulation variance in egg thiamine levels, with colors representing consumed prey size (see legend on right). The solid black line represents a model female exposed to the average food intake, while the colors of the background indicate the time spent at wintering, stopover and breeding grounds (see legend on the left). The shaded area around the thick black line represents the variability between individuals due to stochastic food intake. Scenarios were modelled with the offspring value γ determined by m_d=0.01 (see eq. 1 in the main text), the rate of thiamine loss set by c_t=3 and b=1 (see eq. 2 in the main text), and other model parameters at their baseline values. Plots of thiamine and reserve trajectories are representative examples of one year in the life of modelled females.

4.5 *Stochastically fluctuating food intake*

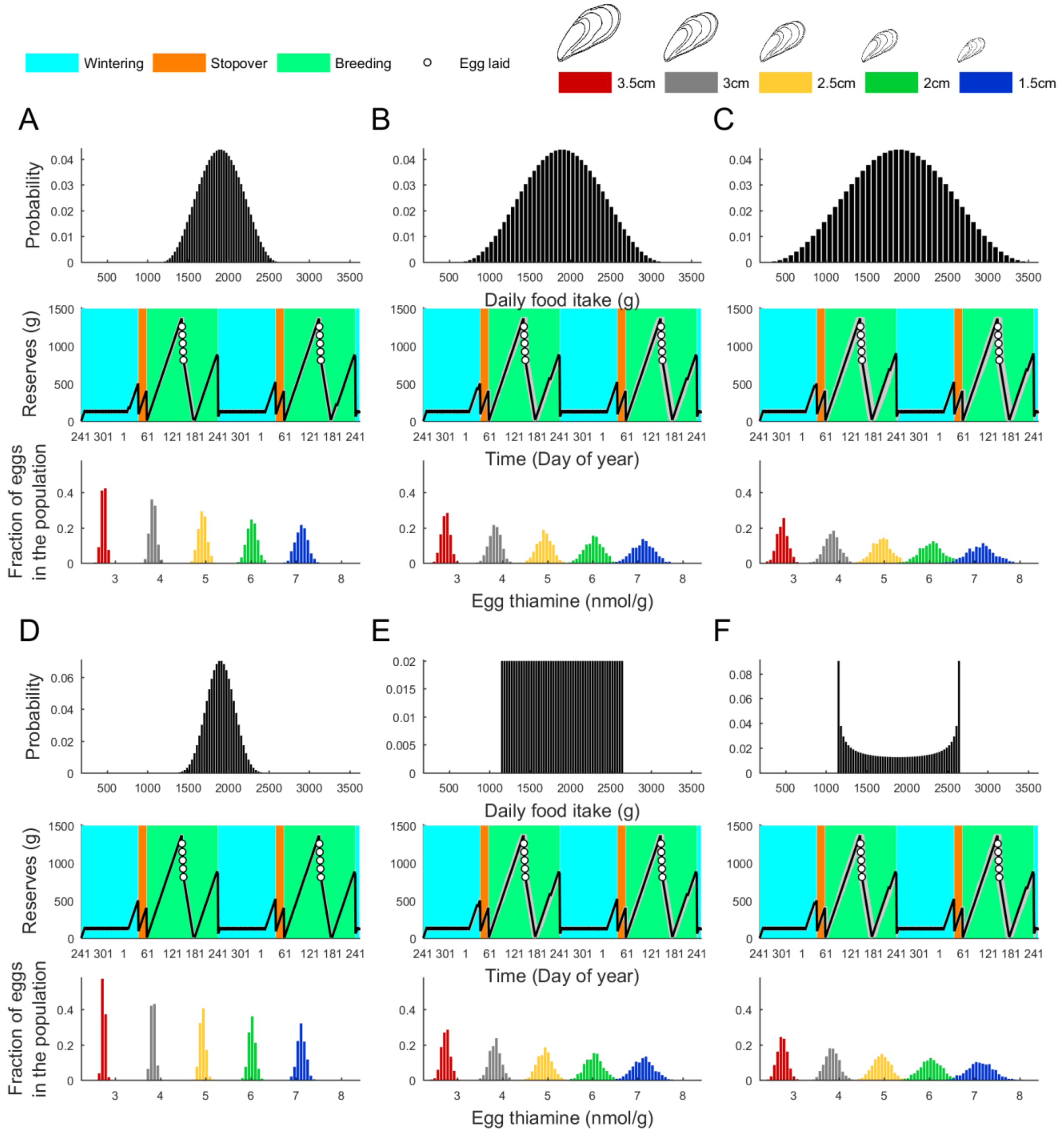

Fig 10. Thiamine levels and reserve trajectories in scenarios with different stochastic variation of daily food intake. Stochastic daily food intake in the baseline scenario (panel A) was set by a β distribution (black histogram in A) with variance and shape set by x_1=0.6, x_2=1.4, and β_1=β_2=4 (see section 3.1 Stochastic food intake above). Other scenarios presented were modelled with stochastic food intake determined by β distributions (black histograms) with the following parameters: (B) x_1=0.3, x_2=1.7, β_1=β_2=4, (C) x_1=0.1, x_2=1.9, β_1=β_2=4, (D) x_1=0.6, x_2=1.4, β_1=β_2=10, (E) x_1=0.6, x_2=1.4, β_1=β_2=1, (F) x_1=0.6, x_2=1.4, β_1=β_2=0.5. (A-F) Histograms with colors show intrapopulation variance in egg thiamine levels, with consumed prey size in legend on the right. The solid black line represents a model female exposed to the average food intake,

while the colors of the background indicate the time spent at wintering, stopover and breeding grounds (see legend on the left). The shaded area around the thick black line represents the variability between individuals due to stochastic food intake. Scenarios were modelled with the offspring value γ determined by m_d=0.01 (see eq. 1 in the main text), the rate of thiamine loss set by c_t=3 and b=1 (see eq. 2 in the main text), and other model parameters at their baseline values. Plots of thiamine and reserve trajectories show representative examples of two nesting seasons in the life of modelled females.

4.6 *Food to reserves conversion*

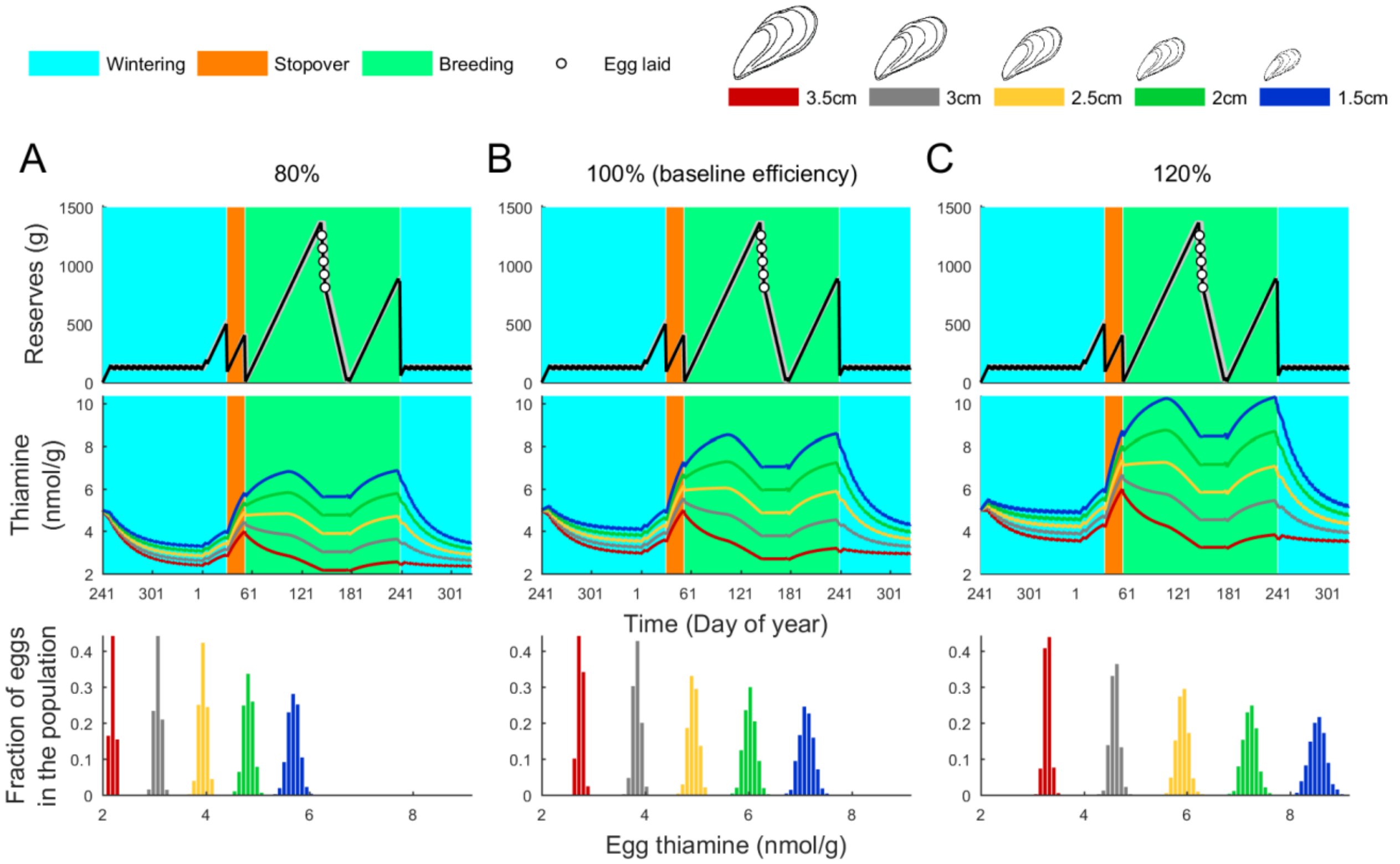

Fig 11. Thiamine levels and reserve trajectories in scenarios with different conversion rates of consumed food to reserve gain. (B) In the baseline scenario we assumed the average reserve gain w=15g corresponds to daily consumption of 1.9 kg of mussels, while A and C show scenarios with conversion efficiency set to 80 and 120% (see labels above panels). (A-C) Histograms show intrapopulation variance in egg thiamine levels, with colors representing consumed prey size (see legend on right). The solid black line represents a model female exposed to the average food intake, while the colors of the background indicate the time spent at wintering, stopover and breeding grounds (see legend on the left). The shaded area around the thick black line represents the variability between individuals due to stochastic food intake. Scenarios were modelled with the offspring value γ determined by m_d=0.01 (see eq. 1 in the main text), the rate of thiamine loss set by c_t=3 and b=1 (see eq. 2 in the main text), and other model parameters at their baseline values. Plots of thiamine and reserve trajectories show representative examples of two nesting seasons in the life of modelled females.

4.7 *Maximal clutch size*

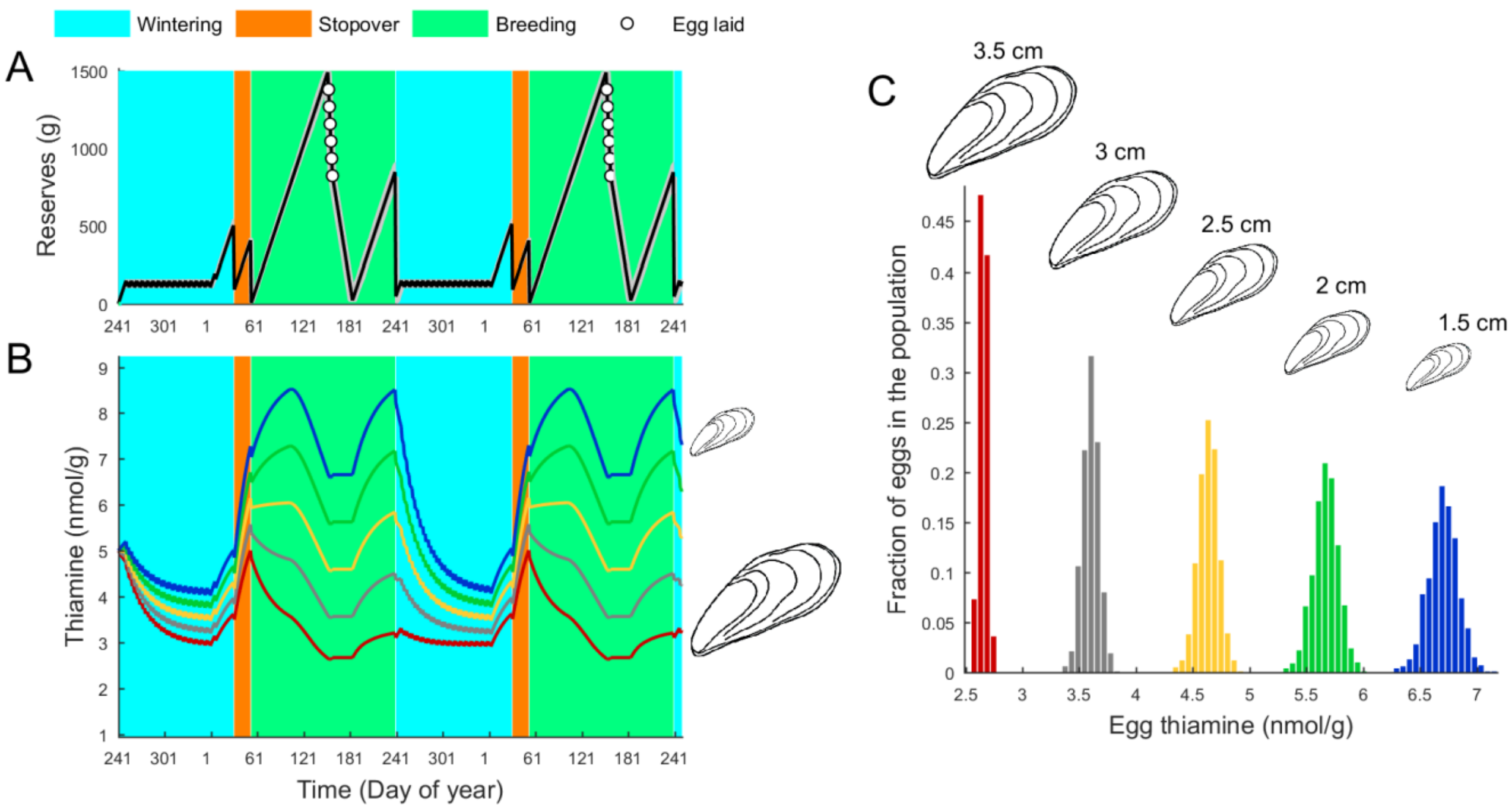

Fig 12. Dynamics of body reserves and thiamine tissue concentrations with maximal clutch size set to 6 eggs. (A) The seasonal dynamics of female body reserves, timing of migration and nesting that maximize fitness. The solid black line represents a model female exposed to average food intake, while the shaded area around the thick black line represents variability between individuals due to stochastic food intake. (B) Thiamine concentrations in the body of females with different consumed prey sizes, ranging from 1.5 to 3.5 cm of the blue mussels shell length. The colors of the thick lines in B and C match. (C) Thiamine levels in the eggs produced by females in the population at the start of incubation. The distributions show the intrapopulation variance in egg thiamine levels, while the colors represent the consumed prey size (see legend). (A-C) The offspring value γ was determined by m_d=0.01, the rate of thiamine loss was set by the shape parameters c_t=3 and *b*=2, with other model parameters at their baseline values. (A-B) Panels show trajectories over the first two years of a female's life, representative of the 20 years of life assumed in the optimization.

5. References

Beuth, J. M., P. W. C. Paton, J. E. Osenkowski, and S. R. McWilliams. 2016. Body composition dynamics of common eider during winter: An application of the deuterium dilution method. Wildlife Society Bulletin **40**:464-469.

Bolduc, F., and M. Guillemette. 2003. Incubation constancy and mass loss in the Common Eider Somateria mollissima. Ibis **145**:329-332.

Brown, S. B., D. C. Honeyfield, and L. Vandenbyllaardt. 1998. Thiamine analysis in fish tissues. Pages 73-81 Early Life Stage Mortality Syndrome in Fishes of the Great Lakes and Baltic Sea.

Erikstad, K. E., J. O. Bustnes, and T. Moum. 1993. Clutch-size determination in precocial birds - a study of the common eider. Auk **110**:623-628.

Futia, M. H., S. Hallenbeck, A. D. Noyes, D. C. Honeyfield, G. E. Eckerlin, and J. Rinchard. 2017. Thiamine deficiency and the effectiveness of thiamine treatments through broodstock injections and egg immersion on Lake Ontario steelhead trout. Journal of Great Lakes Research **43**:352-358.

Futia, M. H., and J. Rinchard. 2019. Evaluation of adult and offspring thiamine deficiency in salmonine species from Lake Ontario. Journal of Great Lakes Research **45**:811-820.

Gabrielsen, G. W., F. Mehlum, H. E. Karlsen, O. Andresen, and H. Parker. 1991. Energy cost during incubation and thermoregulation in the female common eider Somateria mollissima. Norsk Polarinstitute Skrifter **195**:51–62.

Guillemette, M. 1998. The effect of time and digestion constraints in Common Eiders while feeding and diving over Blue Mussel beds. Functional Ecology **12**:123-131.

Guillemette, M., and J. F. Ouellet. 2005. Temporary flightlessness in pre-laying Common Eiders Somateria mollissima: are females constrained by excessive wing-loading or by minimal flight muscle ratio? Ibis **147**:293-300.

Hamilton, D. J., T. D. Nudds, and J. Neate. 1999. Size-selective predation of blue mussels (Mytilus edulis) by common eiders (Somateria mollissima) under controlled field conditions. Auk **116**:403-416.

Hylander, S., H. Farnelid, E. Fridolfsson, M. M. Hauber, V. Todisco, M. J. Ejsmond, and E. Lindehoff. 2024. Thiamin (vitamin B1, thiamine) transfer in the aquatic food web from lower to higher trophic levels. Plos One **19**.

Karasov, W. H. 1990. Digestion in birds: Chemical and physiological determinants and ecological implications. Studies in Avian Biology **13**:391-415.

Korschgen, C. E. 1977. Breeding stress of female Eiders in Maine. Journal of Wildlife Management **41**:360-373.

Laursen, K., A. P. Moller, L. Haugaard, M. Öst, and J. Vainio. 2019. Allocation of body reserves during winter in eider Somateria mollissima as preparation for spring migration and reproduction. Journal of Sea Research **144**:49-56.

Mohring, B., F. Angelier, K. Jaatinen, B. Steele, E. Lönnberg, and M. Öst. 2022. Drivers of within- and among-individual variation in risk-taking behaviour during reproduction in a long-lived bird. Proceedings of the Royal Society B: Biological Sciences **289**.

Nicol-Harper, A., K. A. Wood, A. W. Diamond, H. L. Major, A. Petersen, G. Tertitski, C. P. Doncaster, T. H. G. Ezard, and G. M. Hilton. 2021. Vital rate estimates for the common eider Somateria mollissima, a data-rich exemplar of the seaduck tribe. Ecological Solutions and Evidence **2**.

Öst, M., and B. B. Steele. 2010. Age-specific nest-site preference and success in eiders. Oecologia **162**:59-69.

Parker, H., and H. Holm. 1990. Patterns of Nutrient and Energy Expenditure in Female Common Eiders Nesting in the High ArcticEiders Nesting in the High Arctic. Auk **107**:660-668.

Pelletier, D., M. Guillemette, J. M. Grandbois, and P. J. Butler. 2008. To fly or not to fly: high flight costs in a large sea duck do not imply an expensive lifestyle. Proceedings of the Royal Society B: Biological Sciences **275**:2117-2124.

Rigou, Y., and M. Guillemette. 2010. Foraging Effort and Pre-laying Strategy in Breeding Common Eiders. Waterbirds **33**:314-322.

Vuorinen, P. J., R. Parmanne, T. Vartiainen, M. Keinänen, H. Kiviranta, O. Kotovuori, and F. Halling. 2002. PCDD, PCDF, PCB and thiamine in Baltic herring (Clupea harengus L.) and sprat (Sprattus sprattus L.) as a background to the M74 syndrome of Baltic salmon (Salmo salar L.). ICES Journal of Marine Science **59**:480-496.

Waltho, C., and J. Coulson. 2015. The Common Eider. Bloomsbury Publishing.

Watson, M. D., G. J. Robertson, and F. Cooke. 1993. Egg-laying time and laying interval in the common eider. Condor **95**:869-878.
