# Supplementary material for: The risk of thiamine deficiency in common eider ducklings increases when females consume large prey
Source: PLoS One. 2026 Sep 16;21(9):e0356777. doi: 10.1371/journal.pone.0356777 (PMC13580947; doi:10.1371/journal.pone.0356777)
Supplement: Supplementary file 2 [file pone.0356777.s002.pdf]

```
% Selection for large prey increases risk of mass juvenile mortality due to
thiamine deficiency

% Supplementary Material Appendix S2
% Matlab code of the programme

clear; clc; close all;
CnsMusL=[1.5 2 2.5 3 3.5]; % MUSSEL SHELL LENGTH
ThrBr=3; b=2; % THIAMINE EXCRETION RATE;
% Active excretion: Th<ThrBr -> Brk=a.*Th.^b; Passive filtration: for Th>=ThrBr-
Brk=mxBR.*(Th./Cx)
ThBioav=0.5; % THIAMINE BIOAVAILABILITY
m_d=0.01; % OFFSPRING VALUE - shape parameter
% --- THIAMINE
Cx=15; %Max. concentration nmol/g
mxBR=0.5; % Max.excretion rate nmol/g/d
a=(ThrBr.*mxBR)./(Cx.*ThrBr.^b);
AvThS_L=5; % Forward thiamine simulations start level
% --- LIFE HISTROY AND BIOLOGY
AvFoodGain=1900; %g
ResGain_Av = 15; % g reserve gain
Res2Food=AvFoodGain/ResGain_Av;
CostOfIncub=800; % Costs of incubation (26 days)
CostOfMig_BtoW=800; % Costs of migration (Breeding to Wintering)
CostOfMig_Spring=400; % Costs of migration (Wintering to Stopover, Stopover to
Breeding)
Clutchsize_max = 5; Eggweight=110+16.5;
IncubT=26; T=360;
Reserves_max = 1500; LeanBody=1100; %g
N=1000; % Population size (forward simulations)
NoY=10; % backkward optimization timscale (set >20 for fine-precision)
imaxReserv = 200; %grid resolution
afbt=4; % --- STOCHASTIC FOOD INTAKE shape
wm=0.6; wx=1.4;  % --- STOCHASTIC FOOD INTAKE range
BinEde = (0:0.02:1-0.02)+(0.04/2);
PrbDsGn_Frge=diff(betacdf([0 BinEde],afbt,afbt));
FodGaIn=abs(wm-wx)/length(PrbDsGn_Frge);
Vect_wghtGain=wm:FodGaIn:wx;
Vect_wghtGain=Vect_wghtGain(1:end-1)+(FodGaIn/2);
Vect_wghtGain = Vect_wghtGain .* ResGain_Av;
lStG_Frg=length(Vect_wghtGain);
PrbDsGn_Rest=1; VlDst_Rest=-37.5; % Daily reserves utilization for resting birds
lStG_Rst=length(VlDst_Rest);
% ---SEASONALITY
t_NestStrt=120; %(DoY) Nesting season starts (offspring value maximal)
t_NestEnd=t_NestStrt+3*30; %(DoY) Nesting season ends
t_DepBre=240; %(DoY) Day of departure from breeding grounds
t_FPDepW=30; % first possible day of departure from wintering grounds
%---MORTALITY
p_rest=0.87^(1/360); % daily survival - resting
p_forag=p_rest; % daily survival - foraging
p_lay=p_rest; % egg laying - probability of surviving of one day
p_incub=p_rest^5; % incubation - probability of surviving
```

```
p_mig=p_rest; % migration - probability of surviving
p_Starv=0.8; % Starvation mortality - additional component
ResWinStr=100; % critical mass of reserves during wintering
c_d=1e-6; % Mortatlity cost of reserves: y=exp(-reserves.*c_d)
%---Excessive reserves affected body condition at arrival (see main text):
cstMigr=min([CostOfMig_Spring CostOfMig_BtoW]);
b_mr=(100*Reserves_max)/(Reserves_max-cstMigr);
a_mr=-100/(Reserves_max-cstMigr);
xtt=cstMigr:1:Reserves_max; ytt=a_mr.*xtt+b_mr;
if t_DepBre<=t_NestEnd; error('Nesting must end before t_DepBre'); end
% Time axis starts at wintering grounds i.e. t_DepBre+1
DoY=[t_DepBre+1:T,repmat(1:T,1,NoY-1),1:t_DepBre+1];
% OFFSPRING VALUE
offvalue=exp(-[0:(t_NestEnd-t_NestStrt)].*m_d);
offvalue_v=[zeros(1,length(t_DepBre+1:T)),...
    repmat([zeros(1,t_NestStrt-1),exp(-[0:(t_NestEnd-t_NestStrt)].*m_d),zeros(1
length(t_NestEnd+1:T))],1,NoY-1), ...
    [zeros(1,t_NestStrt-1),exp(-[0:(t_NestEnd-t_NestStrt)].*m_d),zeros(1,t_DepBre
t_NestEnd+1)]];
% BACKWARD OPTIMIZATION; 1-forage, 2-rest, 3-lay egg, 4-incubate, 5-migrate
Fitness=zeros(imaxReserv,length(DoY),Clutchsize_max+1,3);
Strategy=ones(imaxReserv,length(DoY),Clutchsize_max+1,3);
Fitness(:,:,2:end,2:3) = NaN; Strategy(:,:,2:end,2:3) = NaN;
for t = length(DoY)-1:-1:1
    for iReserv = 1:imaxReserv
        Reserves = (iReserv-1)/(imaxReserv-1) * Reserves_max;
        for iGL=1:3 % (1-breeding grounds, 2-stopover, 3-wintering grounds)
            FitIfFrgVct=zeros(1,lStG_Frg);
            for ilstF=1:lStG_Frg
                if DoY(t)<=t_NestEnd && iGL==1
                    Reserves_new = Reserves +  Vect_wghtGain(ilstF);
                else
                    Reserves_new = Reserves +  Vect_wghtGain(ilstF);
                end
                if (DoY(t)<=t_FPDepW ||  DoY(t)>t_DepBre)
                    if Reserves_new<=ResWinStr  || iGL==2
                        p_StrvOp=p_Starv; else; p_StrvOp=1; end
                else
                    if Reserves_new<=0; p_StrvOp=p_Starv; else; p_StrvOp=1; end
                end
                Condition_new = (Reserves_new/Reserves_max)*(imaxReserv-1)+1;
                intC = max(1, min(floor(Condition_new),imaxReserv-1));
                dC = max(0., min(Condition_new-intC , 1));
                FitFrgTmpF = (dC * Fitness(intC+1, t+1, 1, iGL) + (1-dC) * Fitnes
(intC, t+1, 1, iGL)) * p_forag.*p_StrvOp;
                FitIfFrgVct(ilstF) = FitFrgTmpF * exp(-Reserves_new.*c_d);
            end
            FitnessIfForag_0egs = sum(FitIfFrgVct.*PrbDsGn_Frge);
            FitIfRstVct=zeros(1,lStG_Rst);
            for ilstR=1:lStG_Rst
                Reserves_new = Reserves +  VlDst_Rest(ilstR);
                if (DoY(t)<=t_FPDepW ||  DoY(t)>t_DepBre)
                    if Reserves_new<=ResWinStr  || iGL==2
```

```
                    p_StrvOp=p_Starv; else; p_StrvOp=1; end
                else
                    if Reserves_new<=0; p_StrvOp=p_Starv;
                    else; p_StrvOp=1; end
                end
                Condition_new = (Reserves_new/Reserves_max)*(imaxReserv-1)+1;
                intC = max(1, min(floor(Condition_new),imaxReserv-1));
                dC = max(0., min(Condition_new-intC , 1));
                FitFrgTmpR = (dC * Fitness(intC+1, t+1, 1, iGL) + (1-dC) * Fitness
(intC, t+1, 1, iGL)) * p_rest.*p_StrvOp;
                FitIfRstVct(ilstR) = FitFrgTmpR*exp(-Reserves_new.*c_d);
            end
            FitnessIfRest_0egs = sum(FitIfRstVct.*PrbDsGn_Rest);
            if iGL~=1
                FitnessIfLay=0; FitnessIfIncub=0;
                if (Reserves>=CostOfMig_Spring) && (DoY(t)>t_FPDepW && DoY(t)
<t_DepBre)
                    Reserves_new_migNB = min([Reserves-CostOfMig_Spring  a_mr
*Reserves+b_mr]);
                    Condition_new = (Reserves_new_migNB/Reserves_max)*(imaxReserv-
1)+1;
                    intC = max(1, min(floor(Condition_new),imaxReserv-1));
                    dC = max(0., min(Condition_new-intC , 1));
                    if iGL==3
                        FitnessIfMigNB = (dC * Fitness(intC+1, t+1, 1, 2) + (1-dC)
* Fitness(intC, t+1, 1, 2)) * p_mig * exp(-Reserves_new_migNB.*c_d);
                    elseif iGL==2
                        FitnessIfMigNB = (dC * Fitness(intC+1, t+1, 1, 1) + (1-dC)
* Fitness(intC, t+1, 1, 1)) * p_mig * exp(-Reserves_new_migNB.*c_d);
                    end
                else
                    FitnessIfMigNB=0;
                end
                FitCompoNB=[FitnessIfRest_0egs FitnessIfForag_0egs FitnessIfLay
FitnessIfIncub FitnessIfMigNB];
                [~,ActNB]=max(FitCompoNB);
                Fitness(iReserv,t,1,iGL) = FitCompoNB(ActNB);
                Strategy(iReserv,t,1,iGL) = ActNB;
            elseif iGL==1
                for EggsInNest = 0:Clutchsize_max
                    idxEgInNe=EggsInNest+1;
                    if EggsInNest==0
                        FitnessIfForag=FitnessIfForag_0egs;
                        FitnessIfRest=FitnessIfRest_0egs;
                    else
                        FitnessIfForag = 0; FitnessIfRest = 0;
                    end
                    if (DoY(t)>=t_NestStrt && (DoY(t)+IncubT)<=t_NestEnd)
                        if (Reserves>=Eggweight && EggsInNest<Clutchsize_max)
                            Reserves_new_lay = Reserves - Eggweight + ResGain_Av;
                            Condition_new_Lay = (Reserves_new_lay/Reserves_max)*
(imaxReserv-1)+1;
                            intC = max(1, min(floor(Condition_new_Lay),imaxReserv
```

```
1));
                              dC = max(0., min(Condition_new_Lay-intC , 1));
                              FitnessIfLay = (dC * Fitness(intC+1, t+1, idxEgInNe+1↙
1) + (1-dC) * Fitness(intC, t+1, idxEgInNe+1, 1)) * p_lay * exp(-Reserves_new_lay↙
*c_d);
                         else
                              FitnessIfLay=0;
                         end
                         if (Reserves>=CostOfIncub)
                              DaysToDep=t_DepBre-DoY(t);
                              Reserves_new_incub = Reserves - CostOfIncub;
                              Condition_new_Incub = (Reserves_new_incub/Reserves_max↙
*(imaxReserv-1)+1;
                              intC = max(1, min(floor(Condition_new_Incub)↙
imaxReserv-1));
                              dC = max(0., min(Condition_new_Incub-intC , 1));
                              FitnessIfIncub=EggsInNest*offvalue_v(t+IncubT)↙
p_incub^IncubT  + ...
                                   (dC * Fitness(intC+1, t+1, 1, 1) + (1-dC) * Fitnes↙
(intC, t+1, 1, 1)) * p_forag^(DaysToDep-1);
                         else
                              FitnessIfIncub = 0;
                         end
                    else
                         FitnessIfLay = 0; FitnessIfIncub = 0;
                    end
                    if (DoY(t)>=t_DepBre || DoY(t)<t_FPDepW)
                         if (Reserves>=CostOfMig_BtoW)
                              Reserves_new_migBtoW = min([Reserves-CostOfMig_BtoW↙
a_mr.*Reserves+b_mr]);
                              Condition_new = (Reserves_new_migBtoW/Reserves_max)↙
(imaxReserv-1)+1;
                              intC = max(1, min(floor(Condition_new),imaxReserv-1));
                              dC = max(0., min(Condition_new-intC , 1));
                              FitnessIfMigBtoW = (dC * Fitness(intC+1, t+1, 1, 3) ↙
(1-dC) * Fitness(intC, t+1, 1, 3)) * p_mig * exp(-Reserves_new_migBtoW.*c_d);
                         else
                              FitnessIfMigBtoW=0;
                         end
                         FitnessIfRest=FitnessIfRest_0egs.*p_Starv;
                         FitnessIfForag=FitnessIfForag_0egs.*p_Starv;
                         FitnessIfLay=0; FitnessIfIncub=0;
                    else
                         FitnessIfMigBtoW=0;
                    end
                    if EggsInNest>0 && (DoY(t)<t_NestStrt || (DoY(t)+IncubT)↙
>t_NestEnd)
                         FitnessIfRest=0; FitnessIfForag=0; FitnessIfLay=0↙
FitnessIfIncub=0; FitnessIfMigBtoW=0;
                    end
                    FitCompo=[FitnessIfRest FitnessIfForag FitnessIfLa↙
FitnessIfIncub FitnessIfMigBtoW];
                    [~,Act]=max(FitCompo);
```

```
                    Fitness(iReserv,t,idxEgInNe,iGL) = FitCompo(Act);
                    Strategy(iReserv,t,idxEgInNe,iGL) = Act;
                end
            end
        end
    end
end
% FORWARD SIMULATIONS
ResStr=0; GeoStr=3; % condition and location at start
% The trajectory for the expected food intake is added as the last row of matrices
Reserv_Trj=[ones(N+1,1).*ResStr,zeros(N+1,length(DoY)-1)];
GeoLoc_Trj=[ones(N+1,1).*GeoStr, zeros(N+1,length(DoY)-1)];
Decision_Trj=zeros(N+1,length(DoY));
SurvProb_Trj=zeros(N+1,length(DoY));
EggsInNest_Trj=zeros(N+1,length(DoY));
IncubLasts=zeros(N+1,length(DoY)); % flag for incubation
ResCFrFoodIt_Trj=zeros(N+1,length(DoY));
csm_DstGainFrg=cumsum(PrbDsGn_Frge);
csm_DstGainRst=cumsum(PrbDsGn_Rest);
for t_f = 1:length(DoY)-1
    CurrResLev = (Reserv_Trj(:,t_f)./Reserves_max).*(imaxReserv-1)+1;
    intC = max([ones(N+1,1) min([floor(CurrResLev) ones(N+1,1).*(imaxReserv-1)],[]↙
2)],[],2);
    dC = max([zeros(N+1,1) min([CurrResLev-intC , ones(N+1,1)],[],2)],[],2);
    idxEgInNe_trj=EggsInNest_Trj(:,t_f)+1;
    for ij=1:N+1
        [~,imx]=max([dC(ij) * Fitness(intC(ij)+1, t_f+1, idxEgInNe_trj(ij)↙
GeoLoc_Trj(ij,t_f)), (1-dC(ij)) * Fitness(intC(ij), t_f+1, idxEgInNe_trj(ij)↙
GeoLoc_Trj(ij,t_f))]);
        ActBoth=[Strategy(intC(ij)+1, t_f+1, idxEgInNe_trj(ij), GeoLoc_Trj(ij↙
t_f)), Strategy(intC(ij), t_f+1, idxEgInNe_trj(ij), GeoLoc_Trj(ij,t_f))];
        ActTmp=ActBoth(imx);
        if IncubLasts(ij,t_f)~=true
            switch ActTmp
                case 1
                    GeoLoc_Trj(ij,t_f+1)=GeoLoc_Trj(ij,t_f);
                    if ij<=N
                        WghtGnRs=VlDst_Rest(sum(csm_DstGainRst<rand(1))+1);
                    else
                        WghtGnRs=VlDst_Rest;
                    end
                    ResCFrFoodIt_Trj(ij,t_f)=WghtGnRs;
                    Reserv_Trj(ij,t_f+1)=Reserv_Trj(ij,t_f) + WghtGnRs;
                    Decision_Trj(ij,t_f)=1;
                case 2
                    if ij<=N
                        WghtGnFr=Vect_wghtGain(sum(csm_DstGainFrg<rand(1))+1);
                    else
                        WghtGnFr=ResGain_Av;
                    end
                    if DoY(t_f)<=t_NestEnd && GeoLoc_Trj(ij,t_f)==1
                        ResCFrFoodIt_Trj(ij,t_f)=WghtGnFr;
                        Reserv_Trj(ij,t_f+1)=Reserv_Trj(ij,t_f) +  WghtGnFr;
```

```
                    else
                        ResCFrFoodIt_Trj(ij,t_f)=WghtGnFr;
                        Reserv_Trj(ij,t_f+1)=Reserv_Trj(ij,t_f) +  WghtGnFr;
                    end
                    GeoLoc_Trj(ij,t_f+1)=GeoLoc_Trj(ij,t_f);
                    Decision_Trj(ij,t_f)=2;
                case 3
                    ResCFrFoodIt_Trj(ij,t_f)=ResGain_Av;
                    GeoLoc_Trj(ij,t_f+1)=GeoLoc_Trj(ij,t_f);
                    Reserv_Trj(ij,t_f+1)=Reserv_Trj(ij,t_f)- Eggweight ↙
ResGain_Av;
                    Decision_Trj(ij,t_f)=3;
                    EggsInNest_Trj(ij,t_f+1)=EggsInNest_Trj(ij,t_f)+1;
                case 4
                    ResCFrFoodIt_Trj(ij,t_f)=0;
                    GeoLoc_Trj(ij,t_f+1)=GeoLoc_Trj(ij,t_f);
                    Reserv_Trj(ij,t_f+1)=Reserv_Trj(ij,t_f) - CostOfIncub/IncubT;
                    Decision_Trj(ij,t_f)=4;
                    EggsInNest_Trj(ij,t_f+1)=0;
                    IncubLasts(ij,t_f:t_f+IncubT-1)=true;
                case 5
                    % 1-breeding grounds, 2-stopover, 3-wintering grounds
                    if GeoLoc_Trj(ij,t_f)==1
                        GeoLoc_Trj(ij,t_f+1)=3;
                        Reserv_Trj(ij,t_f+1)=min([Reserv_Trj(ij,t_f)-CostOfMig_Bto↙
a_mr.*Reserv_Trj(ij,t_f)+b_mr]);
                    elseif GeoLoc_Trj(ij,t_f)==2
                        GeoLoc_Trj(ij,t_f+1)=1;
                        Reserv_Trj(ij,t_f+1)=min([Reserv_Trj(ij,t_f)↙
CostOfMig_Spring a_mr.*Reserv_Trj(ij,t_f)+b_mr]);
                    elseif GeoLoc_Trj(ij,t_f)==3
                        GeoLoc_Trj(ij,t_f+1)=2;
                        Reserv_Trj(ij,t_f+1)=min([Reserv_Trj(ij,t_f)↙
CostOfMig_Spring a_mr.*Reserv_Trj(ij,t_f)+b_mr]);
                    end
                    Decision_Trj(ij,t_f)=5;
            end
        else
            GeoLoc_Trj(ij,t_f+1)=GeoLoc_Trj(ij,t_f);
            Reserv_Trj(ij,t_f+1)=Reserv_Trj(ij,t_f) - CostOfIncub/IncubT;
            Decision_Trj(ij,t_f)=4;
            EggsInNest_Trj(ij,t_f+1)=EggsInNest_Trj(ij,t_f);
            ResCFrFoodIt_Trj(ij,t_f)=0;
        end
    end
    if any(Reserv_Trj(:,t_f)<0)
        idmn0=Reserv_Trj(:,t_f)<0;
        Reserv_Trj(idmn0,t_f)=0;
    end
end
YolFem_Trj=Reserv_Trj; %Reserves used for reproduction only - calculations
YolFem_Trj(:,1)=0;
for iv1=1:size(Reserv_Trj,1)
```

```
    EgLL=0; CountON=false;
    for iv2=size(Reserv_Trj,2):-1:2
        if CountON==false
            YolFem_Trj(iv1,iv2)=0;
        end
        if Decision_Trj(iv1,iv2)==3
            EgLL=EgLL+1;
            CountON=true;
            YolFem_Trj(iv1,iv2)=EgLL*Eggweight;
            MxResTmp=Reserv_Trj(iv1,iv2);
        elseif Decision_Trj(iv1,iv2)==4
            YolFem_Trj(iv1,iv2)=0;
        end
        if Decision_Trj(iv1,iv2-1)~=3 && CountON==true
            ThrResLv=MxResTmp-EgLL*Eggweight;
            if Reserv_Trj(iv1,iv2)-ThrResLv>=0
                YolFem_Trj(iv1,iv2)=Reserv_Trj(iv1,iv2)-ThrResLv;
            else
                EgLL=0;
                CountON=false;
                YolFem_Trj(iv1,iv2)=0;
            end
        end
    end
end
% SLOPE AND INTERCEPT OF REGRESSIONS (muscle shell length vs. thiamine
% concentration per g of whole mussel mass) - see Appendix S1
SlopeLocWint=-0.030942516577439; % wintering grounds
InterceptLocWint=0.266735466616556; % wintering grounds
SlopeLocStOv=-0.061163357113658; % stopover
InterceptLocStOv=0.463339363251470; % stopover
SlopeLocBrGr=-0.107403742055564; % breeding grounds
InterceptLocBrGr=0.502759340100576; % breeding grounds
AvThInt_Wint=zeros(1,length(CnsMusL));
AvThInt_StOv=zeros(1,length(CnsMusL));
AvThInt_Bree=zeros(1,length(CnsMusL));
for iCML=1:length(CnsMusL)
    AvThInt_Wint(iCML) = SlopeLocWint.*CnsMusL(iCML) + InterceptLocWint;
    AvThInt_StOv(iCML) = SlopeLocStOv.*CnsMusL(iCML) + InterceptLocStOv;
    AvThInt_Bree(iCML) = SlopeLocBrGr.*CnsMusL(iCML) + InterceptLocBrGr;
end
% TRAJECTORIES - FORWARD SIMULATION
FoGa_Trj=ResCFrFoodIt_Trj.*Res2Food; % Daily food gain
FoGa_Trj(FoGa_Trj<0)=0;
InEggTh=zeros(N+1,length(CnsMusL)); FlInEggTh=true(N+1,length(CnsMusL));
ThConc_Trj=zeros(N+1,length(DoY),length(CnsMusL));
ThConc_Trj(:,1,:)=AvThS_L;
for iCML=1:length(CnsMusL)
    ThInt_Trj=GeoLoc_Trj;
    ThInt_Trj(GeoLoc_Trj==3)=AvThInt_Wint(iCML);
    ThInt_Trj(GeoLoc_Trj==2)=AvThInt_StOv(iCML);
    ThInt_Trj(GeoLoc_Trj==1)=AvThInt_Bree(iCML);
    ThInt_Trj=ThInt_Trj.*FoGa_Trj.*ThBioav; %Thiamine consumed, digested and↙
```

```
assimilated
    %Calculation of thiamine concentration in lean body and yolk
    for t_f = 1:length(DoY)-1
        idmn0=ThConc_Trj(:,t_f,iCML)<0;
        ThConc_Trj(idmn0,t_f,iCML)=0;
        idmn1=ThConc_Trj(:,t_f,iCML)>=Cx;
        ThConc_Trj(idmn1,t_f,iCML)=Cx;
        flIncNxtD=Decision_Trj(:,t_f)==4;
        xThBas=ThConc_Trj(:,t_f,iCML);
        ThBRK=zeros(size(xThBas));
        ThBRK(xThBas<ThrBr) = a.*xThBas(xThBas<ThrBr).^b;
        ThBRK(xThBas>=ThrBr)=mxBR.*(xThBas(xThBas>=ThrBr)./Cx);
        ThCoInt=ThInt_Trj(:,t_f)./(LeanBody+YolFem_Trj(:,t_f));
        ThConc_Trj(~flIncNxtD,t_f+1,iCML)=ThConc_Trj(~flIncNxtD,t_f,iCML) +ThCoInt
(~flIncNxtD) -ThBRK(~flIncNxtD);
        ThConc_Trj(flIncNxtD,t_f+1,iCML)=ThConc_Trj(flIncNxtD,t_f,iCML);
        Fltmp=FlInEggTh(:,iCML) & Decision_Trj(:,t_f)==3;
        InEggTh(Fltmp,iCML)=ThConc_Trj(Fltmp,t_f,iCML);
        FlInEggTh(Fltmp,iCML)=false;
    end
end
scrsz = get(0,'ScreenSize');
wd_f=scrsz(3)/1.8;
hd_f=scrsz(4)/1.9;
hand1=figure('Position',[50 50 wd_f hd_f]);
flb=7.5; fax=5;
pn1=axes('Position',[0.085 0.62 0.37 0.29]); % Reserves trajectories
pn2=axes('Position',[0.085 0.085 0.37 0.47]); % Thiamine concentration
pn3=axes('Position',[0.57  0.085 0.37 0.80]); % Egg thiamine disitrbution
clmb=[0 0.2 0.8; 0 0.8 0.2; 1 0.8 0.2; 0.5 0.5 0.5; 0.8 0 0];
colormap(clmb)
BRc=[0 1 0.5]; %color - breeding
STOVc=[1 0.5 0]; %color - stopover
WINc=[0 1 1]; %color - wintering
criBg=[]; criEd=[]; flgB=0; flgE=0; GeoLocDet=[];
for cri=2:size(GeoLoc_Trj,2)-1
    if flgB==0
        if GeoLoc_Trj(end,cri-1)==GeoLoc_Trj(end,cri)
            GeoLocDet=[GeoLocDet GeoLoc_Trj(end,cri)];%#ok
            criBg=[criBg cri-1]; flgB=1; flgE=0;%#ok
        end
    end
    if flgE==0
        if GeoLoc_Trj(end,cri)~=GeoLoc_Trj(end,cri+1)
            criEd=[criEd cri]; flgB=0; flgE=1;%#ok
        end
    end
end
criEd=[criEd size(GeoLoc_Trj,2)];
axes(pn1) % ## RESERVES
plot(-500,-500,'-','color',WINc,'Linewidth',8)
hold on
plot(-500,-500,'-','color',STOVc,'Linewidth',8)
```

```
plot(-500,-500,'-','color',BRc,'Linewidth',8)
plot(-500,-500,'ok','markerFaceColor','w','MarkerSize',3.5)
tmp_lg=legend('Wintering','Stopover','Breeding','Egg↙
laid','orientation','horizonthal','AutoUpdate','off');
set(tmp_lg,'position',[0.17 0.94 .20 0.05],'box','off','fontsize',fax+2);
for drRe=1:length(GeoLocDet)
    switch GeoLocDet(drRe)
        case 1
            area([criBg(drRe) criEd(drRe)],[Reserves_max Reserves_max],'FaceColor',↙
BRc,'EdgeColor','none')
        case 2
            area([criBg(drRe) criEd(drRe)],[Reserves_max Reserves_max],'FaceColor',↙
STOVc,'EdgeColor','none')
        case 3
            area([criBg(drRe) criEd(drRe)],[Reserves_max Reserves_max],'FaceColor',↙
WINc,'EdgeColor','none')
    end
    hold on
end
plot(repmat(1:length(DoY),N,1)',Reserv_Trj(1:end-1,:)','Color',[0.8 0.8 0.8])
plot(1:length(DoY),Reserv_Trj(end,:),'-k','Linewidth',1)
xao=1:length(DoY);
for t_f=1:length(Decision_Trj(end,:))
    if Decision_Trj(end,t_f)==3
        plot(xao(t_f+1),Reserv_Trj(end,↙
t_f+1),'ok','markerFaceColor','w','MarkerSize',3.5)
    end
end
set(gca,'xtick',1:60:length(DoY),'xticklabel',DoY(1:60:length(DoY)))
axis([1 1.4*T -0.5 Reserves_max.*1.01])
set(gca,'box','off','fontsize',fax)
ylabel('Reserves (g)','Fontsize',flb)
clBThEg=[0 0.2 0.8; 0 0.8 0.2; 1 0.8 0.2; 0.5 0.5 0.5; 0.8 0 0];
axes(pn2) % THIAMINE CONCENTRATION IN LEAN BODY (TRAJECTORY)
%Legend
lg='';
for iCML=1:length(CnsMusL)
    plot(1:length(DoY),ThConc_Trj(end,:,iCML),'color',clBThEg(iCML,:),'Linewidth',↙
4) % individual with average (deterministic) reserves gain
    hold on
    if iCML==1
        lg=[lg,'''',num2str(CnsMusL(iCML)),' cm''']; %#ok
    else
        lg=[lg,',''',num2str(CnsMusL(iCML)),' cm''']; %#ok
    end
end
eval(['tmp_lg2=legend(',↙
lg,',''orientation'',''horizonthal'',''AutoUpdate'',''off'');']);
set(tmp_lg2,'position',[0.68 0.94 .15 0.05],'box','off','fontsize',fax+2);
for drRe=1:length(GeoLocDet)
    switch GeoLocDet(drRe)
        case 1 %breeing grounds
            area([criBg(drRe) criEd(drRe)],[Cx Cx],'FaceColor',↙
```

```
BRc,'EdgeColor','none')
        case 2 %stopover site
            area([criBg(drRe) criEd(drRe)],[Cx Cx],'FaceColor',↙
STOVc,'EdgeColor','none')
        case 3 %wintering grounds
            area([criBg(drRe) criEd(drRe)],[Cx Cx],'FaceColor',↙
WINc,'EdgeColor','none')
    end
    hold on
end
for iCML=length(CnsMusL):-1:1
    plot(1:length(DoY),ThConc_Trj(end,:,iCML),'color',clBThEg(iCML,:),'Linewidth',↙
1) % individual with average (deterministic) reserves gain
    hold on
end
set(gca,'xtick',1:60:length(DoY),'xticklabel',DoY(1:60:length(DoY)))
axis([1 1.4*T min(ThConc_Trj(:)) max(ThConc_Trj(:)).*1.01])
set(gca,'box','off','fontsize',fax)
ylabel('Thiamine in females (nmol/g)','Fontsize',flb)
xlabel('Time (Day of year)','Fontsize',flb)
axes(pn3) % ### DISTRIBUTION OF THIAMINE IN THE EGGS
mnThEg=min(InEggTh(:)); mxThEg=max(InEggTh(:));
intThE=(mxThEg-mnThEg)/80;
binEdgThEg=mnThEg-intThE:intThE:mxThEg+intThE;
binCentrThEg=binEdgThEg(1:end-1)+intThE/2;
Fr_InEggTh=zeros(length(CnsMusL),length(binCentrThEg));
for iCML=1:length(CnsMusL)
    [Fr_InEggTh(iCML,:)] = histcounts(InEggTh(:,iCML),binEdgThEg);
end
for iCML=length(CnsMusL):-1:1
    hh=bar(binCentrThEg,Fr_InEggTh(iCML,:)./N);
    hh.FaceColor=clBThEg(iCML,:);
    hh.EdgeColor='none';
    hold on
end
xlim([min(binEdgThEg) max(binEdgThEg)])
set(gca,'box','off','Fontsize',fax)
xlabel('Egg thiamine (nmol/g)','Fontsize',flb)
ylabel('Fraction of eggs in the population','Fontsize',flb)
set(hand1,'PaperUnits','centimeters','Papersize',[16 8.5],'Paperposition',[0 0 16↙
8.5]);
```
