# Supplementary material for: The risk of thiamine deficiency in common eider ducklings increases when females consume large prey
Source: PLoS One. 2026 Sep 16;21(9):e0356777. doi: 10.1371/journal.pone.0356777 (PMC13580947; doi:10.1371/journal.pone.0356777)
Supplement: Supplementary file 3 [file pone.0356777.s003.pdf]

Reviewer Comments to Authors
Manuscript ID: PONE-D-25-50236

Selection for large prey increases risk of mass juvenile mortality due to thiamine deficiency

by Maciej Jan Ejsmond, Anna Ejsmond, Marc M. Hauber, Jón Einar Jónsson, Andreas M. Waser & Samuel Hylander

This manuscript investigates how size selection of prey (blue mussels *Mytilus edulis trossulus*; hereafter mussels) may influence thiamine (vitamin B1) status in consumers (common eiders *Somateria mollissima*; hereafter eiders) and thereby potentially influence offspring survival, combining biochemical measurements of thiamine concentrations in Baltic blue mussels with a dynamic state-variable life-history model of thiamine dynamics in female eiders depending on breeding phenology and fecundity. Mussel assays across wintering, stopover, and breeding areas show that mass-specific thiamine content declines with increasing shell length; these measurements then parameterize scenarios that vary dietary bioavailability and renal excretion. Model results indicate that consumption of small rather than large mussels was associated with lower predicted egg thiamine, whereas producing a smaller clutch may partially elevate egg thiamine, and these patterns seemed robust to alternative assumptions about seasonal timing, mortality, and food intake. The broader implication is that choosing larger prey may trade energetic gains against essential micronutrient intake, suggesting that the mussel size distribution and size-dependent micronutrient content of prey may potentially shape reproductive output. A strength of the study is the careful analytical work on blue mussels and the linkage of the biochemical data to calculations in the modelling framework.

That being said, however, I have several serious concerns with the manuscript, and I suggest several potential improvements, as detailed below.

The following issues are of major concern:

1) Overstatement of the extent and severity of thiamine deficiency syndromes, particularly in birds, a predilection for tendentiousness, and a lack of defined thresholds for implying detrimental effects.

The manuscript's framing overreaches on scope and causality, beginning with an Abstract that asserts "episodic mass mortality… is a major threat to top marine consumers worldwide," and a title and keywords implying population-level risk—without bird- or eider-specific evidence to support such general claims. Much of the cited support comes from fish or grey literature

and does not rule out dominant alternative drivers (e.g., predation), while the manuscript also lacks a clear, species-relevant yolk-thiamine threshold for inferring detrimental effects. The choice of language throughout (e.g., linking egg thiamine to nest predation, implying adaptive clutch-size responses to thiamine) invites causal inference where only associations are indicated and where key mechanisms remain untested. To remedy the situation, it would be important to temper the rhetoric, aligning claims to demonstrated scope, and explicitly stating any biologically justified thresholds.

2) Unconvincing leap from mussel thiamine to eider reproduction, including mismatches between the model and the theoretical and empirical evidence base.

While the mussel assays are careful, the step from size-dependent thiamine contents in prey to consequences for eider reproduction rests on assumptions and generalizations that are weakly supported or at odds with established theory and data. More specifically, demographic sensitivity in this long-lived species depends more on adult survival than fecundity; claims about large-brained females taking larger prey and thereby trading micronutrients are not robust; "preference" for prey sizes is assumed rather than estimated; key life-history settings (mixed capital breeding, migration/stopover use, feeding during early laying) are simplified; prey-size shifts are modelled at unrealistic increments; and the breeding phenology–clutch size relationship is inverted relative to empirical patterns. Several strong statements (e.g., "unavoidable trade-off," generality beyond the study system) also outrun the analyses. Altogether, the model can illustrate potential mechanisms but does not convincingly demonstrate population-relevant reproductive effects without stronger empirical anchoring or sensitivity checks aligned with the empirical reality.

3. Alternative explanations for the mussel size–thiamine pattern may limit temporal generality.

The observed decrease in thiamine with increasing mussel size is plausibly explained by metabolic scaling, but an equally plausible (and testable) alternative is size-dependent reproductive status at the time of sampling: smaller (often immature) mussels sampled in June may retain higher thiamine, whereas larger, mature individuals near/after spawning can be depleted. Accordingly, this may imply a seasonal, phenology-linked signal rather than a universal year-round size effect. If so, the inferred disadvantage of "large prey" for thiamine intake (and any cascading conclusions for eiders) would be temporally constrained; acknowledging this and, where possible, controlling for maturity/gonadal stage would sharpen inference and clarify scope.

Here are my more specific comments, questions and suggested changes to the manuscript:

1. L. 1, Title: The title implies a causal, population-level link to "mass juvenile mortality," yet the study only models diet-size effects on egg thiamine; it does not measure or estimate juvenile mortality rates, survival probabilities, or population-level risk (e.g., the probability of mass-mortality events). The Abstract notes that the link between thiamine deficiency and predation risk is unclear (LL. 32-33). Your findings are system-specific model predictions; the most pertinent result is lower modelled egg thiamine if consuming larger mussels, not demonstrated mortality. The title should reflect the contents of the manuscript.

Here are two suggestions for more objective titles:

Consumption of larger blue mussels is associated with lower modelled egg-thiamine levels in Baltic common eiders

Feeding on larger blue mussels is associated with lower model-predicted egg-thiamine levels in Baltic common eiders

2. Abstract, LL. 29-30: The first sentence of the Abstract is an overstatement. There is evidence for negative effects of thiamine deficiency in fish such as salmonids, while the link between thiamine levels and reproductive output in common eiders is weak at best. I will detail in my comments below why the supportive evidence is not very convincing.

3. Abstract, LL. 30-32: The sentence "Recent reports of thiamine deficiency…" needs to be omitted, as it is tendentious and speculative, for the reasons detailed in my comments below.

4. Abstract, LL. 32-34: First, it is unclear to me why we should expect egg thiamine status to *directly* raise nest predation. Are predators expected to detect nests with eggs having low thiamine levels?

Second, it is pertinent to note that in long-lived birds, population growth (λ) is most sensitive to adult survival rather than fecundity (e.g., Sæther and Bakke 2000). Consistent with this, several recent studies have indicated that the recent decline and the increasingly male-biased adult sex ratio are best explained by increased predation-induced mortality of breeding females rather than nest predation or fecundity variation alone (e.g., Öst et al. 2016; Ramula et al. 2018; Tjørnløv et al. 2020).

Can you please rephrase this sentence to address the above concerns?

References:
Öst M, Ramula S, Lindén A, Karell P, Kilpi M. 2016. Small-scale spatial and temporal variation in the demographic processes underlying the population decline of eiders in the Baltic Sea. Pop Ecol. 58: 121–133.

Ramula S, Öst M, Linden A, Karell P, Kilpi M. 2018. Increased male bias in eider ducks can be explained by sex-specific survival of prime-age breeders. PLoS One 13:e0195415.
Sæther BE and Bakke Ø. 2000. Avian life history variation and contribution of demographic traits to the population growth rate. Ecology 81: 642-653.
Tjørnløv RS, Ens BJ, Öst M, Jaatinen K, Karell P, Larsson R, Christensen TK, Frederiksen M. 2020. Drivers of spatiotemporal variation in survival in a flyway population: a multi-colony study. Frontiers in Ecology and Evolution 8: 566154.

5. Abstract, LL. 35-36: The evidence that eiders with larger brains feed on larger mussels is not backed up my compelling evidence, so the argument presented here is in need of revision. Please see my comments below for more details.

6. Abstract, L. 36: You do not investigate size selection of blue mussels in this study, so please replace “whether selection for larger prey” with “whether consumption of larger prey”.

7. Abstract, LL. 37-38: This is not a hypothesis in the traditional sense; it describes the approach adopted. I suggest rewriting this as, for example:

We predicted a negative association between pre-breeding mean mussel size in the diet and modelled egg-thiamine levels, since larger mussels may have a lower thiamine concentration per unit mass due to allometric scaling of metabolism.

8. Abstract, L. 41, “reduced thiamine levels”: Your study is an observational one, so you cannot infer causality. This applies to the whole manuscript. Here, for example, please rephrase as: “was associated with lower thiamine levels”.

9. Abstract, L. 44-45: The phrasing suggests females adjust clutch size in response to egg thiamine, which is implausible. Any observed changes in clutch size in this eider population are better explained by empirically documented ecological factors such as predation risk, as detailed in my comments below.

10. Abstract, L. 44-46: This sentence is overly speculative and should either be omitted, or at least entirely rewritten. Thus, your study does not measure female anti-predator ability, nest predation, or offspring survival. What your results show is only an association between mussel size and modelled egg-thiamine; referring to a mechanism whereby females “best able to cope with predation” produce thiamine-deficient offspring is untested.

11. Abstract, LL. 46-47: See my comment 9 above.

12. Abstract, LL. 47-48: You have not tested the preference (i.e., size selection) of prey in your study, so this part needs to be rephrased.

13. Keywords: Remove the word “mass juvenile mortality" as a keyword – it is misleading and tendentious.

14. LL. 56-58: In my view, the PNAS News Feature is grey literature that reports a debate rather than delivering a systematic, peer-reviewed synthesis, and it explicitly acknowledges major gaps which preclude global inference. The most coherent causal story remains the Great Lakes salmonines (alewife thiaminase → low egg thiamine → recruitment failure, mitigation via thiamine supplementation and recovery as alewife declined), but this is a regional case study, not a worldwide pattern.

By contrast, for Baltic common eiders the feature describes smaller clutches and thiamine-deficient eggs, yet more than 90% of duckling losses occurred through herring gull predation after normal hatching, i.e., this case study does not rule out predation as the proximate driver, leaving population-level causation by thiamine deficiency unproven.

More broadly, critics quoted in the article argue that links to population declines are not well established and that other causes (e.g., disease, contaminants) can mimic neurological symptoms; the article itself frames the “global crisis” idea as a hypothesis needing more research. Consequently, this News Feature cannot, on its own, substantiate the strong claim that episodic thiamine-driven juvenile mortality of top marine consumers is a serious conservation problem worldwide. To conclude, you need to temper this statement.

15. LL. 65-69: The sentence overestimates the extent and certainty of these phenomena from local, circumstantial observations, and thus needs to be tempered. In long-lived eiders, very large year-to-year variation in duckling production is common, and in the Baltic Sea, this variation is now strongly and increasingly shaped by predation – currently overriding climatic and other bottom-up signals (Öst et al. 2022) – so low productivity in a given year and/or site is not diagnostic of a single cause. Furthermore, disease outbreaks may also demonstrably produce severe duckling losses in the region (e.g., Hollmén et al. 2002), further cautioning against single-cause explanations.

Furthermore, the main evidence cited for “mass juvenile mortality” rests on observations from one coastal area and an injection-rescue experiment on artificially incubated ducklings (Mörner et al. 2017). While instructive about thiamine physiology, those results may have limited external validity to field settings with intense predation; carcasses were rarely found

and gull predation was frequently observed, making the link to thiamine deficiency in the wild necessarily indirect.

Finally, reduced clutch size is attributed to thiamine deficiency by comparison with historical datasets (Mörner et al. 2017), but clutch size and other breeding decisions are also adjusted to prevailing predator pressure (e.g., Mohring 2025) – an increasingly dominant driver in this system – so confounding by predation and concomitant changes in breeding strategies, reproductive investment and predator-induced partial or complete clutch loss cannot be ruled out.

References:

Hollmén T, Franson JC, Kilpi M, Docherty D, Hansen WR, Hario M (2002) Isolation and characterization of a reovirus from common eiders (*Somateria mollissima*) from Finland. Avian Diseases 46: 478-484.
Mohring B. 2023. Breeding during a predation regime shift: behavioural and physiological responses of female common eiders facing the recovery of their native predator (Doctoral dissertation, Université de La Rochelle & Åbo Akademi University).
Mörner T, Hansson T, Carlsson L, Berg AL, Ruiz Muños Y, Gustavsson H, Mattson R, Balk L. 2017. Thiamine deficiency impairs common eider (*Somateria mollissima*) reproduction in the field. Sci Rep 7:14451.
Öst M, Lehikoinen A, Jaatinen K. 2022. Top-down effects override climate forcing on reproductive success in a declining sea duck. Oikos 2022: e08762.

16. LL. 70-71: You need to moderate the claim about episodic mass juvenile mortality of eiders in the Baltic Sea, and particularly any insinuation that these are causally linked to thiamine deficiency. See, for example, my previous comment 15.

17. LL. 73-75: This sentence misrepresents the evidence for the decline of the Baltic eider population. In long-lived birds such as eiders, population growth (λ) is most sensitive to adult survival rather than fecundity (Öst et al. 2016). Thus, recent work indicates that elevated predation-induced mortality of incubating females, also reflected in the increasingly male-biased adult sex ratio, better explains recent declines than nest predation or duckling mortality alone (Öst et al. 2016; Ramula et al. 2018; Tjørnløv et al. 2020). Please revise accordingly. For example:

"Increased predation on breeding females, together with nest predation and duckling mortality, likely underlies recent negative trends in the Baltic population."

References: see comment 4 above.

18. LL. 77-79: This sentence is cryptic. Please consider rewording it for increased transparency. For example:

“In Baltic eiders, individual differences in brain (central nervous system) size and female behavior are associated with variation in nesting success under predation pressure (Öst and Jaatinen 2015; Jaatinen et al. 2019).”

19. LL. 79-81: This sentence misinterprets both the findings and mechanism presented in the cited work. Öst and Jaatinen (2015) examined nest success, timing of breeding, and post-hatch coalition formation—not recruitment—and formation of brood-rearing coalitions occurs after hatching, affecting duckling survival rather than pre-hatch nest success.

Instead, the evidence shows that relatively large-headed (large-brained) females breed later and use more cautious nest-site selection; this delay confers higher nest success specifically in ‘dangerous’ years when predation pressure peaks early, while the reverse holds in benign years with early phenology (Jaatinen et al. 2019). During such conditions, large-headed females also have a survival advantage, which is reversed under relaxed predation pressure (Jaatinen et al. 2019). To conclude, please revise this section to avoid invoking recruitment and to attribute the pattern to brain-size-dependent timing of breeding rather than coalition speed.

20. LL. 82-85: This section is not a fair representation of what these studies actually found, so this part needs to be rewritten. Jaatinen et al. (2019) did not show a general survival advantage for large-brained females; rather, survival selection on relative head/brain size fluctuated annually—large-headed females out-survived small-headed ones in dangerous years, whereas the reverse held in benign years, consistent with brain-size-dependent breeding phenology as the mechanism. By contrast, the Nordman MSc thesis (2024) MSc does not address relative head/brain size; it analyzes potential temporal change in structural size (radius-ulna), finding an increase over 2008–2023 and no evidence for size-selective predation on incubating females. It therefore cannot be used to infer a brain–body size correlation.

21. LL. 85-87: First, “the superiority of females with large brains under high predation” is an overinterpretation that needs to be tempered (see my comments 19 and 20 above). Second, this sentence overstates what Laursen & Møller (2021) shows. In that study, prey-size data came from gizzard contents, and the core predictor was head volume as a proxy for brain mass. Importantly, the inference is about what was eaten, not observed *preference* relative to local availability. The GLMs included did not include predation intensity as a predictor—and the sample comprised 198 eiders collected in a single Danish wintering region. For blue mussels, head volume was positively associated with mean and maximum mussel size, but the authors’ across-prey effect size (estimate ≈ 0.10 ± 0.02) is not readily interpretable as birds “prefer considerably larger” prey. Critically, the result was not female-specific: the sex term was non-significant for blue mussels, and where sex differences did occur, female gizzards

contained smaller prey. Thus, Laursen & Møller (2021) paper does not demonstrate that females with larger brains prefer considerably larger prey, nor that such an association is linked with predation intensity.

22. LL. 87-88: Baltic eiders are not exceptional in their preference for blue mussels; they are opportunistic feeders, and where blue mussels are abundant, like in the Baltic Sea, but also elsewhere (e.g. Guillemette et al. 1992), they are the preferred prey.

Reference:

Guillemette M, Ydenberg RC, Himmelman JH. 1992. The role of energy intake rate in prey and habitat selection of common eiders in winter: a risk-sensitive interpretation. J Anim Ecol 61:599–610.

23. LL. 91-93: This statement rests on several unsupported assumptions. First, as explained in comment 21 above), Laursen and Møller (2021) did not measure active prey-size preference or selection relative to local availability; their inference is from gizzard contents only, with no concurrent sampling of benthic size distributions. Second, the study was conducted with no measure of predation intensity included among predictors, so the claim that larger mussel consumption is adaptive under increased predation at nesting sites is outside the evidence base. Please rephrase this section for retaining objectivity.

24. L. 94: Please consider replacing 'preference for' with 'consumption of', since you did not investigate prey preference in your study.

25. LL. 97-101: An interested question: your optimization framework seems tailored to eiders remaining near colonies year-round, yet most Baltic eiders are migratory mixed capital breeders whose diet, and therefore mussel size distributions, shift seasonally between wintering and breeding areas (Hobson et al. 2015, Jaatinen et al. 2016). Individuals also vary in the balance of 'imported' verus local reserves, with early-laying, good-condition females relying more on stores gathered at the wintering quarters, while others feed more locally prior to laying (Jaatinen et al. 2016). Moreover, predation risk has lengthened the spring arrival-to-nesting interval in northern parts of the Baltic Sea (Öst et al. 2022), expanding the scope for pre-laying foraging at the breeding locales.

Given all the above complexity, could you clarify how your model accommodates (or could be extended to accommodate) these axes of heterogeneity, i.e., seasonal prey-size shifts across areas, state-dependent capital versus income use, and variable arrival-to-lay intervals, and whether your central result that small shifts toward larger mussels depress egg thiamine remains robust under such realistic variation?

References:

Hobson KA, Jaatinen K, Öst M. 2016. Differential contributions of endogenous and exogenous nutrients to egg components in wild Baltic common eiders (*Somateria mollissima*): A test of alternative stable isotope approaches. Auk 132:624‑633.

Jaatinen K, Öst M, Hobson KA. 2016. State-dependent capital and income breeding: A novel approach to evaluating individual strategies with stable isotopes. Frontiers in Zoology, 13: 24.

Öst M, Lehikoinen A, Jaatinen K. 2022. Top‑down effects override climate forcing on reproductive success in a declining sea duck. Oikos 2022:e08762.

26. LL. 103-104: In my opinion, these empirical observations represent the most valuable contributions of this study. The biochemical analyses seem meticulously conducted, and the illustrations included are impressive.

27. LL. 108-111: One potentially important factor that was not considered in this study was the state of sexual maturation/gonadal development of the analyzed mussels, which may affect not only the spatial variation in thiamine levels, but, more importantly, the observed size-dependence of thiamine levels in mussels. Please consult my comment 41 below for more detailed discussion.

28. LL. 120-122: More recent research has provided a more nuanced view of the breeding strategies in this species; it is nowadays considered a mixed capital breeder, relying both on reserves acquired at the wintering grounds and on food ingested at the breeding sites for financing the costs of egg production and incubation (Sénéchal et al. 2011, Hobson et al. 2015, Jaatinen et al. 2016).

References:

Hobson KA, Jaatinen K, Öst M. 2016. Differential contributions of endogenous and exogenous nutrients to egg components in wild Baltic common eiders (*Somateria mollissima*): A test of alternative stable isotope approaches. Auk 132:624‑633.

Jaatinen K, Öst M, Hobson KA. 2016. State-dependent capital and income breeding: A novel approach to evaluating individual strategies with stable isotopes. Frontiers in Zoology, 13: 24.

Sénéchal É, Bety J, Gilchrist HG, Hobson KA, Jamieson SE. 2011. Do purely capital layers exist among flying birds? Evidence of exogenous contribution to arctic-nesting common eider eggs. Oecologia 165:593-604.

29. LL. 143-144: Please note that although stopovers occur, evidence suggests that the spring migration of Baltic common eiders is highly synchronized and fast, leaving little time for prolonged staging (e.g., Alerstam et al. 1974).

Reference:

Alerstam T, Bauer C, Roos G. 1974: Spring migration of eiders *Somateria mollissima* in southern Scandinavia. Ibis 116: 194–210.

30. LL. 147-149: I was puzzled by the assumption that females with high reserves would reach their destination close to or in a lean body condition" and by its formalization in Appendix S1, where birds departing with maximal reserves are forced to arrive essentially lean via eq. S.1 (Sa) and fixed migration costs (400 g per leg to the breeding area) (Appendix S1).

For Baltic common eiders, this seems biologically unlikely for several reasons:

1. Spring migration is short, rapid and highly synchronized (Alerstam et al. 1974), and northward movements may often occur without stopovers. Thus, the need to consume large stores *en route* is limited.

2. Empirical data indicate that females depart Danish wintering grounds in good body condition. Laursen et al. (2019) report a mean adult female body mass of 2.23 kg in late winter (departure). These patterns are inconsistent with systematic arrival in a lean state.

3. Stable-isotope evidence from the northern Baltic shows that early-laying, heavy females predominantly finance yolk formation from stored (endogenous) reserves, shifting to more local income later in the season, as a part of their mixed capital-breeding strategy (Jaatinen et al. 2016). This requires arriving with significant amounts of stored reserves, particularly for early breeders.

Because the model forces birds that depart with large reserves to arrive nearly lean, it likely underestimates early-season capital use and could skew your thiamine and fitness projections. Can you comment, please?

References:
Alerstam T, Bauer C, Roos G. 1974: Spring migration of eiders *Somateria mollissima* in southern Scandinavia. Ibis 116: 194–210.
Jaatinen K, Öst M, Hobson KA. 2016. State-dependent capital and income breeding: A novel approach to evaluating individual strategies with stable isotopes. Frontiers in Zoology 13: 24.
Laursen K, Møller AP, Öst M. 2019. Body condition of eiders at Danish wintering grounds and at pre-breeding grounds in Åland. Journal of Ornithology 160: 239–248.

31. LL. 154-157: The conclusion that female eiders feed during incubation should be interpreted with some degree of caution. Rigou and Guillemette (2010) show that incubating females very occasionally dive for a few minutes per day, but their loggers cannot demonstrate prey ingestion. In contrast, Parker and Holm (1990) reported complete fasting during incubation in a high-Arctic population of eiders. As a sensitivity check, please consider re-running the model assuming no feeding occurs during incubation.

32. LL. 157-160: You may want to include some references demonstrating lower reproductive success with advancing laying date in eiders as well as, such as Descamps et al. (2011) and Jaatinen et al. (2013).

References:

Descamps S, Bêty J., Love OP, Gilchrist HG. 2011. Individual optimization of reproduction in a long-lived migratory bird: A test of the condition-dependent model of laying date and clutch size: Optimal reproduction in a long-lived bird. *Functional Ecology* 25: 671–681.
Jaatinen K, Seltmann MW, Hollmén T, Atkinson S, Mashburn K, Öst M. 2013. Context dependency of baseline glucocorticoids as indicators of individual quality in a capital breeder. General and Comparative Endocrinology 191: 231–238.

33. LL. 168-170: The assumption that female eiders with extensive body reserves have lower survival appears at odds with the available empirical evidence. First, Guillemette and Ouellet (2005) document a mechanistic risk—temporary flightlessness in still air during late pre-laying—and they themselves caution against directly inferring survival costs from impaired take-off alone. Furthermore, the mass increase they quantify is largely due to ovarian growth while somatic mass remains roughly constant, so 'extensive reserves' per se may not show the assumed relationship with survival. Indeed, longitudinal capture-mark-recapture data from Baltic eiders show that female apparent survival increases with body condition measured at the first known breeding event (variable chosen to minimize age effects), with habitat type included as a covariate. There are several, not mutually explanations why we should expect a positive association between body condition and survival, including the asset protection principle (e.g., Clark 1994) or state-dependent safety (e.g., Luttbeg and Sih 2010).

In light of these uncertainties, I encourage the authors to add a sensitivity analysis showing whether the key conclusions are robust when the body reserve-survival link is either removed, or reversed to a small *positive* condition effect outside the brief pre-laying window, consistent with the empirical patterns reported in Ekroos et al. (2012).

References:

Clark CW. 1994. Antipredator behavior and the asset-protection principle. Behavioral Ecology 5: 159–170.
Luttbeg B, Sih A. 2010. Risk, resources and state-dependent adaptive behavioural syndromes. Philosophical Transactions of the Royal Society B: Biological Sciences 365: 3977–3990.

34. LL. 184-187: Here, you envision a cost of reproduction trade-off. This is a strong claim in need of supporting evidence. Please either (i) cite empirical or theoretical work showing micronutrient (ideally thiamine or close analogs) retention by mothers at the expense of eggs in birds, or (ii) rephrase to avoid implying a general rule or active maternal restraint. Also note that elsewhere you write that thiamine cannot be long-term stored (liver half-life ca 2.5 weeks, LL. 132-133), which seems at odds with the hypothesized maternal 'retention' of these micronutrients. Consider instead framing the assumption neutrally (e.g., egg thiamine reflects maternal status at laying).

35. LL. 194-196: This wording reads as if prey-size preference was estimated from your data. However, neither the Methods nor Appendix S1 present empirical estimates of prey-size selection by eiders at the wintering, stopover, or breeding sites. The mussel work provides

thiamine-mussel size relationships from lab-processed, size-stratified samples, with pooling of small individuals and subsampling of large ones to achieve target tissue masses. This design ensures coverage of the full size range, but does not represent ambient benthic size distributions of mussels, nor does it include diet or foraging data from eiders (e.g., gizzard contents).

In the main text you also frame prey-size differences as scenario comparisons rather than quantities estimated from field data (LL. 133-135), and Fig. 1D shows the assumed daily intake distribution, rather than empirical preference.

To remedy the situation, I suggest clarifying that prey-size ‘preference’ is an assumed model parameter explored in scenarios (with the specific size range you use), not something derived from your sampling.

36. L. 211: Please define the acronym RMR; it is not common knowledge to all readers.

37. LL. 219-222: Please note that a 1 cm shift is big relative to what female eiders actually eat, particularly in the central and northern parts of the Baltic Sea. Thus, in the western Gulf of Finland, females typically ingest mussels in the size range of 17-18 mm, which is below the mean mussel size at the bottom (21 mm). A 10 mm change would move diets beyond the central, observed diet size range. To increase realism, please consider using smaller steps in your scenarios (e.g., 2–5 mm steps), and/or clarify that 1 cm increments reflect an analytical unit rather than a typical behavioural shift in this study system.

38. LL. 239-244: The assumptions underlying these results are problematic for several reasons, as they seem inconsistent with the current reality in the Baltic Sea and conflict with well-established phenology-fecundity patterns.

First, under the current predation regime, rapidly intensifying predation (notably by white-tailed eagles) is associated with later breeding, a longer arrival-to-laying interval, reduced breeding synchrony, and smaller clutches (Öst et al. 2022); these are classic fear-effects of increased predation danger, which delay breeding despite the benefits of early hatching and favour smaller clutches.

Second, in eiders (Descamps et al. 2011, Lehikoinen et al. 2006, Öst et al. 2022) and birds in general (e.g., Verhulst and Nilsson 2008), earlier laying is linked to larger clutches (clutch size typically declines with laying date), so the hypothesized causality (earlier onset leads to smaller clutches) runs counter to the theoretical and empirical evidence.

I suggest clarifying the scope of this Result, as it may be most likely to hold under low predation risk settings. Furthermore, to reconcile the model with observed long-term trends in the Baltic Sea, you may consider exploring scenarios which allow adult/nest predation risk to vary, and to model clutch size as condition- and timing-dependent, assuming that better body condition and earlier laying is associated with larger clutches.

References:
Descamps S, Bêty J., Love OP, Gilchrist HG. 2011. Individual optimization of reproduction in a long-lived migratory bird: A test of the condition-dependent model of laying date and clutch size: Optimal reproduction in a long-lived bird. Functional Ecology 25: 671–681.
Lehikoinen A, Kilpi M, Öst M. 2006. Winter climate affects subsequent breeding success of common eiders: Global Change Biology 12: 1355–1365.
Öst M, Lehikoinen A, Jaatinen K. 2022. Top‑down effects override climate forcing on reproductive success in a declining sea duck. Oikos 2022:e08762.
Verhulst S, Nilsson J-Å. 2008. The timing of birds' breeding seasons: A review of experiments that manipulated timing of breeding. *Philosophical Transactions of the Royal Society B: Biological Sciences* 363: 399–410.

39. LL. 252-255: This is an overstatement considering what the results actually indicate. Your study combines measured mussel size-thiamine content relationships with a modelled life-history/physiology framework and then compares assumed prey-size preference scenarios; it does not measure foraging time, energetic trade-offs, or prey-size selection in the field, which are key elements of optimal foraging theory. I suggest softening this claim to reflect that the study complements energy-focused optimal foraging theory by highlighting a micronutrient constraint rather than claiming to go "far beyond" it.

40. LL. 255-257: Again, this overstates what the study shows. The manuscript documents a negative thiamine content-size relationship in mussels and then explores assumed prey-size scenarios within a life-history model with assumed daily intake; it does not quantify foraging costs or empirical prey selection, so an "unavoidable trade-off" is not demonstrated here. Please soften the statement.

41. LL. 257-266: One of the key findings is the negative relationship between mussel size and thiamine concentration, which is interpreted as a consequence of decreasing mass-specific metabolic rates in larger individuals. While this explanation is plausible, I would like to offer an alternative, potentially complementary hypothesis: the observed pattern may instead reflect size-dependent reproductive status at the time of sampling.

The rationale of this hypothesis is the following: In Baltic Sea blue mussel populations, individuals typically reach sexual maturity between 15–25 mm in shell length, depending on salinity and environmental conditions (Kautsky 1982). Thus, smaller mussels (< 2.5 cm) sampled in June likely include many immature individuals that had not yet initiated gametogenesis, whereas larger mussels were presumably at various stages of reproduction or post-spawning depletion. Given that thiamine levels are known to decline during reproductive investment (e.g., Seguineau et al. 2001ab), the mussel size–thiamine content gradient may reflect physiological shifts linked to maturity rather than only metabolic scaling.

Importantly, this has broader implications: while the metabolic-rate explanation implies a general, year-round disadvantage of consuming large mussels from a thiamine deficiency perspective, the reproductive-status mechanism suggests that such disadvantages are temporary and seasonal, peaking around the reproductive period. This distinction may limit

the temporal generality of the observed size-thiamine pattern and should be discussed explicitly. In this context it is pertinent that all sampling of blue mussels in the manuscript were conducted during a narrow time window in June, coinciding with the peak phase of reproduction in mature Baltic blue mussels.

References:

Kautsky N. 1982. Growth and size structure in a Baltic *Mytilus edulis* population. Marine Biology 68: 117–133.
Seguineau C, Saout C, Paulet YM, Muzellec ML, Quéré C, Moal J, Samain J-F. 2001a. Changes in tissue concentrations of the vitamins B1 and B2 during reproductive cycle of bivalves: Part 1: The scallop *Pecten maximus*. Aquaculture 196: 125–137.
Seguineau C, Migaud H, Quéré C, Moal J, Samain J-F. 2001b. Changes in tissue concentrations of the vitamins B1 and B2 during reproductive cycle of bivalves: Part 2: The Pacific oyster *Crassostrea gigas.* Aquaculture 196: 139–150.

42. LL. 267-277: This paragraph is speculative and needs to be either removed or entirely rewritten. The hypothesis presented here implies that females may select small mussels for their thiamine content. However, to the best of my knowledge, there is no empirical evidence that eiders (or vertebrate consumers generally) can assess prey vitamin content during feeding, and none is presented here. By contrast, there is strong, long-standing evidence that eider foraging is constrained by shell processing and digestion: eiders swallow prey whole, ingest large masses of inorganic shell each day, and the gizzard grinding-defecation process is a major, time-consuming component of the feeding cycle, with resting bouts devoted to shell crushing (e.g., Guillemette et al. 1992, Hario and Öst 2002). These digestive constraints, not micronutrient sensing, are the established mechanism behind selection for smaller mussels and minimization of shell mass.

A further argument pointing to the improbability of this hypothesis is that the text reads as if micronutrient-driven evolutionary responses might already shape prey-size selection. Given the (presumably) short and uncertain time window over which thiamine deficiency may have been relevant in the Baltic Sea, such adaptation is speculative without evidence. I recommend removing or softening any implication of rapid evolutionary change and framing your results as proximate, testable predictions about nutritional constraints that might influence behaviour, a possibility rather than a demonstrated evolutionary response.

References:
Guillemette M, Ydenberg RC, Himmelman JH. 1992. The role of energy intake rate in prey and habitat selection of common eiders *Somateria mollissima* in winter: a risk-sensitive interpretation. Journal of Animal Ecology 61: 599–610.
Hario M, Öst M. 2002. Does heavy investment in foraging implicate low food acquisition for female common eiders *Somateria mollissima*? Ornis Fennica 79: 111-120.

43. LL. 278-289: This paragraph assumes a general preference of large prey, but this is not demonstrated for your focal system and may, in fact, be contradicted by empirical evidence

from the Baltic Sea and elsewhere. For example, Öst and Kilpi (1998) found no unambiguous size-selective feeding and note that, if anything, there may have been a preference for consuming mussels slightly smaller than the average found on mussel beds. Besides, your study does not measure preference (no paired benthos-diet comparisons), yet the Discussion repeatedly infers a female preference for large blue mussels.

Furthermore, Laursen and Møller (2021), central to your argument, correlate maximum sizes of mussels in gizzards with head volume at Danish wintering sites, but lacks concurrent benthic size distributions and acknowledges possible overestimation of large items; the experimental work they cite shows selection for 10-20 mm mussels due to their higher flesh to shell ratios (Bustnes 1998). Given this, the extrapolation that a widespread "large-prey preference" would drive micronutrient limitation is speculative. Please either provide direct evidence (paired benthos-diet data demonstrating larger-than-available prey) or reframe this section as a hypothesis, and temper claims about generality across consumers, especially for eiders that ingest whole prey and are strongly constrained by shell-processing and digestion rather than energy intake per se (see my previous comment 42 above).

References:
Bustnes JO. 1998. Size selection of blue mussels *Mytilus edulis* by common eiders *Somateria mollissima* in relation to shell content. Can J Zool 76:1787-1790.
Laursen K, Møller AP. 2021. Brain mass explains prey size selection better than beak, gizzard and body size in a benthivorous duck species. PLoS ONE 16:e0248615.
Öst M, Kilpi M. 1998. Blue mussels *Mytilus edulis* in the Baltic : good news for foraging eiders *Somateria mollissima*. Wildl Biol. 4: 81-89.

44. LL. 290-297: I was unable to find a statement in the manuscript that defines a functional yolk-thiamine threshold for detrimental effects. However, in the previous literature, a concentration of ca 5 nmol g^{-1} has been mentioned. If you intend to invoke a biological threshold, please cite it explicitly and justify its species- and context-specific relevance.

45. LL. 298-299. Please consult my comments 21 and 23 (see above) for a motivation why the claim that females successful at nesting prefer large prey is unwarranted.

46. LL. 299-304: The "order-of-magnitude" comparison appears misleading, at least for the central/northern parts of the Baltic Sea. Local diet reconstructions show weak size selection and a narrow prey-size distribution by female eiders (e.g., faeces 18.2 ± 1.8 mm; intestines 17.4 ± 1.1 mm; matched benthos sample 19.1 ± 4.3 mm; no significant diet-benthos difference) (Öst and Kilpi 1998), and the authors conclude that no unambiguous size-selective feeding occurs in this region. Please either justify this claim with data or rephrase to avoid implying that a 1 mm shift is negligible in this context.

47. LL. 304-308: The cited studies (Christie et al. 2020, Baden et al. 2021) concern the Skagerrak-eastern North Sea area and emphasize predation-driven declines in blue mussels; these findings may not be directly applicable to blue mussel dynamics in the Baltic Sea.

Please either provide Baltic-specific evidence that low mussel recruitment leads to a local over-representation of large mussels in eider diets or remove this sentence.

48. LL. 308-311: This phrasing is confusing: it implies lipid-rich prey items have high thiaminase. Please separate and explain the two distinct mechanisms: (i) thiaminase in prey degrades thiamine before absorption; (ii) high-lipid diets increase thiamine demand and oxidative degradation.

49. LL. 316-319: This sentence overstates both the causal certainty and the taxonomic/geographic scope of the alleged thiamine-deficiency-induced mass mortality, and hence needs to be rephrased.

Thus, of the four citations, three concern salmonids/fish (Bengtsson 1999, Fitzsimons 1999, Mantua 2021) and therefore do not establish that offspring mortality is generally caused by thiamine deficiency across vertebrates. Only Mörner et al. 2017 addresses eiders, and that report describes a single field episode outside the Baltic proper (Swedish west coast), where reduced/failed duckling production could also plausibly arise via other, more general ecological mechanisms (e.g., predation, disease). Furthermore, the wording "mortality episodes in offspring" invites readers to infer that such episodes are established and common in birds, whereas the cited fish literature is not transferable evidence for eider ducklings or other birds alike. Within your own manuscript you also emphasize that predation is a predominant driver of poor recruitment in Baltic eiders, which directly conflicts with the categorical phrasing here.

50. LL. 320-322: This statement is not supported by the cited literature for eiders. In Balk et al. (2009), the explicit clutch-size analysis and its correlation with yolk thiamine were shown for herring gulls, whereas the eider evidence concerns low yolk thiamine and thiamine-deficiency biomarkers; there is no eider-specific test linking thiamine status to clutch size. Further, Mörner et al. (2017) report eider thiamine deficiency and long-term nest counts but do not present an analysis of eider clutch size versus thiamine, nor do they exclude alternative mechanisms, most notably partial or complete clutch predation, that can reduce the observed number of eggs (e.g., Öst et al. 2008). Accordingly, please either (i) provide eider-specific empirical evidence demonstrating that thiamine deficiency reduces clutch size independent of predation and other ecological processes, or (ii) rephrase to avoid implying such an association in eiders.
Reference:
Öst M, Wickman M, Matulionis E, Steele B. 2008. Habitat-specific clutch size and cost of incubation in eiders reconsidered. Oecologia 158:205–216.

51. LL. 322-323: Please note that the logic underlying the life-history model is inverted (earlier breeding phenology is expected to be associated with an earlier, not later, breeding phenology; see my comment 38 above).

52. L. 326: However, please note that the use of stopover sites may be very limited in spring-migrating eiders in the Baltic (see my comments 29 and 30 above).

53. LL. 326-329: This sentence is an overstatement of a syndrome that not been confirmed with certainty in eiders, and it further assumes a hypothesized adaptive response by females, which is equally unconfirmed.

First, "early mortality syndrome" (EMS) is a term established for salmonids in the fish literature (e.g., Fitzsimons 1999) and is not an established syndrome in eiders. Please avoid implying that an EMS exists in eiders or that such "years" are documented for the Baltic population. Please use taxon-appropriate terminology and restrict claims to cases demonstrated in the cited taxon.

Second, the manuscript's results show that a ~40% reduction in clutch size in the model can elevate egg thiamine by up to ~20%, but the model does not link thiamine to duckling survival or recruitment, and it does not incorporate alternative mechanisms (e.g.. partial/complete clutch predation, disease) that can reduce observed clutch size. Accordingly, the inference that clutch reduction is an adaptive response that "increases average recruitment" is not supported by the current evidence or model design and should be softened.

54. LL. 331: This statement is not entirely correct. In fact, several studies report that female eiders continue feeding intensively until the second or third egg is laid, after which nearly permanent presence at the nest begins (e.g., Hanssen et al. 2002; Andersson and Waldeck 2006).

References:

Andersson M, Waldeck P. 2006. Reproductive tactics under severe egg predation: an eider's dilemma. Oecologia 148:350–355.

Hanssen SA, Engebretsen H, Erikstad KE. 2002. Incubation start and egg size in relation to body reserves in the common eider. Behav Ecol Sociobiol 52:282–288.

55. LL. 334-336: This sentence makes several unsupported inferences. First, the manuscript does not provide empirical evidence that eider females detect short-term changes in maternal thiamine and plastically reduce clutch size; the results show only that, in the model, fewer eggs could raise per-egg thiamine contents. Second, the proposed "rapid" response overlooks the follicle-recruitment schedule in birds, which constrains seasonal clutch size adjustment and would require data on intra-season thiamine variability and a demonstrated decision pathway**.** Third, "offspring production" conflates eggs laid with post-laying outcomes; a smaller observed clutch size can arise from partial/complete clutch predation or other causes, and do not necessarily imply maternal adjustment. Finally, the cross-taxon contrast with salmon implies a general rule without comparative evidence on timing, plasticity, or fitness consequences.

56. LL. 341-343: The broader claim that “size-specific reduction in concentration is an important factor in the dynamics of essential metabolic micronutrients in consumers” generalizes beyond what was analyzed (one micronutrient, one prey taxon, one consumer modelled). Please moderate this concluding sentence.

I hope you will find these comments constructive and helpful for improving the manuscript!
