# Supplementary material for: The risk of thiamine deficiency in common eider ducklings increases when females consume large prey
Source: PLoS One. 2026 Sep 16;21(9):e0356777. doi: 10.1371/journal.pone.0356777 (PMC13580947; doi:10.1371/journal.pone.0356777)
Supplement: Supplementary file 4 [file pone.0356777.s004.pdf]

Review of PONE-D-25-50326. Selection for large prey increases risk of mass juvenile mortality due to thiamine deficiency

General comments

This is a very interesting manuscript. I was not aware of the severity of the thiamine deficiency issue or the reports of mass mortality of common eiders in the Baltic population. This paper provides an important new contribution by assessing how thiamine deficiencies could arise through prey size selection of blue mussels by common eiders. The authors show that there is a strong pattern of thiamine tissue concentration with respect to mussel size and this could have important ramifications not only for diet selection, but for thiamine deficiencies in eggs and ducklings that could increase juvenile mortality and contribute to the long-term decline of these populations.

The paper is based on analysis of thiamine tissue concentrations from a collection of mussels from the wintering, stopover, and breeding grounds, as well as a comprehensive modeling analysis using dynamic programming to derive optimal life histories and then using that to predict how prey size selection could impact survival and reproductive success in common eiders. I thought this was a novel approach and while it is based on predictive modeling and assumes that eiders do indeed follow optimal life histories (what if they don't?), it clearly illustrates how diet selection could significantly impact juvenile mortality, and might have the greatest effect on larger females that would be otherwise expected to reproduce well.

I have only a few comments/suggestions. I was a bit confused because the paper describes large eiders as selecting larger mussels (lines 85-93), which makes sense (perhaps based on brain size), and one would expect that larger mussels provide a greater energetic value overall. Hence, the concern is that if selection for larger mussels reduces the amount of thiamine that females acquire and provide to their eggs, this could have negative consequences. However, later in the manuscript, the authors note that in experimental studies, common eiders typically select the smallest class of mussels (line2 70-271). So, I am a bit unclear about which direction is expect for prey size selection – larger or smaller? I could use a bit more clarification.

Following on this point, I can see how there might be selection for large mussel size based purely on energy content, and larger females (with larger brains) might be better able to capture the larger mussels. And I can also see how that, in turn, could directly impact clutch size simply because of the greater energy and nutrient content available for making more eggs. The energetic pathway is clear and well established.

It's less clear to me how eiders would directly select for thiamine content (i.e. selecting mussel sizes for their thiamine content), and how that would directly or physiologically affect clutch size. Is there a direct physiological link between thiamine concentration and the production of eggs? Could it simply be that eiders that do select for small mussels are less able (energetically) to produce as many eggs (and so clutch sizes are smaller), but the unintended (and perhaps unselected for) consequence is that thiamine concentration is actually higher and so those birds end up doing better in terms of egg/juvenile survival. A sort of hidden physiological reversal of fortunes –> smaller prey size (but greater thiamine concentration) -> less energy -> smaller clutch sizes -> greater juvenile survival (due to higher thiamine concentration). The opposite would obtain for larger females selecting larger prey. I understand that this is what the authors are describing. My point was simply that I found it hard to imagine how eiders might be actively adjusting selection of prey sizes based on thiamine concentration – what is the mechanism by how they would assess that or how that would directly influence the production of eggs. OK – just some musings.

I wondered what the outcome of the model would be in females did not follow the optimal life history derived from the dynamic state-variable model? Might thiamine deficiency also influence the timing of breeding or seasonal resource allocation by females such the impacts are even greater or different? Perhaps a comment on this in the discussion. It might also be useful to consider any other uncertainties or assumptions of the model that might impact the outcomes (perhaps as avenues for future work).

Fig 1 shows the range of mussel sizes in each location, but it might also be useful to add a plot of the overall frequency distribution of mussel sizes in the samples collected. It appears from the scatterplots in Fig. 1B that most mussels were <3 cm and many <2cm. So, an eider simply foraging at random might choose mussels that are in the higher range of thiamine concentrations than had they selected mussels >3 cm (Figure 2C).

I am not a physiologist, nor do I know very much about thiamine accumulation, metabolism, reabsorption ,or excretion, and it would be worthwhile to have a physiologist evaluate the rates of thiamine intake and loss that were used in the model. However, to my uninformed eye, the analyses and assumptions made appear thorough, well-supported and thoughtful.

I have a few other smaller line-item comments below, but overall, this is a valuable and important contribution. I hope my comments are useful.

Specific comments

Lines 86-87. Is this just a function of larger brain size or overall larger body size as well? Did the Laursen and Moller 2021 paper control for body size?

Lines 113. Did each sample comprise mussels of varying sizes? Could you add another plot summarizing the frequency distribution of mussel sizes samples from each location (perhaps not necessary since this can be inferred from the scatterplots in Fig. 1B but a summary frequency distribution would be helpful.).Could differences in the availability of mussel size classes among locations or seasons also impact diet patterns and hence prey size selection and thiamine concentrations in the diet?

Lines 128-129. This is very reasonable but what if all or some females did not follow the optimal life history derived from the dynamic state-variable model? As noted above, might thiamine deficiency also influence the timing of breeding or resource allocation by females such the impacts of thiamine deficiency are even greater, or different?

Lines 221-224. Why the differences in the R^2 values among locations/season? Thoughts?

Lines 238. I wonder if a small table listing the parameter, their symbols and brief description could be included in the text. It would help a reader as one reads through the results on the effects of different parameter values and rate coefficients.

Lines line 270-277 and . This is where I got a bit hung up on whether eiders generally select for larger or smaller mussels. And if they do select for smaller mussels, how would they assess thiamine content and how would that directly impact the number of eggs they lay. I can see how females might select prey based on handling and search times and energetic content, and that would directly translate into the energy available to produce eggs clutch sizes. I have a less clear understanding how females could select for thiamine content other than as an incidental effect of selecting for small sizes (perhaps for other reasons) and how that physiologically might limit clutch size. Maybe a bit more discussion on this idea.

Line 377. The lines for the two alternative scenarios of offspring value (solid blue and dotted blue) in G=Fug. 1C are hard to see against the green background. Maybe just use black lines?
