# Supplementary material for: The risk of thiamine deficiency in common eider ducklings increases when females consume large prey
Source: PLoS One. 2026 Sep 16;21(9):e0356777. doi: 10.1371/journal.pone.0356777 (PMC13580947; doi:10.1371/journal.pone.0356777)
Supplement: Supplementary file 5 [file pone.0356777.s005.pdf]

Dear Editors of PLOS One,

Thank you for your thorough evaluation of our manuscript. We have now revised the manuscript based on the reviewers' suggestions and comments. Below, we explain in detail the changes we have made to address the issues raised during the review process (our responses follow the reviewers' comments). For your convenience, our responses are in dark blue and are accompanied by line numbers that refer to the appropriate locations in the manuscript with changes tracked.

Yours sincerely,

Maciej Ejsmond

On behalf of the authors

PONE-D-25-50326
Selection for large prey increases risk of mass juvenile mortality due to thiamine deficiency
PLOS ONE

Dear Dr. Ejsmond,

Thank you for submitting your manuscript to PLOS ONE. After careful consideration, we feel that it has merit but does not fully meet PLOS ONE's publication criteria as it currently stands. Therefore, we invite you to submit a revised version of the manuscript that addresses the points raised during the review process.

Please submit your revised manuscript by Jan 10 2026 11:59PM. If you will need more time than this to complete your revisions, please reply to this message or contact the journal office at plosone@plos.org. When you're ready to submit your revision, log on to https://www.editorialmanager.com/pone/ and select the 'Submissions Needing Revision' folder to locate your manuscript file.

Please include the following items when submitting your revised manuscript:

- A rebuttal letter that responds to each point raised by the academic editor and reviewer(s). You should upload this letter as a separate file labeled 'Response to Reviewers'.
- A marked-up copy of your manuscript that highlights changes made to the original version. You should upload this as a separate file labeled 'Revised Manuscript with Track Changes'.
- An unmarked version of your revised paper without tracked changes. You should upload this as a separate file labeled 'Manuscript'.

If you would like to make changes to your financial disclosure, please include your updated statement in your cover letter. Guidelines for resubmitting your figure files are available below the reviewer comments at the end of this letter.

If applicable, we recommend that you deposit your laboratory protocols in protocols.io to enhance the reproducibility of your results. Protocols.io assigns your protocol its own identifier (DOI) so that it can be cited independently in the future. For instructions see: https://journals.plos.org/plosone/s/submission-guidelines#loc-laboratory-protocols. Additionally, PLOS ONE offers an option for publishing peer-reviewed Lab Protocol articles, which describe protocols hosted on protocols.io. Read more information on sharing protocols at

https://plos.org/protocols?utm_medium=editorial-email&utm_source=authorletters&utm_campaign=protocols.

We look forward to receiving your revised manuscript.
Kind regards,
Juan J Loor
Academic Editor
PLOS ONE

Journal requirements:

When submitting your revision, we need you to address these additional requirements.

1. Please ensure that your manuscript meets PLOS ONE's style requirements, including those for file naming. The PLOS ONE style templates can be found at

https://journals.plos.org/plosone/s/file?id=wjVg/PLOSOne_formatting_sample_main_body.pdf and

https://journals.plos.org/plosone/s/file?id=ba62/PLOSOne_formatting_sample_title_authors_affiliations.pdf

Response #1: Due to the length of the review reports obtained and the time required to respond to the comments of the reviewers (which comprise more than 110 compounded questions and issues), we have decided to make the final formatting changes to the text after the review process is complete. Given the detailed feedback from the reviewers, we hope that introducing the formatting changes later in the review process will make the text with highlighted changes easier to follow.

2. Thank you for stating the following financial disclosure:

"European Union's Horizon Europe research and innovation programme under the Marie Skłodowska-Curie grant agreement (101126636) to ME.

Carl Trygger Foundation, The Swedish research council VR (grant no.: 2019-04251), and the Swedish research council FORMAS (FR-2020/0008) to SH and MH.

University of Iceland Recruitment Fund, grant no. 2900-293501 to AE.

The project MARBEFES of the European Union's Horizon Europe Programme (Grant no. 101060937,project MARBEFES, www. marbefes.eu) to AW.

Wroclaw Centre for Networking and Supercomputing (http://wcss.pl), grant No. 576 to ME."

Please state what role the funders took in the study. If the funders had no role, please state: "The funders had no role in study design, data collection and analysis, decision to publish, or preparation of the manuscript."

If this statement is not correct you must amend it as needed.

Please include this amended Role of Funder statement in your cover letter; we will change the online submission form on your behalf.

Response #2: We now state in the Cover Letter that 'the funders had no role in study design, data collection and analysis, decision to publish, or preparation of the manuscript'.

3. Please update your submission to use the PLOS LaTeX template. The template and more information on our requirements for LaTeX submissions can be found at http://journals.plos.org/plosone/s/latex.

Response #3: As far as we understand, LaTeX format is optional. We prefer to submit using the docx format.

4. We note that Figure 1A in your submission contain copyrighted images. All PLOS content is published under the Creative Commons Attribution License (CC BY 4.0), which means that the manuscript, images, and Supporting Information files will be freely available online, and any third party is permitted to access, download, copy, distribute, and use these materials in any way, even commercially, with proper attribution. For more information, see our copyright guidelines: http://journals.plos.org/plosone/s/licenses-and-copyright.

We require you to either (1) present written permission from the copyright holder to publish these figures specifically under the CC BY 4.0 license, or (2) remove the figures from your submission:

1. You may seek permission from the original copyright holder of Figure 1A to publish the content specifically under the CC BY 4.0 license.

We recommend that you contact the original copyright holder with the Content Permission Form (http://journals.plos.org/plosone/s/file?id=7c09/content-permission-form.pdf) and the following text:

"I request permission for the open-access journal PLOS ONE to publish XXX under the Creative Commons Attribution License (CCAL) CC BY 4.0 (http://creativecommons.org/licenses/by/4.0/). Please be aware that this license allows unrestricted use and distribution, even commercially, by third parties. Please reply and provide explicit written permission to publish XXX under a CC BY license and complete the attached form."

Please upload the completed Content Permission Form or other proof of granted permissions as an "Other" file with your submission.

In the figure caption of the copyrighted figure, please include the following text: "Reprinted from [ref] under a CC BY license, with permission from [name of publisher], original copyright [original copyright year]."

2. If you are unable to obtain permission from the original copyright holder to publish these figures under the CC BY 4.0 license or if the copyright holder's requirements are incompatible with the CC BY 4.0 license, please either i) remove the figure or ii) supply a replacement figure that complies with the CC BY 4.0 license. Please check copyright information on all replacement figures and update the figure caption with source information. If applicable, please specify in the figure caption text when a figure is similar but not identical to the original image and is therefore for illustrative purposes only.

Response #4: Figure 1A was created by Marc M. Hauber, one of the authors of this paper. This is now stated in the figure caption: 'Illustration by Marc M. Hauber' (L493). As he is one of the manuscript's co-authors, he has agreed to the article being licensed under CC BY 4.0.

5. We note that Figure 1B in your submission contain [map/satellite] images which may be copyrighted. All PLOS content is published under the Creative Commons Attribution License (CC BY 4.0), which means that the manuscript, images, and Supporting Information files will be freely available online, and any third party is permitted to access, download, copy, distribute, and use these materials in any way, even commercially, with proper attribution. For these reasons, we cannot publish previously copyrighted maps or satellite images created using proprietary data, such as Google software (Google Maps, Street View, and Earth). For more information, see our copyright guidelines: http://journals.plos.org/plosone/s/licenses-and-copyright.

We require you to either (1) present written permission from the copyright holder to publish these figures specifically under the CC BY 4.0 license, or (2) remove the figures from your submission:

1. You may seek permission from the original copyright holder of Figure 1B to publish the content specifically under the CC BY 4.0 license.

We recommend that you contact the original copyright holder with the Content Permission Form (http://journals.plos.org/plosone/s/file?id=7c09/content-permission-form.pdf) and the following text:

“I request permission for the open-access journal PLOS ONE to publish XXX under the Creative Commons Attribution License (CCAL) CC BY 4.0 (http://creativecommons.org/licenses/by/4.0/). Please be aware that this license allows unrestricted use and distribution, even commercially, by third parties. Please reply and provide explicit written permission to publish XXX under a CC BY license and complete the attached form.”

Please upload the completed Content Permission Form or other proof of granted permissions as an "Other" file with your submission.

In the figure caption of the copyrighted figure, please include the following text: “Reprinted from [ref] under a CC BY license, with permission from [name of publisher], original copyright [original copyright year].”

2. If you are unable to obtain permission from the original copyright holder to publish these figures under the CC BY 4.0 license or if the copyright holder’s requirements are incompatible with the CC BY 4.0 license, please either i) remove the figure or ii) supply a replacement figure that complies with the CC BY 4.0 license. Please check copyright information on all replacement figures and update the figure caption with source information. If applicable, please specify in the figure caption text when a figure is similar but not identical to the original image and is therefore for illustrative purposes only.

The following resources for replacing copyrighted map figures may be helpful:

USGS National Map Viewer (public domain): http://viewer.nationalmap.gov/viewer/

The Gateway to Astronaut Photography of Earth (public domain): http://eol.jsc.nasa.gov/sseop/clickmap/

Maps at the CIA (public domain): https://www.cia.gov/library/publications/the-world-factbook/index.html and https://www.cia.gov/library/publications/cia-maps-publications/index.html

NASA Earth Observatory (public domain): http://earthobservatory.nasa.gov/

Landsat: http://landsat.visibleearth.nasa.gov/

USGS EROS (Earth Resources Observatory and Science (EROS) Center) (public domain): http://eros.usgs.gov/#

Natural Earth (public domain): http://www.naturalearthdata.com/

Response #5: We replaced the background map in Figure 1B with one downloaded from Wikipedia, which was created using data from the Natural Earth repository. The map's source has now been cited in the manuscript (L495-497).

6. Please include captions for your Supporting Information files at the end of your manuscript, and update any in-text citations to match accordingly. Please see our Supporting Information guidelines for more information: http://journals.plos.org/plosone/s/supporting-information.

If the reviewer comments include a recommendation to cite specific previously published works, please review and evaluate these publications to determine whether they are relevant and should be cited. There is no requirement to cite these works unless the editor has indicated otherwise.

Response #5b: Corrected according to the PLOS ONE guidelines.

[Note: HTML markup is below. Please do not edit.]

Reviewers' comments:

Reviewer's Responses to Questions

Comments to the Author

1. Is the manuscript technically sound, and do the data support the conclusions?

The manuscript must describe a technically sound piece of scientific research with data that supports the conclusions. Experiments must have been conducted rigorously, with appropriate controls, replication, and sample sizes. The conclusions must be drawn appropriately based on the data presented.

Reviewer #1: No

Reviewer #2: Yes

Reviewer #3: Yes

---

2. Has the statistical analysis been performed appropriately and rigorously?

Reviewer #1: N/A

Reviewer #2: No

Reviewer #3: Yes

---

3. Have the authors made all data underlying the findings in their manuscript fully available?

The PLOS Data policy requires authors to make all data underlying the findings described in their manuscript fully available without restriction, with rare exception (please refer to the Data Availability Statement in the manuscript PDF file). The data should be provided as part of the manuscript or its supporting information, or deposited to a public repository. For example, in addition to summary statistics, the data points behind means, medians and variance measures should be available. If there are restrictions on publicly sharing data—e.g. participant privacy or use of data from a third party—those must be specified.

Reviewer #1: Yes

Reviewer #2: Yes

Reviewer #3: Yes

---

4. Is the manuscript presented in an intelligible fashion and written in standard English?

PLOS ONE does not copyedit accepted manuscripts, so the language in submitted articles must be

clear, correct, and unambiguous. Any typographical or grammatical errors should be corrected at revision, so please note any specific errors here.

Reviewer #1: Yes

Reviewer #2: Yes

Reviewer #3: Yes

---

5. Review Comments to the Author

Please use the space provided to explain your answers to the questions above. You may also include additional comments for the author, including concerns about dual publication, research ethics, or publication ethics. (Please upload your review as an attachment if it exceeds 20,000 characters)

Reviewer #1: My Reviewer Comments to the Author are provided as a separate file, as they exceed the length limit (48 076 characters with spaces).

I have no concerns about dual publication, research ethics, or publication ethics.

Response #6: Thank you for the tremendous effort by the Reviewer #1 to improve our study. Given the volume of the review report by Reviewer #1, we would like to clarify the general scientific background and our views on some of the comments.

The decline of the Baltic population of common eiders has sparked intense scientific discussion with several hypotheses proposed. One of the hypotheses states that the recent decline in the population of common eiders is due to mass mortality of juveniles caused by thiamine deficiency (Balk et al., 2009; Balk et al., 2016; Mörner et al., 2017). However, the dominant hypothesis postulates that the population decline is due to increased predation on breeding females (e.g. Ramula et al. 2018; Tjørnløv et al. 2020). The background observations are: (i) a reduction in proportion of females in the population, and (ii) a dramatic reduction in recruitment to the first-year age class (e.g. Ekroos et al. 2012). Reviewer #1 is sceptical about the 'thiamine-deficiency driven mortality' hypothesis, as expressed in their comments, and supports the 'predation-driven decline' hypothesis. Note that we have acknowledged predation as the main cause of the decline in Baltic eiders (see L33-34, 73-79 of the previous version of the text) but we also emphasize this in the corrected version (L33-34, 83-85, 383-386, Response #13). However, we believe that the decrease in recruitment and observations of high mortality of common eider ducklings caused by thiamine deficiency (e.g. Mörner et al., 2017) justify considerations of thiamine-deficiency as a factor that can contribute to the negative population trends of Baltic eiders, along with the well documented predation effect.

Reviewer #1 questions the quality of the scientific literature supporting the idea that thiamine deficiency may lead to very high mortality of common eiders. As supporting evidence, we cite a research report (Svedäng et al., 2018), which Reviewer #1 considers a 'grey' literature, as well as three scientific papers (Balk et al., 2009; Balk et al., 2016; Mörner et al., 2017). We are aware that some work of L. Balk is considered controversial by certain groups of scientists but rejection of this work, as in the case of some comments by Reviewer #1, is unjustified. In our opinion, the work of Balk provides at least partial support that episodic mass mortality events in marine birds have been caused by thiamine deficiency (Balk et al., 2009; Balk et al., 2016). Please note, that results by Balk have been widely cited and have not been scientifically falsified to our knowledge. We also cite work by Mörner et al. (2017), who documented thiamine deficiency and experimentally estimated that lack of thiamine causes a sevenfold increase in mortality of common eider ducklings. Moreover, thiamine deficiency-driven mass mortality has been observed in many other top marine consumers, with evidence emerging from several distinct geographical regions, including the Baltic Sea, the Great

Lakes and the West Coast of North America (see citations in Hylander et al. 2024 and the recently published study in PNAS Mantua et al. 2025, https://doi.org/10.1073/pnas.2426011122). Overall, we disagree with the suggestion that the scientific literature we cite is flawed. We are not advocating for the thiamine-deficiency hypothesis but argue that it is sound scientific practice to consider it along with other data. Therefore, we did not follow fully the reviewer's guidelines that questioned the role of thiamine deficiency as a potential contributing driver of high mortality of juvenile marine top consumers and common eiders. However, we did not reject suggestions of Reviewers #1 completely and have corrected several places in our manuscript to attain a more balanced rhetoric, falling between the sceptics and supporters of the 'thiamine-deficiency driven mortality' hypothesis.

Reviewer Comments to Authors

Manuscript ID: PONE-D-25-50236

Selection for large prey increases risk of mass juvenile mortality due to thiamine deficiency

by Maciej Jan Ejsmond, Anna Ejsmond, Marc M. Hauber, Jón Einar Jónsson, Andreas M. Waser & Samuel Hylander

This manuscript investigates how size selection of prey (blue mussels *Mytilus edulis trossulus*; hereafter mussels) may influence thiamine (vitamin B1) status in consumers (common eiders *Somateria mollissima*; hereafter eiders) and thereby potentially influence offspring survival, combining biochemical measurements of thiamine concentrations in Baltic blue mussels with a dynamic state-variable life-history model of thiamine dynamics in female eiders depending on breeding phenology and fecundity. Mussel assays across wintering, stopover, and breeding areas show that mass-specific thiamine content declines with increasing shell length; these measurements then parameterize scenarios that vary dietary bioavailability and renal excretion. Model results indicate that consumption of small rather than large mussels was associated with lower predicted egg thiamine, whereas producing a smaller clutch may partially elevate egg thiamine, and these patterns seemed robust to alternative assumptions about seasonal timing, mortality, and food intake. The broader implication is that choosing larger prey may trade energetic gains against essential micronutrient intake, suggesting that the mussel size distribution and size-dependent micronutrient content of prey may potentially shape reproductive output. A strength of the study is the careful analytical work on blue mussels and the linkage of the biochemical data to calculations in the modelling framework.

That being said, however, I have several serious concerns with the manuscript, and I suggest several potential improvements, as detailed below.

The following issues are of major concern:

1) Overstatement of the extent and severity of thiamine deficiency syndromes, particularly in birds, a predilection for tendentiousness, and a lack of defined thresholds for implying detrimental effects.

The manuscript's framing overreaches on scope and causality, beginning with an Abstract that asserts "episodic mass mortality… is a major threat to top marine consumers worldwide," and a title and keywords implying population-level risk—without bird- or eider-specific evidence to support such general claims. Much of the cited support comes from fish or grey literature and does not rule out dominant alternative drivers (e.g., predation), while the manuscript also lacks a clear, species-relevant yolk-thiamine threshold for inferring detrimental effects. The choice of language throughout (e.g., linking egg thiamine to nest predation, implying adaptive clutch-size responses to thiamine) invites causal inference where only associations are indicated and where key mechanisms remain untested. To remedy the situation, it would be important to temper the rhetoric, aligning claims to demonstrated

scope, and explicitly stating any biologically justified thresholds.

Response #7: There are several points raised by the Reviewer #1 in this comment:

(a) thiamine deficiency driven mass mortality of top marine consumers is not a factor that influences population trends in marine top consumers. **Response #7a:** See Responses #6, #22, #23.

(b) current literature that not support the link between thiamine deficiency, mass mortality, and negative populational trends in marine birds and common eider as the 'cited support' comes from grey or fish literature **Response #7b:** See Responses #6, #22.

(c) predation is a dominant and alternative driver of negative population trends in common eiders.

Response #7c: We acknowledged predation as the main cause of the decline in the population of Baltic common eiders in the previous submitted version of the manuscript (see L32-34, L73-79 in the previous version of our work) and in the corrected version (see Response #6, #13, #25).

(d) "choice of language … invites causal inference where only associations are indicated and where key mechanisms remain untested". **Response #7d:** We have softened the rhetorics in several places of our manuscript. Although, we believe that the reviewer's claim is based in particular on she/he questioning of the existing scientific literature supporting evidence for thiamine deficiency-driven mass mortality of marine birds and other top consumers (see Responses #6, #22, #23).

(e) a lack of clear, species-relevant yolk-thiamine threshold for inferring detrimental effects.

Response #7e: We now present the range of thiamine concentrations reported to be close to the threshold for detrimental physiological effects in common eiders (L266-272). Please note that the model predicts that maternal concentrations of thiamine and thiamine content in the eggs fall within the range of empirically reported concentrations. This demonstrates that our simplified model, which optimises the life history, breeding and migration phenology of the common eider in order to maximise fitness, is not flawed with respect to the parameterisation of thiamine consumption and excretion rates (see also Response #52, #67).

However, data on such concentration thresholds are scarce, and there are fundamental reasons why these thresholds could vary depending on body size or even physiological conditions. Overall, the factors affecting thiamine thresholds are not well understood (see Hylander et al., 2024). Therefore, we are sceptical about a more detailed discussion of our results in the context of thiamine thresholds associated with detrimental physiological effects. Firstly, we are aware of the controversies surrounding the discussion of the impact of thiamine deficiency on populations of top marine consumers, with sceptics and advocates adopting an 'all-or-nothing' thinking. Directly comparing much simplified scenarios of consumed prey size with scarce data on the thiamine threshold at which detrimental physiological symptoms occur would inevitably lead readers to draw bold and risky conclusions, which we believe are not justified by our study. Once again, we advocate for more balanced rhetoric, placing the scientific debate between the groups of sceptics and advocates of hypotheses on thiamine deficiency-induced mass mortality. Secondly, we wanted to focus on the qualitative link between consuming large prey and the subsequent decrease in thiamine concentration in offspring. We believe that any controversy surrounding the interpretation of the spectra of prey consumed with respect to thiamine threshold levels would distract the reader from our main qualitative conclusion.

2) Unconvincing leap from mussel thiamine to eider reproduction, including mismatches between the model and the theoretical and empirical evidence base.

While the mussel assays are careful, the step from size-dependent thiamine contents in prey to consequences for eider reproduction rests on assumptions and generalizations that are weakly supported or at odds with established theory and data. More specifically, demographic sensitivity in this long-lived species depends more on adult survival than fecundity; claims about large-brained females taking larger prey and thereby trading micronutrients are not robust; "preference" for prey sizes is assumed rather than estimated; key life-history settings (mixed capital breeding, migration/stopover use, feeding during early laying) are simplified; prey-size shifts are modelled at unrealistic increments; and the breeding phenology–clutch size relationship is inverted relative to

empirical patterns. Several strong statements (e.g., "unavoidable trade-off," generality beyond the study system) also outrun the analyses. Altogether, the model can illustrate potential mechanisms but does not convincingly demonstrate population-relevant reproductive effects without stronger empirical anchoring or sensitivity checks aligned with the empirical reality.

Response #8:

The reviewer mention six specific points;

(a) Questionable consequences of thiamine deficiency for population trends.

Response #8a: We agree that our work does not directly investigates the population consequences of the thiamine deficiency and we have corrected several parts of our work to include concerns raised by the Reviewer #1. However, due to a massive increase in mortality of ducklings due to thiamine-deficiency, and in the light of considerable drop in the recruitment of common eiders juveniles to 1st year age class, thiamine deficiency cannot be ruled out as a factor contributing to negative population trends of Baltic eiders (see Responses #10, #6, #13). However, along with the comment we have now moderated the language to encompass the uncertainties.

(b) claims about large-brained females taking large pray are not robust.

Response #8b: Following the suggestions of Reviewer #2 and the points raised by Reviewer #1 regarding the robustness of the findings by Laursen and Moller (2021), we have moved the discussion on 'large-brained females taking large prey' to the Discussion section (see Responses #14, #15, #70). Consequently, we no longer use elements of prey size preference related to brain size or the links between brain size and antipredator strategies of females as the main justification for our work (see Responses #14, #15, #70).

(c) key life history traits are simplified

Response #8c: It is true that our model simplifies certain aspects of the life history of common eiders. One of our objectives was to assess how robust the main model's predictions are in response to changes in seasonality and thiamine excretion rates (presented in S1 Appendix). To achieve this, it was necessary to simplify the model. Similarly, a simplified structure of the life history models allows analysis, as complex model structures hinder tractability. We now present additional simulations in the S1 Appendix, showing that the simplifications pointed out by Reviewer 1, did not affect the main conclusion of our work. Reviewer 1 pointed out:

- issues with the arrival of females on breeding grounds in a lean body condition, and the degree of capital breeding in Baltic eiders (Responses #33, #36)
- usage of stopovers during spring migration (Responses #37, #95)
- feeding during early laying period (Response #39)
- the breeding phenology–clutch size relationship is inverted relative to empirical patterns (see Responses #38, #46)
- rapidly intensifying predation (notably by white-tailed eagles) (see last paragraph in Response #46)

(d) "prey size shifts are modelled with unrealistic increments"

Response #8d: We disagree that the scenarios are modelled with unrealistic prey size increments. We have modelled scenarios that differed in average of consumed prey size by 5 mm, ranging from 1.5 to 3.5 cm. In the review book "The Common Eider" Waltho and Coulson (2015), report that common eiders in the Baltic Sea forage on mussels in range of 10-30mm, and for Wadden Sea (one of the main wintering population of common eiders, including Baltic eiders) the size range was reported as 20-40mm (Table 4.4 in Waltho and Coulson 2015). Reviewer #1 in the comment below (Point No. 46., Response #54) cite the work by Öst and Kilpi (1998) as the reference point for distribution of mussels consumed by eiders at a single location on the Finish coast of the Baltic Sea. Below we pasted the Figure 1 from Öst and Kilpi (1998) illustrating this distribution.

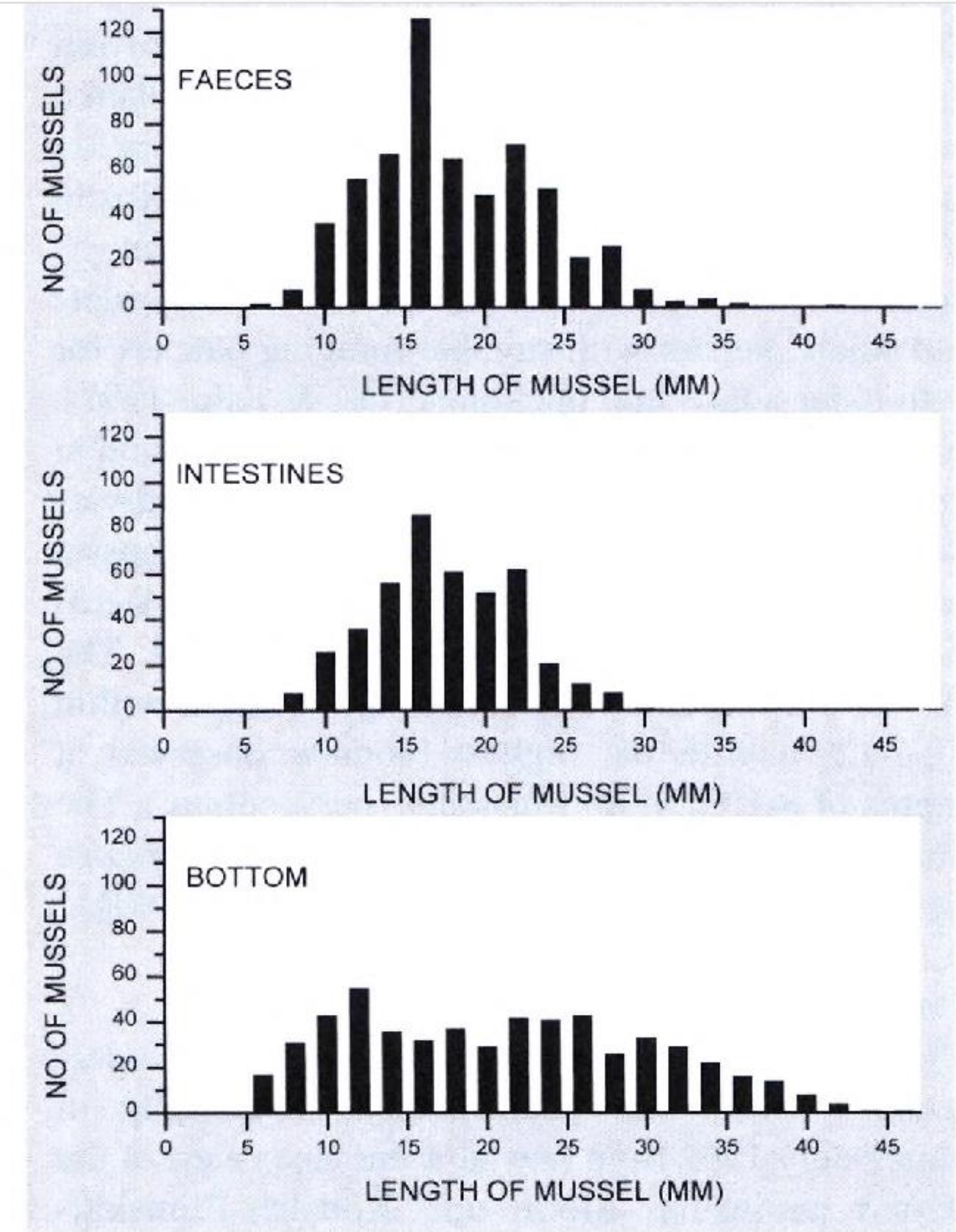

Figure 1. Size-frequency distribution of mussels from eider faeces (N = 20 eiders/600 mussels), intestines (N = 6 eiders/429 mussels) and from the bottom (N = 19 samples/560 mussels). The data is grouped in 2 mm size classes.

from Öst and Kilpi (1998)

Note that Reviewer #1 provided in their comment below (Point No. 46., Response #54) the mean size of blue mussels consumed: faeces 18.2 ± 1.8 mm; intestines 17.4 ± 1.1 mm; matched benthos sample 19.1 ± 4.3 mm. However, the deviations given match standard errors (standard deviation of sample means) (see Table 1 in Öst and Kilpi 1998) rather than characterize variance of the distribution of mussels consumed by common eiders in this particular location.

(e) breeding phenology-clutch size relationship is inverted to empirical patterns
Response #8e. We now present simulations that predict a decrease in clutch size with the onset of nesting, but the conclusions of our work remain unchanged (See Responses #38, #46).

(f) "several strong statements that outran analyses"; "Altogether, the model can illustrate potential mechanisms but does not convincingly demonstrate population-relevant reproductive effects without stronger empirical anchoring or sensitivity checks aligned with the empirical reality.".
Response #8f: We have corrected several places of our manuscript to attain a more balanced rhetoric, falling between the sceptics and supporters of the 'thiamine-deficiency driven mortality' hypothesis. For example, we have softened several statements in the Discussion section that were criticised by Reviewer #1 (see e.g. Response #48, #61, #57, #64). However, we feel that some of Reviewer #1's comments regarding the discussion imply that our statements should refer exclusively to the results of our study. Discussion sections in scientific papers are not typically framed in this way. We believe that

we are taking the standard approach by presenting our results in a broader context and proposing general implications based on our results and existing scientific literature. We also believe that comments by Reviewer #1 related to thiamine-deficiency driven mortality were biased by the fact they question the cited literature with scepticism towards the hypothesis of thiamine deficiency causing mass mortality in top consumers (see Responses #6, #22, #23).

3. Alternative explanations for the mussel size–thiamine pattern may limit temporal generality.

The observed decrease in thiamine with increasing mussel size is plausibly explained by metabolic scaling, but an equally plausible (and testable) alternative is size-dependent reproductive status at the time of sampling: smaller (often immature) mussels sampled in June may retain higher thiamine, whereas larger, mature individuals near/after spawning can be depleted. Accordingly, this may imply a seasonal, phenology-linked signal rather than a universal year-round size effect. If so, the inferred disadvantage of "large prey" for thiamine intake (and any cascading conclusions for eiders) would be temporally constrained; acknowledging this and, where possible, controlling for maturity/gonadal stage would sharpen inference and clarify scope.
Response #9: Thank you for your comment. We now discuss the alternative view suggested by Reviewer #1 in our manuscript (L423-429, See Response #49).

Here are my more specific comments, questions and suggested changes to the manuscript:

1. L. 1, Title: The title implies a causal, population-level link to "mass juvenile mortality," yet the study only models diet-size effects on egg thiamine; it does not measure or estimate juvenile mortality rates, survival probabilities, or population-level risk (e.g., the probability of mass-mortality events). The Abstract notes that the link between thiamine deficiency and predation risk is unclear (LL. 32-33). Your findings are system-specific model predictions; the most pertinent result is lower modelled egg thiamine if consuming larger mussels, not demonstrated mortality. The title should reflect the contents of the manuscript.
Here are two suggestions for more objective titles:
Consumption of larger blue mussels is associated with lower modelled egg-thiamine levels in Baltic common eiders
Feeding on larger blue mussels is associated with lower model-predicted egg-thiamine levels in Baltic common eiders
Response #10: Thank you for your constructive comment. We agree that the previous version of the title was incorrect, as our research does not focus on the population-level effects of lowered thiamine levels. We also acknowledge that this was an overstatement. We have adjusted the title to address the concerns raised by Reviewer #1. However, our model is only inspired by the life history of the Baltic population of common eiders, and the conclusions we have drawn can easily be extrapolated to other populations of common eiders and avian marine top consumers. Similarly, the combination of modelling and empirical analyses in our approach, as well as the reference to modelling in the title, may suggest that the work is purely theoretical. Hence, the new version of the title does not refer to the Baltic population or modelling.

2. Abstract, LL. 29-30: The first sentence of the Abstract is an overstatement. There is evidence for negative effects of thiamine deficiency in fish such as salmonids, while the link between thiamine levels and reproductive output in common eiders is weak at best. I will detail in my comments below why the supportive evidence is not very convincing.
Response #11: We disagree that the first sentence of the abstract is an overstatement, given the numerous examples of marine top consumers affected by episodic, thiamine deficiency-induced mass mortality and at least three scientific papers showing the problem concerns common eiders (see Response #6, #22).

3. Abstract, LL. 30-32: The sentence “Recent reports of thiamine deficiency…” needs to be omitted, as it is tendentious and speculative, for the reasons detailed in my comments below.
Response #12: We have changed ‘mass mortality’ to ‘high mortality rates’ and rewrote the sentence to use a more balanced rhetoric, falling between the sceptics and supporters of the 'thiamine-deficiency induced mass mortality' hypothesis (see Response #6). However, we disagree that the sentence is speculative. The high mortality rate of common eider ducklings due to thiamine deficiency is supported by scientific literature (see Response #6, #22, #23).

4. Abstract, LL. 32-34: First, it is unclear to me why we should expect egg thiamine status to *directly* raise nest predation. Are predators expected to detect nests with eggs having low thiamine levels?

Second, it is pertinent to note that in long-lived birds, population growth (λ) is most sensitive to adult survival rather than fecundity (e.g., Sæther and Bakke 2000). Consistent with this, several recent studies have indicated that the recent decline and the increasingly male-biased adult sex ratio are best explained by increased predation-induced mortality of breeding females rather than nest predation or fecundity variation alone (e.g., Öst et al. 2016; Ramula et al. 2018; Tjørnløv et al. 2020).
Can you please rephrase this sentence to address the above concerns?
References:
Öst M, Ramula S, Lindén A, Karell P, Kilpi M. 2016. Small-scale spatial and temporal variation in the demographic processes underlying the population decline of eiders in the Baltic Sea. Pop Ecol. 58: 121–133.
Ramula S, Öst M, Linden A, Karell P, Kilpi M. 2018. Increased male bias in eider ducks can be explained by sex-specific survival of prime-age breeders. PLoS One 13:e0195415.
Sæther BE and Bakke Ø. 2000. Avian life history variation and contribution of demographic traits to the population growth rate. Ecology 81: 642-653.
Tjørnløv RS, Ens BJ, Öst M, Jaatinen K, Karell P, Larsson R, Christensen TK, Frederiksen M. 2020. Drivers of spatiotemporal variation in survival in a flyway population: a multi-colony study. Frontiers in Ecology and Evolution 8: 566154.
Response #13: Thank you for the comment. Even in a long-lived bird species, where population growth (λ) is most sensitive to adult survival, a 60 percentage point drop in the proportion of first-year recruits in the total population (i.e. a drop from 80% to 20%; see Fig. 2a in Ekroos et al., 2012) will considerably affect the population growth rate due to the anticipated change in this population's age structure. This dramatic decrease in recruitment is particularly relevant in the case of Baltic eiders, given the accompanying reduction in overall population number. To avoid any misunderstandings, we have rewritten the sentence in the Abstract to refer directly to the dramatic decrease in the number of common eiders reaching their first year of life (L33-34). In light of the evidence showing a drastic increase in the mortality rate of Baltic eider ducklings due to thiamine deficiency (Morner et al. 2017), it is scientifically justified to question the potential role of episodic thiamine deficiency in the decline in recruitment rates. However, please note that we now acknowledge the leading role of predation in the recent negative population trends of Baltic eiders in both the abstract and the introduction (L33-34, 83-85, 383-386, Response #6).

5. Abstract, LL. 35-36: The evidence that eiders with larger brains feed on larger mussels is not backed up my compelling evidence, so the argument presented here is in need of revision. Please see my comments below for more details.
Response #14: We disagree with the reviewer's assertion that "The evidence that eiders with larger brains feed on larger mussels is not backed up my compelling evidence". Laursen and Møller (2021) in PLOS ONE paper showed that eiders with large head volume consumed larger prey with variation explained better by brain mass than beak, gizzard, and body size. We do not share the scepticism of the Reviewer #1 regarding the work by Laursen and Møller (2021), though we agree that this study shows prey size preferences at some given geographic location. Hence, we agree that the described relationship was perhaps not general enough to be used as a main motivation of our work. The

sentence in the Abstract has been replaced with one that now represents the main justification for our work. As the main justification for our work, we refer to the well-supported relationship of thiamine concentration and the mass-specific metabolic rate that decrease with body size at the interspecific level. In a new paragraph of the introduction (L90-104), we cite papers showing that body size allometry of metabolic rate sets the demand for water soluble vitamins and explain why the allometry of metabolic rates in blue mussels can lead to a decrease in thiamine concentration with body size. Our results confirm this expectation.

Please note that Reviewer #2 (see Response #70) suggested to discuss the link between brain size and consumed prey rather in the Discussion section. Hence, we no longer refer in the Abstract and Introduction to the link between brain size and consumed prey and work by Laursen and Møller (2021). Instead, the text in the Introduction on the link between brain size and consumed prey (Lines 73-90 in the previous version of the manuscript) has been rewritten and moved to the Discussion section (L38-40, 383-385).

6. Abstract, L. 36: You do not investigate size selection of blue mussels in this study, so please replace "whether selection for larger prey" with "whether consumption of larger prey".
Response #15: We agree that the model scenarios, which differ in terms of the size of the prey consumed, are simplified and do not cover several complex aspects of behavioural ecology regarding prey size selection. Please note that our model is already quite complex, for example due to modelling of life history trade-offs or thiamine excretion. Hence, including behavioural aspects of prey size selection could make it hard for analysis and not feasible for understanding by readers. We have corrected the manuscript and now refer to 'consumption of large prey' rather than 'selection for large prey' (L39, 45, 48, 59, 179, 233, 243, 251, 309, 311, 314, 316, 333, 335, 457). Please also note that the work of Laursen and Møller (2021) is now mentioned in the discussion, rather than being used as the main justification for our work (see response #14, #48, #70).

7. Abstract, LL. 37-38: This is not a hypothesis in the traditional sense; it describes the approach adopted. I suggest rewriting this as, for example:
We predicted a negative association between pre-breeding mean mussel size in the diet and modelled egg-thiamine levels, since larger mussels may have a lower thiamine concentration per unit mass due to allometric scaling of metabolism.
Response #16: We have corrected the sentence by removing the controversial statement (L40-42).

8. Abstract, L. 41, "reduced thiamine levels": Your study is an observational one, so you cannot infer causality. This applies to the whole manuscript. Here, for example, please rephrase as: "was associated with lower thiamine levels".
Response #17: This sentence describes the model predictions i.e. an effect of the ecological and physiological mechanisms and feedbacks implemented in the model. Therefore, rephrasing 'reduced thiamine levels' to 'associated with lower thiamine levels' would be both incorrect and misleading. We have corrected this and the following sentences in the Abstract to make it clear that we report the predictions of the model (L44-46, see also Responses #18, #19).

9. Abstract, L. 44-45: The phrasing suggests females adjust clutch size in response to egg thiamine, which is implausible. Any observed changes in clutch size in this eider population are better explained by empirically documented ecological factors such as predation risk, as detailed in my comments below.
Response #18: We have corrected the sentence to clarify that this is the prediction of the model (L48-49).

10. Abstract, L. 44-46: This sentence is overly speculative and should either be omitted, or at least entirely rewritten. Thus, your study does not measure female anti-predator ability, nest predation, or offspring survival. What your results show is only an association between mussel size and modelled egg-thiamine; referring to a mechanism whereby females "best able to cope with predation" produce

thiamine-deficient offspring is untested.
Response #18b: This sentence has been rewritten and now briefly present our results in the context of the mass-specific decrease in thiamine content with body size (L49-51, see Response #48**)**.

11. Abstract, LL. 46-47: See my comment 9 above.
Response #19: We have corrected the sentence to make it clear that this is the prediction of the model (L52-56).

12. Abstract, LL. 47-48: You have not tested the preference (i.e., size selection) of prey in your study, so this part needs to be rephrased.
Response #20: Corrected as suggested (L54-55).

13. Keywords: Remove the word "mass juvenile mortality" as a keyword – it is misleading and tendentious.
Response #21: We changed the keyword 'mass juvenile mortality' to 'high juvenile mortality' to better position rhetoric in our paper between the views of sceptics and supporters of the hypothesis that episodic mass juvenile mortality in common eiders is caused by thiamine deficiency (L59).

14. LL. 56-58: In my view, the PNAS News Feature is grey literature that reports a debate rather than delivering a systematic, peer-reviewed synthesis, and it explicitly acknowledges major gaps which preclude global inference. The most coherent causal story remains the Great Lakes salmonines (alewife thiaminase → low egg thiamine → recruitment failure, mitigation via thiamine supplementation and recovery as alewife declined), but this is a regional case study, not a worldwide pattern.
By contrast, for Baltic common eiders the feature describes smaller clutches and thiamine-deficient eggs, yet more than 90% of duckling losses occurred through herring gull predation after normal hatching, i.e., this case study does not rule out predation as the proximate driver, leaving population-level causation by thiamine deficiency unproven.
More broadly, critics quoted in the article argue that links to population declines are not well established and that other causes (e.g., disease, contaminants) can mimic neurological symptoms; the article itself frames the "global crisis" idea as a hypothesis needing more research. Consequently, this News Feature cannot, on its own, substantiate the strong claim that episodic thiamine-driven juvenile mortality of top marine consumers is a serious conservation problem worldwide. To conclude, you need to temper this statement.
Response #22: We agree that some elements of the scientific debate on thiamine deficiency driven episodic mortality are controversial and we have therefore moderated the language throughout the manuscript. However, we do not agree that PNAS News Feature i.e. the paper by Gilbert 2018 cited in our manuscript is 'grey' literature. PNAS is a top-ranked, highly cited scientific journal with News Feature playing an important role in the emerging trends for scientific research worldwide. Thiamine deficiency has been shown to cause episodic mass mortality in several ecosystems in the Northern Hemisphere, including the Baltic Sea, the Great Lakes, New York Finger Lakes, and California's Central Valley (see citations in Ejsmond et al. 2025, Hylander et al. 2024). Most recently, a group in California (PNAS) published an example of deficiency in a Pacific salmonid and it has been suggested in reptiles (see Mantua et al. 2026 https://doi.org/10.1073/pnas.2426011122) which we now cite in our work (L73). We agree that there is a much stronger support for mass episodic mortality in fish top consumers, but we do not agree that the only support for thiamine-deficiency driven mortality in common eider population comes from grey literature (see Responses #6, #23). In particular, we mean here the work by Mörner et al. (2017) who showed that naturally occurring thiamine deficiency in eggs translated to ca. 7-times higher death rate of common eider ducklings. Hence, while we should be cautious of over-emphasizing the issue (particularly in less studied bird species) we argue that these publications together suggest that we should be open to the possibility of a wide-spread environmental issue.

15. LL. 65-69: The sentence overestimates the extent and certainty of these phenomena from local, circumstantial observations, and thus needs to be tempered. In long-lived eiders, very large year-to-year variation in duckling production is common, and in the Baltic Sea, this variation is now strongly and increasingly shaped by predation – currently overriding climatic and other bottom-up signals (Öst et al. 2022) – so low productivity in a given year and/or site is not diagnostic of a single cause. Furthermore, disease outbreaks may also demonstrably produce severe duckling losses in the region (e.g., Hollmén et al. 2002), further cautioning against single-cause explanations.
Furthermore, the main evidence cited for "mass juvenile mortality" rests on observations from one coastal area and an injection-rescue experiment on artificially incubated ducklings (Mörner et al. 2017). While instructive about thiamine physiology, those results may have limited external validity to field settings with intense predation; carcasses were rarely found and gull predation was frequently observed, making the link to thiamine deficiency in the wild necessarily indirect.
Finally, reduced clutch size is attributed to thiamine deficiency by comparison with historical datasets (Mörner et al. 2017), but clutch size and other breeding decisions are also adjusted to prevailing predator pressure (e.g., Mohring 2025) – an increasingly dominant driver in this system – so confounding by predation and concomitant changes in breeding strategies, reproductive investment and predator-induced partial or complete clutch loss cannot be ruled out.
References:
Hollmén T, Franson JC, Kilpi M, Docherty D, Hansen WR, Hario M (2002) Isolation and characterization of a reovirus from common eiders (*Somateria mollissima*) from Finland. Avian Diseases 46: 478-484.
Mohring B. 2023. Breeding during a predation regime shift: behavioural and physiological responses of female common eiders facing the recovery of their native predator (Doctoral dissertation, Université de La Rochelle & Åbo Akademi University).
Mörner T, Hansson T, Carlsson L, Berg AL, Ruiz Muños Y, Gustavsson H, Mattson R, Balk L. 2017. Thiamine deficiency impairs common eider (*Somateria mollissima*) reproduction in the field. Sci Rep 7:14451.
Öst M, Lehikoinen A, Jaatinen K. 2022. Top-down effects override climate forcing on reproductive success in a declining sea duck. Oikos 2022: e08762.
Response #23: We disagree that the aforementioned sentence overestimates "the extent and certainty of these phenomena from local, circumstantial observations, and thus needs to be tempered". However, we have modified the sentence to keep the rhetorics of the text a more balanced in between the sceptics and supporters of the 'mass mortality caused by thiamine-deficiency' hypothesis (L73-75).

Changes in the age structure and population numbers of wintering common eiders show a sharp decline in recruitment to the first-year age class (see Figures 2a and 2b in Ekroos et al., 2012). Our manuscript now clearly acknowledges the importance of predation and clarifies that the mortality of adult females at nesting sites is likely a key driver of the recent decline in population numbers (L383-385, Response #25). In our work, we cite the research of Mörner et al. (2017), who demonstrated a sevenfold increase in the mortality rate of ducklings due to thiamine deficiency in controlled laboratory conditions and an injected-rescue experiment. We also referred to scientific papers (Balk et al., 2009; Balk et al., 2016) and research reports (Svedäng et al., 2018). In our opinion, this provides a sufficient support for considering thiamine deficiency as a factor that can contribute to the decline in recruitment of juveniles and population number of Baltic eiders (see also Responses #6, #22).

Please note that thiamine deficiency-driven mass mortality in fish affect specific populations in only some years (e.g. Majaneva et al., 2020), which makes the phenomenon difficult to investigate. Also note that physiological dysfunctions caused by thiamine deficiency would manifest as elevated vulnerability to predation and disease due to abnormal behaviour, impaired locomotion and metabolic dysfunction. Furthermore, falsifying the thiamine-deficiency-driven mortality hypothesis would require measuring this micronutrient concentration in field studies, as the organism's condition is

strongly dependent on its metabolism. In our view, this may be a question of the ultimate and proximate drivers of the negative population trends in Baltic common eiders.

16. LL. 70-71: You need to moderate the claim about episodic mass juvenile mortality of eiders in the Baltic Sea, and particularly any insinuation that these are causally linked to thiamine deficiency. See, for example, my previous comment 15.
Response #24: We disagree with Reviewer #1's assertion that the part in question is an insinuation (see Responses #6, #22, #23). However, we have modified the sentence to keep the rhetorics of the text a more balanced in between the sceptics and supporters of the 'mass mortality caused by thiamine-deficiency' hypothesis (L79-83).

17. LL. 73-75: This sentence misrepresents the evidence for the decline of the Baltic eider population. In long-lived birds such as eiders, population growth (λ) is most sensitive to adult survival rather than fecundity (Öst et al. 2016). Thus, recent work indicates that elevated predation-induced mortality of incubating females, also reflected in the increasingly male-biased adult sex ratio, better explains recent declines than nest predation or duckling mortality alone (Öst et al. 2016; Ramula et al. 2018; Tjørnløv et al. 2020). Please revise accordingly. For example: "Increased predation on breeding females, together with nest predation and duckling mortality, likely underlies recent negative trends in the Baltic population." References: see comment 4 above.
Response #25: This sentence has been corrected and moved to Discussion in line with Reviewer #2 suggestions (L383-386 see Responses #70, #14) and now acknowledge the importance of the elevated mortality of incubating females induced by predation in the recent decline in the number of Baltic eiders. (see also Response #13).

18. LL. 77-79: This sentence is cryptic. Please consider rewording it for increased transparency. For example: "In Baltic eiders, individual differences in brain (central nervous system) size and female behavior are associated with variation in nesting success under predation pressure (Öst and Jaatinen 2015; Jaatinen et al. 2019)."
Response #26: This sentence has been reworded according to Reviewer #1 suggestions and moved to Discussion in line with Reviewer #2 suggestions (L386-388, see Responses #70, #14).

19. LL. 79-81: This sentence misinterprets both the findings and mechanism presented in the cited work. Öst and Jaatinen (2015) examined nest success, timing of breeding, and post-hatch coalition formation—not recruitment—and formation of brood-rearing coalitions occurs after hatching, affecting duckling survival rather than pre-hatch nest success.
Instead, the evidence shows that relatively large-headed (large-brained) females breed later and use more cautious nest-site selection; this delay confers higher nest success specifically in 'dangerous' years when predation pressure peaks early, while the reverse holds in benign years with early phenology (Jaatinen et al. 2019). During such conditions, large-headed females also have a survival advantage, which is reversed under relaxed predation pressure (Jaatinen et al. 2019). To conclude, please revise this section to avoid invoking recruitment and to attribute the pattern to brain-size-dependent timing of breeding rather than coalition speed.
Response #27: In our work, we have used the term 'recruitment' as it is routinely interpreted in life history, i.e. the likelihood that a juvenile will enter next age classes, rather than as a phenomenon closely related to fecundity. To avoid further misunderstanding, we now refer to offspring survival. This sentence has been corrected and moved to Discussion in line with Reviewer #2 suggestions (L388-390, see Responses #70, #14).

20. LL. 82-85: This section is not a fair representation of what these studies actually found, so this part needs to be rewritten. Jaatinen et al. (2019) did not show a general survival advantage for large-brained females; rather, survival selection on relative head/brain size fluctuated annually—large-headed females out-survived small-headed ones in dangerous years, whereas the reverse held in benign years, consistent with brain-size-dependent breeding phenology as the mechanism. By

contrast, the Nordman MSc thesis (2024) MSc does not address relative head/brain size; it analyzes potential temporal change in structural size (radius-ulna), finding an increase over 2008–2023 and no evidence for size-selective predation on incubating females. It therefore cannot be used to infer a brain–body size correlation.
Response #28: Thank you for the comment. In line with the hints by Reviewer #1, we have removed the sentence that referred to the results presented in the Master of Science Thesis by Nordman (2024) (L114-116).

21. LL. 85-87: First, "the superiority of females with large brains under high predation" is an overinterpretation that needs to be tempered (see my comments 19 and 20 above). Second, this sentence overstates what Laursen & Møller (2021) shows. In that study, prey-size data came from gizzard contents, and the core predictor was head volume as a proxy for brain mass. Importantly, the inference is about what was eaten, not observed *preference* relative to local availability. The GLMs included did not include predation intensity as a predictor—and the sample comprised 198 eiders collected in a single Danish wintering region. For blue mussels, head volume was positively associated with mean and maximum mussel size, but the authors' across-prey effect size (estimate ≈ 0.10 ± 0.02) is not readily interpretable as birds "prefer considerably larger" prey. Critically, the result was not female-specific: the sex term was non-significant for blue mussels, and where sex differences did occur, female gizzards contained smaller prey. Thus, Laursen & Møller (2021) paper does not demonstrate that females with larger brains prefer considerably larger prey, nor that such an association is linked with predation intensity.
Response #29: The sentence has been changed as we agree that the paper by Laursen and Møller (2021), concerns prey size preferences in a certain geographic location (see Response #14). We have tempered rhetoric in this sentence according to reviewers' suggestions and moved to Discussion in line with comments by Reviewer #2 suggestions (L390-393, see Responses #70, #14).

22. LL. 87-88: Baltic eiders are not exceptional in their preference for blue mussels; they are opportunistic feeders, and where blue mussels are abundant, like in the Baltic Sea, but also elsewhere (e.g. Guillemette et al. 1992), they are the preferred prey.
Reference:
Guillemette M, Ydenberg RC, Himmelman JH. 1992. The role of energy intake rate in prey and habitat selection of common eiders in winter: a risk-sensitive interpretation. J Anim Ecol 61:599–610.
Response #30: Thank you for the comment. The sentence has been corrected according to suggestions by Reviewer #1 and moved up in the Discussion (L87-90, 119-122).

23. LL. 91-93: This statement rests on several unsupported assumptions. First, as explained in comment 21 above), Laursen and Møller (2021) did not measure active prey-size preference or selection relative to local availability; their inference is from gizzard contents only, with no concurrent sampling of benthic size distributions. Second, the study was conducted with no measure of predation intensity included among predictors, so the claim that larger mussel consumption is adaptive under increased predation at nesting sites is outside the evidence base. Please rephrase this section for retaining objectivity.
Response #31: Thank you for your comment. Please note that Reviewer #1 raised concerns about the insufficient generality in the assumption that eiders with larger brains select larger prey (see Responses #14 and #15), and Reviewer #2 recommended moving these considerations to the Discussion section (see Response #70). The sentence in question has therefore been rewritten to represent the main justification for our work i.e. decrease with body size of size-specific thiamine content and metabolic rate (L122-125) (see response #14).

24. L. 94: Please consider replacing 'preference for' with 'consumption of', since you did not investigate prey preference in your study.
Response #32: Replaced (L126).

25. LL. 97-101: An interested question: your optimization framework seems tailored to eiders remaining near colonies year-round, yet most Baltic eiders are migratory mixed capital breeders whose diet, and therefore mussel size distributions, shift seasonally between wintering and breeding areas (Hobson et al. 2015, Jaatinen et al. 2016). Individuals also vary in the balance of 'imported' verus local reserves, with early-laying, good-condition females relying more on stores gathered at the wintering quarters, while others feed more locally prior to laying (Jaatinen et al. 2016). Moreover, predation risk has lengthened the spring arrival-to-nesting interval in northern parts of the Baltic Sea (Öst et al. 2022), expanding the scope for pre-laying foraging at the breeding locales.
Given all the above complexity, could you clarify how your model accommodates (or could be extended to accommodate) these axes of heterogeneity, i.e., seasonal prey-size shifts across areas, state-dependent capital versus income use, and variable arrival-to-lay intervals, and whether your central result that small shifts toward larger mussels depress egg thiamine remains robust under such realistic variation?
References:
Hobson KA, Jaatinen K, Öst M. 2016. Differential contributions of endogenous and exogenous nutrients to egg components in wild Baltic common eiders (*Somateria mollissima*): A test of alternative stable isotope approaches. Auk 132:624–633.
Jaatinen K, Öst M, Hobson KA. 2016. State-dependent capital and income breeding: A novel approach to evaluating individual strategies with stable isotopes. Frontiers in Zoology, 13: 24.
Öst M, Lehikoinen A, Jaatinen K. 2022. Top–down effects override climate forcing on reproductive success in a declining sea duck. Oikos 2022:e08762.
Response #33: Thank you for the comment. Our model was originally designed for long-distance migratory populations of common eiders, where females arrive at the breeding grounds in a lean body condition (Ejsmond et al., 2021; Ejsmond and Ejsmond, 2022). The reviewer mentions papers showing that, in the Baltic population, the proportion of reproductive investment covered by reserves brought by females from wintering grounds may vary. We now present the results of additional simulations in which females arrive at the breeding grounds with reserves (see Response #38, #46 for details). Please note that the conclusions of our work did not change regardless of the contribution of reserves gathered outside the breeding grounds to reproduction.

However, we are sceptical about using in our work the definition of capital breeding adopted by Reviewer 1. The terms 'capital breeding' and 'income breeding' are typically used to describe the degree to which reproductive investment is covered by stored internal reserves. The inclusion of the distinction between 'imported' versus 'local' reserves in the term 'capital breeding' has been criticised (e.g. Bonnet et al. 1998; Stephens et al. 2009). This is mainly because numerous taxa that store extensive reserves for reproduction are considered capital breeders but do not migrate or feed during winter hibernation (Bonnet et. al. 2001, Stephens et al. 2014, Ejsmond et al. 2018). Overall, capital breeding is associated with the distinction between 'local' and 'imported' reserves only in a relatively narrow cases of certain bird taxa e.g. migratory water birds. Please note that for example owls are to a high degree capital breeders, with many species being mainly sedentary. Therefore, we would like to retain a more general definition of capital breeding that does not refer to the distinction between 'imported' versus 'local' reserves. We now clarify in L163-167 that capital breeding is defined as no concurrent feeding during reproduction and the accumulation of reserves that are used later for breeding.

Bonnet, X., D. Bradshaw, and R. Shine. 1998. Capital versus income breeding: an ectothermic perspective. Oikos 83:333–342.
Bonnet, X., G. Naulleau, R. Shine, and O. Lourdais. 2001. Short-term versus long-term effects of food intake on reproductive output in a viviparous snake, Vipera aspis. Oikos 92:297–308.

Ejsmond, M.J., McNamara, J.M., Søreide, J., and Ø. Varpe. Gradients of season length and mortality risk cause shifts in body size, reserves and reproductive strategies of determinate growers. Functional Ecology. 2018;00:1–12.

Stephens, P. A., I. L. Boyd, J. M. McNamara, and A. I. Houston. 2009. Capital breeding and income breeding: their meaning, measurement, and worth. Ecology 90:2057–2067.
Stephens, P. A., A. I. Houston, K. C. Harding, I. L. Boyd, and J. M. McNamara. 2014. Capital and income breeding: the role of food supply. Ecology 95:882–896.

26. LL. 103-104: In my opinion, these empirical observations represent the most valuable contributions of this study. The biochemical analyses seem meticulously conducted, and the illustrations included are impressive.
Response #34: Thank you for appreciating certain elements of our work.

27. LL. 108-111: One potentially important factor that was not considered in this study was the state of sexual maturation/gonadal development of the analyzed mussels, which may affect not only the spatial variation in thiamine levels, but, more importantly, the observed size-dependence of thiamine levels in mussels. Please consult my comment 41 below for more detailed discussion.
Response #35: See L423-429 and Response #49.

28. LL. 120-122: More recent research has provided a more nuanced view of the breeding strategies in this species; it is nowadays considered a mixed capital breeder, relying both on reserves acquired at the wintering grounds and on food ingested at the breeding sites for financing the costs of egg production and incubation (Sénéchal et al. 2011, Hobson et al. 2015, Jaatinen et al. 2016).
References:
Hobson KA, Jaatinen K, Öst M. 2016. Differential contributions of endogenous and exogenous nutrients to egg components in wild Baltic common eiders (*Somateria mollissima*): A test of alternative stable isotope approaches. Auk 132:624–633.
Jaatinen K, Öst M, Hobson KA. 2016. State-dependent capital and income breeding: A novel approach to evaluating individual strategies with stable isotopes. Frontiers in Zoology, 13: 24.
Sénéchal É, Bety J, Gilchrist HG, Hobson KA, Jamieson SE. 2011. Do purely capital layers exist among flying birds? Evidence of exogenous contribution to arctic-nesting common eider eggs. Oecologia 165:593-604.
Response #36: See L163-167 we now also present scenarios in which females arrive at breeding grounds with reserves (for more details see Response #33, #38, #46).

29. LL. 143-144: Please note that although stopovers occur, evidence suggests that the spring migration of Baltic common eiders is highly synchronized and fast, leaving little time for prolonged staging (e.g., Alerstam et al. 1974).
Reference: Alerstam T, Bauer C, Roos G. 1974: Spring migration of eiders *Somateria mollissima* in southern Scandinavia. Ibis 116: 194–210.
Response #37: Please note that, the optimal life history strategies generated by our model (i.e. those that maximised fitness), females spent relatively little time at migration stopovers in spring (see Fig. 2A). Also note that changes in time spent on stopovers during migration, due to the manipulation of migration cost, did not affect the conclusions of our work, i.e. feeding on large prey decreased thiamine concentration in the eggs regardless of the time spent on stopovers (see e.g. Fig. 7 in S1 Appendix) or if females skip stopover during spring migration (see Fig. 5A-B in S1 Appendix).

30. LL. 147-149: I was puzzled by the assumption that females with high reserves would reach their destination close to or in a lean body condition" and by its formalization in Appendix S1, where birds departing with maximal reserves are forced to arrive essentially lean via eq. S.1 (Sa) and fixed migration costs (400 g per leg to the breeding area) (Appendix S1).
For Baltic common eiders, this seems biologically unlikely for several reasons:
1. Spring migration is short, rapid and highly synchronized (Alerstam et al. 1974), and northward movements may often occur without stopovers. Thus, the need to consume large stores *en route* is limited.
2. Empirical data indicate that females depart Danish wintering grounds in good body condition.

Laursen et al. (2019) report a mean adult female body mass of 2.23 kg in late winter (departure). These patterns are inconsistent with systematic arrival in a lean state.
3. Stable-isotope evidence from the northern Baltic shows that early-laying, heavy females predominantly finance yolk formation from stored (endogenous) reserves, shifting to more local income later in the season, as a part of their mixed capital-breeding strategy (Jaatinen et al. 2016). This requires arriving with significant amounts of stored reserves, particularly for early breeders. Because the model forces birds that depart with large reserves to arrive nearly lean, it likely underestimates early-season capital use and could skew your thiamine and fitness projections. Can you comment, please?
References:
Alerstam T, Bauer C, Roos G. 1974: Spring migration of eiders *Somateria mollissima* in southern Scandinavia. Ibis 116: 194–210.
Jaatinen K, Öst M, Hobson KA. 2016. State-dependent capital and income breeding: A novel approach to evaluating individual strategies with stable isotopes. Frontiers in Zoology 13: 24.
Laursen K, Møller AP, Öst M. 2019. Body condition of eiders at Danish wintering grounds and at pre-breeding grounds in Åland. Journal of Ornithology 160: 239–248.
Response #38: Thank you for your comment. See Response #37 regarding the time spent on stopovers. With regard to females arriving at the breeding grounds in a lean body condition in spring, we assumed that, according to the data for some populations according to the book The Common Eider by Waltho and Coulson (2015). However, we agree that, in the case of a short winter period or short migration distance, the condition of females arriving at the breeding grounds in the Baltic population can be much better than just lean. Therefore, we now present additional simulations exploring scenarios in which mild winters cause females to depart from wintering grounds early and arrive at the breeding grounds with reserves. These additional simulations show that the conclusions of our work did not change when we allowed females arrive at the breeding grounds with high reserves level (see Fig. 5A in S1 in Appendix 1). We also briefly mention this in the main text (L194-196).

31. LL. 154-157: The conclusion that female eiders feed during incubation should be interpreted with some degree of caution. Rigou and Guillemette (2010) show that incubating females very occasionally dive for a few minutes per day, but their loggers cannot demonstrate prey ingestion. In contrast, Parker and Holm (1990) reported complete fasting during incubation in a high-Arctic population of eiders. As a sensitivity check, please consider re-running the model assuming no feeding occurs during incubation.
Response #39: We have now clarified that our model was run with no feeding during incubation (L 202-205).

32. LL. 157-160: You may want to include some references demonstrating lower reproductive success with advancing laying date in eiders as well as, such as Descamps et al. (2011) and Jaatinen et al. (2013).
References:
Descamps S, Bêty J., Love OP, Gilchrist HG. 2011. Individual optimization of reproduction in a long-lived migratory bird: A test of the condition-dependent model of laying date and clutch size: Optimal reproduction in a long-lived bird. *Functional Ecology* 25: 671–681.
Jaatinen K, Seltmann MW, Hollmén T, Atkinson S, Mashburn K, Öst M. 2013. Context dependency of baseline glucocorticoids as indicators of individual quality in a capital breeder. General and Comparative Endocrinology 191: 231–238.
Response #40: Thank you for the suggestions. We have now cited the articles that investigated the common eider (L207-208).

33. LL. 168-170: The assumption that female eiders with extensive body reserves have lower survival appears at odds with the available empirical evidence. First, Guillemette and Ouellet (2005) document

a mechanistic risk—temporary flightlessness in still air during late pre-laying—and they themselves caution against directly inferring survival costs from impaired take-off alone. Furthermore, the mass increase they quantify is largely due to ovarian growth while somatic mass remains roughly constant, so ‘extensive reserves’ per se may not show the assumed relationship with survival. Indeed, longitudinal capture-mark-recapture data from Baltic eiders show that female apparent survival increases with body condition measured at the first known breeding event (variable chosen to minimize age effects), with habitat type included as a covariate. There are several, not mutually explanations why we should expect a positive association between body condition and survival, including the asset protection principle (e.g., Clark 1994) or state-dependent safety (e.g., Luttbeg and Sih 2010).
In light of these uncertainties, I encourage the authors to add a sensitivity analysis showing whether the key conclusions are robust when the body reserve-survival link is either removed, or reversed to a small *positive* condition effect outside the brief pre-laying window, consistent with the empirical patterns reported in Ekroos et al. (2012).
References:
Clark CW. 1994. Antipredator behavior and the asset-protection principle. Behavioral Ecology 5: 159–170.
Luttbeg B, Sih A. 2010. Risk, resources and state-dependent adaptive behavioural syndromes. Philosophical Transactions of the Royal Society B: Biological Sciences 365: 3977–3990.
Response #41: In our model we followed a classic ecological theory, according to which the optimal level of fat reserves in birds is determined by the trade-off between starvation and predation. Fat reserves create the energetic buffer to sustain metabolism but increase vulnerability to predation due to impeded locomotion (e.g. Lima 1986, McNamara and Houston 1990, Rogers 2015, Zimmer et al. 2011). The papers by Lima (1986) and McNamara and Houston (1990), which introduced the concept have been cited over thousand of times in ecological and evolutionary literature, with tremendous amount of supporting evidence. According to the theory, the level of fat reserves in birds is optimised, not maximised. Hence, monotonic positive relationship between the mass of fat reserves and the survival of females, suggested by Reviewer #1, is inconsistent with the theory and the supporting evidence.

Please note that in our model, the survival rate of females with insufficient body reserves is substantially reduced (see L216-219). This accounts for the ecological role of storage in sustaining physiological needs, as referred to by Reviewer #1 in their comment. The paper by Clark (1994) referred to by Reviewer #1, discusses maximization of the reproductive value. According to classic life history theory, the reproductive value at a given age is essentially the cumulative expected offspring production (i.e. weighted by survival prospects), discounted by population size trends. According to Clark's paper, excessive fat reserves that impede flight and predator avoidance abilities in birds and the common eider (cf. Guillemette and Ouellet, 2005) would decrease survival and thus reproductive value. The paper by Luttbeg and Sih (2010), also referred to by Reviewer #1, uses the state-dependent optimisation approach, i.e. the approach adopted also by McNamara and Houston (1990) who proposed the trade-off between starvation and predation.

Luttbeg and Sih (2010) demonstrated that the accumulation of substantial reserves enhances fitness, when individuals with greater energy reserves, are better at coping with predators. However, the common eider is often described as being 'at the limit of flight capability in vertebrates' (see Guillemette and Ouellet, 2005), and individuals with extensive body reserves are more vulnerable to predators due to impaired flight abilities. While the prevailing evidence for the starvation-predation trade-off in birds concerns passerines, which may not be particularly relevant to our work, there is also evidence in other groups of birds, including ducks (e.g. Zimmer et al., 2011). We now cite this paper in our work to support the assumption of an elevated mortality risk for birds with extensive body reserves (L218-219).

- Lima, S. L. 1986. Predation risk and unpredictable feeding conditions: Determinants of body mass in

birds. Ecology 67:366–376.
- McNamara J.M., Houston, A. 1990. The value of fat reserves and the trade-off between starvation and predation. Acta Biotheoretica 38:37–61.
- Rogers, C. M. 2015. Testing optimal body mass theory: Evidence for cost of fat in wintering birds. Ecosphere 6(4):55.
- Zimmer C, Boos M, Poulin N, Gosler A, Petit O, et al. (2011) Evidence of the Trade-Off between Starvation and Predation Risks in Ducks. PLoS ONE 6(7): e22352.

34. LL. 184-187: Here, you envision a cost of reproduction trade-off. This is a strong claim in need of supporting evidence. Please either (i) cite empirical or theoretical work showing micronutrient (ideally thiamine or close analogs) retention by mothers at the expense of eggs in birds, or (ii) rephrase to avoid implying a general rule or active maternal restraint. Also note that elsewhere you write that thiamine cannot be long-term stored (liver half-life ca 2.5 weeks, LL. 132-133), which seems at odds with the hypothesized maternal 'retention' of these micronutrients. Consider instead framing the assumption neutrally (e.g., egg thiamine reflects maternal status at laying).
Response #42: Thank you for your comment. We have corrected the text in line with the reviewer's advice by removing the sentence that referred to the anticipated evolutionary rationale for the allocation of thiamine by female common eiders. Instead, we now cite the only study that provides insights into how thiamine concentrations in gonads and somatic tissues change at the intraspecific level (L235-241). Our study of Atlantic salmon found that the thiamine status of the gonads and muscles correlates (Ejsmond et al. 2025). This relationship becomes more proportional as the mass of the gonads increases shortly before breeding (Ejsmond et al. 2025).

Ejsmond MJ, Todisco V, Hauber MM, Hindar K, Hylander S. Physiological Mechanisms and Life History Trade-Offs in Salmonids Shape In-Tissue Correlations of an Essential Micronutrient. Ecology and Evolution. 2025 Oct 16;15(10):e72339.

35. LL. 194-196: This wording reads as if prey-size preference was estimated from your data. However, neither the Methods nor Appendix S1 present empirical estimates of prey-size selection by eiders at the wintering, stopover, or breeding sites. The mussel work provides thiamine-mussel size relationships from lab-processed, size-stratified samples, with pooling of small individuals and subsampling of large ones to achieve target tissue masses. This design ensures coverage of the full size range, but does not represent ambient benthic size distributions of mussels, nor does it include diet or foraging data from eiders (e.g., gizzard contents).
In the main text you also frame prey-size differences as scenario comparisons rather than quantities estimated from field data (LL. 133-135), and Fig. 1D shows the assumed daily intake distribution, rather than empirical preference.
To remedy the situation, I suggest clarifying that prey-size 'preference' is an assumed model parameter explored in scenarios (with the specific size range you use), not something derived from your sampling.
Response #43: We have corrected the sentence by removing the link to prey-size preference and clarifying that we meant scenarios in which the size of the prey consumed differs (L251).

36. L. 211: Please define the acronym RMR; it is not common knowledge to all readers.
Response #44: The RMR acronym has been defined earlier (see L176 in the previous version of the manuscript).

37. LL. 219-222: Please note that a 1 cm shift is big relative to what female eiders actually eat, particularly in the central and northern parts of the Baltic Sea. Thus, in the western Gulf of Finland, females typically ingest mussels in the size range of 17-18 mm, which is below the mean mussel size at the bottom (21 mm). A 10 mm change would move diets beyond the central, observed diet size range. To increase realism, please consider using smaller steps in your scenarios (e.g., 2–5 mm steps), and/or clarify that 1 cm increments reflect an analytical unit rather than a typical behavioural shift in

this study system.
Response #45: The 1 cm increment was mentioned as an example for visualisation purposes. In our model, we did not explicitly modelled shifts in prey size. We investigated scenarios in which the prey size was fixed, and these scenarios differed from each other by 0.5 cm of mussel length (see, for example, the legend in Fig. 2). This paragraph has been rewritten in response to Reviewer #2's suggestion that a more detailed statistical analysis of the empirical results is needed (see L281-298, and Response #71).

38. LL. 239-244: The assumptions underlying these results are problematic for several reasons, as they seem inconsistent with the current reality in the Baltic Sea and conflict with well-established phenology-fecundity patterns.
First, under the current predation regime, rapidly intensifying predation (notably by white-tailed eagles) is associated with later breeding, a longer arrival-to-laying interval, reduced breeding synchrony, and smaller clutches (Öst et al. 2022); these are classic fear-effects of increased predation danger, which delay breeding despite the benefits of early hatching and favour smaller clutches.
Second, in eiders (Descamps et al. 2011, Lehikoinen et al. 2006, Öst et al. 2022) and birds in general (e.g., Verhulst and Nilsson 2008), earlier laying is linked to larger clutches (clutch size typically declines with laying date), so the hypothesized causality (earlier onset leads to smaller clutches) runs counter to the theoretical and empirical evidence.
I suggest clarifying the scope of this Result, as it may be most likely to hold under low predation risk settings. Furthermore, to reconcile the model with observed long-term trends in the Baltic Sea, you may consider exploring scenarios which allow adult/nest predation risk to vary, and to model clutch size as condition- and timing-dependent, assuming that better body condition and earlier laying is associated with larger clutches.
References:
Descamps S, Bêty J., Love OP, Gilchrist HG. 2011. Individual optimization of reproduction in a long-lived migratory bird: A test of the condition-dependent model of laying date and clutch size: Optimal reproduction in a long-lived bird. Functional Ecology 25: 671–681.
Lehikoinen A, Kilpi M, Öst M. 2006. Winter climate affects subsequent breeding success of common eiders: Global Change Biology 12: 1355–1365.
Öst M, Lehikoinen A, Jaatinen K. 2022. Top–down effects override climate forcing on reproductive success in a declining sea duck. Oikos 2022:e08762.
Verhulst S, Nilsson J-Å. 2008. The timing of birds' breeding seasons: A review of experiments that manipulated timing of breeding. *Philosophical Transactions of the Royal Society B: Biological Sciences* 363: 399–410.
Response #46: Thank you for your constructive comment. We now show that our model predicts reduction in clutch size with postponement of nesting onset and that thiamine level in the eggs increases with reduction in clutch size, no matter the phenological causes of clutch size reduction (see L323-329).

The reaction of clutch size to breeding date is not uniform across populations of common eider. Our modelling framework was tailored for long-distance migrating common eiders in which females arrive at breeding grounds in lean body condition (see Response #38) and in which early spring onset is associated with smaller clutches (our study, Ejsmond et al. 2021). We agree that in at least some of the breeding populations of Baltic eiders, but also other North Atlantic populations, females lay more eggs when spring arrives early (Descamps et al. 2011, Öst et al. 2022). Overall common eider populations seem to be variable with this respect. We now present additional simulations exploring the scenario in which mild winter is associated with arrival of females with reserves (see Fig. 5 in S1 Appendix 1). In these new simulations clutch size decreases with the date of nesting i.e. in a similar manner as suggested by the Reviewer 1 and work by Descamps et al. (2011) and Öst et al. (2022).

Reviewer #1 pointed out that changes in phenology, such as altered nesting onset and reduced

clutch size, are mediated by the intensification of predation (notably by white-tailed eagles). In the S1 Appendix, we present the outcomes of scenarios involving changes in the breeding phenology consistent with a reaction to elevated predation by white-tailed eagles (see Fig. 5 in the S1 Appendix). Please note that we have also tested the sensitivity of the model predictions to various combinations of mortality rates (see Fig. 6A–C in the S1 Appendix), and in all tested scenarios, consumption of large prey reduced egg thiamine levels, with a reduction in clutch size being associated with increase in egg thiamine levels.

39. LL. 252-255: This is an overstatement considering what the results actually indicate. Your study combines measured mussel size-thiamine content relationships with a modelled life-history/physiology framework and then compares assumed prey-size preference scenarios; it does not measure foraging time, energetic trade-offs, or prey-size selection in the field, which are key elements of optimal foraging theory. I suggest softening this claim to reflect that the study complements energy-focused optimal foraging theory by highlighting a micronutrient constraint rather than claiming to go "far beyond" it.
Response #47: The sentence has been rewritten, and it now states that the classic optimal foraging theory fails to account for fitness consequences caused by changes in the ingestion of metabolic micronutrients due to preference of prey of given size (L340-345).

40. LL. 255-257: Again, this overstates what the study shows. The manuscript documents a negative thiamine content-size relationship in mussels and then explores assumed prey-size scenarios within a life-history model with assumed daily intake; it does not quantify foraging costs or empirical prey selection, so an "unavoidable trade-off" is not demonstrated here. Please soften the statement.
Response #48: Thank you for your comment. We have softened the sentence by removing the word 'unavoidable' (L345-347). Please note that we now elaborate in the Introduction on the link between body size, metabolic rate, and the physiological demand for thiamine at the interspecific level (L90-104). In this paragraph, we also explain why intraspecific variation in metabolic rates in blue mussels can lead to a decrease in thiamine concentration with body size. Our results confirm this expectation. Therefore, our study complements the scientific literature's view that thiamine concentration decreases with body size at the intra- and interspecific levels. Please also note that, in this sentence, we are proposing a general implication rather than making a strong statement by using the phrase 'may lead to'.

41. LL. 257-266: One of the key findings is the negative relationship between mussel size and thiamine concentration, which is interpreted as a consequence of decreasing mass-specific metabolic rates in larger individuals. While this explanation is plausible, I would like to offer an alternative, potentially complementary hypothesis: the observed pattern may instead reflect size-dependent reproductive status at the time of sampling.
The rationale of this hypothesis is the following: In Baltic Sea blue mussel populations, individuals typically reach sexual maturity between 15–25 mm in shell length, depending on salinity and environmental conditions (Kautsky 1982). Thus, smaller mussels (< 2.5 cm) sampled in June likely include many immature individuals that had not yet initiated gametogenesis, whereas larger mussels were presumably at various stages of reproduction or post-spawning depletion. Given that thiamine levels are known to decline during reproductive investment (e.g., Seguineau et al. 2001ab), the mussel size–thiamine content gradient may reflect physiological shifts linked to maturity rather than only metabolic scaling.
Importantly, this has broader implications: while the metabolic-rate explanation implies a general, year-round disadvantage of consuming large mussels from a thiamine deficiency perspective, the reproductive-status mechanism suggests that such disadvantages are temporary and seasonal, peaking around the reproductive period. This distinction may limit the temporal generality of the observed size-thiamine pattern and should be discussed explicitly. In this context it is pertinent that all sampling of blue mussels in the manuscript were conducted during a narrow time window in June, coinciding

with the peak phase of reproduction in mature Baltic blue mussels.
References:
Kautsky N. 1982. Growth and size structure in a Baltic *Mytilus edulis* population. Marine Biology 68: 117–133.
Seguineau C, Saout C, Paulet YM, Muzellec ML, Quéré C, Moal J, Samain J-F. 2001a. Changes in tissue concentrations of the vitamins B1 and B2 during reproductive cycle of bivalves: Part 1: The scallop *Pecten maximus*. Aquaculture 196: 125–137.
Seguineau C, Migaud H, Quéré C, Moal J, Samain J-F. 2001b. Changes in tissue concentrations of the vitamins B1 and B2 during reproductive cycle of bivalves: Part 2: The Pacific oyster *Crassostrea gigas.* Aquaculture 196: 139–150.
Response #49: Yes, this is a valid and interesting comment. A negative relationship would emerge if small and large mussels have different investments in reproduction. However, in another project we have quantified thiamine in a broader range of mussel populations across the Baltic Sea and the negative relationships shown here are also present in September-October, i.e. outside the main reproduction period. This suggests that the metabolic-rate explanation is valid. However, this is an interesting aspect, and we have now mentioned this caveat in the discussion (L423-429) adding the two references indicated by Reviewer #1.

42. LL. 267-277: This paragraph is speculative and needs to be either removed or entirely rewritten. The hypothesis presented here implies that females may select small mussels for their thiamine content. However, to the best of my knowledge, there is no empirical evidence that eiders (or vertebrate consumers generally) can assess prey vitamin content during feeding, and none is presented here. By contrast, there is strong, long-standing evidence that eider foraging is constrained by shell processing and digestion: eiders swallow prey whole, ingest large masses of inorganic shell each day, and the gizzard grinding-defecation process is a major, time-consuming component of the feeding cycle, with resting bouts devoted to shell crushing (e.g., Guillemette et al. 1992, Hario and Öst 2002). These digestive constraints, not micronutrient sensing, are the established mechanism behind selection for smaller mussels and minimization of shell mass.
A further argument pointing to the improbability of this hypothesis is that the text reads as if micronutrient-driven evolutionary responses might already shape prey-size selection. Given the (presumably) short and uncertain time window over which thiamine deficiency may have been relevant in the Baltic Sea, such adaptation is speculative without evidence. I recommend removing or softening any implication of rapid evolutionary change and framing your results as proximate, testable predictions about nutritional constraints that might influence behaviour, a possibility rather than a demonstrated evolutionary response.
References:
Guillemette M, Ydenberg RC, Himmelman JH. 1992. The role of energy intake rate in prey and habitat selection of common eiders *Somateria mollissima* in winter: a risk-sensitive interpretation. Journal of Animal Ecology 61: 599–610.
Hario M, Öst M. 2002. Does heavy investment in foraging implicate low food acquisition for female common eiders *Somateria mollissima*? Ornis Fennica 79: 111-120.
Response #50: We have removed the text, as Reviewer #3 also found the paragraph in question problematic (L357-367). Although our statement in this paragraph, '…common eider females may select small blue mussels for their thiamine content', was not intended to imply that diet choices are based on micronutrient content, we agree that this sentence could be interpreted as such. Please note that the removed paragraph was a relatively small part of the discussion.

43. LL. 278-289: This paragraph assumes a general preference of large prey, but this is not demonstrated for your focal system and may, in fact, be contradicted by empirical evidence from the Baltic Sea and elsewhere. For example, Öst and Kilpi (1998) found no unambiguous size-selective feeding and note that, if anything, there may have been a preference for consuming mussels slightly smaller than the average found on mussel beds. Besides, your study does not measure preference (no

paired benthos-diet comparisons), yet the Discussion repeatedly infers a female preference for large blue mussels.

Furthermore, Laursen and Møller (2021), central to your argument, correlate maximum sizes of mussels in gizzards with head volume at Danish wintering sites, but lacks concurrent benthic size distributions and acknowledges possible overestimation of large items; the experimental work they cite shows selection for 10-20 mm mussels due to their higher flesh to shell ratios (Bustnes 1998). Given this, the extrapolation that a widespread "large-prey preference" would drive micronutrient limitation is speculative. Please either provide direct evidence (paired benthos-diet data demonstrating larger-than-available prey) or reframe this section as a hypothesis, and temper claims about generality across consumers, especially for eiders that ingest whole prey and are strongly constrained by shell-processing and digestion rather than energy intake per se (see my previous comment 42 above).

References:

Bustnes JO. 1998. Size selection of blue mussels *Mytilus edulis* by common eiders *Somateria mollissima* in relation to shell content. Can J Zool 76:1787-1790.

Laursen K, Møller AP. 2021. Brain mass explains prey size selection better than beak, gizzard and body size in a benthivorous duck species. PLoS ONE 16:e0248615.

Öst M, Kilpi M. 1998. Blue mussels *Mytilus edulis* in the Baltic : good news for foraging eiders *Somateria mollissima*. Wildl Biol. 4: 81-89.

Response #51: Thank you for your comment. We have amended the paragraph to make it clearer that this section discusses the general implications of recent literature (e.g. Hylander et al., 2024) and our work in the context of prey size selection and thiamine intake (see response #8f). We have clarified that we do not assume general preference for large prey but rather consider consequences in terms of metabolic micronutrient ingestion of consumers that feed on larger prey (see L371-382). Please note that this paragraph does not refer to prey size preference by common eiders.

44. LL. 290-297: I was unable to find a statement in the manuscript that defines a functional yolk-thiamine threshold for detrimental effects. However, in the previous literature, a concentration of ca 5 nmol g^{-1} has been mentioned. If you intend to invoke a biological threshold, please cite it explicitly and justify its species- and context-specific relevance.

Response #52: Our model does indeed predict similar egg thiamine levels to the threshold provided by Reviewer 1 (see added L266-272). However, we are sceptical about drawing bold conclusions from our simplified model regarding the links between prey size consumed and thiamine threshold levels at which detrimental effects occur, for the reasons explained in Response #7e.

45. LL. 298-299. Please consult my comments 21 and 23 (see above) for a motivation why the claim that females successful at nesting prefer large prey is unwarranted.

Response #53: The phrase has been corrected to 'consumption' (L402-403).

46. LL. 299-304: The "order-of-magnitude" comparison appears misleading, at least for the central/northern parts of the Baltic Sea. Local diet reconstructions show weak size selection and a narrow prey-size distribution by female eiders (e.g., faeces 18.2 ± 1.8 mm; intestines 17.4 ± 1.1 mm; matched benthos sample 19.1 ± 4.3 mm; no significant diet-benthos difference) (Öst and Kilpi 1998), and the authors conclude that no unambiguous size-selective feeding occurs in this region. Please either justify this claim with data or rephrase to avoid implying that a 1 mm shift is negligible in this context.

Response #54: This sentence represents the given values in several articles reviewed in the book by Waltho and Coulson 2015 (Table 4.4), including the paper by Öst and Kilpi 1998 (see Response #8d).

47. LL. 304-308: The cited studies (Christie et al. 2020, Baden et al. 2021) concern the Skagerrak-eastern North Sea area and emphasize predation-driven declines in blue mussels; these findings may not be directly applicable to blue mussel dynamics in the Baltic Sea. Please either provide Baltic-specific evidence that low mussel recruitment leads to a local over-representation of large mussels in

eider diets or remove this sentence.
Response #55: Thank you for your comment. In this paragraph of the discussion, we intended to highlight the ecological factors that may be considered risk factors for the common eider population in the North Atlantic and Baltic Sea region in the context of our results. We discuss factors that could lead in the future to thiamine deficiency in areas within common eider species distribution. We have revised the text to avoid this misunderstanding (L409-415).

48. LL. 308-311: This phrasing is confusing: it implies lipid-rich prey items have high thiaminase. Please separate and explain the two distinct mechanisms: (i) thiaminase in prey degrades thiamine before absorption; (ii) high-lipid diets increase thiamine demand and oxidative degradation.
Response #56: Corrected to avoid further confusion (L416).

49. LL. 316-319: This sentence overstates both the causal certainty and the taxonomic/geographic scope of the alleged thiamine-deficiency-induced mass mortality, and hence needs to be rephrased. Thus, of the four citations, three concern salmonids/fish (Bengtsson 1999, Fitzsimons 1999, Mantua 2021) and therefore do not establish that offspring mortality is generally caused by thiamine deficiency across vertebrates. Only Mörner et al. 2017 addresses eiders, and that report describes a single field episode outside the Baltic proper (Swedish west coast), where reduced/failed duckling production could also plausibly arise via other, more general ecological mechanisms (e.g., predation, disease). Furthermore, the wording "mortality episodes in offspring" invites readers to infer that such episodes are established and common in birds, whereas the cited fish literature is not transferable evidence for eider ducklings or other birds alike. Within your own manuscript you also emphasize that predation is a predominant driver of poor recruitment in Baltic eiders, which directly conflicts with the categorical phrasing here.
Response #57: We have modified the sentence to adjust our rhetoric and position it between the sceptics and supporters of the 'thiamine-deficiency induced mass mortality' hypotheses (L430-434). However, we disagree with the scepticism of the Reviewer #1 regarding the support for the link between high episodic mortality of juveniles and thiamine deficiency (see Response #6, #22, #23).

50. LL. 320-322: This statement is not supported by the cited literature for eiders. In Balk et al. (2009), the explicit clutch-size analysis and its correlation with yolk thiamine were shown for herring gulls, whereas the eider evidence concerns low yolk thiamine and thiamine-deficiency biomarkers; there is no eider-specific test linking thiamine status to clutch size. Further, Mörner et al. (2017) report eider thiamine deficiency and long-term nest counts but do not present an analysis of eider clutch size versus thiamine, nor do they exclude alternative mechanisms, most notably partial or complete clutch predation, that can reduce the observed number of eggs (e.g., Öst et al. 2008). Accordingly, please either (i) provide eider-specific empirical evidence demonstrating that thiamine deficiency reduces clutch size independent of predation and other ecological processes, or (ii) rephrase to avoid implying such an association in eiders.
Reference:
Öst M, Wickman M, Matulionis E, Steele B. 2008. Habitat-specific clutch size and cost of incubation in eiders reconsidered. Oecologia 158:205–216.
Response #58: Thank you for spotting this. We have corrected the sentence so that clutch-size effects refer to herring gulls (lines 435-437). We have also added a caveat that this potential effect has to be verified empirically among eiders (line 444-445).

51. LL. 322-323: Please note that the logic underlying the life-history model is inverted (earlier breeding phenology is expected to be associated with an earlier, not later, breeding phenology; see my comment 38 above).
Response #59: Thank you for your comment. As shown in Response #46, we now demonstrate that thiamine levels increase in the event of a reduction in clutch size, regardless of whether this is

associated with advanced or postponed breeding phenology (see Figs 4-5 in S1 Appendix S1) and L323-332.

52. L. 326: However, please note that the use of stopover sites may be very limited in spring-migrating eiders in the Baltic (see my comments 29 and 30 above).
Response #60: see Response #37.

53. LL. 326-329: This sentence is an overstatement of a syndrome that not been confirmed with certainty in eiders, and it further assumes a hypothesized adaptive response by females, which is equally unconfirmed.
First, "early mortality syndrome" (EMS) is a term established for salmonids in the fish literature (e.g., Fitzsimons 1999) and is not an established syndrome in eiders. Please avoid implying that an EMS exists in eiders or that such "years" are documented for the Baltic population. Please use taxon-appropriate terminology and restrict claims to cases demonstrated in the cited taxon.
Second, the manuscript's results show that a ~40% reduction in clutch size in the model can elevate egg thiamine by up to ~20%, but the model does not link thiamine to duckling survival or recruitment, and it does not incorporate alternative mechanisms (e.g.. partial/complete clutch predation, disease) that can reduce observed clutch size. Accordingly, the inference that clutch reduction is an adaptive response that "increases average recruitment" is not supported by the current evidence or model design and should be softened.
Response #61: In the literature, the term 'early mortality syndrome' has been used as a synonym for mass juvenile mortality associated with preceding symptoms characteristic for thiamine deficiency i.e. paralysis, disorientation, impaired locomotion. The sentence has been corrected to position our rhetoric, between the sceptics and supporters of the 'thiamine-deficiency driven mortality' hypothesis (L442-445, see Responses #6, #22, #23).

54. LL. 331: This statement is not entirely correct. In fact, several studies report that female eiders continue feeding intensively until the second or third egg is laid, after which nearly permanent presence at the nest begins (e.g., Hanssen et al. 2002; Andersson and Waldeck 2006).
References:
Andersson M, Waldeck P. 2006. Reproductive tactics under severe egg predation: an eider's dilemma. Oecologia 148:350–355.
Hanssen SA, Engebretsen H, Erikstad KE. 2002. Incubation start and egg size in relation to body reserves in the common eider. Behav Ecol Sociobiol 52:282–288.
Response #62: Corrected according to suggestions (L447-448).

55. LL. 334-336: This sentence makes several unsupported inferences. First, the manuscript does not provide empirical evidence that eider females detect short-term changes in maternal thiamine and plastically reduce clutch size; the results show only that, in the model, fewer eggs could raise per-egg thiamine contents. Second, the proposed "rapid" response overlooks the follicle-recruitment schedule in birds, which constrains seasonal clutch size adjustment and would require data on intra-season thiamine variability and a demonstrated decision pathway**.** Third, "offspring production" conflates eggs laid with post-laying outcomes; a smaller observed clutch size can arise from partial/complete clutch predation or other causes, and do not necessarily imply maternal adjustment. Finally, the cross-taxon contrast with salmon implies a general rule without comparative evidence on timing, plasticity, or fitness consequences.
Response #63: We have rewritten the sentence (L450-454) to clarify that there are great differences in time scales of follicle recruitment and growth in salmon and common eiders. These differences make a potential reduction in clutch size short before eggs release more likely in common eiders than in Atlantic salmon. We disagree with the reviewer's assertion that our statement overlooks the mechanisms of the follicle recruitment schedule. We now cite a review by Haywood (2013) on follicle recruitment mechanisms, which supports our point that the formation of eggs is a very rapid

process in female birds. This allows for a tremendous capability to reduce clutch size shortly before laying (Haywood 2013). Haywood's work (2013) also shows that females can reduce their clutch size in response to various external and internal (physiological) signals. Hence, birds are capable of reducing their clutch size in response to a deterioration in their physiological condition. Please note that insufficient levels of thiamine are expected to reduce metabolic rate, so the reduction in clutch size can be viewed as an unavoidable consequence of thiamine deficiency rather than an effect of the signalling pathway that detects variation in maternal thiamine levels.

Haywood S. 2013. Origin of evolutionary change in avian clutch size. Biological Reviews. 2013 88(4): 895-911.

56. LL. 341-343: The broader claim that "size-specific reduction in concentration is an important factor in the dynamics of essential metabolic micronutrients in consumers" generalizes beyond what was analyzed (one micronutrient, one prey taxon, one consumer modelled). Please moderate this concluding sentence.
Response #64: We have corrected the sentence to better reflect the main justification for our study. We have retained the reference to metabolic micronutrients as the sentence is a part of the Discussion and it describes our expectations due the use of the phrase 'our work suggests that', and that in the introduction we now elaborate on the links between prey body size, metabolic rate, and micronutrient demand (L458-461, cf. Response #14).

I hope you will find these comments constructive and helpful for improving the manuscript!
Response #65: We are grateful to Reviewer #1 for all their tremendous effort and constructive comments that helped us to considerably improve our manuscript!

Reviewer #2: Comments to the Authors:
In this manuscript, the authors rely on a modelling approach to assess how prey size selection affects adult and egg thiamine levels in common eiders. They find that selection for larger prey (here, mussels) leads to lower thiamine concentrations in female eiders and their eggs/ducklings.
While I find the manuscript well-written and I like the modelling approach of female eider life histories, I have some concerns regarding the validity of some of the assumptions made in the models and therefore the results and conclusions of the study (see details in comments below). One of my main issues in the current version of the manuscript is the lack of link between this study and field- or lab-based knowledge of thiamine concentrations in the wild.
Response #66: Thank you for your constructive comments. See our responses below.

Major comments:
1. How reliable are the results of this study? I like the modelling approach of the study and the fact that the authors quantified thiamine levels in mussels at wintering, stopover and breeding locations. However, it is not clear whether concentrations in adults and eggs/ducklings reflect concentrations that would be found in the wild. I believe that some studies have been carried out to monitor thiamine levels in birds (including in eider eggs). It seems crucial that the authors mention these studies (e.g., in the discuss) and compare the thiamine levels in their models to the ones measured in the field. This would greatly improve the reliability of the findings and conclusions of the study.
Response #67: We now present information on thiamine levels in common eiders obtained from the literature. Our model's predictions for various prey sizes are comparable to the reported concentrations (see L266-272). However, as described in Response #7e, we are sceptical about drawing bald conclusions regarding the detrimental effects caused by thiamine deficiency and consumed prey size. Our main objective was to demonstrate the general relationship between the risk of thiamine deficiency and the size of the prey consumed. We would like to avoid drawing bold conclusions based on our simplified model as the model is not powerful enough. In our opinion, the scientific debate on thiamine deficiency-driven mass mortality is currently hindered by both

supporters and sceptics jumping to radical conclusions too quickly (see Response #7e).

2. The title of the manuscript as well as some of the content of the manuscript highlight an interest in assessing how selection for large prey will increase the risk of mass juvenile mortality (mediated by thiamine deficiency). To properly assess or discuss the risk of mass juvenile mortality, it seems important to know the levels at which thiamine is considered to be deficient. At what concentrations are impacts on birds expected (e.g. symptoms of neural dysfunction, impaired behaviour, locomotion…)? Maybe adults or eggs have lower thiamine levels when females prey on bigger mussels, but if levels are high enough it may not be constraining and may not have detrimental consequences. Could egg hatchability and juvenile mortality risk be evaluated in this study?
Response #68: Thank you for your comment. The previous version of the title was incorrect, as our research did not examine the population-level effects of thiamine levels. Please note that Reviewer #1 raised a similar point (see Response #10).

3. Model assumptions. I am not an expert in thiamine and I am wondering what we really know about thiamine concentrations in birds. Is there knowledge of how maternal transfer in the egg works? The model is based on the assumption that "thiamine egg levels reflect the thiamine concentration in the body of the female at laying initiation". How realistic is that? Has there been any study measuring thiamine in females and eggs to validate this assumption? Could thiamine be deposited differently in the first vs the other eggs (as it can be seen in maternal transfer of contaminants for example)? Given that one would expect low levels of thiamine to have an effect on duckling mortality, I wonder what the same levels would have on adult females (if egg thiamine concentration reflects female thiamine concentration then could the same impact be expected on female health)?
Response #69: Most of the knowledge regarding maternal transfer of thiamine to eggs derives from fish models. Recent studies demonstrates that the female muscle and gonad concentrations of thiamine in Atlantic salmon are proportional. We have added the reference to validate this model assumption, and we acknowledged the knowledge gap by pointing out that we lack underlying data for bird models (L235-241).

4. L.70-93. In the introduction, the authors put the emphasis on brain size and its link with predation risk and mussel size preference. While I get the idea, I think that this would be more interesting in the discussion section rather than in the introduction section. Putting the emphasis on prey size preference by itself (without necessarily linking it to brain size) would make the manuscript more attractive to a broader audience – especially because the authors test an effect of prey size preference per se, rather than brain size.
Response #70: Thank you for the comment. In line with Reviewer #2's suggestions, we have rewritten and moved the paragraph to the Discussion section (L383-393). Please note that we have described the link between the size of the prey consumed, brain size and predation avoidance strategies as justification for the aim of our work. However, Reviewer #1 questioned the generality of the finding that common eider females with larger brains select for larger prey. They also questioned the applicability of our model with respect to prey size selection as a phenomenon that would justify our research. Therefore, we have decided to discuss these aspects in the 'Discussion' section and now refer to the allometry of thiamine concentration and metabolic rate with body size instead of using references to the predation of prey size preferences as the main justification for our work (see Response #8b, #14, #15, #48).

5. Results on thiamine mass-specific levels in the soft tissues of blue mussels (l. 218 and after). I think that this section should be improved according to the following points. Could the authors please specify in the methods what statistical tests they used to produce the results. It would be great for the reader to have access to the average thiamine levels in the three areas (for an average mussel mass/size) and know if they statistically differ. Is the decrease in thiamine concentration with mussel

size significant, and does the rate of decrease significantly differ at the three locations?
Response #71: Yes, this is a good idea that helps the reader digest the thiamine concentration results. We have therefore included the statistical methods used when assessing the size-specific thiamine levels in blue mussels (L157-159). We have also included the results from the linear regressions along with estimated marginal means to confirm that size is an important predictor for tissue-specific thiamine concentration but with somewhat different relationships among sites (L281-298).

6. Maybe I missed this information in the methods, but how did the authors model prey size preference? Was each individual modelled as having a preference for a given prey size (e.g., 3cm) and foraging mussels of this exact size or a size following a normal distribution centered around 3cm? I am surprised that in all the figures egg thiamine levels hardly ever overlap between prey size groups.
Response #72: Thank you for your comment. There was indeed a lack of clear description of the modelled scenarios of consumed prey size. We have now included this information in the manuscript (L231-232). Please note that, as our model included aspects of life history and physiology of thiamine, we simplified aspects related to prey size consumption. These simplifications were necessary to keep the model feasible for analysis and understanding. Consequently, our model did not consider behavioural biology aspects related to prey size preference, like the costs of prey searching and handling or the availability of preferred prey. We therefore agree with the points raised by Reviewer #1 on reducing the degree to which we refer to prey size preference in our work (see Responses #8b, #10, #14 and #15).

Minor comments:
• Title: I think that it would be more transparent for readers to mention in the title that this manuscript is based on modelling rather than analysis of empirical data. For example, the mention "simulation-based model" could be added to the title.
Response #73: We combined modelling with empirical analyses and corrected several parts of the abstract to make it clear which results are model predictions (see Responses #17, #18). Therefore, we would advocate for not referring directly to modelling in the title of our work.

• Title: the authors emphasize the risk of mass juvenile mortality but is it really what is assessed in the manuscript (see major comment 2)?
Response #74: Thank you for the comment. This point was raised also by the Reviewer #1, and we agree that the previous version of the title was incorrect, as our research does not focus on the population-level effects of thiamine levels. The title has been changed (see Response #10).

• L. 32-34: I am not sure why the authors draw a specific link with "nest predation". This would mean that either thiamine deficiency has an effect on the behaviour of adult females and makes them less good at protecting their nest from predators, or that thiamine deficiency reduces hatchability of eggs. In precocial species such as eiders, the wording nest predation refers to egg predation and not duckling predation, as they usually leave the nest within 24h. Please make it clearer to the reader.
Response #75: This sentence has been rewritten (L29-33) to link thiamine deficiency with decreased recruitment of common eiders in the Baltic population (see also Response #13).

• L.30-34: Overall I find the reasoning and the transition between the two sentences hard to follow. Could the authors please rewrite them to make it easier for the reader to follow?
Response #76: We have rewritten these sentences in the Abstract to refer directly to the dramatic decrease in the number of common eiders reaching their first year of life (L29-33) (see also Responses #12, #13).

• L. 35: Selection for larger prey may not be entirely driven by brain size. I think that the authors would benefit from making the statement broader as what is important is that some individuals may

select for larger prey and that this choice can potentially lead to thiamine deficiency and have negative impacts on the population. Making the statement more general will make it of interest for a broader audience (also see major comment 4).
Response #77: We have removed this sentence from the Abstract (L36-38, see Response #70).

• L.40: give the scientific name of blue mussels
Response #78: Added (L43-44).

• L. 41: does it reduce the levels in eggs because it reduces the levels in the female? Maybe this is worth mentioning as the authors say l. 37 that they are also interested in adults.
Response #79: Corrected (L44-46).

• L. 44: when reading the abstract, it is hard to understand why the authors find that clutch size reduction increased thiamine levels in the eggs. Could this result only be driven by some constraints of the model (i.e., model assumptions within the modelling framework)?
Response #80: This result is an effect of the optimisation of life-history strategies to maximise fitness. In our model, the concentration of thiamine in the eggs reflected the thiamine status of the mothers when they laid them (see Response #42). The model optimised the timing of breeding and the size of the clutch. Consequently, females that laid fewer eggs were capable of producing offspring with a higher thiamine concentration (see also Response #46).

• L. 44-46: I guess that this is based on assumptions from brain size, which could be clarified as what the results really show is that females selecting larger prey are more likely to produce offspring with insufficient thiamine levels. I think that stating in the abstract that the authors find that females selecting larger prey are more likely to produce offspring with insufficient thiamine levels is what is the most important (for the reader)
Response #81: This sentence has been rewritten because we do no longer refer to the relationship between brain size and antipredator strategies in females as a main justification for our work (see Response #14, #18b, #48) .

• L. 58: To aid readers unfamiliar with thiamine, could the authors briefly explain the mechanism by which thiamine deficiency leads to early mortality? Why do juveniles die if they lack thiamine? Is it because they become more vulnerable to predation or because of impairment of essential functions within the organism (or both)? Can thiamine deficiency induce embryo mortality in the egg as well?
Response #82: We have added a brief explanation that thiamine deficiency can lead to death due to physiological reasons (L65-68).

• L. 66: add the scientific name of common eiders in the main text.
Response #83: Added (L75).

• L. 72: write "one third" instead of 1/3
Response #84: Corrected (L82).

• L. 73: the word "duckling" should be preferred over "nestling" as eiders are precocial (unless the authors want to specifically refer to the stage when they have hatched and have not left the nest yet).
Response #85: Thank you for the comment. In response to the points made by Reviewer #1, the sentence has been corrected and no longer contains the word 'nestling' (L104-106). This term is not used elsewhere in our work either.

• L. 77: the authors mention predation of nests and incubating females, please also add duckling predation (once they have left the nest) as it is definitely very high too.

Response #86: In response to the points made by Reviewer #1, the sentence has been rewritten to better represent the results presented in the literature cited (see Response #26).

• L.91-93: The sentence is misleading, I don't think that the preference for large prey itself is what is adaptive, per se, under increased predation risk.
Response #87: Thank you for the comment. The sentence has been rewritten (L122-125, Response #31).

• L. 99-101: "Our work showed that relatively small shifts in prey size selection towards larger mussels can significantly reduce the thiamine concentration in the produced eggs." this is a result and should be in the result/discussion sections rather than in the introduction.
Response #88: Corrected (L131-134).

• L. 108-109: how were these areas defined? Please note that they are not representative of the entire Baltic Sea eider population and this should be discussed somewhere in the manuscript. Also, please show the exact location of mussel collection in fig 1.B.
Response #89: The sampling locations have been defined within geographic areas that are known to be used by Baltic eiders for wintering, stopovers and breeding. These wide geographic areas have been defined based on literature: e.g. Waltho and Coulson (2015), Laursen et al. (2019). See below Fig. 1 from Laursen et al. 2019 (now cited in the manuscript). We have included a short description of how areas were defined in the material and methods (L142-150). As including the sampling locations in Fig. 1B caused visualisation problems due to the sites being located in areas of the map covered by inset regression plots, we created a separate map showing the locations of the sampling sites (see Fig 1 in S1 Appendix 1). We agree with Reviewer #2 that the sampling points do not cover all environmental variability, particularly in the wintering area which is extensive. However, the amount of material sampled was restricted by our capacity for performing analysis of thiamine content in blue mussels.

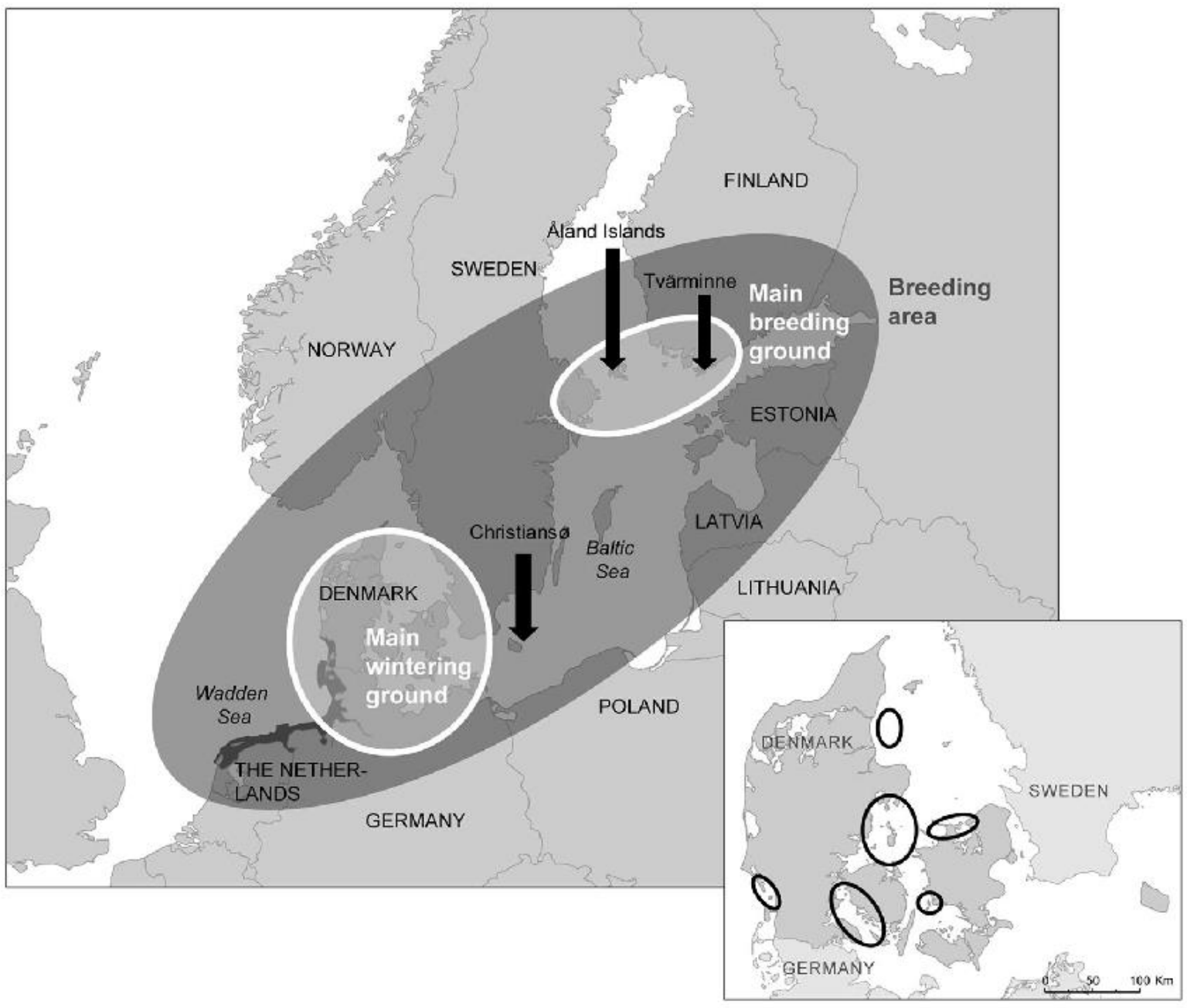

Figure 1. A schematic map showing the breeding area of the Baltic/Wadden Sea eider population. The main breeding area and wintering areas are shown. In addition, the breeding colonies of Christiansø, Åland Islands and Tvärminne are indicated. The inserted map shows the areas where the eiders were collected during autumn and winter in Danish water. The size of the circles indicates the number of eiders collected ($N = 489$).

Laursen, K., Moller, A. P, and K. A. Hobson. 2019. N-Isotopes in Feathers and Abundance of Eiders

Respond to Nutrients in Seawater. Ecosystems 22:1271-1279

• L. 108-109: Based on the appendix, it seems that all mussels were sampled in June. Is it known whether thiamine concentrations show seasonal variation in mussel? Are measurements in June reliable to infer thiamine concentration in the wintering grounds?
Response #90: see L423-429 and Response #49.

• L. 108-109: Based on the appendix, it seems that measurements from different areas are pooled in "wintering" and "stopover". Did the authors account for that when assessing the decrease in thiamine with mussel size?
Response #91: Yes, good comment. We have added more information regarding the sampling sites and the rationale for pooling some nearby sites into the groups in the wintering and stopover areas (L144-150). The main rationale is to maintain high sample sizes with the assumption that birds can move freely between closely located habitats within the wintering and stopover sites.

• L. 119: "We modelled the seasonal thiamine dynamics in common eider females and ducklings at hatching.". What about thiamine dynamics/levels in egg? I am not sure if I have understood how thiamine levels are expected to vary between egg laying and hatching. Is it assumed to be constant from egg laying to hatching?
Response #92: Thank you for your comment. As stated in the abstract and elsewhere in the manuscript, our work investigates egg thiamine levels, and we have corrected the text mentioned by the reviewer for clarification (L162-163). We did not model changes in thiamine concentration in the eggs during incubation, as this would require us to make several assumptions about thiamine breakdown rates related to the growth process during incubation.

• L. 121 or l. 153: before laying, eiders can feed both in the wintering, stopover and breeding areas. Does the model allow females to feed on the breeding grounds?
Response #93: Yes. Clarified (L225).

• L. 132: what do we know about maternal transfer of thiamine (from the female to her eggs)? I think that this is something important to consider. Do all eggs produced by a given female have the same thiamine concentration?
Response #94: Yes, we agree. This is an important factor (See response #69 and L235-241).

• L. 144: how did the authors define this stopover location? Do they rely on empirical data from a monitored eider population? If so, this would be important to mention in the manuscript, especially because the duration of spring migration stopovers can be very short in eiders in the Baltic Sea and I don t think that this area is a well-known stopover area for the entire Baltic Sea eider population.
Response #95: We now briefly describe in the manuscript how the sampling locations at the wintering, stopover and breeding grounds were defined (L142-144, Response #89). Although our study was inspired by the life history of Baltic eiders, we aimed to create a model that could address eider populations migrating at short or relatively long distances, with or without prolonged stopovers, to increase the generality of our findings. Please note that these differences in migration tactic did not affect the conclusions of our study: feeding on large prey decreased thiamine concentration in the eggs, regardless of the time spent on stopovers (see Response #37 and Figs 5, 7 in S1 Appendix).

• L. 149: is that for the arrival at the wintering location?
Response #96: See added L194-196 and Response #38.

• L. 154: clutch size typically varies between 3 and 6 eggs in eiders in the Baltic Sea, why did the authors limit it to a maximum of five?

Response #97: Thank you for your comment. We acknowledge Reviewer #2's point and have added an additional figure to the S1 Appendix showing that our conclusions did not change when the maximal clutch size was set to 6 (see Fig. 9 in the S1 Appendix) and mention that in the main text (L201-202).

We assumed the maximum clutch size of 5 in accordance with the literature. According to Waltho and Coulson (2015), clutch size in common Baltic eiders monitored over several years in nesting areas in Finland rarely exceeds five eggs per nest (see Figure 8.3 from Waltho and Coulson 2015 below). In some areas where common eiders breed in dense colonies, egg dumping is a common strategy that can increase the number of eggs per nest (see for example, Ejsmond et al. 2021, Ejsmond and Ejsmond 2022). This phenomenon was not modelled in our work, as it would only increase the complexity of the model without direct link to thiamine levels.

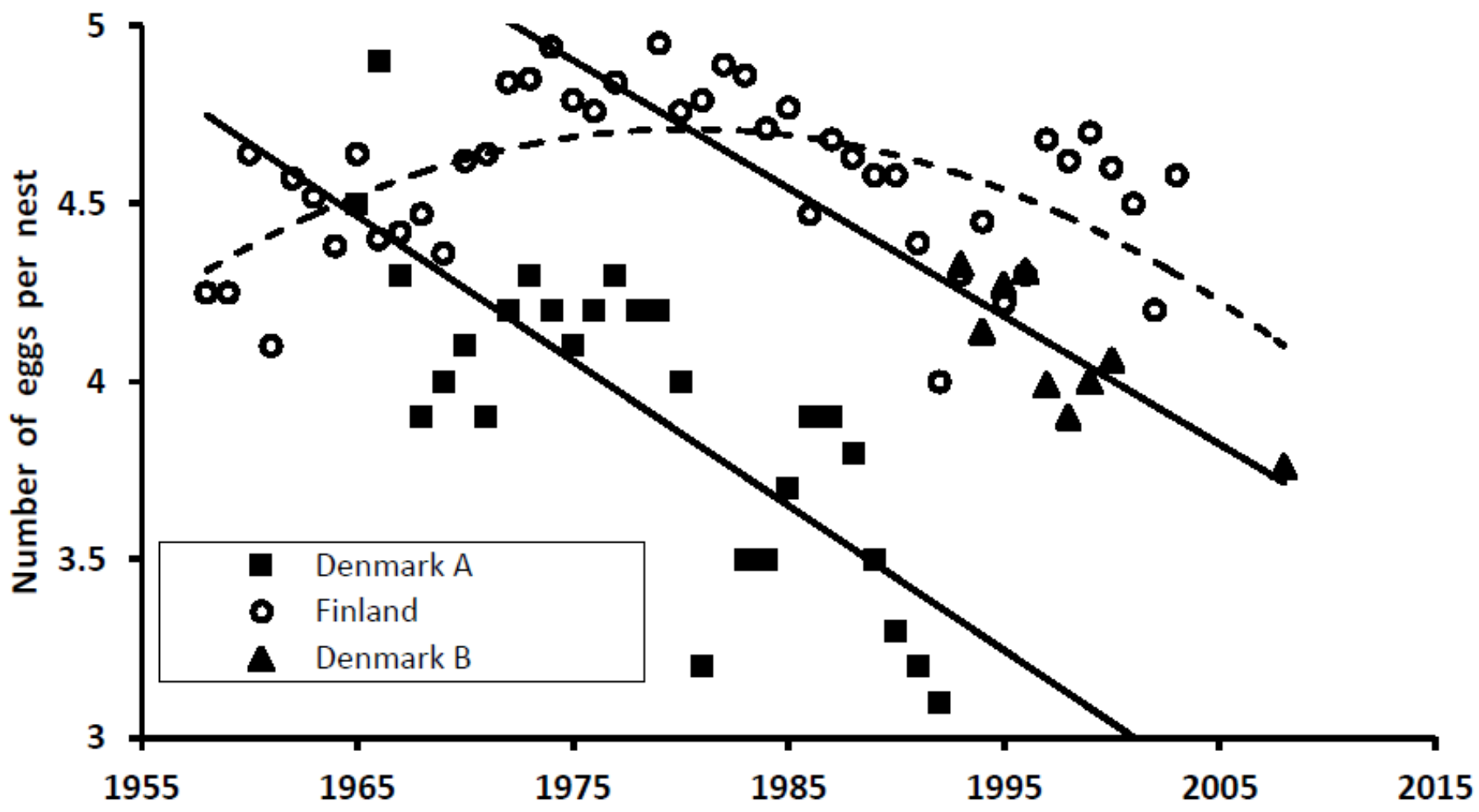

Figure 8.3. *The annual and long-term trend in the number of Common Eider eggs per nest on a nesting area in Finland (circles and dashed trend line) and at two sites in Denmark (solid squares and triangles and continuous trend lines). Average values for individual years shown by symbols. All three locations show a decline in recent years.*

• L. 225: "The life history of the model females resembled the life cycle of the Baltic common eiders, with accumulation of body reserves preceding migration, egg laying and incubation (Fig 2A)." Do the authors have access to empirical data to support this statement?

Response #98: In our work, we used the literature to parameterise our model with respect to the life history and phenology of the common eider in the Baltic Sea cited in the manuscript and S1 Appendix. Our model was designed to simulate migratory populations of common eiders, as Baltic population. However, we had to simplify the model's structure with regard to many life history aspects in order to incorporate thiamine consumption while maintaining the model's feasibility for analysis. We agree that some aspects of the life history and migration of Baltic eiders can be variable and our model did not cover all of these aspects. However, we now address all discrepancies pointed out by reviewers and demonstrate that when these aspects are included the conclusions of our study do not change. These aspects are the following:

- arrival of females on breeding grounds in a lean body condition, and the degree of capital breeding in Baltic eiders (Responses #33, #36)
- limited usage of stopovers during spring migration in Baltic eiders (Responses #37, #95)
- feeding during early laying period (Response #39)
- the clutch size decrease with postponement of breeding (Responses #38, #46)
- rapidly intensifying predation (notably by white-tailed eagles) (see last paragraph in Response #46).

• L.252-254: I think that the paper would benefit from introducing optimal foraging theory in the introduction.
Response #99: In response to Reviewer #1's comments regarding the generality of Laursen and Moller's (2021) findings and the relevance of these results in justifying our work, aspects of prey-size selection and optimal foraging are now mentioned only in the Discussion section. Please see Responses #8b, #14, #15, #48 and #70 for details.

• L. 294: The sentence seems incomplete, please check and correct it.
Response #100: Corrected (L398-399).

• L. 316: when referring to "mortality episodes in offspring" caused by thiamine deficiency in this sentence, are the authors still referring to ducklings or all species? Please clarify.
Response #101: Clarified (L430-434).

Reviewer #3: Review of PONE-D-25-50326. Selection for large prey increases risk of mass juvenile mortality due to thiamine deficiency

General comments

This is a very interesting manuscript. I was not aware of the severity of the thiamine deficiency issue or the reports of mass mortality of common eiders in the Baltic population. This paper provides an important new contribution by assessing how thiamine deficiencies could arise through prey size selection of blue mussels by common eiders. The authors show that there is a strong pattern of thiamine tissue concentration with respect to mussel size and this could have important ramifications not only for diet selection, but for thiamine deficiencies in eggs and ducklings that could increase juvenile mortality and contribute to the long-term decline of these populations.

The paper is based on analysis of thiamine tissue concentrations from a collection of mussels from the wintering, stopover, and breeding grounds, as well as a comprehensive modeling analysis using dynamic programming to derive optimal life histories and then using that to predict how prey size selection could impact survival and reproductive success in common eiders. I thought this was a novel approach and while it is based on predictive modeling and assumes that eiders do indeed follow optimal life histories (what if they don't?), it clearly illustrates how diet selection could significantly impact juvenile mortality, and might have the greatest effect on larger females that would be otherwise expected to reproduce well.
I have only a few comments/suggestions. I was a bit confused because the paper describes large eiders as selecting larger mussels (lines 85-93), which makes sense (perhaps based on brain size), and one would expect that larger mussels provide a greater energetic value overall. Hence, the concern is that if selection for larger mussels reduces the amount of thiamine that females acquire and provide to their eggs, this could have negative consequences. However, later in the manuscript, the authors note that in experimental studies, common eiders typically select the smallest class of mussels (line2 70-271). So, I am a bit unclear about which direction is expect for prey size selection – larger or smaller? I could use a bit more clarification.
Response #102: In light of the criticism from Reviewer #1 and the suggestions from Reviewer #2, we have decided to abandon our primary justification for the work, which was based on the relationship between prey size preferences and brain size (see Responses #14, #15 and #70). Instead, we now refer to the well-documented relationship between body size, thiamine content, and metabolic rate (see Responses #14 and #48).

Following on this point, I can see how there might be selection for large mussel size based purely on

energy content, and larger females (with larger brains) might be better able to capture the larger mussels. And I can also see how that, in turn, could directly impact clutch size simply because of the greater energy and nutrient content available for making more eggs. The energetic pathway is clear and well established.
It's less clear to me how eiders would directly select for thiamine content (i.e. selecting mussel sizes for their thiamine content), and how that would directly or physiologically affect clutch size. Is there a direct physiological link between thiamine concentration and the production of eggs? Could it simply be that eiders that do select for small mussels are less able (energetically) to produce as many eggs (and so clutch sizes are smaller), but the unintended (and perhaps unselected for) consequence is that thiamine concentration is actually higher and so those birds end up doing better in terms of egg/juvenile survival. A sort of hidden physiological reversal of fortunes –> smaller prey size (but greater thiamine concentration) -> less energy -> smaller clutch sizes -> greater juvenile survival (due to higher thiamine concentration). The opposite would obtain for larger females selecting larger prey.
I understand that this is what the authors are describing. My point was simply that I found it hard to imagine how eiders might be actively adjusting selection of prey sizes based on thiamine concentration – what is the mechanism by how they would assess that or how that would directly influence the production of eggs. OK – just some musings.
Response #103: Thank you for your comment. In our opinion, selecting prey size according to thiamine content in food is unlikely to be driven by a direct mechanism, though selecting smaller mussels would indeed indirectly increase thiamine intake (see Response #50). We are not aware of any direct link between egg production and maternal thiamine levels. However, insufficient levels of thiamine are expected to reduce the metabolic rate, which is expected to mechanistically decrease the capability of females to produce eggs (see also response #63 regarding the relationship between clutch size and maternal thiamine levels).

I wondered what the outcome of the model would be in females did not follow the optimal life history derived from the dynamic state-variable model? Might thiamine deficiency also influence the timing of breeding or seasonal resource allocation by females such the impacts are even greater or different? Perhaps a comment on this in the discussion. It might also be useful to consider any other uncertainties or assumptions of the model that might impact the outcomes (perhaps as avenues for future work).
Response #104: Great comment! This question is actually about including maternal tissue thiamine levels as a factor in fitness optimisation of life history strategies. Such a modelling approach would likely require merging the presented model with one concerning the allocation of thiamine to somatic tissues (impacting adult survival) and reproductive tissues (impacting offspring recruitment). To our knowledge, our recent work on Atlantic salmon is the only study to investigate the fitness consequences of optimising micronutrient allocation between tissues (see Ejsmond et al. 2025). Unfortunately, this model based solely on thiamine concentrations have already predicted complex, non-linear temporal dynamics of thiamine allocation between gonads and muscles (Ejsmond et al. 2025). Therefore, merging this two types of models would likely result in a highly complex model structure difficult for analysis and understanding. We believe that further studies on the optimal allocation of micronutrients between tissues are needed before attempting to merge the approaches of fitness optimisation focused on resources (energy) and micronutrients.

Ejsmond MJ, Todisco V, Hauber MM, Hindar K, Hylander S. 2025. Physiological Mechanisms and Life History Trade-Offs in Salmonids Shape In-Tissue Correlations of an Essential Micronutrient. Ecology and Evolution 15(10):e72339

Fig 1 shows the range of mussel sizes in each location, but it might also be useful to add a plot of the overall frequency distribution of mussel sizes in the samples collected. It appears from the scatterplots in Fig. 1B that most mussels were <3 cm and many <2cm. So, an eider simply foraging at random

might choose mussels that are in the higher range of thiamine concentrations than had they selected mussels >3 cm (Figure 2C).
Response #105: We now present the frequency distributions of sampled mussels requested by the Reviewer (see Fig 2 in S1 Appendix 1, referred to in L145, 146-147, 150).

I am not a physiologist, nor do I know very much about thiamine accumulation, metabolism, reabsorption ,or excretion, and it would be worthwhile to have a physiologist evaluate the rates of thiamine intake and loss that were used in the model. However, to my uninformed eye, the analyses and assumptions made appear thorough, well-supported and thoughtful.
I have a few other smaller line-item comments below, but overall, this is a valuable and important contribution. I hope my comments are useful.
Response #106: Thank you for appreciating our work and thank you for all your comments.

Specific comments

Lines 86-87. Is this just a function of larger brain size or overall larger body size as well? Did the Laursen and Moller 2021 paper control for body size?
Response #107: The sentence has been rewritten, and the paragraph has been moved to the Discussion section (see Responses #70, #14).

Lines 113. Did each sample comprise mussels of varying sizes? Could you add another plot summarizing the frequency distribution of mussel sizes samples from each location (perhaps not necessary since this can be inferred from the scatterplots in Fig. 1B but a summary frequency distribution would be helpful.).Could differences in the availability of mussel size classes among locations or seasons also impact diet patterns and hence prey size selection and thiamine concentrations in the diet?
Response #108: We now present the frequency distribution plot requested by Reviewer #3 (see Fig 2 in S1 Appendix 1 and Response #105). Regrading the degree to which we now refer to prey size selection in our work - please see Responses #14, #15, #31.

Lines 128-129. This is very reasonable but what if all or some females did not follow the optimal life history derived from the dynamic state-variable model? As noted above, might thiamine deficiency also influence the timing of breeding or resource allocation by females such the impacts of thiamine deficiency are even greater, or different?
Response #109: See Response #104.

Lines 221-224. Why the differences in the R2 values among locations/season? Thoughts?
Response #110: In response to Reviewer #2's suggestions that a more detailed statistical analysis of the empirical results is needed (see Response #71) we now present a more detailed statistical analysis of sampled blue mussels (L281-298).

Lines 238. I wonder if a small table listing the parameter, their symbols and brief description could be included in the text. It would help a reader as one reads through the results on the effects of different parameter values and rate coefficients.
Response #111: We now present table showing key model parameters (see Table 1 in S1 Appendix).

Lines line 270-277 and . This is where I got a bit hung up on whether eiders generally select for larger or smaller mussels. And if they do select for smaller mussels, how would they assess thiamine content and how would that directly impact the number of eggs they lay. I can see how females might select prey based on handling and search times and energetic content, and that would directly translate into

the energy available to produce eggs clutch sizes. I have a less clear understanding how females could select for thiamine content other than as an incidental effect of selecting for small sizes (perhaps for other reasons) and how that physiologically might limit clutch size. Maybe a bit more discussion on this idea.

Response #112: This paragraph has been removed (L357-367) in light of the comments made by Reviewer #1, as well as the risk of readers misinterpreting some elements of the discussion (see Response #50).

Line 377. The lines for the two alternative scenarios of offspring value (solid blue and dotted blue) in Fig. 1C are hard to see against the green background. Maybe just use black lines?

Response #113: We have changed to dark red lines, i.e. lines with a more contrasting colour than the blue previously used (see Fig 1C).

---

6. PLOS authors have the option to publish the peer review history of their article (what does this mean?). If published, this will include your full peer review and any attached files.

If you choose "no", your identity will remain anonymous but your review may still be made public.

Do you want your identity to be public for this peer review? For information about this choice, including consent withdrawal, please see our Privacy Policy.

Reviewer #1: No

Reviewer #2: No

Reviewer #3: **Yes:** John M. Eadie

[NOTE: If reviewer comments were submitted as an attachment file, they will be attached to this email and accessible via the submission site. Please log into your account, locate the manuscript record, and check for the action link "View Attachments". If this link does not appear, there are no attachment files.]

To ensure your figures meet our technical requirements, please review our figure guidelines: https://journals.plos.org/plosone/s/figures

You may also use PLOS's free figure tool, NAAS, to help you prepare publication quality figures: https://journals.plos.org/plosone/s/figures#loc-tools-for-figure-preparation.

NAAS will assess whether your figures meet our technical requirements by comparing each figure against our figure specifications.

In compliance with data protection regulations, you may request that we remove your personal registration details at any time. (Remove my information/details). Please contact the publication office if you have any questions.
