# Supplementary material for: The risk of thiamine deficiency in common eider ducklings increases when females consume large prey
Source: PLoS One. 2026 Sep 16;21(9):e0356777. doi: 10.1371/journal.pone.0356777 (PMC13580947; doi:10.1371/journal.pone.0356777)
Supplement: Supplementary file 6 [file pone.0356777.s006.pdf]

Reviewer Comments to Authors

Manuscript ID: PONE-D-25-50326R1

The risk of thiamine deficiency in common eider ducklings increases when females consume large prey

by Maciej Jan Ejsmond, Anna Ejsmond, Marc M. Hauber, Jón Einar Jónsson, Andreas M. Waser & Samuel Hylander

This revised manuscript combines empirical measurements of thiamine concentrations in Baltic blue mussels with a life-history model of female common eiders to examine whether consumption of larger mussels could reduce maternal and egg thiamine levels.

The manuscript has clearly undergone substantial revision, and several changes have improved readability and transparency. The authors have tempered some of the strongest formulations, added new sensitivity analyses in S1 Appendix (including alternative calculations of thiamine, stochastic food intake, migration-related assumptions, and maximal clutch size), provided some additional statistical output for the mussel thiamine-size analysis, and acknowledged more explicitly that predation is a major and probably dominant driver of the decline of Baltic eiders. I also appreciate that the revised Discussion now at least recognizes the potential seasonal dependence of the mussel size-thiamine relationship (e.g., due to variable reproductive state of mussels), and that some points raised by Reviewer #2, such as the maximal clutch size assumption and the transparency of the statistical results, have been addressed to some extent. These are important and meaningful improvements.

I recognize that my earlier review may at times have sounded severe, reflecting my continued skepticism toward the thiamine-deficiency hypothesis in this system. That was not intended unfairly, and I appreciate the substantial effort the authors have made in revising the manuscript. Nevertheless, I remain disappointed that several of the core concerns were either only partly addressed, treated superficially, or discussed so vaguely that readers may still struggle to understand their full implications.

The following issues are of major concern:

1) Framing and hierarchy of evidence about thiamine deficiency remain too strong.

Although the rhetoric is softer than in the previous version, the revised manuscript still often presents thiamine deficiency as if it were a well-established major driver of Baltic eider population decline. In my reading, the hierarchy of evidence remains the reverse: increased predation pressure on breeding females is presented in the manuscript itself as the best-supported candidate driver of recent Baltic declines, whereas any additional contribution of thiamine deficiency remains uncertain. This distinction is not reflected clearly enough in the Abstract, opening Introduction, or several parts of the Discussion. Likewise, statements about recruitment failure, adaptive clutch size reduction, and “years with thiamine deficiency” still go beyond the actual evidence base.

2) The mechanistic interpretation of the mussel size-thiamine relationship remains stronger than the evidence supports.

The negative association between mussel size and thiamine concentration is one of the most interesting empirical findings of the study, but its cause is still not resolved. The manuscript still leans strongly toward a metabolic/allometric explanation while alternative mechanisms, especially size-dependent reproductive status of mussels sampled during June, remain incompletely addressed. The new text now mentions that reproductive state may play a role, but still does not explain the expected direction clearly enough for readers. More generally, the paper continues to move too quickly from an interesting observed pattern to a broader mechanistic claim about prey size and micronutrient dynamics in consumers.

3) The biological realism and transparency of the life-history model remain insufficiently improved.

Several core modelling assumptions still appear weakly justified biologically, incompletely explained, or in tension with empirical knowledge of Baltic common eiders. These include the treatment of stopovers, the assumption that females with high reserves arrive essentially lean, the imposed mortality cost of reserves, the handling of breeding phenology and clutch size responses, the starvation threshold, the baseline maximal clutch size, and the treatment of thiamine allocation relative to body fat reserves. The added sensitivity analyses are helpful, but in several cases they show only that the qualitative sign of the effect is retained, not that the baseline assumptions are biologically fully convincing.

4) Statistical reporting remains too dispersed.

Reviewer #2 was right to request clearer statistical reporting for the mussel thiamine-size analysis. The revised manuscript now reports more statistical output, but the setup of these analyses is not transparent enough because details are dispersed across Methods, Results, S1 Appendix, and response text. In particular, the underlying fitted model for the empirical mussel analysis is still not described as transparently as it should be, and the rationale for the estimated marginal means contrasts remains insufficiently explained. A short dedicated Statistical analysis section would substantially improve transparency.

5) The Discussion still overextends inferences about prey size selection and misuses some cited empirical literature.

The revised Discussion continues to overinterpret what the present study actually shows about prey size choice and consumer behaviour. The manuscript does not measure prey size preference, foraging costs, or energetic profitability, yet still discusses “large-prey preference”, diversification of diets or tactics, and generalized trade-offs as if these followed directly from the current data and model. In the focal eider-mussel system, more straightforward explanations based on flesh-to-shell ratios and shell-processing profitability remain at least as plausible. In addition, some cited literature is still not represented accurately enough, especially with respect to Hylander et al. (2024), Öst and Jaatinen (2015), Jaatinen et al. (2019), and Laursen & Møller (2021).

Here are my more specific comments, questions and suggested changes to the manuscript:

1. Abstract, LL. 29-48: The Abstract has been improved stylistically, but it still tends to present thiamine deficiency as more strongly established than the manuscript itself justifies. In particular, the present wording still does not make sufficiently clear that predation is the best-supported driver of the Baltic eider decline, whereas any contribution of thiamine deficiency remains uncertain (see Major comment 1 above).

2. Abstract, L. 29: may constitute a threat.

3. Abstract, LL. 31-34: This sentence is still misleading because it highlights a possible link between egg thiamine and the drastic reduction in recruitment without informing the reader that, in the manuscript itself, increased predation pressure is presented as the main driver of the population decline, whereas the contribution of thiamine deficiency remains uncertain. Accordingly, the Abstract should be rephrased to reflect that hierarchy of evidence more explicitly.

4. Abstract, LL. 34-36: The new body-size/thiamine motivation remains ecologically incomplete. The phrase "thiamine concentration decreases with the size of organisms at the interspecific level" in the Abstract does not clearly motivate the actual question being asked, which concerns size variation within a single prey species, occupying the same trophic level. As written, the text jumps from an across-species allometric pattern to an intraspecific hypothesis about blue mussels without explaining that inferential step. Moreover, this framing does not fit especially well with Baltic ecology: blue mussels have declined in size over time at least in the northerns parts (e.g., Åkermark et al. 2022; see manuscript for reference), and future warmer, less saline conditions are expected primarily to reduce mussel viability and abundance rather than favour larger mussels (Jaatinen et al. 2021). Thus, the broader interspecific relationship between body size and thiamine content should be presented only as a general rationale for testing whether a similar pattern occurs within blue mussels, not as implying that ongoing Baltic environmental change would strengthen the proposed prey size/thiamine mechanism. If anything, the opposite may be expected, with this particular risk potentially decreasing in importance if mussel size distributions continue to shift toward smaller individuals.

Reference:

Jaatinen K, Westerbom M, Norkko A, Mustonen O, Koons DN. 2021. Detrimental impacts of climate change may be exacerbated by density-dependent population regulation in blue mussels. *Journal of Animal Ecology*, *90*(3), 562–573.

5. Abstract, LL. 40-47: As written, this part still implies that females actively reduce clutch size in response to poor thiamine status to improve offspring quality. However, in the manuscript, clutch size reduction is associated with adjustments of breeding phenology/feeding constraints rather than from thiamine input itself, and the manuscript does not exclude alternative ecological causes of reduced observed clutch size, especially predation (e.g., Öst et al. 2008).

Reference:

Öst M, Wickman M, Matulionis E, Steele B. 2008. Habitat-specific clutch size and cost of incubation in eiders reconsidered. Oecologia 158:205–216.

6. Keywords, LL. 50-52: The keyword “high juvenile mortality” remains misleading. It is rhetorically loaded, and the manuscript does not provide juvenile mortality data; it provides prey-thiamine measurements and modelled consequences for female and egg thiamine levels. Please replace this with a more neutral and directly relevant keyword.

7. Introduction, LL. 56-58: This opening sentence stating that thiamine-deficiency-driven juvenile mortality constitutes a serious conservation problem “affecting ecosystems worldwide” is too broad. Rather, the evidence summarized here appears limited to a number of aquatic systems and mainly observed in top consumers such as salmonids, while the relevance to Baltic common eiders is presented later in the manuscript as uncertain and potentially subordinate to predation. I therefore recommend tempering this sentence and aligning it more closely with the actual taxonomic and geographic scope of the evidence cited.

8. Introduction, LL. 66-70: I appreciate that this revised sentence is rhetorically softer than the original, but it still sidesteps rather than resolves my earlier concern. By stating that juvenile mortality episodes in Baltic common eiders “raise concerns” about thiamine deficiency in benthic food webs, the text still implicitly hints at a causal connection between eider juvenile mortality and thiamine deficiency, despite the fact that the manuscript itself later states that increasing predation pressure has been suggested as the main cause of the population decline and that any contribution of thiamine deficiency remains unclear. I therefore recommend rewriting this passage so that the hierarchy of evidence is presented immediately and explicitly, rather than first emphasizing thiamine and only later acknowledging predation as the dominant explanation.

9. Introduction, L. 68 and again in the Discussion: “Top consumer” seems an imprecise label for common eiders. Because eiders are themselves preyed upon, the term may overstate their trophic position. A more accurate label would be e.g. “benthic feeder” or “molluscivore”.

10. Introduction, LL. 82-97: I scrutinized the paper by Hylander et al. (2024), and I remain unconvinced that it supports the arguments presented here. That paper demonstrates trophic dilution of thiamine across a pelagic food web, i.e., declining concentrations from phytoplankton to zooplankton to planktivorous and piscivorous fish, and discusses this pattern in terms of food web transfer, transfer efficiency, and community composition. It does not demonstrate that body size *per se* is the causal driver of reduced thiamine concentrations, nor does it provide direct support for extrapolating such a pattern to size variation within a single prey species such as blue mussels, all of which occupy the same trophic level. The current wording therefore conflates trophic position with body size. If the authors wish to motivate a size effect on thiamine levels in mussels, that rationale should be presented separately, rather than as if it would follow directly from the trophic-level analysis in Hylander et al. (2024). The rationale based on allometry between body size and metabolic rate is clearly preferable here, but it remains a hypothesis-generating framework rather than a demonstrated mechanism.

11. Introduction, LL. 99-109: The wording “Here we test whether…” is potentially misleading. The manuscript does not empirically test thiamine levels in common eider ducklings; rather, it measures thiamine concentrations in blue mussels of different sizes and then uses a life-history model to predict consequences for female and egg thiamine status. Please rephrase this

sentence to distinguish clearly between the empirical mussel analysis and the model-based inference about eider eggs and offspring.

12. Material and Methods, LL. 114-123: The treatment of stopovers remains too categorical. While stopovers can occur in spring-migrating common eiders, published evidence and the general migration pattern of Baltic common eiders suggest that spring migration is often rapid and highly synchronized (Alerstam et al. 1974; see my previous reviewer report for reference), with some females moving north without prolonged staging. I would therefore recommend softer wording, e.g., “potential stopover areas”, unless the authors can provide direct evidence that spring staging in these locations is frequent and typical for Baltic female common eiders.

13. Material and Methods, L. 132: The statistical description is imprecise here. Because site is a categorical factor whereas mussel size is a continuous covariate, this analysis is more appropriately described as a general linear model/ANCOVA (including site, length, and their interaction) rather than simple “linear regression”.

14. Outline, LL. 137-149: The phrase “during the pre-breeding period” suggests that reserve accumulation occurs before breeding begins. That seems too restrictive, given that females often continue feeding intensively into the early laying period (e.g., Hanssen et al. 2002; Andersson and Waldeck 2006; see my comment #62 in the response letter).

15. Life history and migration assumptions, LL. 164–167: I remain unconvinced by the assumption that females with high reserves would reach the breeding grounds “close to or in a lean body condition”. Although the authors now note that alternative high-arrival-reserve scenarios did not qualitatively change the direction of the prey-size effect, that does not remove the need to justify the baseline assumption biologically. In its present form, the model still seems to suppress spring carry-over capital more strongly than current evidence of mixed capital-breeding strategies (Sénéchal et al. 2011, Hobson et al. 2015, Jaatinen et al. 2016; see my previous reviewer report for references) would suggest.

16. LL. 137–139, 164–167: Relatedly, there is an element of internal inconsistency here. The text states that reserve accumulation precedes migration, egg laying and incubation, yet Appendix S1 simultaneously assumes that females departing with maximal reserves arrive at the breeding grounds essentially lean. This can only be reconciled if reserve accumulation before reproduction is assumed to occur mainly after arrival rather than being carried through migration. If so, this should be stated explicitly; otherwise the current wording misleadingly suggests substantial spring carry-over capital, even though the model largely suppresses it.

17. LL. 165–167; see also Fig. 5 in S1 Appendix (migration-cost sensitivity): I checked Fig. 5 in the S1 Appendix, and the statement that the “conclusions did not change” is too vague. What appears unchanged is primarily the qualitative ranking that larger consumed prey leads to lower thiamine levels. That does not demonstrate that the baseline migration assumption is biologically realistic, only that the sign of the effect is robust.

18. Clutch size parameterization, LL. 171–173: I appreciate that the authors now show, in response to Reviewer #2, that the main conclusions are qualitatively similar when maximal clutch size is set to 6. However, the choice of 5 as the biological default still appears restrictive. In Baltic common eiders, clutches of six eggs are not exceptional, and direct evidence exists that a single female may lay up to seven eggs in the same nest (Waldeck et al. 2004). I therefore

suggest either using 6 as the biologically more realistic default upper bound, or justifying more clearly why 5 was retained as the baseline despite the broader observed range.

Reference:

Waldeck P, Kilpi M, Öst M, Andersson M. 2004. Brood parasitism in a population of common eider (*Somateria mollissima*). Behaviour 141:725–739.

19. Timing of nesting / breeding phenology, LL. 208–213: The phrase "timing of nesting" is ambiguous. Based on my reading, it appears that this variable may refer to laying dates rather than hatching dates. If so, the assumptions and wording should be made more explicit. Likewise, the current causal chain linking winter conditions, arrival timing, reserve accumulation, and clutch size remains difficult to follow in places and should be explained more transparently.

20. LL. 214–220; see also S1 Appendix, section 2.3: I can see from the Appendix code that incubation survival is set as p_incub = p_rest^5, but I could not identify a direct empirical reference justifying the exponent 5 itself. Please clarify whether this multiplier is based on data, a scaling assumption, or model convenience.

21. Mortality cost of reserves, LL. 216–220: I remain unconvinced by the assumption that survival declines as body reserves increase. This is not a minor modelling detail: the Appendix code imposes an explicit exponential mortality cost of reserves, thereby shaping the optimal strategy and subsequent thiamine trajectories. Yet the empirical literature points in the opposite direction. In the Baltic population, Ekroos et al. (2012) found that female body condition at the first documented breeding attempt, specifically chosen to avoid confounding with age, was positively associated with annual apparent survival. Likewise, Laursen et al. (2019; see previous reviewer report) present high female body condition as beneficial for breeding performance, including brood size, survival of young, and hatching success. This empirical evidence of a positive relationship between body condition and survival is also in line with theoretical expectations, such as the asset protection principle (Clark 1994; see previous report) or the state-dependent safety hypothesis (e.g., Luttbeg and Sih 2010; see previous report). The authors now show some sensitivity analyses, but the biological basis for the baseline reserve-survival penalty still seems insufficiently justified.

Reference:

Ekroos J, Öst M, Karell P, Jaatinen K, Kilpi M. 2012. Philopatric predisposition to predation-induced ecological traps: Habitat-dependent mortality of breeding eiders. *Oecologia*, *170*(4), 979–986.

22. Thresholds and reserve parameters, LL. 219–220; see also S1 Appendix, sections 2.1–2.3: I checked both the S1 Appendix and the code, and I could not locate any transparent derivation of the threshold Sr = 100 g. It appears to me that this threshold applies to wintering/stopover survival, not to breeding initiation on the breeding grounds. Please clarify the biological meaning of this parameter and where it comes from.

23. LL. 223–227: Please specify the time unit, i.e. that h = 37.5 is a daily reserve use rate (g day^{-1}).

24. Mussel intake and reserve gain, LL. 223–231: The assumed conversion between daily mussel intake and reserve gain may not be generically applicable across geographical areas. In particular, Baltic blue mussels in the less saline northern Baltic Sea have a considerably higher flesh proportion and a lower shell mass than Atlantic mussels. E.g., Öst and Kilpi (1998) showed that the amount of shell an eider must ingest to meet its daily energy needs is much lower in their northern Baltic study area than in Atlantic settings. I therefore doubt that the generic conversion of reserve gain to 1.9 kg of mussels per day is appropriate for all modelled sites. Please discuss this limitation more clearly

Reference:

Öst M, Kilpi M. 1998. Blue mussels *Mytilus edulis* in the Baltic: good news for foraging eiders *Somateria mollissima*. Wildlife Biology 4:81-89.

25. Tentative threshold values, LL. 266–272: The motivation for using the egg-thiamine threshold has improved relative to the previous version because the manuscript now explicitly cites the putative threshold and correctly frames it as tentative. Nevertheless, I do not think the threshold can carry the amount of interpretive weight the manuscript occasionally places on it. From a logical perspective, the fact that model-predicted egg thiamine values fall both below and above a tentative threshold does not demonstrate that the model is “not flawed” with respect to inference about offspring quality.

26. Thiamine allocation and fat reserves, LL. 273–278: I could not locate a reference in the manuscript or S1 Appendix supporting the statement that thiamine “is not allocated to body fat reserves”. Is this a modelling assumption or an empirically documented fact? Please either provide an appropriate reference or make it explicit that this is an assumption. I appreciate that the authors now include a sensitivity analysis of this point, but that does not remove the need to justify biologically why the main formulation is preferable.

27. Statistical transparency, LL. 129–132 and 281–298: I think the manuscript would benefit from a short, separate Statistical analysis section. While the current structure is not wrong, the statistical details are so dispersed that the setup of analyses becomes rather hard to follow, especially because the paper combines empirical mussel analyses with model-based simulations and sensitivity analyses. A dedicated statistical analysis subsection would substantially improve transparency. In this respect I agree with Reviewer #2.

28. Empirical mussel analysis/EMMs, LL. 281–298: In my view, listing the pairwise EMM results alone does not allow the reader to assess whether these *post hoc* tests were appropriately specified. Please clarify the underlying fitted model, whether the interaction between site and length was retained in the model used for the EMMs, why 1.5 cm and 3.0 cm were chosen as the comparison points, what multiplicity correction was applied to the pairwise contrasts, and which software/package was used. Under a strict ANCOVA framework, I would have expected the nonsignificant interaction to be removed (e.g., Engqvist 2005) and the main effects interpreted from the reduced model. Estimated marginal means from the full model are not automatically wrong, but then they should be described as exploratory conditional contrasts rather than as follow-up tests of an interaction that was not clearly supported.

Reference:

Engqvist, L. (2005). The mistreatment of covariate interaction terms in linear model analyses of behavioural and evolutionary ecology studies. Animal Behaviour, 70(4), 967–971.

29. Results/life-history interpretation, LL. 223–231 and 319–337: It should be noted that prey size selection based on flesh-to-shell ratios and the subsequent optimization of breeding timing and clutch size are biologically connected, because prey profitability should influence reserve accumulation, which in turn affects laying date and clutch size. However, the present model seems to link prey size mainly to thiamine intake, not to a prey selection mechanism based on shell mass minimization or to prey size-dependent reserve gain. As a result, the manuscript treats the subsequent reproductive optimization more explicitly than the initial foraging mechanism that may generate the observed association between consumed mussel size and thiamine intake in the first place.

30. Results/phenology logic, LL. 319–327: I find the internal logic of the winter severity and clutch size sentences difficult to grasp. From S1 Appendix, later departure should indeed shorten the time available to build reserves before laying. What remains unclear is why this would then cause clutch size to increase with postponed nesting onset. Biologically, one would intuitively expect reduced time for reserve accumulation to delay laying and/or reduce clutch size, not increase it. Likewise, under the current predation regime in the Baltic, breeding phenology and the interval between arrival and breeding are now strongly shaped by predation risk (Jaatinen et al. 2016; Öst et al. 2022; see my previous reviewer report), which should be acknowledged more explicitly when interpreting these scenarios.

31. Results/robustness summary, LL. 294–298: This is a sweeping statement. The Appendix does suggest that the qualitative prey-size effect on egg thiamine is retained across a range of sensitivity scenarios, but it is not clear which aspect of the result is “relatively insensitive”. Is it the sign of the effect, its magnitude, the separation among prey-size scenarios, or the associated reserve/thiamine trajectories? Please provide a short but more precise summary of what exactly remained robust across Figs. 4-9 in the S1 Appendix.

32. Discussion opener, LL. 300–316: This Discussion opener remains too broad and not fully faithful to the actual scope of the study. The manuscript shows that consumed prey size may affect modelled thiamine levels in females and eggs under the assumptions of the model, but it does not measure fitness consequences for adult females, eggs, or ducklings. I therefore suggest replacing “fitness of consumers” with wording that more closely reflects the actual contents.

33. Discussion, prey-size trade-off, LL. 305–307: Please express this statement more cautiously. Your study does not quantify actual prey size selection, foraging costs, or energetic profitability, and therefore does not directly demonstrate the proposed trade-off. Furthermore, in this study system the opposite scenario is also plausible: because shell weight increases more rapidly than meat weight with increasing mussel size (Öst & Kilpi 1998; see comment 24 above), smaller mussels have a more profitable flesh-to-shell ratio, and if they also contain more thiamine per unit mass, then both the energetic/mechanical and micronutrient considerations would align rather than trade off. I therefore suggest rephrasing this as a conditional possibility, e.g., that prey size-dependent differences in micronutrient content may either reinforce or counteract other drivers of prey size selection, depending on the context.

34. Discussion, mechanism linking mussel size to thiamine content, LL. 368–373: I appreciate that the authors now acknowledge reproductive state as a possible seasonal modifier of the mussel size-thiamine relationship in their response letter. However, this point remains only partly addressed. The mussels analyzed here were sampled during a narrow window in June, and neither stage of maturity nor gonadal condition appears to have been measured or controlled for. Thus, the present dataset does not allow the alternative explanation based on size-dependent reproductive status to be excluded, especially given empirical evidence that thiamine concentrations can decline during reproductive investment in bivalves (e.g., Seguineau et al. 2001ab; see my previous reviewer report). The response letter refers to additional observations from September and October from another project (#49 in the response letter), which could indeed be relevant, but these data are not presented in the revised manuscript or supporting information and therefore cannot resolve the issue in the present submission. I therefore recommend that the authors frame metabolic scaling as one plausible explanation rather than the sole established mechanism, and explicitly discuss the alternative reproductive-status hypothesis in the Discussion, since it remains a viable alternative and hence should not be dismissed too lightly when interpreting the results.

35. Discussion, use of Hylander et al. (2024), LL. 317–319: To recap my earlier comment, I do not think that Hylander et al. (2024) can be used here as direct support for the association between mussel size and thiamine content. This paper addresses thiamine transfer and trophic dilution across different trophic levels, not body size variation within a single prey species at one trophic level. The present wording therefore overextends the scope of that reference. Please rephrase this section so that the trophic-position-based mechanism discussed in Hylander et al. (2024) is not presented as direct evidence for the intraspecific pattern reported here.

36. Discussion, behavioural consequences, LL. 320–325 and 357–367: The present study does not show that prey thiamine content drives diversification of diet or foraging tactics, and in common eiders prey size choice can be explained by simple and straightforward processes such as shell processing/flesh-to-shell ratios. The current wording therefore introduces an additional speculative mechanism that is not tested here and may be less parsimonious than existing explanations. Please rephrase as a speculative possibility or delete this section completely. Likewise, the phrase “large-prey preference” seems too axiomatic here. This manuscript does not measure prey size preference, and such a preference is not generally established for the focal system. Please rephrase more neutrally, for example as “consumption of relatively large prey,” so that the text does not imply a demonstrated behavioural tendency that has not been shown in the present study.

37. Discussion, interpretation of brain-size literature, LL. 335–339: Unfortunately, this sentence still misrepresents both the findings and the mechanism of the cited studies. Öst and Jaatinen (2015) concerned nest success (egg predation risk), breeding phenology, and post-hatch brood-rearing coalition formation; it did not test whether offspring of relatively larger-brained females had higher survival after hatching. Moreover, the likely explanation for the higher nest success of relatively larger-headed females in dangerous years is not faster coalition formation, a process only affecting post-hatch offspring survival, but rather their later breeding phenology in relation to the seasonal timing of predation. As shown in Jaatinen et al. (2019), predation peaks earlier in high-risk years, such that later-breeding, relatively larger-headed females may enjoy higher adult survival and nest success under those conditions, whereas the reverse holds in benign

years. The faster formation of brood-rearing coalitions by relatively larger-headed females in dangerous years was interpreted as evidence of greater responsiveness to prevailing predation risk, not as a demonstrated cause of higher offspring survival. I therefore recommend rewriting this passage to distinguish clearly among nest success, adult female survival, and post-hatch antipredator behaviour.

38. Discussion, Laursen & Møller (2021), LL. 339–342: I do not think this revision fully resolves the concern raised previously. While the claim has been moved from the Abstract to the Discussion, the text still states that females with large brains "feed on considerably larger prey" and then refers to a "preference" for large prey. This still overstates what Laursen & Møller (2021) actually shows. That study infers prey size from gizzard contents in a single wintering region; it does not measure preference relative to prey availability or establish a female-specific pattern for blue mussels (both sexes were pooled in the analysis). I therefore recommend replacing this with more neutral wording, e.g. that in one geographic setting, head volume in adult eiders was positively associated with the size of consumed prey, and avoiding the term "preference" unless selection relative to availability has been demonstrated.

39. Profitability-based prey choice, LL. 357–367: I am not convinced that the manuscript adequately addresses the more biologically realistic alternative that prey size choice is driven primarily by profitability-related rules of thumb. Good alternative candidates include shell-processing constraints and maximization of flesh-to-shell ratios. These mechanisms are not peripheral to the manuscript's logic: they bear directly on whether smaller mussels might be favoured for reasons entirely independent of thiamine, or whether energetic/mechanical and micronutrient considerations might actually point in the same direction (also see comment 33 above).

I hope you will find these comments constructive and helpful for further improving the manuscript!
