# Supplementary material for: The risk of thiamine deficiency in common eider ducklings increases when females consume large prey
Source: PLoS One. 2026 Sep 16;21(9):e0356777. doi: 10.1371/journal.pone.0356777 (PMC13580947; doi:10.1371/journal.pone.0356777)
Supplement: Supplementary file 7 [file pone.0356777.s007.pdf]

Dear Editors of PLOS One,

Thank you for evaluating our manuscript. We have revised the manuscript based on the reviewers' suggestions and comments, and we have added additional simulations to demonstrate that the conclusions presented are robust. Our responses to the issues raised by the reviewers are provided below, alongside an explanation of the changes we have made. For your convenience, our responses are in dark blue and refer to the locations in the manuscript with changes tracked.

Yours sincerely,
Maciej Ejsmond
On behalf of the authors

PONE-D-25-50326R1

The risk of thiamine deficiency in common eider ducklings increases when females consume large prey

PLOS One

Dear Dr. Ejsmond,

Thank you for submitting your manuscript to PLOS ONE. After careful consideration, we feel that it has merit but does not fully meet PLOS ONE's publication criteria as it currently stands. Therefore, we invite you to submit a revised version of the manuscript that addresses the points raised during the review process.

Please submit your revised manuscript by May 29 2026 11:59PM. If you will need more time than this to complete your revisions, please reply to this message or contact the journal office at plosone@plos.org. When you're ready to submit your revision, log on to https://www.editorialmanager.com/pone/ and select the 'Submissions Needing Revision' folder to locate your manuscript file.

Please include the following items when submitting your revised manuscript:

- A letter that responds to each point raised by the academic editor and reviewer(s). You should upload this letter as a separate file labeled 'Response to Reviewers'.

- A marked-up copy of your manuscript that highlights changes made to the original version. You should upload this as a separate file labeled 'Revised Manuscript with Track Changes'.
- An unmarked version of your revised paper without tracked changes. You should upload this as a separate file labeled 'Manuscript'.

If you would like to make changes to your financial disclosure, please include your updated statement in your cover letter. Guidelines for resubmitting your figure files are available below the reviewer comments at the end of this letter.

If applicable, we recommend that you deposit your laboratory protocols in protocols.io to enhance the reproducibility of your results. Protocols.io assigns your protocol its own identifier (DOI) so that it can be cited independently in the future. For instructions see: https://journals.plos.org/plosone/s/submission-guidelines#loc-laboratory-protocols. Additionally, PLOS ONE offers an option for publishing peer-reviewed Lab Protocol articles, which describe protocols hosted on protocols.io. Read more information on sharing protocols at https://plos.org/protocols?utm_medium=editorial-email&utm_source=authorletters&utm_campaign=protocols.

As the corresponding author, your ORCID iD is verified in the submission system and will appear in the published article. PLOS supports the use of ORCID, and we encourage all coauthors to register for an ORCID iD and use it as well. Please encourage your coauthors to verify their ORCID iD within the submission system before final acceptance, as unverified ORCID iDs will not appear in the published article. *Only* the individual author can complete the verification step; PLOS staff *cannot* verify ORCID iDs on behalf of authors.

We look forward to receiving your revised manuscript.

Kind regards,

Juan J Loor
Academic Editor
PLOS One

Journal Requirements:

If the reviewer comments include a recommendation to cite specific previously published works, please review and evaluate these publications to determine whether they are relevant and should be cited. There is no requirement to cite these works unless the editor has indicated otherwise.

[Note: HTML markup is below. Please do not edit.]

Reviewers' comments:

Reviewer's Responses to Questions

Comments to the Author

1. If the authors have adequately addressed your comments raised in a previous round of review and you feel that this manuscript is now acceptable for publication, you may indicate that here to bypass the “Comments to the Author” section, enter your conflict of interest statement in the “Confidential to Editor” section, and submit your "Accept" recommendation.

Reviewer #1: (No Response)

Reviewer #2: (No Response)

Reviewer #3: (No Response)

---

2. Is the manuscript technically sound, and do the data support the conclusions?

The manuscript must describe a technically sound piece of scientific research with data that supports the conclusions. Experiments must have been conducted rigorously, with appropriate controls, replication, and sample sizes. The conclusions must be drawn appropriately based on the data presented.

Reviewer #1: Partly

Reviewer #2: Yes

Reviewer #3: Yes

---

3. Has the statistical analysis been performed appropriately and rigorously?

Reviewer #1: Yes

Reviewer #2: Yes

Reviewer #3: Yes

---

4. Have the authors made all data underlying the findings in their manuscript fully available?

The PLOS Data policy requires authors to make all data underlying the findings described in their manuscript fully available without restriction, with rare exception (please refer to the Data Availability Statement in the manuscript PDF file). The data should be provided as part of the manuscript or its supporting information, or deposited to a public repository. For example, in

addition to summary statistics, the data points behind means, medians and variance measures should be available. If there are restrictions on publicly sharing data—e.g. participant privacy or use of data from a third party—those must be specified.

Reviewer #1: Yes

Reviewer #2: Yes

Reviewer #3: Yes

---

5. Is the manuscript presented in an intelligible fashion and written in standard English?

PLOS ONE does not copyedit accepted manuscripts, so the language in submitted articles must be clear, correct, and unambiguous. Any typographical or grammatical errors should be corrected at revision, so please note any specific errors here.

Reviewer #1: Yes

Reviewer #2: Yes

Reviewer #3: Yes

---

6. Review Comments to the Author

Please use the space provided to explain your answers to the questions above. You may also include additional comments for the author, including concerns about dual publication, research ethics, or publication ethics. (Please upload your review as an attachment if it exceeds 20,000 characters)

Reviewer #1: My comments are too long to fit into this window, so I attach a separate file with my comments (PDF).

Response #1: Comments by Reviewer 1 with responses are pasted below.

Reviewer Comments to Authors

Manuscript ID: PONE-D-25-50326R1

The risk of thiamine deficiency in common eider ducklings increases when females consume large prey

by Maciej Jan Ejsmond, Anna Ejsmond, Marc M. Hauber, Jón Einar Jónsson, Andreas M. Waser & Samuel Hylander

This revised manuscript combines empirical measurements of thiamine concentrations in Baltic blue mussels with a life-history model of female common eiders to examine whether consumption of larger mussels could reduce maternal and egg thiamine levels.

The manuscript has clearly undergone substantial revision, and several changes have improved readability and transparency. The authors have tempered some of the strongest formulations, added new sensitivity analyses in S1 Appendix (including alternative calculations of thiamine, stochastic food intake, migration-related assumptions, and maximal clutch size), provided some additional statistical output for the mussel thiamine-size analysis, and acknowledged more explicitly that predation is a major and probably dominant driver of the decline of Baltic eiders. I also appreciate that the revised Discussion now at least recognizes the potential seasonal dependence of the mussel size-thiamine relationship (e.g., due to variable reproductive state of mussels), and that some points raised by Reviewer #2, such as the maximal clutch size assumption and the transparency of the statistical results, have been addressed to some extent. These are important and meaningful improvements.

I recognize that my earlier review may at times have sounded severe, reflecting my continued skepticism toward the thiamine-deficiency hypothesis in this system. That was not intended unfairly, and I appreciate the substantial effort the authors have made in revising the manuscript. Nevertheless, I remain disappointed that several of the core concerns were either only partly addressed, treated superficially, or discussed so vaguely that readers may still struggle to understand their full implications.

Response #1: Thank you for acknowledging our efforts to revise the manuscript, and for taking the time to help us improve our work. We appreciate Reviewer 1's comments and have incorporated many of their suggestions to enhance the manuscript. Please note that we acknowledge the leading role of predation and its impact on the Baltic population of common eiders (see L35, 77–79 and 383–386). We have also moderated our rhetoric that was in favor of the thiamine deficiency hypothesis (this change has been noted by Reviewer 3; see Response #52). We do not consider the severity of the Reviewer 1 report to be problematic or unfair.

However, we do see a core problem in Reviewer 1's comments: the use of rhetoric on the 'not evidence-based and vague impact of thiamine deficiency on Baltic common eiders' in our text, while Reviewer 1 completely marginalize the work of Balk et al. (2009, 2016) and Mörner et al. (2017) in their comments. The aforementioned papers have been cited in our manuscript and: (1) document thiamine deficiency in common eiders with negative consequences for survival; (2) concern the Baltic Sea population of common eiders; and (3) have been highly cited and recognized by the scientific community. These results were further supported by Svedäng et al. (2018), who reported the mass mortality of thiamine-deficient common eider ducklings in a Swedish colony.

Although Reviewer 1 is highly skeptical of the thiamine deficiency hypothesis, their narrative, which practically rejects the conclusions of the aforementioned articles, should be based on arguments. Neither Reviewer 1 nor the publications suggested in their report have provided these arguments. Therefore, we agree with Reviewer 3's suggestion (see Response #55) that Reviewer 1 should formally develop a discussion on the importance and negative effects of

thiamine deficiency on Baltic common eiders by writing a commentary or opinion piece presenting their logic.

In our view, the results and conclusions of Balk et al. (2009, 2016), Mörner et al. (2017) and Svedäng et al. (2018) are sufficient to consider thiamine deficiency to be a factor impacting the survival of common eiders in the Baltic Sea. This constitutes a fundamental difference between us and Reviewer 1, whose statements practically reject the aforementioned literature. Therefore, we believe that introducing claims by Reviewer 1 that further soften or marginalize the role of thiamine deficiency is inconsistent with our scientific logic. However, we are open to introduce further changes according to the suggestions by the Editors.

The following issues are of major concern:

1) Framing and hierarchy of evidence about thiamine deficiency remain too strong.

Although the rhetoric is softer than in the previous version, the revised manuscript still often presents thiamine deficiency as if it were a well-established major driver of Baltic eider population decline. In my reading, the hierarchy of evidence remains the reverse: increased predation pressure on breeding females is presented in the manuscript itself as the best-supported candidate driver of recent Baltic declines, whereas any additional contribution of thiamine deficiency remains uncertain. This distinction is not reflected clearly enough in the Abstract, opening Introduction, or several parts of the Discussion. Likewise, statements about recruitment failure, adaptive clutch size reduction, and "years with thiamine deficiency" still go beyond the actual evidence base.
Response #2: We disagree with the assertion that "the revised manuscript still often presents thiamine deficiency as if it were a well-established major driver of Baltic eider population decline" (see Response 1). We have introduced several changes to position our rhetoric between sceptics and advocates of the thiamine deficiency hypothesis, and these changes have been remarked by Reviewer 3. However, we are open to introducing further specific changes if requested by the Editors.

2) The mechanistic interpretation of the mussel size-thiamine relationship remains stronger than the evidence supports.

The negative association between mussel size and thiamine concentration is one of the most interesting empirical findings of the study, but its cause is still not resolved. The manuscript still leans strongly toward a metabolic/allometric explanation while alternative mechanisms, especially size-dependent reproductive status of mussels sampled during June, remain incompletely addressed. The new text now mentions that reproductive state may play a role, but still does not explain the expected direction clearly enough for readers. More generally, the paper continues to move too quickly from an interesting observed pattern to a broader mechanistic claim about prey size and micronutrient dynamics in consumers.
Response #3: We mention in the text that the reproductive state may play a role in shaping thiamine content (L421-424). However, we are unsure to what extent the potential correlation between thiamine levels and the reproductive cycle depends on blue mussel size. There is also no clear underlying mechanism, which makes it problematic to determine the "expected direction"

mentioned by Reviewer 1 and explain it to readers. Conversely, thiamine concentrations in the gonads of salmon decrease towards spawning (Todisco et al. 2024), and we now mention this in the discussion (L424-425). Overall, we believe that further studies are required to understand the underlying mechanisms of seasonal variation in thiamine levels in the gonads in relation to body size. These aspects are now also discussed in the paragraph of the discussion dedicated to model limitations and future directions of the model development (see L458-462 and Response #54).

While we agree that there could be a different mechanism underlying the phenomenon of thiamine concentration decreasing with body size, this does not affect the results of our model, regardless of which mechanistic interpretation is given more attention. This is because the model's input was the correlation between mussel size and thiamine concentration. Therefore, the reviewer's point concerns the amount of attention in the discussion and introduction given to potential underlying mechanisms. The proposed metabolic-allometry-based mechanism is strongly supported as it is based on well-understood biochemical reactions in which thiamine acts as a cofactor in metabolic processes (see Response #16). There is also a strong and well documented relationship between body mass and metabolic rate in mussels and other organisms.

3) The biological realism and transparency of the life-history model remain insufficiently improved.

Several core modelling assumptions still appear weakly justified biologically, incompletely explained, or in tension with empirical knowledge of Baltic common eiders. These include the treatment of stopovers, the assumption that females with high reserves arrive essentially lean, the imposed mortality cost of reserves, the handling of breeding phenology and clutch size responses, the starvation threshold, the baseline maximal clutch size, and the treatment of thiamine allocation relative to body fat reserves. The added sensitivity analyses are helpful, but in several cases they show only that the qualitative sign of the effect is retained, not that the baseline assumptions are biologically fully convincing.

Response #4: Firstly, please note that we have introduced several changes to the manuscript in response to specific points raised by Reviewer 1 regarding the modelling of the common eider life histories (see Responses 18, 21, 23, 24, 26, 27 and 30). These clarifications also refer to a new sensitivity analysis of the model outcomes to variation in life history traits.

Reviewer 1 also pointed out that an earlier arrival and an earlier start to nesting are correlated with the production of larger clutches in common eiders (see Response #36 in this report and Response #46 in the previous review report). Our model is flexible and can predict various reactions of clutch size to changes in phenology of migration (see, for example, Fig 4D–F in S1 Appendix and Fig 5 in the S1 Appendix). However, we agree that the main text should present the prevailing pattern in Baltic eiders. This is also because recent shifts in the predation regime (see Jaatinen et al., 2016; Öst et al., 2022), which are now acknowledged in the main text (L178-181), lead to a reduction in clutch size when females arrive later at the breeding grounds. Therefore, we now present scenarios with earlier arrival and nesting correlated with larger clutches in Fig 4D-F of the main text (see Response #36 for details).

While we hope the above-mentioned changes will improve clarity, we disagree with the

general assertion that our assumptions are weakly justified biologically. Rather, life histories of common eiders vary between populations (see literature cited now in L167-171 for examples of this variability in phenology of migration, reserves level at arrival, and clutch size). We have also stated explicitly in the manuscript that our aim was to keep the model general enough so the conclusions can be extrapolated to various populations of common eider (see L167-171 and L273-275).

The life-history traits of the common eider population, as for example clutch size, vary considerably (see Response #24). Similarly, the level of reserves in females arriving in spring depends on the distance they have migrated from wintering to breeding grounds (see Response #21). Please note that we present a sensitivity analysis showing that this variation does not affect the main conclusion of our work (Responses #21, #24). In the case of some of the traits identified by the reviewer, such as the variability of the food-to-reserves conversion rate (see Response #30) or the starvation threshold (see Response #27), parameterization would be difficult due to the scarcity of data. In these cases, we also present additional simulations showing that the main conclusions of our work are not sensitive to changes in starvation thresholds or food-to-reserves conversion rates (see Responses #27, #30).

Please note that sensitivity analysis of model predictions with respect to parameter values is routinely adopted in theoretical ecology, particularly when there is no good data coverage or strict parameterization would make the model very complex.

4) Statistical reporting remains too dispersed.

Reviewer #2 was right to request clearer statistical reporting for the mussel thiamine-size analysis. The revised manuscript now reports more statistical output, but the setup of these analyses is not transparent enough because details are dispersed across Methods, Results, S1 Appendix, and response text. In particular, the underlying fitted model for the empirical mussel analysis is still not described as transparently as it should be, and the rationale for the estimated marginal means contrasts remains insufficiently explained. A short dedicated Statistical analysis section would substantially improve transparency.

Response #5: The statistical methods are now detailed in the material and methods (L 135-143). According to a more conservative modelling approach we have now removed the interaction effect and therefore also the estimated marginal means (L 289-311). We agree that this improves the transparency and stringency of the results.

5) The Discussion still overextends inferences about prey size selection and misuses some cited empirical literature.

The revised Discussion continues to overinterpret what the present study actually shows about prey size choice and consumer behaviour. The manuscript does not measure prey size preference, foraging costs, or energetic profitability, yet still discusses “large-prey preference”, diversification of diets or tactics, and generalized trade-offs as if these followed directly from the current data and model. In the focal eider-mussel system, more straightforward explanations based on flesh-to-shell ratios and shell-processing profitability remain at least as plausible. In addition, some cited literature is still not represented accurately enough, especially with respect to Hylander et al.

(2024), Öst and Jaatinen (2015), Jaatinen et al. (2019), and Laursen & Møller (2021).
Response #6: In line with the suggestions from Reviewer 1 (see Responses #43, #44), we have corrected parts of the discussion that refer to papers by Öst and Jaatinen (2015), Jaatinen et al. (2019), and Laursen and Møller (2021). We now also acknowledge the importance of flesh-to-shell mass ratio in determining common eider prey size preferences (see L230-233 and Response #30) and discuss the potential limitations and future directions of model development in relation to flesh-to-shell mass ratio (see L455-461 and Response #54). However, we disagree with the claim that the paper by Hylander et al. (2024) was not accurately represented in the discussion – see Responses #16 and #41 for details.

Here are my more specific comments, questions and suggested changes to the manuscript:

1. Abstract, LL. 29-48: The Abstract has been improved stylistically, but it still tends to present thiamine deficiency as more strongly established than the manuscript itself justifies. In particular, the present wording still does not make sufficiently clear that predation is the best-supported driver of the Baltic eider decline, whereas any contribution of thiamine deficiency remains uncertain (see Major comment 1 above).
Response #7: We now acknowledge in the abstract that predation is the main cause of the decline of Baltic eiders (L35). For our response to Reviewer 1's claims regarding thiamine deficiency, see Responses #1 and #2.

2. Abstract, L. 29: may constitute a threat.
Response #8: In our opinion, the existing literature justifies the statement in the abstract that "episodic mass mortality due to thiamine (vitamin B1) deficiency is a major threat to top marine consumers worldwide" (see Response #1, #2).

3. Abstract, LL. 31-34: This sentence is still misleading because it highlights a possible link between egg thiamine and the drastic reduction in recruitment without informing the reader that, in the manuscript itself, increased predation pressure is presented as the main driver of the population decline, whereas the contribution of thiamine deficiency remains uncertain. Accordingly, the Abstract should be rephrased to reflect that hierarchy of evidence more explicitly.
Response #9: This sentence has been rewritten to acknowledge that increased predation is the main driver of recent population trends in Baltic eiders (see Response #7).

4. Abstract, LL. 34-36: The new body-size/thiamine motivation remains ecologically incomplete. The phrase "thiamine concentration decreases with the size of organisms at the interspecific level" in the Abstract does not clearly motivate the actual question being asked, which concerns size variation within a single prey species, occupying the same trophic level. As written, the text jumps from an across-species allometric pattern to an intraspecific hypothesis about blue mussels without explaining that inferential step. Moreover, this framing does not fit especially well with Baltic ecology: blue mussels have declined in size over time at least in the northerns parts (e.g., Åkermark et al. 2022; see manuscript for reference), and future warmer, less saline conditions are expected primarily to reduce mussel viability and abundance rather than favour larger mussels (Jaatinen et al. 2021). Thus, the broader interspecific relationship between body size and thiamine

content should be presented only as a general rationale for testing whether a similar pattern occurs within blue mussels, not as implying that ongoing Baltic environmental change would strengthen the proposed prey size/thiamine mechanism. If anything, the opposite may be expected, with this particular risk potentially decreasing in importance if mussel size distributions continue to shift toward smaller individuals.

Reference: Jaatinen K, Westerbom M, Norkko A, Mustonen O, Koons DN. 2021. Detrimental impacts of climate change may be exacerbated by density-dependent population regulation in blue mussels. *Journal of Animal Ecology*, *90*(3), 562–573.

Response #10: We do not agree with the reviewer's 1 main statement: 'The phrase 'thiamine concentration decreases with the size of organisms at the interspecific level' in the Abstract does not clearly motivate the actual question being asked, which concerns size variation within a single prey species, occupying the same trophic level. …". There are classic hypotheses investigating allometry in ecological physiology at the intraspecific level that have been formed as a consequence of interspecific relationships. For example, numerous intraspecific studies have been conducted on the Bergmann rule or the temperature-size rule, and those concepts originated as interspecific phenomena. This is why we disagree that 'the text jumps from an across-species allometric pattern to an intraspecific hypothesis about blue mussels without explaining that inferential step'.

We also disagree that the logic does not fit the Baltic Sea ecosystem. Metabolic rate is governed by enzymatic activity, and key enzymes are dysfunctional without thiamine. In our opinion, this biochemical mechanistic link is sufficient to draw general hypotheses or questions concerning various geographic locations and ecosystems.

However, we are open to editing the aforementioned part of the abstract in accordance with the Editor's guidelines.

5. Abstract, LL. 40-47: As written, this part still implies that females actively reduce clutch size in response to poor thiamine status to improve offspring quality. However, in the manuscript, clutch size reduction is associated with adjustments of breeding phenology/feeding constraints rather than from thiamine input itself, and the manuscript does not exclude alternative ecological causes of reduced observed clutch size, especially predation (e.g., Öst et al. 2008).

Reference:
Öst M, Wickman M, Matulionis E, Steele B. 2008. Habitat-specific clutch size and cost of incubation in eiders reconsidered. Oecologia 158:205–216.

Response #11: Corrected as suggested (L45-47).

6. Keywords, LL. 50-52: The keyword "high juvenile mortality" remains misleading. It is rhetorically loaded, and the manuscript does not provide juvenile mortality data; it provides prey-thiamine measurements and modelled consequences for female and egg thiamine levels. Please replace this with a more neutral and directly relevant keyword.

Response #12: The term 'high juvenile mortality' has replaced the previously used term 'mass juvenile mortality', which is used to describe episodes with mass juvenile mortality due to thiamine

deficiency. To balance the rhetoric, we now use 'high juvenile mortality' and we do not consider this term to be rhetorically loaded. It should be noted that thiamine-deficient common eider ducklings suffer a very high mortality rate (a sevenfold increase, according to Mörner et al. 2017). However, we are open to softening the term further if requested by Editors.

7. Introduction, LL. 56-58: This opening sentence stating that thiamine-deficiency-driven juvenile mortality constitutes a serious conservation problem "affecting ecosystems worldwide" is too broad. Rather, the evidence summarized here appears limited to a number of aquatic systems and mainly observed in top consumers such as salmonids, while the relevance to Baltic common eiders is presented later in the manuscript as uncertain and potentially subordinate to predation. I therefore recommend tempering this sentence and aligning it more closely with the actual taxonomic and geographic scope of the evidence cited.
Response #13: We disagree with Reviewer 1 that the opening sentence is too broad. It is common in scientific articles to provide a broad background to the work in order to increase its impact and appeal to a wider readership (see also Responses #1 and #2). However, we are open to changing the sentence if requested by the Editors.

8. Introduction, LL. 66-70: I appreciate that this revised sentence is rhetorically softer than the original, but it still sidesteps rather than resolves my earlier concern. By stating that juvenile mortality episodes in Baltic common eiders "raise concerns" about thiamine deficiency in benthic food webs, the text still implicitly hints at a causal connection between eider juvenile mortality and thiamine deficiency, despite the fact that the manuscript itself later states that increasing predation pressure has been suggested as the main cause of the population decline and that any contribution of thiamine deficiency remains unclear. I therefore recommend rewriting this passage so that the hierarchy of evidence is presented immediately and explicitly, rather than first emphasizing thiamine and only later acknowledging predation as the dominant explanation.
Response #14: We disagree. The existing literature (see Responses #1 and #2) provides sufficient evidence to conclude that there is "a causal connection between eider juvenile mortality and thiamine deficiency". In our opinion, this comment reflects the reviewer's attitude towards the thiamine deficiency hypothesis. We do not think it is necessary to rewrite the sentence. However, we are open to introducing changes if requested by the Editors.

Please note, we have already softened our rhetoric about thiamine deficiency. It almost appears that reviewer 1 is trying to slowly remove any statement on thiamine deficiency from our article, which eventually would lead to our hypothesis not being 'valid' anymore, making the study unnecessary/not worth publishing.

Finally, we agree with Reviewer 3 that predation and thiamine deficiency can interact to affect offspring mortality (see Reviewer 3's suggestion below that low thiamine levels may potentially increase vulnerability to predation). Please note that studies on predation in Baltic eiders mentioned by the Reviewer 1, rarely analyze thiamine levels in common eiders. This could result in an imbalance in the focus given to the proximate and ultimate drivers of the decline in the Baltic eider population.

9. Introduction, L. 68 and again in the Discussion: “Top consumer” seems an imprecise label for common eiders. Because eiders are themselves preyed upon, the term may overstate their trophic position. A more accurate label would be e.g. “benthic feeder” or “molluscivore”.
Response #15: Corrected (L71, L81-82).

10. Introduction, LL. 82-97: I scrutinized the paper by Hylander et al. (2024), and I remain unconvinced that it supports the arguments presented here. That paper demonstrates trophic dilution of thiamine across a pelagic food web, i.e., declining concentrations from phytoplankton to zooplankton to planktivorous and piscivorous fish, and discusses this pattern in terms of food web transfer, transfer efficiency, and community composition. It does not demonstrate that body size *per se* is the causal driver of reduced thiamine concentrations, nor does it provide direct support for extrapolating such a pattern to size variation within a single prey species such as blue mussels, all of which occupy the same trophic level. The current wording therefore conflates trophic position with body size. If the authors wish to motivate a size effect on thiamine levels in mussels, that rationale should be presented separately, rather than as if it would follow directly from the trophic-level analysis in Hylander et al. (2024). The rationale based on allometry between body size and metabolic rate is clearly preferable here, but it remains a hypothesis-generating framework rather than a demonstrated mechanism.
Response #16: Data reviewed by Hylander et al. (2024) showed a decrease in thiamine concentration by one order of magnitude among multicellular consumers, alongside a decrease in body size (see Fig. 7 from the aforementioned work, pasted below). Thiamine is an essential cofactor in the pyruvate dehydrogenase complex, connecting glycolysis to the Krebs cycle (citric acid cycle) by converting pyruvate into acetyl-CoA. Without thiamine, the enzyme is not functional, which disables the Krebs cycle. We also cite papers by Rucker and Steinberg (2002) and Rucker and Storms (2002), who demonstrated that thiamine demand is proportional to metabolic rate and body size (see L91). In our opinion, the essential role of thiamine in determining metabolic rate and the fact that the metabolic rate per unit mass decreases with body size among life forms justifies what has been written in the introduction (L85–101). Furthermore, we also cite papers by Hauber et al. (2026a, 2026b), which shows that changes in thiamine concentration can decrease with body size at the intraspecific level in fish. Furthermore, in L99-101, we state that “we hypothesized that the thiamine levels decrease with mussel size, and that consuming large blue mussels by common eiders, may alter thiamine intake with diet (Fig 1A).”, which clearly signals the formulation of a hypothesis rather than a reference to 'a demonstrated mechanism', as suggested by Reviewer 1.

However, we are open to further change the introduction in line with the Editors' suggestions.

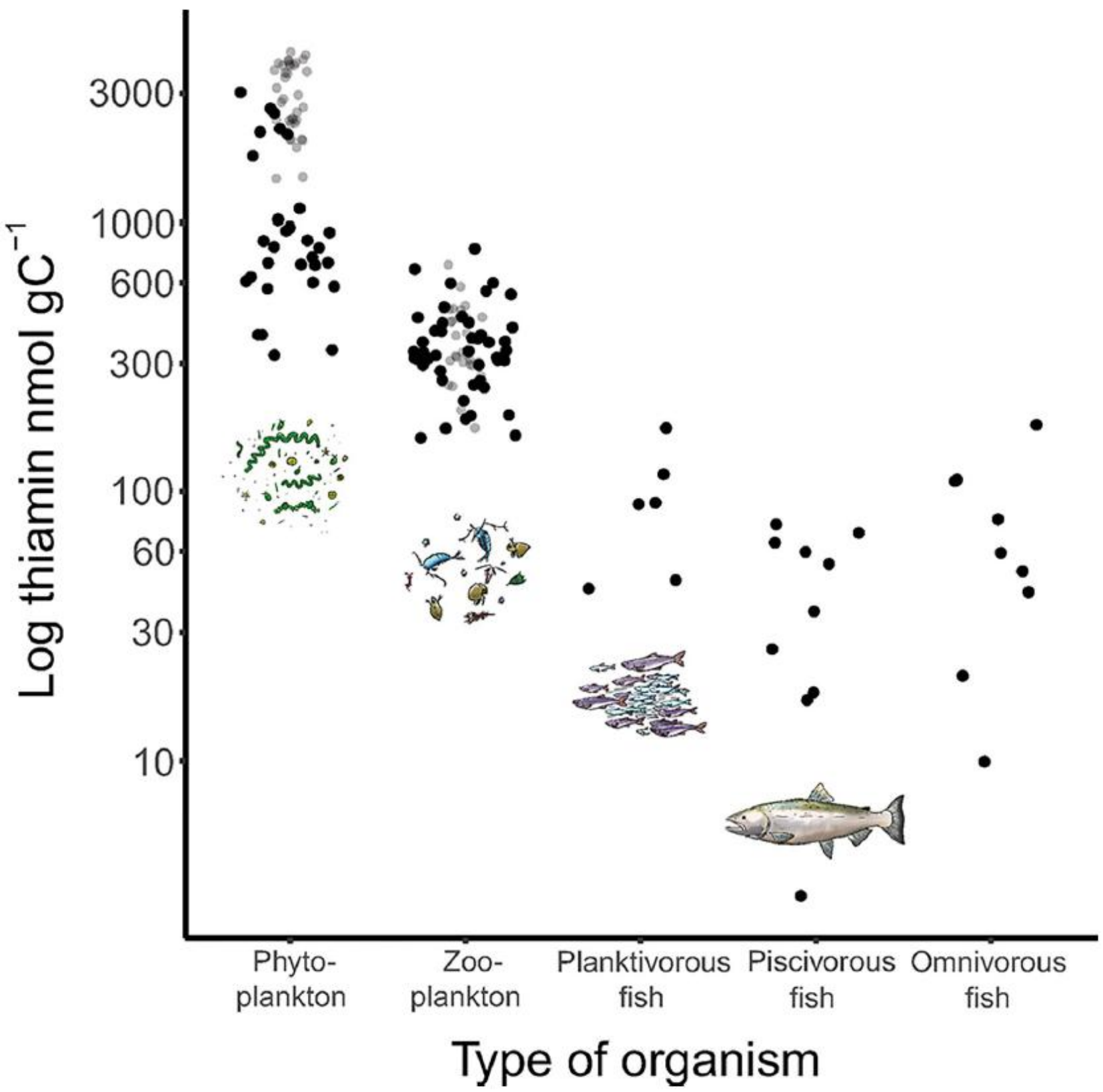

Fig 7. Total thiamin content (log; nmol gC^{-1}) in phytoplankton, zooplankton, planktivorous fish, piscivorous fish and omnivorous fish (drawings modified from [52]). Data collected in a literature search (black dots) on the concentrations of thiamin in different organisms (phytoplankton, zooplankton, planktivorous fish, piscivorous fish, and omnivorous fish). The grey dots represent mesocosm concentrations of phytoplankton and zooplankton.

11. Introduction, LL. 99-109: The wording "Here we test whether..." is potentially misleading. The manuscript does not empirically test thiamine levels in common eider ducklings; rather, it measures thiamine concentrations in blue mussels of different sizes and then uses a life-history model to predict consequences for female and egg thiamine status. Please rephrase this sentence to distinguish clearly between the empirical mussel analysis and the model-based inference about eider eggs and offspring.
Response #17: The sentence has been changed so it does not imply empirical test (L102).

12. Material and Methods, LL. 114-123: The treatment of stopovers remains too categorical. While stopovers can occur in spring-migrating common eiders, published evidence and the general migration pattern of Baltic common eiders suggest that spring migration is often rapid and highly synchronized (Alerstam et al. 1974; see my previous reviewer report for reference), with some females moving north without prolonged staging. I would therefore recommend softer wording, e.g., "potential stopover areas", unless the authors can provide direct evidence that spring staging in these locations is frequent and typical for Baltic female common eiders.
Response #18: Corrected as suggested (L117).

13. Material and Methods, L. 132: The statistical description is imprecise here. Because site is a categorical factor whereas mussel size is a continuous covariate, this analysis is more appropriately described as a general linear model/ANCOVA (including site, length, and their interaction) rather than simple "linear regression".
Response #19: See Response #5.

14. Outline, LL. 137-149: The phrase "during the pre-breeding period" suggests that reserve accumulation occurs before breeding begins. That seems too restrictive, given that females often continue feeding intensively into the early laying period (e.g., Hanssen et al. 2002; Andersson and Waldeck 2006; see my comment #62 in the response letter).
Response #20: Now specified (L148).

15. Life history and migration assumptions, LL. 164–167: I remain unconvinced by the assumption that females with high reserves would reach the breeding grounds "close to or in a lean body condition". Although the authors now note that alternative high-arrival-reserve scenarios did not qualitatively change the direction of the prey-size effect, that does not remove the need to justify the baseline assumption biologically. In its present form, the model still seems to suppress spring carry-over capital more strongly than current evidence of mixed capital-breeding strategies (Sénéchal et al. 2011, Hobson et al. 2015, Jaatinen et al. 2016; see my previous reviewer report for references) would suggest.
Response #21: In populations of common eiders that undertake long-distance migration, females arrive in lean or near-lean body condition (e.g. Waltho and Coulson 2015, Ejsmond et al. 2021). While we agree that females migrating shorter distances will arrive with a certain level of reserves, body condition cannot be derived mechanistically without modelling the geographic structure of the population. This would make our work complex and difficult to follow. In the previous round of reviews, we therefore introduced a sensitivity analysis showing that the main conclusions of our work remain unchanged regardless of whether females arrive at the breeding grounds with body reserves (see Fig. 5 in S1 Appendix).

16. LL. 137–139, 164–167: Relatedly, there is an element of internal inconsistency here. The text states that reserve accumulation precedes migration, egg laying and incubation, yet Appendix S1 simultaneously assumes that females departing with maximal reserves arrive at the breeding grounds essentially lean. This can only be reconciled if reserve accumulation before reproduction is assumed to occur mainly after arrival rather than being carried through migration. If so, this should be stated explicitly; otherwise, the current wording misleadingly suggests substantial spring carry-over capital, even though the model largely suppresses it.
Response #22: Now stated explicitly that reserve accumulation is assumed to occur mainly after arrival to breeding grounds (L149).

17. LL. 165–167; see also Fig. 5 in S1 Appendix (migration-cost sensitivity): I checked Fig. 5 in the S1 Appendix, and the statement that the "conclusions did not change" is too vague. What appears unchanged is primarily the qualitative ranking that larger consumed prey leads to lower thiamine levels. That does not demonstrate that the baseline migration assumption is biologically realistic,

only that the sign of the effect is robust.
Response #23: This has now been specified (L187-192). It is realistic for female common eiders to arrive in a lean or close-to-lean body condition, and the condition at arrival represent variation in the distance of migration among common eider populations (see Response #21).

18. Clutch size parameterization, LL. 171–173: I appreciate that the authors now show, in response to Reviewer #2, that the main conclusions are qualitatively similar when maximal clutch size is set to 6. However, the choice of 5 as the biological default still appears restrictive. In Baltic common eiders, clutches of six eggs are not exceptional, and direct evidence exists that a single female may lay up to seven eggs in the same nest (Waldeck et al. 2004). I therefore suggest either using 6 as the biologically more realistic default upper bound, or justifying more clearly why 5 was retained as the baseline despite the broader observed range.

Reference: Waldeck P, Kilpi M, Öst M, Andersson M. 2004. Brood parasitism in a population of common eider (*Somateria mollissima*). Behaviour 141:725–739.
Response #24: We used in the model clutch size of 5 eggs in line with the long-term data on average clutch size in Finland in Denmark according to the book "The Common eider" by Waltho and Coulson 2015 (see Fig 8.3 in Waltho and Coulson 2015 for a long-term analysis of average clutch size in Finland and Denmark, and our Response #97 in the previous file with Responses to reviewers). We agree that there is individual variation and geographical variation in clutch size and that is why we have included sensitivity analysis – a standard technique in theoretical modelling used to show if the conclusion of the work is sensitive to changes in value of particular parameter. Note that the conclusion for scenarios with 5 and 6 eggs were in fact qualitatively the same – i.e. did not change the main messages of our work (see Fig 2 in the main text and Fig 12 in S1 Appendix). Hence, we argue that we clearly justify why 5 eggs was used as a baseline and show that changing it to 6 has no impact on our conclusions.

19. Timing of nesting / breeding phenology, LL. 208–213: The phrase "timing of nesting" is ambiguous. Based on my reading, it appears that this variable may refer to laying dates rather than hatching dates. If so, the assumptions and wording should be made more explicit. Likewise, the current causal chain linking winter conditions, arrival timing, reserve accumulation, and clutch size remains difficult to follow in places and should be explained more transparently.
Response #25: Please note, the line numbering referred to by the reviewer in their previous comments (above) matched the clear copy of the main text. However, lines 208–213 of the clear main text describe changes in thiamine levels during incubation without using a phrase "timing of nesting". The reviewer likely meant lines 208–213 of the text with changes marked, which indeed fit with the context and contain the phrase 'timing of nesting'. In our responses below we specify which copy of the text (clear or with changed marked) has been referred to by the Reviewer 1 in our opinion.

We have now specified the term 'timing of nesting' and use timing of hatching instead (see L201). We have also clarified the description of the model in lines L202-207.

20. LL. 214–220; see also S1 Appendix, section 2.3: I can see from the Appendix code that incubation survival is set as p_incub = p_rest^5, but I could not identify a direct empirical reference justifying the exponent 5 itself. Please clarify whether this multiplier is based on data, a scaling assumption, or model convenience.

Response #26: Reviewer 1 refers to the text with changes marked (see Response #25).

In the S1 Appendix we stated that "For simplicity, we also assumed that survival m_f applied to all female behaviors except incubation (Mohring et al. 2022), during which the probability of survival was lower and given by $m_i = m_f^v$, with v=5 in the main results". As we assumed v=5 for the sake of simplicity, we now present additional scenarios with v set to 2 and 10, that show our model predictions are not sensitive to assumed value of v (see added Fig 8 in S1 Appendix 1). This is now also stated in the main text (L210-212).

21. Mortality cost of reserves, LL. 216–220: I remain unconvinced by the assumption that survival declines as body reserves increase. This is not a minor modelling detail: the Appendix code imposes an explicit exponential mortality cost of reserves, thereby shaping the optimal strategy and subsequent thiamine trajectories. Yet the empirical literature points in the opposite direction. In the Baltic population, Ekroos et al. (2012) found that female body condition at the first documented breeding attempt, specifically chosen to avoid confounding with age, was positively associated with annual apparent survival. Likewise, Laursen et al. (2019; see previous reviewer report) present high female body condition as beneficial for breeding performance, including brood size, survival of young, and hatching success. This empirical evidence of a positive relationship between body condition and survival is also in line with theoretical expectations, such as the asset protection principle (Clark 1994; see previous report) or the state-dependent safety hypothesis (e.g., Luttbeg and Sih 2010; see previous report). The authors now show some sensitivity analyses, but the biological basis for the baseline reserve-survival penalty still seems insufficiently justified.

Reference:

Ekroos J, Öst M, Karell P, Jaatinen K, Kilpi M. 2012. Philopatric predisposition to predation-induced ecological traps: Habitat-dependent mortality of breeding eiders. *Oecologia*, *170*(4), 979–986.

Response #27: Reviewer 1 refers to the text with changes marked (see Response #25).

We responded to a similar point raised by Reviewer 1 previously (see Response #41 in the previous Responses to reviewers). To recap the main point of our response, there is tremendous theoretical and empirical support for the assumption that the mortality rate increases with reserve level in birds, including ducks. This phenomenon, known as the trade-off between starvation and predation, is well recognized in the ecological literature.

However, in order to address the points raised by Reviewer 1 regarding the correlations between annual survival and body condition in common eiders (cf. Descamps et al. 2011, Ekroos et al. 2011; now mentioned also in the main text, lines 217-220), we present additional simulations in which an increased level of reserves is necessary to avoid a high risk of starvation-driven mortality (see added Fig 7 in the S1 Appendix). Nevertheless, our sensitivity analysis shows that increasing the reserves required for starvation protection, i.e. in line with the assumption that survival increases with energy buffers, does not change the model's conclusion (see Fig 7 in S1 Appendix

and L343-349).

Please note, that referring to the work of Clark (1994) in support of the concept of reserves level maximization by natural selection is unmatched. Colin W. Clark is the co-author, with Marc Mangel, of two influential books: Dynamic Modeling in Behavioral Ecology and Dynamic State Variable Models in Ecology. These books are essentially handbooks on how to apply mathematical optimization procedures in modelling of trait optimization by natural selection. In Clark's models, traits are optimized (i.e. they have an optimum), not maximized, as suggested by the interpretation given by Reviewer 1.

22. Thresholds and reserve parameters, LL. 219–220; see also S1 Appendix, sections 2.1–2.3: I checked both the S1 Appendix and the code, and I could not locate any transparent derivation of the threshold Sr = 100 g. It appears to me that this threshold applies to wintering/stopover survival, not to breeding initiation on the breeding grounds. Please clarify the biological meaning of this parameter and where it comes from.

Response #28: Reviewer 1 refers to the text with changes marked (see Response #25).

The sentence in question reads: "Females in lean body condition at breeding grounds and those with reserves $S(t)<S_r$ with S_r set to 100 g (Beuth et al. 2016) at other geographic locations, starved with a 20% reduction in survival (see S1 Appendix)." We now added a comma after '...condition at breeding grounds...' to avoid potential further confusion (L215). Sr sets starvation threshold at wintering grounds and stopover sites and was set according to the work by Beuth et al. 2016 (see also Response #27).

23. LL. 223–227: Please specify the time unit, i.e. that h = 37.5 is a daily reserve use rate (g day^{-1}).

Response #29: Reviewer 1 refers to the text with changes marked (see Response #25).

Specified (L225).

24. Mussel intake and reserve gain, LL. 223–231: The assumed conversion between daily mussel intake and reserve gain may not be generically applicable across geographical areas. In particular, Baltic blue mussels in the less saline northern Baltic Sea have a considerably higher flesh proportion and a lower shell mass than Atlantic mussels. E.g., Öst and Kilpi (1998) showed that the amount of shell an eider must ingest to meet its daily energy needs is much lower in their northern Baltic study area than in Atlantic settings. I therefore doubt that the generic conversion of reserve gain to 1.9 kg of mussels per day is appropriate for all modelled sites. Please discuss this limitation more clearly

Reference: Öst M, Kilpi M. 1998. Blue mussels *Mytilus edulis* in the Baltic: good news for foraging eiders *Somateria mollissima*. Wildlife Biology 4:81-89.

Response #30: Reviewer 1 refers to the text with changes marked (see Response #25).

Thank you for your comment. We have now cited the suggested literature and presented additional sensitivity analyses with scenarios that differ in terms of the conversion of consumed food into reserves gained (see L230-233 and added Fig 11 in S1 Appendix). Please note that, while a lower conversion factor reduces the level of thiamine in the eggs, our conclusion that the level of thiamine in eggs decreases with the size of the prey consumed remains unchanged (L343-349). We

also discussed prey size selection based on the ratio of flesh to shell mass as a potential future direction in model development (L454-462, Response #54).

25. Tentative threshold values, LL. 266–272: The motivation for using the egg-thiamine threshold has improved relative to the previous version because the manuscript now explicitly cites the putative threshold and correctly frames it as tentative. Nevertheless, I do not think the threshold can carry the amount of interpretive weight the manuscript occasionally places on it. From a logical perspective, the fact that model-predicted egg thiamine values fall both below and above a tentative threshold does not demonstrate that the model is "not flawed" with respect to inference about offspring quality.

Response #31: Reviewer 1 refers to the text with changes marked (see Response #25).

Our model was designed as a general representation of common eider life history, capable of capturing variation in different populations (see Response #4). Hence, we aimed to demonstrate the consistency of the relative differences in thiamine egg levels caused by the consumption of prey of different sizes. As a general tool designed to facilitate understanding, our model lacks the predictive power required to interpret thiamine egg levels in terms of threshold levels related to juvenile mortality rates. This has now been explained in the main text (L273-275).

Modelling of mortality of juveniles and thiamine threshold levels in the eggs, would require strict parameterization and an energy budget model likely specific to a single population. Such models typically do not consider evolution as it would make them difficult to follow. On the other hand, energy budget models require precise data on many aspects that were roughly approximated in our model like for example e.g. food preference, conversion of food into reserves, etc. Moreover, we do not think such data are currently available. Please note also that there are only few estimates of egg thiamine threshold levels in marine birds.

Finally, in a simplified, general model such as ours, thiamine levels falling in the range of thiamine threshold estimates show that assumptions regarding thiamine intake and excretion rates have been realistically combined with a general life history of common eiders in our model. It should be noted that the approach based on including in the models a rough approximation of general physiological, demographic or life history aspects, is often adopted in life history evolution studies to bring models closer to certain taxonomic groups or species (e.g. Houston et al. 2007, Ejsmond et al. 2018).

- Houston AI, Stephens PA, Boyd IL, Harding KC, McNamara JM. 2007. Capital or income breeding? A theoretical model of female reproductive strategies, *Behavioral Ecology* 18(1) : 241–250

- Ejsmond MJ, McNamara JM, Søreide J, Varpe Ø. 2018. Gradients of season length and mortality risk cause shifts in body size, reserves and reproductive strategies of determinate growers. *Functional Ecology* 32 : 2395–2406

26. Thiamine allocation and fat reserves, LL. 273–278: I could not locate a reference in the manuscript or S1 Appendix supporting the statement that thiamine "is not allocated to body fat reserves". Is this a modelling assumption or an empirically documented fact? Please either provide an appropriate reference or make it explicit that this is an assumption. I appreciate that the authors

now include a sensitivity analysis of this point, but that does not remove the need to justify biologically why the main formulation is preferable.

Response #32: Reviewer 1 refers to the text with changes marked (see Response #25).

Thiamine is a water-soluble vitamin, and not a fat-soluble one. This means that it cannot be stored in fat tissues. Now specified in the text (L280).

27. Statistical transparency, LL. 129–132 and 281–298: I think the manuscript would benefit from a short, separate Statistical analysis section. While the current structure is not wrong, the statistical details are so dispersed that the setup of analyses becomes rather hard to follow, especially because the paper combines empirical mussel analyses with model-based simulations and sensitivity analyses. A dedicated statistical analysis subsection would substantially improve transparency. In this respect I agree with Reviewer #2.

Response #33: Statistical analyses have now been better described, see Response #5.

28. Empirical mussel analysis/EMMs, LL. 281–298: In my view, listing the pairwise EMM results alone does not allow the reader to assess whether these *post hoc* tests were appropriately specified. Please clarify the underlying fitted model, whether the interaction between site and length was retained in the model used for the EMMs, why 1.5 cm and 3.0 cm were chosen as the comparison points, what multiplicity correction was applied to the pairwise contrasts, and which software/package was used. Under a strict ANCOVA framework, I would have expected the nonsignificant interaction to be removed (e.g., Engqvist 2005) and the main effects interpreted from the reduced model. Estimated marginal means from the full model are not automatically wrong, but then they should be described as exploratory conditional contrasts rather than as follow-up tests of an interaction that was not clearly supported.

Reference: Engqvist, L. (2005). The mistreatment of covariate interaction terms in linear model analyses of behavioural and evolutionary ecology studies. Animal Behaviour, 70(4), 967–971.

Response #34: Reviewer 1 refers to the text with changes marked (see Response #25). Please see Response #5.

29. Results/life-history interpretation, LL. 223–231 and 319–337: It should be noted that prey size selection based on flesh-to-shell ratios and the subsequent optimization of breeding timing and clutch size are biologically connected, because prey profitability should influence reserve accumulation, which in turn affects laying date and clutch size. However, the present model seems to link prey size mainly to thiamine intake, not to a prey selection mechanism based on shell mass minimization or to prey size-dependent reserve gain. As a result, the manuscript treats the subsequent reproductive optimization more explicitly than the initial foraging mechanism that may generate the observed association between consumed mussel size and thiamine intake in the first place.

Response #35: Reviewer 1 refers to the text with changes marked (see Response #25).

With regard to the (referred as lines 223–231), we now acknowledge shortly afterwards that the flesh-to-shell ratio is an important determinant of prey size selection in common eiders (see Response #30). As for part referred as lines 319–337, this section describes the results of a model

formulated earlier in the text, and no additional interpretation is permitted here. However, we are open to making further changes to this section if requested by the Editors.

30. Results/phenology logic, LL. 319–327: I find the internal logic of the winter severity and clutch size sentences difficult to grasp. From S1 Appendix, later departure should indeed shorten the time available to build reserves before laying. What remains unclear is why this would then cause clutch size to increase with postponed nesting onset. Biologically, one would intuitively expect reduced time for reserve accumulation to delay laying and/or reduce clutch size, not increase it. Likewise, under the current predation regime in the Baltic, breeding phenology and the interval between arrival and breeding are now strongly shaped by predation risk (Jaatinen et al. 2016; Öst et al. 2022; see my previous reviewer report), which should be acknowledged more explicitly when interpreting these scenarios.
Response #36: Reviewer 1 refers to the text with changes marked (see Response #25).

Please note that our model is flexible. In response to earlier nesting, it can predict an increase or reduction in clutch size depending on which model parameters are changed (see, for example, Fig 4D–F in S1 Appendix and Fig 5 in the S1 Appendix). However, our model only predicts a reduction in clutch size alongside earlier breeding in scenarios involving a radical reduction in the time window for offspring production (see the previous version of Fig 4D–F). We agree with Reviewer 1 that earlier arrival and nesting are associated with larger clutches in Baltic eiders (see, for example, Response #46 in the previous Reviewer 1 report), and our model typically predicts an increase in clutch size with earlier arrival and nesting (see, Fig 4A-C and Fig 5 in the S1 Appendix). We agree that the main text should present the prevailing pattern found in Baltic eiders, particularly given recent shifts in the predation regime (see Jaatinen et al., 2016; Öst et al., 2022), as well as the reasons aforementioned by Reviewer 1. Hence, we have replaced panels D–F of the previous version of Fig 4 in the main text, with the scenarios presented previously in the S1 Appendix, which illustrate that earlier arrival and an earlier start to nesting are correlated with larger clutches (see the current version of Fig 4D–F). In the main text, we now acknowledge recent shifts in the predation regime and its effect on the timing of nesting and clutch size (L178-181). We have also corrected the description of the results that refer to Fig 4 and the figures in the S1 Appendix regarding changes in the phenology of arrival, nesting and clutch size (L334-339).

Most importantly, please note that the main conclusion from our work does not change, regardless of whether early arrival and nesting correlate with the production of larger or smaller clutches (see L339-341).

31. Results/robustness summary, LL. 294–298: This is a sweeping statement. The Appendix does suggest that the qualitative prey-size effect on egg thiamine is retained across a range of sensitivity scenarios, but it is not clear which aspect of the result is "relatively insensitive". Is it the sign of the effect, its magnitude, the separation among prey-size scenarios, or the associated reserve/thiamine trajectories? Please provide a short but more precise summary of what exactly remained robust across Figs. 4-9 in the S1 Appendix.
Response #37: Reviewer 1 refers to clear copy of the main text (see Response #25).

It is now specified which of our model's qualitative predictions are robust with respect to the sensitivity analysis presented (L343-349).

32. Discussion opener, LL. 300–316: This Discussion opener remains too broad and not fully faithful to the actual scope of the study. The manuscript shows that consumed prey size may affect modelled thiamine levels in females and eggs under the assumptions of the model, but it does not measure fitness consequences for adult females, eggs, or ducklings. I therefore suggest replacing "fitness of consumers" with wording that more closely reflects the actual contents.
Response #38: Reviewer 1 refers to clear copy of the main text (see Response #25).

The sentence has been changed (L353).

33. Discussion, prey-size trade-off, LL. 305–307: Please express this statement more cautiously. Your study does not quantify actual prey size selection, foraging costs, or energetic profitability, and therefore does not directly demonstrate the proposed trade-off. Furthermore, in this study system the opposite scenario is also plausible: because shell weight increases more rapidly than meat weight with increasing mussel size (Öst & Kilpi 1998; see comment 24 above), smaller mussels have a more profitable flesh-to-shell ratio, and if they also contain more thiamine per unit mass, then both the energetic/mechanical and micronutrient considerations would align rather than trade off. I therefore suggest rephrasing this as a conditional possibility, e.g., that prey size-dependent differences in micronutrient content may either reinforce or counteract other drivers of prey size selection, depending on the context.
Response #39: Reviewer 1 refers to clear copy of the main text (see Response #25).

Thank you for the comment. Now formulated more cautiously (L356-358). We have responded to the comment 24 by Reviewer 1 above (see Response #30).

34. Discussion, mechanism linking mussel size to thiamine content, LL. 368–373: I appreciate that the authors now acknowledge reproductive state as a possible seasonal modifier of the mussel size-thiamine relationship in their response letter. However, this point remains only partly addressed. The mussels analyzed here were sampled during a narrow window in June, and neither stage of maturity nor gonadal condition appears to have been measured or controlled for. Thus, the present dataset does not allow the alternative explanation based on size-dependent reproductive status to be excluded, especially given empirical evidence that thiamine concentrations can decline during reproductive investment in bivalves (e.g., Seguineau et al. 2001ab; see my previous reviewer report). The response letter refers to additional observations from September and October from another project (#49 in the response letter), which could indeed be relevant, but these data are not presented in the revised manuscript or supporting information and therefore cannot resolve the issue in the present submission. I therefore recommend that the authors frame metabolic scaling as one plausible explanation rather than the sole established mechanism, and explicitly discuss the alternative reproductive-status hypothesis in the Discussion, since it remains a viable alternative and hence should not be dismissed too lightly when interpreting the results.
Response #40: Reviewer 1 refers to clear copy of the main text (see Response #25).

We have responded to a very similar comment by the Reviewer 1 above (Response #3). To sum up there are many unknowns including the ultimate mechanism on why the thiamine level

would increase in reproductive tissues. Also, there are organisms in which thiamine level decreases toward spawning (now mentioned in the main text L424-425). We now mention in the text that the development of this hypothesis is an important future directions for the model development (L458-462, Response #54).

35. Discussion, use of Hylander et al. (2024), LL. 317–319: To recap my earlier comment, I do not think that Hylander et al. (2024) can be used here as direct support for the association between mussel size and thiamine content. This paper addresses thiamine transfer and trophic dilution across different trophic levels, not body size variation within a single prey species at one trophic level. The present wording therefore overextends the scope of that reference. Please rephrase this section so that the trophic-position-based mechanism discussed in Hylander et al. (2024) is not presented as direct evidence for the intraspecific pattern reported here.
Response #41: Reviewer 1 refers to clear copy of the main text (see Response #25).

The data reviewed by Hylander et al. (2024) showed a decrease in thiamine concentration by one order of magnitude among multicellular consumers, alongside a reduction in body size (See Response #16 for details). Please note that in this paragraph we now also cite papers by Hauber et al. (2026a, 2026b), which provide examples of intraspecific decreases in thiamine concentration with body size in cod and whitefish (L370-371).

36. Discussion, behavioural consequences, LL. 320–325 and 357–367: The present study does not show that prey thiamine content drives diversification of diet or foraging tactics, and in common eiders prey size choice can be explained by simple and straightforward processes such as shell processing/flesh-to-shell ratios. The current wording therefore introduces an additional speculative mechanism that is not tested here and may be less parsimonious than existing explanations. Please rephrase as a speculative possibility or delete this section completely. Likewise, the phrase "large-prey preference" seems too axiomatic here. This manuscript does not measure prey size preference, and such a preference is not generally established for the focal system. Please rephrase more neutrally, for example as "consumption of relatively large prey," so that the text does not imply a demonstrated behavioural tendency that has not been shown in the present study.
Response #42: Reviewer 1 refers to clear copy of the main text (see Response #25).

Rephrased as suggested (L371-377). For changes in part referred to as lines 357-367 see Response #45.

37. Discussion, interpretation of brain-size literature, LL. 335–339: Unfortunately, this sentence still misrepresents both the findings and the mechanism of the cited studies. Öst and Jaatinen (2015) concerned nest success (egg predation risk), breeding phenology, and post-hatch brood-rearing coalition formation; it did not test whether offspring of relatively larger-brained females had higher survival after hatching. Moreover, the likely explanation for the higher nest success of relatively larger-headed females in dangerous years is not faster coalition formation, a process only affecting post-hatch offspring survival, but rather their later breeding phenology in relation to the seasonal timing of predation. As shown in Jaatinen et al. (2019), predation peaks earlier in high-risk years, such that later-breeding, relatively larger-headed females may enjoy higher adult survival and nest success under those conditions, whereas the reverse holds in benign years. The faster formation of

brood-rearing coalitions by relatively larger-headed females in dangerous years was interpreted as evidence of greater responsiveness to prevailing predation risk, not as a demonstrated cause of higher offspring survival. I therefore recommend rewriting this passage to distinguish clearly among nest success, adult female survival, and post-hatch antipredator behaviour.

Response #43: Reviewer 1 refers to clear copy of the main text (see Response #25).

Corrected as suggested (L388-391).

38. Discussion, Laursen & Møller (2021), LL. 339–342: I do not think this revision fully resolves the concern raised previously. While the claim has been moved from the Abstract to the Discussion, the text still states that females with large brains "feed on considerably larger prey" and then refers to a "preference" for large prey. This still overstates what Laursen & Møller (2021) actually shows. That study infers prey size from gizzard contents in a single wintering region; it does not measure preference relative to prey availability or establish a female-specific pattern for blue mussels (both sexes were pooled in the analysis). I therefore recommend replacing this with more neutral wording, e.g. that in one geographic setting, head volume in adult eiders was positively associated with the size of consumed prey, and avoiding the term "preference" unless selection relative to availability has been demonstrated.

Response #44: Reviewer 1 refers to clear copy of the main text (see Response #25).

Corrected as suggested (L391-394).

39. Profitability-based prey choice, LL. 357–367: I am not convinced that the manuscript adequately addresses the more biologically realistic alternative that prey size choice is driven primarily by profitability-related rules of thumb. Good alternative candidates include shell-processing constraints and maximization of flesh-to-shell ratios. These mechanisms are not peripheral to the manuscript's logic: they bear directly on whether smaller mussels might be favoured for reasons entirely independent of thiamine, or whether energetic/mechanical and micronutrient considerations might actually point in the same direction (also see comment 33 above).

Response #45: Reviewer 1 refers to clear copy of the main text (see Response #25).

We have now removed the sentence suggesting that recent shifts in age structure of blue mussels in the Baltic Sea may lead to a decrease in thiamine intake by common eiders, due to consumption of larger prey (L409-412). In the main text, we now also acknowledge that the flesh-to-shell mass ratio is an important determinant of prey size selection in the common eider (see Responses #30, #35). We also discuss prey size selection based on the flesh-to-shell mass ratio in the paragraph discussing limitations in interpretation and future directions for model development (L454-462, see Response #54).

I hope you will find these comments constructive and helpful for further improving the manuscript!

Reviewer #2: I thank the Authors for their thorough revision of the manuscript and I appreciate the way the authors have revised the manuscript. I think that this manuscript provides a good modelling framework that could be linked to empirical data in the future or extended to other

molecules than thiamine.
Response #46: Thank you for your efforts in improving our manuscript. We have now included a separate paragraph in the discussion section that suggests future directions for its development (L451-462 and Response #54).

I only have a few minor comments:
- Maybe I missed it but did the authors mention what software was used for statistical analyses and modelling? I think that it would be helpful for researchers wanting to re-use the code.
Response #47: This has been stated in Appendix 2 of the file containing the code, but it is now also included in the main text (L282-283).

- l. 89: "rates scales" : "s" should be removed from one of the two words
Response #48: Corrected (L92).

- l. 201: I still find this part a bit hard to understand but I think that I have now understood that for each scenario the size of the mussel is fixed. Could the authors maybe write it as "Below, we present the results of five scenarios in which females foraged on mussels of fixed lengths: 1.5, 2, 2.5, 3 and 3.5cm". As the rest of the modelling incorporates variation around parameters, I was also expected that for prey preference and I think it can help readers to be clear about it.
Response #49: Correction made as suggested (L234). Thank you for the comment. Yes, prey size scenarios were indeed exceptional in that no variation was considered. This is because it would require assumptions about the behavioral aspects of prey preference and foraging strategies, which obviously determine the distribution of consumed prey sizes. We did not want to enter this field as this would be too much for a single paper in our opinion. However, we now discuss modelling of prey size preference in the new paragraph of the discussion dedicated to the model's limitations and future development directions (see L451-462 and response #54).

- l. 389: this sentence is long, and I suggest splitting it in two to improve readability (e.g., at the end of l. 389
Response #50: Corrected as suggested (L442-445).

- l.390: I don t think that the word spawning is used for birds and I would use laying instead.
Response #51: Corrected as suggested (L444).

Reviewer #3: I've now read the revised manuscript and the comments of reviewer #1 and #2. Overall, I think the authors have responded well to the very detailed and helpful commentary by both reviewers, as well as my own. My comments were relatively minor. However, Reviewer #1 provided extensive feedback - in some cases critical of some of the results or conclusions of the paper. Specifically, Reviewer #1 was concerned with three issues, in my reading. First and foremost was disagreement about the effect and the degree of impact of thiamine deficiency on egg viability and duckling survival versus an alternative hypothesis of predation on ducklings driving the decline in Baltic eiders. It appears to be an issue of considerable debate, and I found it interesting although I knew little of this controversy prior to this manuscript. I'll comment a bit further on this below.

Second, Reviewer #1 had several comments/concerns about issues related to general life history traits of eiders, and their application in the dynamic state model. These ranged from issues about the cost of reproduction, determination of clutch size, capital versus income breeding, migration and breeding phenologies, and breeding strategies writ large. Third, Reviewer #1 had a variety of comments that I'll loosely classify as behavioral based, but specifically related to prey size selection and the mechanisms whereby selection for a certain size classes of mussels could lead to thiamine deficiencies, the relative role of brain size, the risk of predation on female behavior and timing of breeding, etc. The authors responded extensively to Reviewer #1's comments and made several modifications to the paper, notably reducing some of the strong rhetoric in favor of the thiamine deficiency hypothesis.
Response #52: Thank you for your efforts in improving our work, and for summarizing our responses to the points raised by Reviewer 1. While we believe that the rhetoric used in our manuscript is now moderate, we are open to making further changes if requested by the Editors (see Response #1).

Reviewer #2 also commented extensively but more generally on issues such as more detail needed on thiamine concentrations in adults, eggs and ducklings, whether this paper showed population effects, various assumptions in the model - again related to prey size selection/preference and selection - and the influence of brain size. In my opinion, the authors responded well to the comments of Reviewer #2.

My own comments, as noted, were minor, although I too had some questions about the mechanisms and the direction of prey size selection, how maternal thiamine concentrations might impact egg production ,and what might be known about the effects of thiamine deficiency on female behavior and survival (apparently little - most of what is known comes from studies on fishes, especially salmonids which differ considerably of course from a precocial long-lived sea duck). I also wondered about how variants of their life history model (e.g. sub-optimal responses) and assumptions of the model might lead to different outcomes.

Overall, I think the author did a good job addressing all the comments of Reviewer #2 and my comments for the most part. The authors also addressed or rebutted the comments of Reviewer #1, although there's still room for debate and discussion on several issues and assumptions; I suspect that Reviewer #1 might not fully agree with all the author's responses.
Response #53: We would like to thank again Reviewer 3 for summarizing our responses to the points raised by all Reviewers.

I have two suggestions for this manuscript before it's published. First, I think there could be a bit more discussion about the assumptions of their model and its possible limitations – including areas for future research. I appreciate that the authors have moderated their argument to include alternative views on the role of predation versus thiamine deficiency. These not need not be mutually exclusive - thiamine deficiency could certainly reduce a duckling or a female's ability to

detect or evade predators and hence expose them to greater predation risk; the two processes could act together, perhaps magnifying sublethal effects of thiamine deficiency. I did appreciate that the authors, in response to Reviewer #2, reduced the brain size- prey selection idea as a motivation for the study and moved those ideas to the discussion. I had some doubts as well about that relationship as a driver.

Response #54: We have now added a paragraph discussing the most important limitations of our model in the context of the assumptions made. We also briefly outline future directions for model development (see L451-462).

My second suggestion concerns the debate about the relative impact of thiamine deficiency on Baltic eider production and population decline, which appears to be quite controversial. Reviewer #1clearly has much expertise in this area and strong opinions which did not fully coincide with the authors of this paper. I appreciated those concerns. Nonetheless, I do feel that publication of this paper is worthwhile because the authors are not claiming a direct effect of thiamine deficiency on Baltic eiders, but are demonstrating the feasibility of an effect through their model. I think that's helpful. Having said that, I also think it's worthwhile to consider alternative viewpoints and what might make this a more interesting and open discussion is to suggest that Reviewer #1 write a commentary or opinion piece to present some of the logic and citations from their review. My sense is that this is exactly what PLoS One seeks to do – to publish well-reasoned and scientifically credible papers - even if those papers might not agree with all schools of thought. Having Reviewer #1present an alternative published perspective would enhance thoughtful and careful critical examination of these ideas.

Response #55: Thank you for your comment, and for providing a valuable roadmap on how to proceed with these different approaches. We fully support Reviewer 3 suggestion for Reviewer 1 to write a commentary or opinion piece. We are certain that clearly presented logic behind the marginalization of some of the existing literature (see Response #1) would help to move the discussion forward.

I did not receive the supplemental material, so I did not review that, but I appreciate that the authors added more material to the supplement that address some several of the comments and concerns of all reviewers as well as considering alternative scenarios of clutch size, migration and timing of breeding.

John M. Eadie
Distinguished Professor emeritus
D. G. Raveling Endowed Chair emeritus
Dept. of Wildlife, Fish & Conservation Biology
UC Davis

---

7. PLOS authors have the option to publish the peer review history of their article (what does this mean?). If published, this will include your full peer review and any attached files.

If you choose “no”, your identity will remain anonymous but your review may still be made public.

Do you want your identity to be public for this peer review? For information about this choice, including consent withdrawal, please see our Privacy Policy.

Reviewer #1: No

Reviewer #2: No

Reviewer #3: No

[NOTE: If reviewer comments were submitted as an attachment file, they will be attached to this email and accessible via the submission site. Please log into your account, locate the manuscript record, and check for the action link "View Attachments". If this link does not appear, there are no attachment files.]

To ensure your figures meet our technical requirements, please review our figure guidelines: https://journals.plos.org/plosone/s/figures

You may also use PLOS’s free figure tool, NAAS, to help you prepare publication quality figures: https://journals.plos.org/plosone/s/figures#loc-tools-for-figure-preparation.

NAAS will assess whether your figures meet our technical requirements by comparing each figure against our figure specifications.
