# Supplementary material for: The risk of thiamine deficiency in common eider ducklings increases when females consume large prey
Source: PLoS One. 2026 Sep 16;21(9):e0356777. doi: 10.1371/journal.pone.0356777 (PMC13580947; doi:10.1371/journal.pone.0356777)
Supplement: Supplementary file 8 [file pone.0356777.s008.pdf]

Dear Editors of PLOS One,

Thank you for evaluating our manuscript and for providing a thorough review. We have revised the manuscript based on the reviewers' suggestions. Our responses are provided below, alongside an explanation of the changes we have made. For your convenience, our responses are in dark blue and refer to the locations in the manuscript where changes have been made. Note that the citations and reference list have been formatted in accordance with the PLOS One guidelines (not marked). Please do not hesitate to contact us if further changes are required.

Yours sincerely,
Maciej Ejsmond
On behalf of the authors

PONE-D-25-50326R2

The risk of thiamine deficiency in common eider ducklings increases when females consume large prey

PLOS One

Dear Dr. Ejsmond,

Thank you for submitting your manuscript to PLOS ONE. After careful consideration, we feel that it has merit but does not fully meet PLOS ONE's publication criteria as it currently stands. Therefore, we invite you to submit a revised version of the manuscript that addresses the points raised during the review process.

Please submit your revised manuscript by Sep 03 2026 11:59PM. If you will need more time than this to complete your revisions, please reply to this message or contact the journal office at plosone@plos.org. When you're ready to submit your revision, log on to https://www.editorialmanager.com/pone/ and select the 'Submissions Needing Revision' folder to locate your manuscript file.

Please include the following items when submitting your revised manuscript:

- A letter that responds to each point raised by the academic editor and reviewer(s). You should upload this letter as a separate file labeled 'Response to Reviewers'.
- A marked-up copy of your manuscript that highlights changes made to the original version. You should upload this as a separate file labeled 'Revised Manuscript with Track Changes'.
- An unmarked version of your revised paper without tracked changes. You should upload this as a separate file labeled 'Manuscript'.

If you would like to make changes to your financial disclosure, please include your updated statement in your cover letter. Guidelines for resubmitting your figure files are available below the reviewer comments at the end of this letter.

If applicable, we recommend that you deposit your laboratory protocols in protocols.io to enhance the reproducibility of your results. Protocols.io assigns your protocol its own identifier (DOI) so that it can be cited independently in the future. For instructions see: https://journals.plos.org/plosone/s/submission-guidelines#loc-laboratory-protocols. Additionally, PLOS ONE offers an option for publishing peer-reviewed Lab Protocol articles, which describe protocols hosted on protocols.io. Read more information on sharing protocols at https://plos.org/protocols?utm_medium=editorial-email&utm_source=authorletters&utm_campaign=protocols.

As the corresponding author, your ORCID iD is verified in the submission system and will appear in the published article. PLOS supports the use of ORCID, and we encourage all coauthors to register for an ORCID iD and use it as well. Please encourage your coauthors to verify their ORCID iD within the submission system before final acceptance, as unverified ORCID iDs will not appear in the published article. *Only* the individual author can complete the verification step; PLOS staff *cannot* verify ORCID iDs on behalf of authors.

We look forward to receiving your revised manuscript.

Kind regards,

Juan J Loor
Academic Editor
PLOS One

Journal Requirements:

1. If the reviewer comments include a recommendation to cite specific previously published works, please review and evaluate these publications to determine whether they

are relevant and should be cited. There is no requirement to cite these works unless the editor has indicated otherwise.

2. Please review your reference list to ensure that it is complete and correct. If you have cited papers that have been retracted, please include the rationale for doing so in the manuscript text, or remove these references and replace them with relevant current references. Any changes to the reference list should be mentioned in the rebuttal letter that accompanies your revised manuscript. If you need to cite a retracted article, indicate the article's retracted status in the References list and also include a citation and full reference for the retraction notice.

[Note: HTML markup is below. Please do not edit.]

Reviewers' comments:

Reviewer's Responses to Questions

Comments to the Author

1. If the authors have adequately addressed your comments raised in a previous round of review and you feel that this manuscript is now acceptable for publication, you may indicate that here to bypass the "Comments to the Author" section, enter your conflict of interest statement in the "Confidential to Editor" section, and submit your "Accept" recommendation.

Reviewer #2: (No Response)

Reviewer #3: All comments have been addressed

---

2. Is the manuscript technically sound, and do the data support the conclusions?

The manuscript must describe a technically sound piece of scientific research with data that supports the conclusions. Experiments must have been conducted rigorously, with appropriate controls, replication, and sample sizes. The conclusions must be drawn appropriately based on the data presented.

Reviewer #2: Yes

Reviewer #3: Yes

3. Has the statistical analysis been performed appropriately and rigorously?

Reviewer #2: Yes

Reviewer #3: Yes

---

4. Have the authors made all data underlying the findings in their manuscript fully available?

The PLOS Data policy requires authors to make all data underlying the findings described in their manuscript fully available without restriction, with rare exception (please refer to the Data Availability Statement in the manuscript PDF file). The data should be provided as part of the manuscript or its supporting information, or deposited to a public repository. For example, in addition to summary statistics, the data points behind means, medians and variance measures should be available. If there are restrictions on publicly sharing data—e.g. participant privacy or use of data from a third party—those must be specified.

Reviewer #2: Yes

Reviewer #3: Yes

---

5. Is the manuscript presented in an intelligible fashion and written in standard English?

PLOS ONE does not copyedit accepted manuscripts, so the language in submitted articles must be clear, correct, and unambiguous. Any typographical or grammatical errors should be corrected at revision, so please note any specific errors here.

Reviewer #2: Yes

Reviewer #3: Yes

---

6. Review Comments to the Author

Please use the space provided to explain your answers to the questions above. You may also include additional comments for the author, including concerns about dual publication, research ethics, or publication ethics. (Please upload your review as an attachment if it exceeds 20,000 characters)

Reviewer #2: I thank the Authors for their revision of the manuscript.
Response #1: We appreciate the time and effort you put into reviewing our work.

I only have a few minor comments:
- L. 202-207: There is a slight discrepancy in the methods that needs to be revised. The authors mention "offspring recruitment $\gamma(t)$" and in the following sentence "offspring value $\gamma(t)$", which one is it? Later the offspring value seems to be md. Additionally, the authors refer to $\gamma(t)$ and give a formula for $\gamma(i)$ which may be a bit misleading for readers. Please also check that l. 335: "contribution of offspring to fitness (γ)" is the right wording.
Response #2: Thank you for your comment. We have standardized the terminology of γ throughout the manuscript (L178, L277, L437).

- L. 388: Change "large headed" to "large-headed"
Response #3: Corrected (L321).

- L. 453: "complicates" or "complicated"? (add s or d?)
Response #4: Corrected (L380).

Reviewer #3: I previously reviewed this manuscript twice (this is the 3rd time) and I feel that the authors have addressed my concerns, and many of those of the other reviewers. My initial comments were minor. The few small remaining issues, for me, have mostly been addressed in this second revisions (but see my comment below on the possibility that thiamine-deficiency might predispose eiders to greater predation risk). Reviewer #2's concerns also appear to have been addressed.
Response #5: We would like to express our gratitude to Reviewer #3 for their efforts in improving our manuscript. We have added a description of the hypothesis (see Response #8).

Reviewer #1 still has several extensive comments. My impression is that there remains a very strong difference of opinion of Reviewer #1 and the authors on the impact of thiamine-deficiency vs. predation on declines of Baltic common eiders. The authors have made a sincere attempt to address most of Reviewer #1 concerns; the remaining issues are worthy of discussion, but I don't see a clear resolution. Accordingly, I defer to my suggestion in my previous review to encourage the journal to offer Reviewer #1 an opportunity to publish a commentary. (I would hope this would not just be a rebuttal or a point-by-point critique of this paper, but rather a comprehensive evaluation the evidence for or against the thiamine-deficiency vs. predation hypotheses).
Response #6: Thank you for your comment. We agree with the suggestion made by

Reviewer #3.

For my own ease of mind, I looked closely at the five main concerns of Reviewer #1. They were detailed and thoughtful, and I needed to be convinced that the latest revision dealt with these issues as reasonably as possible. My impression is that it has. In quick summary, Reviewer #1 raised five issues on the first revision:

(1) The first issue was the framing of the study and whether there was sufficient justification for thiamine- deficiency to be a factor in the decline of Baltic eider.s I agree with the authors that there is evidence from Balk et al. (2009, 2016) and Mörner et al.(2017), as well as papers on variety of other species of fishes and wildlife, suggesting that thiamine-deficiency could be a cause for concern. Hence, I think there's sufficient justification to explore this further. (Note that the authors also cite Svedäng et al. (2018) in their rebuttal and text, but that paper is not included in their reference list).

Response #7: Thank you for your comment. There was indeed a problem with the line spacing and line breaks, which made the reference to Svedäng et al. (2018) difficult to find. See line 534. Note, the reference list has been formatted in accordance with the PLOS One guidelines.

I note that this revision did not consider my suggestion of a third hypothesis – i.e., an interaction between thiamine-deficiency and predation such that females or ducklings experiencing a thiamine deficiency would be more susceptible to predation. I noted this in my review of the MS and the authors responded that they dealt with this on lines 451-462. However, that paragraph deals only with modelling of micronutrient allocation in a life history context, mechanisms of prey size selection, and the potential seasonal variation of thiamine content as future research directions. The possibility that thiamine-deficiency AND predation could BOTH be interacting should be mentioned - it represents a viable alternative hypothesis that does not pit one explanation (predation) against the other (thiamine-deficiency). (I get an impression that there is a tendency by both these authors and Reviewer #1 to promote one hypothesis over the other). A sentence or two in the paragraph on lines 452-462 would be useful in addressing my comment from that last revision.

Response #8: We apologize for omitting a comment by Reviewer #3 in the previous review report. We agree that predation and thiamine deficiency can interact. We have now added a description of the hypothesis proposed by Reviewer #3. We agree with reviewer's suggestions that this comment is particularly relevant to population trends, and the description has been included earlier in the discussion (see lines 325–332) than the reviewer suggested.

(2) A second issue for Reviewer #1 was the mechanistic cause of mussel size-thiamine relationship. This does appear to be uncertain at this point and clearly needs to be worked on more. The authors have expanded their consideration of alternative mechanisms (albeit briefly); Nonetheless, as the authors point out in this revision and their response, their model simply assumes a correlation between mussel size and thiamine. It does not specify a mechanistic cause. Given that, I think it's fine as they have currently employed it. It does leave open a pathway for future research as they note in the discussion.

(3) Reviewer #1 had several concerns over the biological realism of the model. I appreciate those concerns as well as some of Reviewer #2's, especially regarding determinants of clutch size, body reserves, and timing of breeding. In this latest revision, the authors have undertaken additional sensitivity analyses, and it appears that their results are fairly robust. This was a modeling exercise, in any case, and one can't include every variation, so I personally feel it is reasonable to proceed - as long as there is support for the assumptions being made, the range of parameter values used, and the relationships being modeled, even where there is uncertainty. Future versions of the model could explore some of the remaining issues.

(4) Reviewers #1 and 2 both requested that the statistical reporting be more explicit and less dispersed, and the current revision does so.

Response #9: Thank you for appreciating our work on the points described in (2), (3) and (4).

(5) Finally, Reviewer #1 felt that the discussion still overextends inferences about prey size selection. The authors have addressed some of this in the final revision although mechanism of the prey size selection issue is still unresolved. The authors assumed that eiders chose a single fixed prey size with no variation, which they acknowledge is unrealistic but was necessary for the model. I wonder how reduced the effects of thiamine deficiency might be if eiders in the model were able to consume a broader range of prey sizes?

Response #10: Please note that our model considers variability in daily thiamine intake due to the stochastic variation in food consumption (see Fig 1D, L201–203). While the variance in thiamine intake with consumed food did not affect the main conclusion of our work (see Fig. S10 in S1 Appendix), for large variance in the distribution of consumed food, the distribution of thiamine levels in the eggs could overlap for scenarios with prey of a similar size (see Fig. S10F in S1 Appendix). The assumption that eiders consume mussels of various sizes would introduce similar variation in thiamine intake and broaden the

distribution of thiamine levels in the eggs. We believe that our assumption of a single, fixed prey size with no variation facilitates understanding of the model.

The authors consider Reviewer #1 suggestions that flesh-to-shell ratios and shell-processing profitability remain plausible alternatives. Still, for me, I was left wondering, if eiders actively select mussel sizes (and several papers suggest they do), what characteristics are they selecting for? This may be less important for the current study and model given (1) eiders do chose prey sizes for whatever reason, and (2) prey size impacts the levels of thiamine. How eiders choose prey remains interesting, but not critical to the concern that the size of prey consumed could influence thiamine concentrations (and other essential nutrients, etc.) in their diet, with possible reproductive consequences.
Response #11: Several aspects of our model require more in-depth and realistic analysis. One of these is prey-size selection. Please note that our model is based on several simplifying assumptions also with regard to life history and physiology (e.g. thiamine excretion). However, these simplifications were necessary; otherwise, the model would not be feasible for analysis and understanding. We believe that exploring these aspects requires a separate theoretical paper.

I don't mean to simply revisit all the issues raised by Reviewer #1, but their review of the revised manuscript was critical and thoughtful, and I found myself thinking carefully about whether the remaining concerns were addressed. I believe the authors have made a reasonable and extensive effort to do so. While not every issue is resolved, the manuscript provides a valuable and informative contribution to what appears to be an important debate about the factors influencing eider declines in the Baltic. In closing, I'll just restate my recommendation that Reviewer #1 be offered the opportunity to provide a commentary on the role of predation versus other factors impacting Baltic eiders. Such a discussion, if done thoughtfully, could be very valuable to future researchers and conservation managers.
Response #12: Thank you for your thorough analysis of the aspects of our work that were commented on by Reviewers #1 and #2, and for your analysis of the changes we have made to improve the work. We agree that Reviewer #1's opinion on the role of thiamine deficiency in the context of common eiders survival would be a valuable addition to the discussion on this species' population trends.

---

7. PLOS authors have the option to publish the peer review history of their article (what does this mean?). If published, this will include your full peer review and any attached files.

If you choose "no", your identity will remain anonymous but your review may still be made public.

Do you want your identity to be public for this peer review? For information about this choice, including consent withdrawal, please see our Privacy Policy.

Reviewer #2: No

Reviewer #3: No

[NOTE: If reviewer comments were submitted as an attachment file, they will be attached to this email and accessible via the submission site. Please log into your account, locate the manuscript record, and check for the action link "View Attachments". If this link does not appear, there are no attachment files.]

To ensure your figures meet our technical requirements, please review our figure guidelines: https://journals.plos.org/plosone/s/figures

You may also use PLOS's free figure tool, NAAS, to help you prepare publication quality figures: https://journals.plos.org/plosone/s/figures#loc-tools-for-figure-preparation.

NAAS will assess whether your figures meet our technical requirements by comparing each figure against our figure specifications.

In compliance with data protection regulations, you may request that we remove your personal registration details at any time. (Remove my information/details). Please contact the publication office if you have any questions.
