# Supplementary material for: The risk of thiamine deficiency in common eider ducklings increases when females consume large prey
Source: PLoS One. 2026 Sep 16;21(9):e0356777. doi: 10.1371/journal.pone.0356777 (PMC13580947; doi:10.1371/journal.pone.0356777)
Supplement: Peer review history

# Peer review: Decision Letter 0

**DOI:** 10.1371/journal.pone.0356777.r001

**Authors:** Juan J Loor

26 Nov 2025

Dear Dr. Ejsmond,

Thank you for submitting your manuscript to PLOS ONE. After careful consideration, we feel that it has merit but does not fully meet PLOS ONE’s publication criteria as it currently stands. Therefore, we invite you to submit a revised version of the manuscript that addresses the points raised during the review process.

Please submit your revised manuscript by Jan 10 2026 11:59PM. If you will need more time than this to complete your revisions, please reply to this message or contact the journal office at plosone@plos.org. When you're ready to submit your revision, log on to https://www.editorialmanager.com/pone/ and select the 'Submissions Needing Revision' folder to locate your manuscript file.

- A rebuttal letter that responds to each point raised by the academic editor and reviewer(s). You should upload this letter as a separate file labeled 'Response to Reviewers'.
- A marked-up copy of your manuscript that highlights changes made to the original version. You should upload this as a separate file labeled 'Revised Manuscript with Track Changes'.
- An unmarked version of your revised paper without tracked changes. You should upload this as a separate file labeled 'Manuscript'.

If you would like to make changes to your financial disclosure, please include your updated statement in your cover letter. Guidelines for resubmitting your figure files are available below the reviewer comments at the end of this letter.

If applicable, we recommend that you deposit your laboratory protocols in protocols.io to enhance the reproducibility of your results. Protocols.io assigns your protocol its own identifier (DOI) so that it can be cited independently in the future. For instructions see: https://journals.plos.org/plosone/s/submission-guidelines#loc-laboratory-protocols. Additionally, PLOS ONE offers an option for publishing peer-reviewed Lab Protocol articles, which describe protocols hosted on protocols.io. Read more information on sharing protocols at https://plos.org/protocols?utm_medium=editorial-email&utm_source=authorletters&utm_campaign=protocols.

We look forward to receiving your revised manuscript.

Kind regards,

Juan J Loor

Academic Editor

PLOS ONE

Journal requirements:

When submitting your revision, we need you to address these additional requirements.

1. Please ensure that your manuscript meets PLOS ONE's style requirements, including those for file naming. The PLOS ONE style templates can be found at

https://journals.plos.org/plosone/s/file?id=wjVg/PLOSOne_formatting_sample_main_body.pdf and

https://journals.plos.org/plosone/s/file?id=ba62/PLOSOne_formatting_sample_title_authors_affiliations.pdf

2. Thank you for stating the following financial disclosure:

“European Union’s Horizon Europe research and innovation programme under the Marie Skłodowska-Curie grant agreement (101126636) to ME.

Carl Trygger Foundation, The Swedish research council VR (grant no.: 2019-04251), and the Swedish research council FORMAS (FR-2020/0008) to SH and MH.

University of Iceland Recruitment Fund, grant no. 2900-293501 to AE.

The project MARBEFES of the European Union’s Horizon Europe Programme (Grant no. 101060937,project MARBEFES, www.marbefes.eu) to AW.

Wroclaw Centre for Networking and Supercomputing (http://wcss.pl), grant No. 576 to ME.”

Please state what role the funders took in the study.  If the funders had no role, please state: "The funders had no role in study design, data collection and analysis, decision to publish, or preparation of the manuscript."

If this statement is not correct you must amend it as needed.

Please include this amended Role of Funder statement in your cover letter; we will change the online submission form on your behalf.

3. Please update your submission to use the PLOS LaTeX template. The template and more information on our requirements for LaTeX submissions can be found at http://journals.plos.org/plosone/s/latex.

4. We note that Figure 1A in your submission contain copyrighted images. All PLOS content is published under the Creative Commons Attribution License (CC BY 4.0), which means that the manuscript, images, and Supporting Information files will be freely available online, and any third party is permitted to access, download, copy, distribute, and use these materials in any way, even commercially, with proper attribution. For more information, see our copyright guidelines: http://journals.plos.org/plosone/s/licenses-and-copyright.

We require you to either (1) present written permission from the copyright holder to publish these figures specifically under the CC BY 4.0 license, or (2) remove the figures from your submission:

1. You may seek permission from the original copyright holder of Figure 1A to publish the content specifically under the CC BY 4.0 license.

We recommend that you contact the original copyright holder with the Content Permission Form (http://journals.plos.org/plosone/s/file?id=7c09/content-permission-form.pdf) and the following text:

“I request permission for the open-access journal PLOS ONE to publish XXX under the Creative Commons Attribution License (CCAL) CC BY 4.0 (http://creativecommons.org/licenses/by/4.0/). Please be aware that this license allows unrestricted use and distribution, even commercially, by third parties. Please reply and provide explicit written permission to publish XXX under a CC BY license and complete the attached form.”

Please upload the completed Content Permission Form or other proof of granted permissions as an "Other" file with your submission.

In the figure caption of the copyrighted figure, please include the following text: “Reprinted from [ref] under a CC BY license, with permission from [name of publisher], original copyright [original copyright year].”

2. If you are unable to obtain permission from the original copyright holder to publish these figures under the CC BY 4.0 license or if the copyright holder’s requirements are incompatible with the CC BY 4.0 license, please either i) remove the figure or ii) supply a replacement figure that complies with the CC BY 4.0 license. Please check copyright information on all replacement figures and update the figure caption with source information. If applicable, please specify in the figure caption text when a figure is similar but not identical to the original image and is therefore for illustrative purposes only.

5. We note that Figure 1B in your submission contain [map/satellite] images which may be copyrighted. All PLOS content is published under the Creative Commons Attribution License (CC BY 4.0), which means that the manuscript, images, and Supporting Information files will be freely available online, and any third party is permitted to access, download, copy, distribute, and use these materials in any way, even commercially, with proper attribution. For these reasons, we cannot publish previously copyrighted maps or satellite images created using proprietary data, such as Google software (Google Maps, Street View, and Earth). For more information, see our copyright guidelines: http://journals.plos.org/plosone/s/licenses-and-copyright.

We require you to either (1) present written permission from the copyright holder to publish these figures specifically under the CC BY 4.0 license, or (2) remove the figures from your submission:

1. You may seek permission from the original copyright holder of Figure 1B to publish the content specifically under the CC BY 4.0 license.

We recommend that you contact the original copyright holder with the Content Permission Form (http://journals.plos.org/plosone/s/file?id=7c09/content-permission-form.pdf) and the following text:

“I request permission for the open-access journal PLOS ONE to publish XXX under the Creative Commons Attribution License (CCAL) CC BY 4.0 (http://creativecommons.org/licenses/by/4.0/). Please be aware that this license allows unrestricted use and distribution, even commercially, by third parties. Please reply and provide explicit written permission to publish XXX under a CC BY license and complete the attached form.”

Please upload the completed Content Permission Form or other proof of granted permissions as an "Other" file with your submission.

In the figure caption of the copyrighted figure, please include the following text: “Reprinted from [ref] under a CC BY license, with permission from [name of publisher], original copyright [original copyright year].”

2. If you are unable to obtain permission from the original copyright holder to publish these figures under the CC BY 4.0 license or if the copyright holder’s requirements are incompatible with the CC BY 4.0 license, please either i) remove the figure or ii) supply a replacement figure that complies with the CC BY 4.0 license. Please check copyright information on all replacement figures and update the figure caption with source information. If applicable, please specify in the figure caption text when a figure is similar but not identical to the original image and is therefore for illustrative purposes only.

The following resources for replacing copyrighted map figures may be helpful:

USGS National Map Viewer (public domain): http://viewer.nationalmap.gov/viewer/

The Gateway to Astronaut Photography of Earth (public domain): http://eol.jsc.nasa.gov/sseop/clickmap/

Maps at the CIA (public domain): https://www.cia.gov/library/publications/the-world-factbook/index.html and https://www.cia.gov/library/publications/cia-maps-publications/index.html

NASA Earth Observatory (public domain): http://earthobservatory.nasa.gov/

Landsat: http://landsat.visibleearth.nasa.gov/

USGS EROS (Earth Resources Observatory and Science (EROS) Center) (public domain): http://eros.usgs.gov/#

Natural Earth (public domain): http://www.naturalearthdata.com/

6. Please include captions for your Supporting Information files at the end of your manuscript, and update any in-text citations to match accordingly. Please see our Supporting Information guidelines for more information: http://journals.plos.org/plosone/s/supporting-information.

If the reviewer comments include a recommendation to cite specific previously published works, please review and evaluate these publications to determine whether they are relevant and should be cited. There is no requirement to cite these works unless the editor has indicated otherwise.

[Note: HTML markup is below. Please do not edit.]

Reviewers' comments:

Reviewer's Responses to Questions

**Comments to the Author**

1. Is the manuscript technically sound, and do the data support the conclusions?

Reviewer #1: No

Reviewer #2: Yes

Reviewer #3: Yes

**********

2. Has the statistical analysis been performed appropriately and rigorously?

Reviewer #1: N/A

Reviewer #2: No

Reviewer #3: Yes

**********

3. Have the authors made all data underlying the findings in their manuscript fully available?

The PLOS Data policy

Reviewer #1: Yes

Reviewer #2: Yes

Reviewer #3: Yes

**********

4. Is the manuscript presented in an intelligible fashion and written in standard English?

Reviewer #1: Yes

Reviewer #2: Yes

Reviewer #3: Yes

**********

Reviewer #1: My Reviewer Comments to the Author are provided as a separate file, as they exceed the length limit (48 076 characters with spaces).

I have no concerns about dual publication, research ethics, or publication ethics.

Reviewer #2: Comments to the Authors:

In this manuscript, the authors rely on a modelling approach to assess how prey size selection affects adult and egg thiamine levels in common eiders. They find that selection for larger prey (here, mussels) leads to lower thiamine concentrations in female eiders and their eggs/ducklings.

While I find the manuscript well-written and I like the modelling approach of female eider life histories, I have some concerns regarding the validity of some of the assumptions made in the models and therefore the results and conclusions of the study (see details in comments below). One of my main issues in the current version of the manuscript is the lack of link between this study and field- or lab-based knowledge of thiamine concentrations in the wild.

Major comments:

1. How reliable are the results of this study? I like the modelling approach of the study and the fact that the authors quantified thiamine levels in mussels at wintering, stopover and breeding locations. However, it is not clear whether concentrations in adults and eggs/ducklings reflect concentrations that would be found in the wild. I believe that some studies have been carried out to monitor thiamine levels in birds (including in eider eggs). It seems crucial that the authors mention these studies (e.g., in the discuss) and compare the thiamine levels in their models to the ones measured in the field. This would greatly improve the reliability of the findings and conclusions of the study.

2. The title of the manuscript as well as some of the content of the manuscript highlight an interest in assessing how selection for large prey will increase the risk of mass juvenile mortality (mediated by thiamine deficiency). To properly assess or discuss the risk of mass juvenile mortality, it seems important to know the levels at which thiamine is considered to be deficient. At what concentrations are impacts on birds expected (e.g. symptoms of neural dysfunction, impaired behaviour, locomotion…)? Maybe adults or eggs have lower thiamine levels when females prey on bigger mussels, but if levels are high enough it may not be constraining and may not have detrimental consequences. Could egg hatchability and juvenile mortality risk be evaluated in this study?

3. Model assumptions. I am not an expert in thiamine and I am wondering what we really know about thiamine concentrations in birds. Is there knowledge of how maternal transfer in the egg works? The model is based on the assumption that “thiamine egg levels reflect the thiamine concentration in the body of the female at laying initiation”. How realistic is that? Has there been any study measuring thiamine in females and eggs to validate this assumption? Could thiamine be deposited differently in the first vs the other eggs (as it can be seen in maternal transfer of contaminants for example)? Given that one would expect low levels of thiamine to have an effect on duckling mortality, I wonder what the same levels would have on adult females (if egg thiamine concentration reflects female thiamine concentration then could the same impact be expected on female health)?

4. L.70-93. In the introduction, the authors put the emphasis on brain size and its link with predation risk and mussel size preference. While I get the idea, I think that this would be more interesting in the discussion section rather than in the introduction section. Putting the emphasis on prey size preference by itself (without necessarily linking it to brain size) would make the manuscript more attractive to a broader audience – especially because the authors test an effect of prey size preference per se, rather than brain size.

5. Results on thiamine mass-specific levels in the soft tissues of blue mussels (l. 218 and after). I think that this section should be improved according to the following points. Could the authors please specify in the methods what statistical tests they used to produce the results. It would be great for the reader to have access to the average thiamine levels in the three areas (for an average mussel mass/size) and know if they statistically differ. Is the decrease in thiamine concentration with mussel size significant, and does the rate of decrease significantly differ at the three locations?

6. Maybe I missed this information in the methods, but how did the authors model prey size preference? Was each individual modelled as having a preference for a given prey size (e.g., 3cm) and foraging mussels of this exact size or a size following a normal distribution centered around 3cm? I am surprised that in all the figures egg thiamine levels hardly ever overlap between prey size groups.

Minor comments:

• Title: I think that it would be more transparent for readers to mention in the title that this manuscript is based on modelling rather than analysis of empirical data. For example, the mention “simulation-based model” could be added to the title.

• Title: the authors emphasize the risk of mass juvenile mortality but is it really what is assessed in the manuscript (see major comment 2)?

• L. 32-34: I am not sure why the authors draw a specific link with “nest predation”. This would mean that either thiamine deficiency has an effect on the behaviour of adult females and makes them less good at protecting their nest from predators, or that thiamine deficiency reduces hatchability of eggs. In precocial species such as eiders, the wording nest predation refers to egg predation and not duckling predation, as they usually leave the nest within 24h. Please make it clearer to the reader.

• L.30-34: Overall I find the reasoning and the transition between the two sentences hard to follow. Could the authors please rewrite them to make it easier for the reader to follow?

• L. 35: Selection for larger prey may not be entirely driven by brain size. I think that the authors would benefit from making the statement broader as what is important is that some individuals may select for larger prey and that this choice can potentially lead to thiamine deficiency and have negative impacts on the population. Making the statement more general will make it of interest for a broader audience (also see major comment 4).

• L.40: give the scientific name of blue mussels

• L. 41: does it reduce the levels in eggs because it reduces the levels in the female? Maybe this is worth mentioning as the authors say l. 37 that they are also interested in adults.

• L. 44: when reading the abstract, it is hard to understand why the authors find that clutch size reduction increased thiamine levels in the eggs. Could this result only be driven by some constraints of the model (i.e., model assumptions within the modelling framework)?

• L. 44-46: I guess that this is based on assumptions from brain size, which could be clarified as what the results really show is that females selecting larger prey are more likely to produce offspring with insufficient thiamine levels. I think that stating in the abstract that the authors find that females selecting larger prey are more likely to produce offspring with insufficient thiamine levels is what is the most important (for the reader)

• L. 58: To aid readers unfamiliar with thiamine, could the authors briefly explain the mechanism by which thiamine deficiency leads to early mortality? Why do juveniles die if they lack thiamine? Is it because they become more vulnerable to predation or because of impairment of essential functions within the organism (or both)? Can thiamine deficiency induce embryo mortality in the egg as well?

• L. 66: add the scientific name of common eiders in the main text.

• L. 72: write “one third” instead of 1/3

• L. 73: the word “duckling” should be preferred over “nestling” as eiders are precocial (unless the authors want to specifically refer to the stage when they have hatched and have not left the nest yet).

• L. 77: the authors mention predation of nests and incubating females, please also add duckling predation (once they have left the nest) as it is definitely very high too.

• L.91-93: The sentence is misleading, I don’t think that the preference for large prey itself is what is adaptive, per se, under increased predation risk.

• L. 99-101: “Our work showed that relatively small shifts in prey size selection towards larger mussels can significantly reduce the thiamine concentration in the produced eggs.” this is a result and should be in the result/discussion sections rather than in the introduction.

• L. 108-109: how were these areas defined? Please note that they are not representative of the entire Baltic Sea eider population and this should be discussed somewhere in the manuscript. Also, please show the exact location of mussel collection in fig 1.B.

• L. 108-109: Based on the appendix, it seems that all mussels were sampled in June. Is it known whether thiamine concentrations show seasonal variation in mussel? Are measurements in June reliable to infer thiamine concentration in the wintering grounds?

• L. 108-109: Based on the appendix, it seems that measurements from different areas are pooled in “wintering” and “stopover”. Did the authors account for that when assessing the decrease in thiamine with mussel size?

• L. 119: “We modelled the seasonal thiamine dynamics in common eider females and ducklings at hatching.”. What about thiamine dynamics/levels in egg? I am not sure if I have understood how thiamine levels are expected to vary between egg laying and hatching. Is it assumed to be constant from egg laying to hatching?

• L. 121 or l. 153: before laying, eiders can feed both in the wintering, stopover and breeding areas. Does the model allow females to feed on the breeding grounds?

• L. 132: what do we know about maternal transfer of thiamine (from the female to her eggs)? I think that this is something important to consider. Do all eggs produced by a given female have the same thiamine concentration?

• L. 144: how did the authors define this stopover location? Do they rely on empirical data from a monitored eider population? If so, this would be important to mention in the manuscript, especially because the duration of spring migration stopovers can be very short in eiders in the Baltic Sea and I don t think that this area is a well-known stopover area for the entire Baltic Sea eider population.

• L. 149: is that for the arrival at the wintering location?

• L. 154: clutch size typically varies between 3 and 6 eggs in eiders in the Baltic Sea, why did the authors limit it to a maximum of five?

• L. 225: “The life history of the model females resembled the life cycle of the Baltic common eiders, with accumulation of body reserves preceding migration, egg laying and incubation (Fig 2A).” Do the authors have access to empirical data to support this statement?

• L.252-254: I think that the paper would benefit from introducing optimal foraging theory in the introduction.

• L. 294: The sentence seems incomplete, please check and correct it.

• L. 316: when referring to “mortality episodes in offspring” caused by thiamine deficiency in this sentence, are the authors still referring to ducklings or all species? Please clarify.

Reviewer #3: Review of PONE-D-25-50326. Selection for large prey increases risk of mass juvenile mortality due to thiamine deficiency

General comments

This is a very interesting manuscript. I was not aware of the severity of the thiamine deficiency issue or the reports of mass mortality of common eiders in the Baltic population. This paper provides an important new contribution by assessing how thiamine deficiencies could arise through prey size selection of blue mussels by common eiders. The authors show that there is a strong pattern of thiamine tissue concentration with respect to mussel size and this could have important ramifications not only for diet selection, but for thiamine deficiencies in eggs and ducklings that could increase juvenile mortality and contribute to the long-term decline of these populations.

The paper is based on analysis of thiamine tissue concentrations from a collection of mussels from the wintering, stopover, and breeding grounds, as well as a comprehensive modeling analysis using dynamic programming to derive optimal life histories and then using that to predict how prey size selection could impact survival and reproductive success in common eiders. I thought this was a novel approach and while it is based on predictive modeling and assumes that eiders do indeed follow optimal life histories (what if they don’t?), it clearly illustrates how diet selection could significantly impact juvenile mortality, and might have the greatest effect on larger females that would be otherwise expected to reproduce well.

I have only a few comments/suggestions. I was a bit confused because the paper describes large eiders as selecting larger mussels (lines 85-93), which makes sense (perhaps based on brain size), and one would expect that larger mussels provide a greater energetic value overall. Hence, the concern is that if selection for larger mussels reduces the amount of thiamine that females acquire and provide to their eggs, this could have negative consequences. However, later in the manuscript, the authors note that in experimental studies, common eiders typically select the smallest class of mussels (line2 70-271). So, I am a bit unclear about which direction is expect for prey size selection – larger or smaller? I could use a bit more clarification.

Following on this point, I can see how there might be selection for large mussel size based purely on energy content, and larger females (with larger brains) might be better able to capture the larger mussels. And I can also see how that, in turn, could directly impact clutch size simply because of the greater energy and nutrient content available for making more eggs. The energetic pathway is clear and well established.

It’s less clear to me how eiders would directly select for thiamine content (i.e. selecting mussel sizes for their thiamine content), and how that would directly or physiologically affect clutch size. Is there a direct physiological link between thiamine concentration and the production of eggs? Could it simply be that eiders that do select for small mussels are less able (energetically) to produce as many eggs (and so clutch sizes are smaller), but the unintended (and perhaps unselected for) consequence is that thiamine concentration is actually higher and so those birds end up doing better in terms of egg/juvenile survival. A sort of hidden physiological reversal of fortunes –> smaller prey size (but greater thiamine concentration) -> less energy -> smaller clutch sizes -> greater juvenile survival (due to higher thiamine concentration). The opposite would obtain for larger females selecting larger prey. I understand that this is what the authors are describing. My point was simply that I found it hard to imagine how eiders might be actively adjusting selection of prey sizes based on thiamine concentration – what is the mechanism by how they would assess that or how that would directly influence the production of eggs. OK – just some musings.

I wondered what the outcome of the model would be in females did not follow the optimal life history derived from the dynamic state-variable model? Might thiamine deficiency also influence the timing of breeding or seasonal resource allocation by females such the impacts are even greater or different? Perhaps a comment on this in the discussion. It might also be useful to consider any other uncertainties or assumptions of the model that might impact the outcomes (perhaps as avenues for future work).

Fig 1 shows the range of mussel sizes in each location, but it might also be useful to add a plot of the overall frequency distribution of mussel sizes in the samples collected. It appears from the scatterplots in Fig. 1B that most mussels were <3 cm and many <2cm. So, an eider simply foraging at random might choose mussels that are in the higher range of thiamine concentrations than had they selected mussels >3 cm (Figure 2C).

I am not a physiologist, nor do I know very much about thiamine accumulation, metabolism, reabsorption ,or excretion, and it would be worthwhile to have a physiologist evaluate the rates of thiamine intake and loss that were used in the model. However, to my uninformed eye, the analyses and assumptions made appear thorough, well-supported and thoughtful.

I have a few other smaller line-item comments below, but overall, this is a valuable and important contribution. I hope my comments are useful.

Specific comments

Lines 86-87. Is this just a function of larger brain size or overall larger body size as well? Did the Laursen and Moller 2021 paper control for body size?

Lines 113. Did each sample comprise mussels of varying sizes? Could you add another plot summarizing the frequency distribution of mussel sizes samples from each location (perhaps not necessary since this can be inferred from the scatterplots in Fig. 1B but a summary frequency distribution would be helpful.).Could differences in the availability of mussel size classes among locations or seasons also impact diet patterns and hence prey size selection and thiamine concentrations in the diet?

Lines 128-129. This is very reasonable but what if all or some females did not follow the optimal life history derived from the dynamic state-variable model? As noted above, might thiamine deficiency also influence the timing of breeding or resource allocation by females such the impacts of thiamine deficiency are even greater, or different?

Lines 221-224. Why the differences in the R2 values among locations/season? Thoughts?

Lines 238. I wonder if a small table listing the parameter, their symbols and brief description could be included in the text. It would help a reader as one reads through the results on the effects of different parameter values and rate coefficients.

Lines line 270-277 and . This is where I got a bit hung up on whether eiders generally select for larger or smaller mussels. And if they do select for smaller mussels, how would they assess thiamine content and how would that directly impact the number of eggs they lay. I can see how females might select prey based on handling and search times and energetic content, and that would directly translate into the energy available to produce eggs clutch sizes. I have a less clear understanding how females could select for thiamine content other than as an incidental effect of selecting for small sizes (perhaps for other reasons) and how that physiologically might limit clutch size. Maybe a bit more discussion on this idea.

Line 377. The lines for the two alternative scenarios of offspring value (solid blue and dotted blue) in Fig. 1C are hard to see against the green background. Maybe just use black lines?

**********

what does this mean?). If published, this will include your full peer review and any attached files.

If you choose “no”, your identity will remain anonymous but your review may still be made public.

**Do you want your identity to be public for this peer review?**  For information about this choice, including consent withdrawal, please see our Privacy Policy

Reviewer #1: No

Reviewer #2: No

Reviewer #3: **Yes:** John M. Eadie

**********

[NOTE: If reviewer comments were submitted as an attachment file, they will be attached to this email and accessible via the submission site. Please log into your account, locate the manuscript record, and check for the action link "View Attachments". If this link does not appear, there are no attachment files.]

To ensure your figures meet our technical requirements, please review our figure guidelines: https://journals.plos.org/plosone/s/figures

You may also use PLOS’s free figure tool, NAAS, to help you prepare publication quality figures: https://journals.plos.org/plosone/s/figures#loc-tools-for-figure-preparation.

NAAS will assess whether your figures meet our technical requirements by comparing each figure against our figure specifications.

Attachment Submitted filename: PONE-D-25-50326_Review.pdf

Attachment Submitted filename: Review PONE-D-25-50326.pdf

# Author comment: Author response to Decision Letter 1

**DOI:** 10.1371/journal.pone.0356777.r002

6 Mar 2026

Due to the large number of comments by reviewers, our responses are attached to the cover letter in the form of a 40-page report.

Attachment Submitted filename: 1 Responses to Reviewers.pdf

# Peer review: Decision Letter 1

**DOI:** 10.1371/journal.pone.0356777.r003

**Authors:** Juan J Loor

14 Apr 2026

Dear Dr. Ejsmond,

Thank you for submitting your manuscript to PLOS ONE. After careful consideration, we feel that it has merit but does not fully meet PLOS ONE’s publication criteria as it currently stands. Therefore, we invite you to submit a revised version of the manuscript that addresses the points raised during the review process.

Please submit your revised manuscript by May 29 2026 11:59PM. If you will need more time than this to complete your revisions, please reply to this message or contact the journal office at plosone@plos.org. When you're ready to submit your revision, log on to https://www.editorialmanager.com/pone/ and select the 'Submissions Needing Revision' folder to locate your manuscript file.

- A letter that responds to each point raised by the academic editor and reviewer(s). You should upload this letter as a separate file labeled 'Response to Reviewers'.
- A marked-up copy of your manuscript that highlights changes made to the original version. You should upload this as a separate file labeled 'Revised Manuscript with Track Changes'.
- An unmarked version of your revised paper without tracked changes. You should upload this as a separate file labeled 'Manuscript'.

If you would like to make changes to your financial disclosure, please include your updated statement in your cover letter. Guidelines for resubmitting your figure files are available below the reviewer comments at the end of this letter.

If applicable, we recommend that you deposit your laboratory protocols in protocols.io to enhance the reproducibility of your results. Protocols.io assigns your protocol its own identifier (DOI) so that it can be cited independently in the future. For instructions see: https://journals.plos.org/plosone/s/submission-guidelines#loc-laboratory-protocols. Additionally, PLOS ONE offers an option for publishing peer-reviewed Lab Protocol articles, which describe protocols hosted on protocols.io. Read more information on sharing protocols at https://plos.org/protocols?utm_medium=editorial-email&utm_source=authorletters&utm_campaign=protocols.

As the corresponding author, your ORCID iD is verified in the submission system and will appear in the published article. PLOS supports the use of ORCID, and we encourage all coauthors to register for an ORCID iD and use it as well. Please encourage your coauthors to verify their ORCID iD within the submission system before final acceptance, as unverified ORCID iDs will not appear in the published article. *Only*  the individual author can complete the verification step; PLOS staff *cannot*  verify ORCID iDs on behalf of authors.

We look forward to receiving your revised manuscript.

Kind regards,

Juan J Loor

Academic Editor

PLOS One

Journal Requirements:

If the reviewer comments include a recommendation to cite specific previously published works, please review and evaluate these publications to determine whether they are relevant and should be cited. There is no requirement to cite these works unless the editor has indicated otherwise.

[Note: HTML markup is below. Please do not edit.]

Reviewers' comments:

Reviewer's Responses to Questions

**Comments to the Author**

Reviewer #1: (No Response)

Reviewer #2: (No Response)

Reviewer #3: (No Response)

**********

2. Is the manuscript technically sound, and do the data support the conclusions?

Reviewer #1: Partly

Reviewer #2: Yes

Reviewer #3: Yes

**********

3. Has the statistical analysis been performed appropriately and rigorously?

Reviewer #1: Yes

Reviewer #2: Yes

Reviewer #3: Yes

**********

4. Have the authors made all data underlying the findings in their manuscript fully available?

The PLOS Data policy

Reviewer #1: Yes

Reviewer #2: Yes

Reviewer #3: Yes

**********

5. Is the manuscript presented in an intelligible fashion and written in standard English?

Reviewer #1: Yes

Reviewer #2: Yes

Reviewer #3: Yes

**********

Reviewer #1: My comments are too long to fit into this window, so I attach a separate file with my comments (PDF).

Reviewer #2: I thank the Authors for their thorough revision of the manuscript and I appreciate the way the authors have revised the manuscript. I think that this manuscript provides a good modelling framework that could be linked to empirical data in the future or extended to other molecules than thiamine.

I only have a few minor comments:

- Maybe I missed it but did the authors mention what software was used for statistical analyses and modelling? I think that it would be helpful for researchers wanting to re-use the code.

- l. 89: “rates scales” : “s” should be removed from one of the two words

- l. 201: I still find this part a bit hard to understand but I think that I have now understood that for each scenario the size of the mussel is fixed. Could the authors maybe write it as “Below, we present the results of five scenarios in which females foraged on mussels of fixed lengths: 1.5, 2, 2.5, 3 and 3.5cm”. As the rest of the modelling incorporates variation around parameters, I was also expected that for prey preference and I think it can help readers to be clear about it.

- l. 389: this sentence is long, and I suggest splitting it in two to improve readability (e.g., at the end of l. 389

- l. 390: I don t think that the word spawning is used for birds and I would use laying instead.

Reviewer #3: I've now read the revised manuscript and the comments of reviewer #1 and #2. Overall, I think the authors have responded well to the very detailed and helpful commentary by both reviewers, as well as my own. My comments were relatively minor. However, Reviewer #1 provided extensive feedback - in some cases critical of some of the results or conclusions of the paper. Specifically, Reviewer #1 was concerned with three issues, in my reading. First and foremost was disagreement about the effect and the degree of impact of thiamine deficiency on egg viability and duckling survival versus an alternative hypothesis of predation on ducklings driving the decline in Baltic eiders. It appears to be an issue of considerable debate, and I found it interesting although I knew little of this controversy prior to this manuscript. I'll comment a bit further on this below.

Second, Reviewer #1 had several comments/concerns about issues related to general life history traits of eiders, and their application in the dynamic state model. These ranged from issues about the cost of reproduction, determination of clutch size, capital versus income breeding, migration and breeding phenologies, and breeding strategies writ large. Third, Reviewer #1 had a variety of comments that I'll loosely classify as behavioral based, but specifically related to prey size selection and the mechanisms whereby selection for a certain size classes of mussels could lead to thiamine deficiencies, the relative role of brain size, the risk of predation on female behavior and timing of breeding, etc. The authors responded extensively to Reviewer #1’s comments and made several modifications to the paper, notably reducing some of the strong rhetoric in favor of the thiamine deficiency hypothesis.

Reviewer #2 also commented extensively but more generally on issues such as more detail needed on thiamine concentrations in adults, eggs and ducklings, whether this paper showed population effects, various assumptions in the model - again related to prey size selection/preference and selection - and the influence of brain size. In my opinion, the authors responded well to the comments of Reviewer #2.

My own comments, as noted, were minor, although I too had some questions about the mechanisms and the direction of prey size selection, how maternal thiamine concentrations might impact egg production ,and what might be known about the effects of thiamine deficiency on female behavior and survival (apparently little - most of what is known comes from studies on fishes, especially salmonids which differ considerably of course from a precocial long-lived sea duck). I also wondered about how variants of their life history model (e.g. sub-optimal responses) and assumptions of the model might lead to different outcomes.

Overall, I think the author did a good job addressing all the comments of Reviewer #2 and my comments for the most part. The authors also addressed or rebutted the comments of Reviewer #1, although there's still room for debate and discussion on several issues and assumptions; I suspect that Reviewer #1 might not fully agree with all the author's responses.

I have two suggestions for this manuscript before it's published. First, I think there could be a bit more discussion about the assumptions of their model and its possible limitations – including areas for future research. I appreciate that the authors have moderated their argument to include alternative views on the role of predation versus thiamine deficiency. These not need not be mutually exclusive - thiamine deficiency could certainly reduce a duckling or a female’s ability to detect or evade predators and hence expose them to greater predation risk; the two processes could act together, perhaps magnifying sublethal effects of thiamine deficiency. I did appreciate that the authors, in response to Reviewer #2, reduced the brain size- prey selection idea as a motivation for the study and moved those ideas to the discussion. I had some doubts as well about that relationship as a driver.

My second suggestion concerns the debate about the relative impact of thiamine deficiency on Baltic eider production and population decline, which appears to be quite controversial. Reviewer #1clearly has much expertise in this area and strong opinions which did not fully coincide with the authors of this paper. I appreciated those concerns. Nonetheless, I do feel that publication of this paper is worthwhile because the authors are not claiming a direct effect of thiamine deficiency on Baltic eiders, but are demonstrating the feasibility of an effect through their model. I think that's helpful. Having said that, I also think it's worthwhile to consider alternative viewpoints and what might make this a more interesting and open discussion is to suggest that Reviewer #1 write a commentary or opinion piece to present some of the logic and citations from their review. My sense is that this is exactly what PLoS One seeks to do – to publish well-reasoned and scientifically credible papers - even if those papers might not agree with all schools of thought. Having Reviewer #1present an alternative published perspective would enhance thoughtful and careful critical examination of these ideas.

I did not receive the supplemental material, so I did not review that, but I appreciate that the authors added more material to the supplement that address some several of the comments and concerns of all reviewers as well as considering alternative scenarios of clutch size, migration and timing of breeding.

John M. Eadie

Distinguished Professor emeritus

D. G. Raveling Endowed Chair emeritus

Dept. of Wildlife, Fish & Conservation Biology

UC Davis

**********

what does this mean?). If published, this will include your full peer review and any attached files.

If you choose “no”, your identity will remain anonymous but your review may still be made public.

**Do you want your identity to be public for this peer review?**  For information about this choice, including consent withdrawal, please see our Privacy Policy

Reviewer #1: No

Reviewer #2: No

Reviewer #3: No

**********

[NOTE: If reviewer comments were submitted as an attachment file, they will be attached to this email and accessible via the submission site. Please log into your account, locate the manuscript record, and check for the action link "View Attachments". If this link does not appear, there are no attachment files.]

To ensure your figures meet our technical requirements, please review our figure guidelines: https://journals.plos.org/plosone/s/figures

You may also use PLOS’s free figure tool, NAAS, to help you prepare publication quality figures: https://journals.plos.org/plosone/s/figures#loc-tools-for-figure-preparation.

NAAS will assess whether your figures meet our technical requirements by comparing each figure against our figure specifications.

Attachment Submitted filename: Reviewer1_PONE-D-25-50326_Review2.pdf

# Author comment: Author response to Decision Letter 2

**DOI:** 10.1371/journal.pone.0356777.r004

8 Jun 2026

Our responses to the reviewers are attached in a separate file called 'Responses to Reviewers'.

Attachment Submitted filename: 1 Responses to Reviewers Rev2.pdf

# Peer review: Decision Letter 2

**DOI:** 10.1371/journal.pone.0356777.r005

**Authors:** Juan J Loor

20 Jul 2026

Dear Dr. Ejsmond,

Thank you for submitting your manuscript to PLOS ONE. After careful consideration, we feel that it has merit but does not fully meet PLOS ONE’s publication criteria as it currently stands. Therefore, we invite you to submit a revised version of the manuscript that addresses the points raised during the review process.

Please submit your revised manuscript by Sep 03 2026 11:59PM. If you will need more time than this to complete your revisions, please reply to this message or contact the journal office at plosone@plos.org. When you're ready to submit your revision, log on to https://www.editorialmanager.com/pone/ and select the 'Submissions Needing Revision' folder to locate your manuscript file.

- A letter that responds to each point raised by the academic editor and reviewer(s). You should upload this letter as a separate file labeled 'Response to Reviewers'.
- A marked-up copy of your manuscript that highlights changes made to the original version. You should upload this as a separate file labeled 'Revised Manuscript with Track Changes'.
- An unmarked version of your revised paper without tracked changes. You should upload this as a separate file labeled 'Manuscript'.

If you would like to make changes to your financial disclosure, please include your updated statement in your cover letter. Guidelines for resubmitting your figure files are available below the reviewer comments at the end of this letter.

If applicable, we recommend that you deposit your laboratory protocols in protocols.io to enhance the reproducibility of your results. Protocols.io assigns your protocol its own identifier (DOI) so that it can be cited independently in the future. For instructions see: https://journals.plos.org/plosone/s/submission-guidelines#loc-laboratory-protocols. Additionally, PLOS ONE offers an option for publishing peer-reviewed Lab Protocol articles, which describe protocols hosted on protocols.io. Read more information on sharing protocols at https://plos.org/protocols?utm_medium=editorial-email&utm_source=authorletters&utm_campaign=protocols.

As the corresponding author, your ORCID iD is verified in the submission system and will appear in the published article. PLOS supports the use of ORCID, and we encourage all coauthors to register for an ORCID iD and use it as well. Please encourage your coauthors to verify their ORCID iD within the submission system before final acceptance, as unverified ORCID iDs will not appear in the published article. *Only*  the individual author can complete the verification step; PLOS staff *cannot*  verify ORCID iDs on behalf of authors.

We look forward to receiving your revised manuscript.

Kind regards,

Juan J Loor

Academic Editor

PLOS One

**Journal Requirements:**

1. If the reviewer comments include a recommendation to cite specific previously published works, please review and evaluate these publications to determine whether they are relevant and should be cited. There is no requirement to cite these works unless the editor has indicated otherwise.

2. Please review your reference list to ensure that it is complete and correct. If you have cited papers that have been retracted, please include the rationale for doing so in the manuscript text, or remove these references and replace them with relevant current references. Any changes to the reference list should be mentioned in the rebuttal letter that accompanies your revised manuscript. If you need to cite a retracted article, indicate the article’s retracted status in the References list and also include a citation and full reference for the retraction notice.

[Note: HTML markup is below. Please do not edit.]

Reviewers' comments:

Reviewer's Responses to Questions

**Comments to the Author**

Reviewer #2: (No Response)

Reviewer #3: All comments have been addressed

**********

2. Is the manuscript technically sound, and do the data support the conclusions?

Reviewer #2: Yes

Reviewer #3: Yes

**********

3. Has the statistical analysis been performed appropriately and rigorously?

Reviewer #2: Yes

Reviewer #3: Yes

**********

4. Have the authors made all data underlying the findings in their manuscript fully available?

The PLOS Data policy

Reviewer #2: Yes

Reviewer #3: Yes

**********

5. Is the manuscript presented in an intelligible fashion and written in standard English?

Reviewer #2: Yes

Reviewer #3: Yes

**********

Reviewer #2: I thank the Authors for their revision of the manuscript. I only have a few minor comments:

- L. 202-207: There is a slight discrepancy in the methods that needs to be revised. The authors mention “offspring recruitment γ(t)” and in the following sentence “offspring value γ(t)”, which one is it? Later the offspring value seems to be md. Additionally, the authors refer to γ(t) and give a formula for γ(i) which may be a bit misleading for readers. Please also check that l. 335: “contribution of offspring to fitness (γ)” is the right wording.

- L. 388: Change “large headed” to “large-headed”

- L. 453: “complicates” or “complicated”? (add s or d?)

Reviewer #3: I previously reviewed this manuscript twice (this is the 3rd time) and I feel that the authors have addressed my concerns, and many of those of the other reviewers. My initial comments were minor. The few small remaining issues, for me, have mostly been addressed in this second revisions (but see my comment below on the possibility that thiamine-deficiency might predispose eiders to greater predation risk). Reviewer #2’s concerns also appear to have been addressed.

Reviewer #1 still has several extensive comments. My impression is that there remains a very strong difference of opinion of Reviewer #1 and the authors on the impact of thiamine-deficiency vs. predation on declines of Baltic common eiders. The authors have made a sincere attempt to address most of Reviewer #1 concerns; the remaining issues are worthy of discussion, but I don’t see a clear resolution. Accordingly, I defer to my suggestion in my previous review to encourage the journal to offer Reviewer #1 an opportunity to publish a commentary. (I would hope this would not just be a rebuttal or a point-by-point critique of this paper, but rather a comprehensive evaluation the evidence for or against the thiamine-deficiency vs. predation hypotheses).

For my own ease of mind, I looked closely at the five main concerns of Reviewer #1. They were detailed and thoughtful, and I needed to be convinced that the latest revision dealt with these issues as reasonably as possible. My impression is that it has. In quick summary, Reviewer #1 raised five issues on the first revision:

(1) The first issue was the framing of the study and whether there was sufficient justification for thiamine- deficiency to be a factor in the decline of Baltic eider.s I agree with the authors that there is evidence from Balk et al. (2009, 2016) and Mörner et al.(2017), as well as papers on variety of other species of fishes and wildlife, suggesting that thiamine-deficiency could be a cause for concern. Hence, I think there’s sufficient justification to explore this further. (Note that the authors also cite Svedäng et al. (2018) in their rebuttal and text, but that paper is not included in their reference list).

I note that this revision did not consider my suggestion of a third hypothesis – i.e., an interaction between thiamine-deficiency and predation such that females or ducklings experiencing a thiamine deficiency would be more susceptible to predation. I noted this in my review of the MS and the authors responded that they dealt with this on lines 451-462. However, that paragraph deals only with modelling of micronutrient allocation in a life history context, mechanisms of prey size selection, and the potential seasonal variation of thiamine content as future research directions. The possibility that thiamine-deficiency AND predation could BOTH be interacting should be mentioned - it represents a viable alternative hypothesis that does not pit one explanation (predation) against the other (thiamine-deficiency). (I get an impression that there is a tendency by both these authors and Reviewer #1 to promote one hypothesis over the other). A sentence or two in the paragraph on lines 452-462 would be useful in addressing my comment from that last revision.

(2) A second issue for Reviewer #1 was the mechanistic cause of mussel size-thiamine relationship. This does appear to be uncertain at this point and clearly needs to be worked on more. The authors have expanded their consideration of alternative mechanisms (albeit briefly); Nonetheless, as the authors point out in this revision and their response, their model simply assumes a correlation between mussel size and thiamine. It does not specify a mechanistic cause. Given that, I think it’s fine as they have currently employed it. It does leave open a pathway for future research as they note in the discussion.

(3) Reviewer #1 had several concerns over the biological realism of the model. I appreciate those concerns as well as some of Reviewer #2’s, especially regarding determinants of clutch size, body reserves, and timing of breeding. In this latest revision, the authors have undertaken additional sensitivity analyses, and it appears that their results are fairly robust. This was a modeling exercise, in any case, and one can’t include every variation, so I personally feel it is reasonable to proceed - as long as there is support for the assumptions being made, the range of parameter values used, and the relationships being modeled, even where there is uncertainty. Future versions of the model could explore some of the remaining issues.

(4) Reviewers #1 and 2 both requested that the statistical reporting be more explicit and less dispersed, and the current revision does so.

(5) Finally, Reviewer #1 felt that the discussion still overextends inferences about prey size selection. The authors have addressed some of this in the final revision although mechanism of the prey size selection issue is still unresolved. The authors assumed that eiders chose a single fixed prey size with no variation, which they acknowledge is unrealistic but was necessary for the model. I wonder how reduced the effects of thiamine deficiency might be if eiders in the model were able to consume a broader range of prey sizes?

The authors consider Reviewer #1 suggestions that flesh-to-shell ratios and shell-processing profitability remain plausible alternatives. Still, for me, I was left wondering, if eiders actively select mussel sizes (and several papers suggest they do), what characteristics are they selecting for? This may be less important for the current study and model given (1) eiders do chose prey sizes for whatever reason, and (2) prey size impacts the levels of thiamine. How eiders choose prey remains interesting, but not critical to the concern that the size of prey consumed could influence thiamine concentrations (and other essential nutrients, etc.) in their diet, with possible reproductive consequences.

I don’t mean to simply revisit all the issues raised by Reviewer #1, but their review of the revised manuscript was critical and thoughtful, and I found myself thinking carefully about whether the remaining concerns were addressed. I believe the authors have made a reasonable and extensive effort to do so. While not every issue is resolved, the manuscript provides a valuable and informative contribution to what appears to be an important debate about the factors influencing eider declines in the Baltic. In closing, I’ll just restate my recommendation that Reviewer #1 be offered the opportunity to provide a commentary on the role of predation versus other factors impacting Baltic eiders. Such a discussion, if done thoughtfully, could be very valuable to future researchers and conservation managers.

**********

what does this mean?). If published, this will include your full peer review and any attached files.

If you choose “no”, your identity will remain anonymous but your review may still be made public.

**Do you want your identity to be public for this peer review?**  For information about this choice, including consent withdrawal, please see our Privacy Policy

Reviewer #2: No

Reviewer #3: No

**********

[NOTE: If reviewer comments were submitted as an attachment file, they will be attached to this email and accessible via the submission site. Please log into your account, locate the manuscript record, and check for the action link "View Attachments". If this link does not appear, there are no attachment files.]

To ensure your figures meet our technical requirements, please review our figure guidelines: https://journals.plos.org/plosone/s/figures

You may also use PLOS’s free figure tool, NAAS, to help you prepare publication quality figures: https://journals.plos.org/plosone/s/figures#loc-tools-for-figure-preparation.

NAAS will assess whether your figures meet our technical requirements by comparing each figure against our figure specifications.

# Author comment: Author response to Decision Letter 3

**DOI:** 10.1371/journal.pone.0356777.r006

5 Aug 2026

'Responses to Reviewers' has been submitted as a separate text file.

Attachment Submitted filename: 1 Responses to Reviewers Rev3.pdf

# Editor report: Decision Letter 3

**DOI:** 10.1371/journal.pone.0356777.r007

**Authors:** Juan J Loor

10 Aug 2026

The risk of thiamine deficiency in common eider ducklings increases when females consume large prey

PONE-D-25-50326R3

Dear Dr. Ejsmond,

We’re pleased to inform you that your manuscript has been judged scientifically suitable for publication and will be formally accepted for publication once it meets all outstanding technical requirements.

Within one week, you’ll receive an e-mail detailing the required amendments. When these have been addressed, you’ll receive a formal acceptance letter and your manuscript will be scheduled for publication.

An invoice will be generated when your article is formally accepted. Please note, if your institution has a publishing partnership with PLOS and your article meets the relevant criteria, all or part of your publication costs will be covered. Please make sure your user information is up-to-date by logging into Editorial Manager at Editorial Manager® and clicking the ‘Update My Information' link at the top of the page. For questions related to billing, please contact billing support.

If your institution or institutions have a press office, please notify them about your upcoming paper to help maximize its impact. If they’ll be preparing press materials, please inform our press team as soon as possible -- no later than 48 hours after receiving the formal acceptance. Your manuscript will remain under strict press embargo until 2 pm Eastern Time on the date of publication. For more information, please contact onepress@plos.org.

Kind regards,

Juan J Loor

Academic Editor

PLOS One

Additional Editor Comments (optional):

Reviewers' comments:

# Editor report: Acceptance letter

**DOI:** 10.1371/journal.pone.0356777.r008

**Authors:** Juan J Loor

PONE-D-25-50326R3

PLOS One

Dear Dr. Ejsmond,

I'm pleased to inform you that your manuscript has been deemed suitable for publication in PLOS One. Congratulations! Your manuscript is now being handed over to our production team.

At this stage, our production department will prepare your paper for publication. This includes ensuring the following:

* All references, tables, and figures are properly cited

* All relevant supporting information is included in the manuscript submission,

* There are no issues that prevent the paper from being properly typeset

You will receive further instructions from the production team, including instructions on how to review your proof when it is ready. Please keep in mind that we are working through a large volume of accepted articles, so please give us a few days to review your paper and let you know the next and final steps.

Lastly, if your institution or institutions have a press office, please let them know about your upcoming paper now to help maximize its impact. If they'll be preparing press materials, please inform our press team within the next 48 hours. Your manuscript will remain under strict press embargo until 2 pm Eastern Time on the date of publication. For more information, please contact onepress@plos.org.

You will receive an invoice from PLOS for your publication fee after your manuscript has reached the completed accept phase. If you receive an email requesting payment before acceptance or for any other service, this may be a phishing scheme. Learn how to identify phishing emails and protect your accounts at https://explore.plos.org/phishing.

If we can help with anything else, please email us at customercare@plos.org.

Thank you for submitting your work to PLOS One and supporting open access.

Kind regards,

PLOS ONE Editorial Office Staff

on behalf of

Dr. Juan J Loor

Academic Editor

PLOS One
